# Identification and expression analysis of MAPK cascade gene family in foxtail millet (*Setaria italica*)

**DOI:** 10.1080/15592324.2023.2246228

**Published:** 2023-08-16

**Authors:** Lu Zhang, Cheng Ma, Xin Kang, Zi-Qi Pei, Xue Bai, Juan Wang, Sheng Zheng, Teng-Guo Zhang

**Affiliations:** Laboratory of plant molecular physiology, College of Life Sciences, Northwest Normal University, Lanzhou, China

**Keywords:** Foxtail millet (*Setaria italica*), MAPK cascade, genome-wide identification, gene expression analysis, phylogenetic analysis

## Abstract

The mitogen-activated protein kinase (MAPK) cascade pathway is a highly conserved plant cell signaling pathway that plays an important role in plant growth and development and stress response. Currently, MAPK cascade genes have been identified and reported in a variety of plants including *Arabidopsis thaliana*, *Oryza sativa*, and *Triticum aestivum*, but have not been identified in foxtail millet (*Setaria italica*). In this study, a total of 93 MAPK cascade genes, including 15 *SiMAPKs*, 10 *SiMAPKKs* and 68 *SiMAPKKKs* genes, were identified by genome-wide analysis of foxtail millet, and these genes were distributed on nine chromosomes of foxtail millet. Using phylogenetic analysis, we divided the *SiMAPKs* and *SiMAPKKs* into four subgroups, respectively, and the *SiMAPKKKs* into three subgroups (Raf, ZIK, and MEKK). Whole-genome duplication analysis revealed that there are 14 duplication pairs in the MAPK cascade family in foxtail millet, and they are expanded by segmental replication events. Results from quantitative real-time PCR (qRT-PCR) revealed that the expression levels of most *SiMAPKs* and *SiMAPKKs* were changed under both exogenous hormone and abiotic stress treatments, with *SiMAPK3* and *SiMAPKK4–2* being induced under almost all treatments, while the expression of *SiMAPKK5* was repressed. In a nutshell, this study will shed some light on the evolution of MAPK cascade genes and the functional mechanisms underlying MAPK cascade genes in response to hormonal and abiotic stress signaling pathways in foxtail millet (*Setaria italica*).

## 1. Introduction

The mitogen-activated protein kinase (MAPK) cascade pathway is a widespread and highly conserved signaling module in eukaryotes that translates signals generated by receptors/sensors into cellular responses and thus regulates plant growth and development.^1^ The typical MAPK cascade pathway consists of three sequentially acting protein kinases: the MAP kinase kinase kinases (MAPKKKs), MAP kinase kinases (MAPKKs), and MAP kinases (MAPKs),^2^ among which MAPKKKs can be divided into three subfamilies, namely, MEKK, Raf, and ZIK.^3^ MAPKKKs are serine and threonine (Ser/Thr) protein kinases that activate MAPKKs by phosphorylating two serine or threonine residues in the S/T-XXXXX(3/5)-S/T motif.^4,5^ As a class of bispecific kinases, MAPKKs activated by MAPKKKs could activate MAPKs by phosphorylating serine and threonine residues in the *T*-(D/E)-Y motif.^5–7^ Activated MAPKs act on downstream effector proteins (such as transcription factors and protein kinases) or other signaling components to complete signal transduction and ultimately affect biological growth and development, metabolism, defense, and other pathways.^8,9^ At present, MAPK cascade family genes have been identified in most plants, including *Arabidopsis thaliana*,^5,7^ *Oryza sativa*,^10^ *Brachypodium distachyon*,^11^ *Zea mays*,^12^ *Triticum aestivum*,^13,14^ *Hordeum vulgare*,^15^ *Solanum lycopersicum*,^16^ *Brassica napus*,^17^ and *Gossypium spp*,^18^ etc. These studies provided a resourceful reference for further studies of the MAPK cascade pathway.

The MAPK cascade pathway is a highly conserved signaling pathway in higher plants, involves not only in cell division, apoptosis, and plant growth and development but also in plant responses to abiotic stresses.^19^ Studies showed that MPK6 involved in Arabidopsis post-embryogenic root development through auxin upregulation and cell division plane orientation,^20^ and MPK3 and MPK6 of Arabidopsis are also required for funicular pollen tube guidance.^21^ It has also been reported that Arabidopsis MEKK1 can interact directly with the senescence-related WRKY53 transcription factor on a protein level and can bind to its promoter, thereby regulating leaf senescence.^22^ It has been shown that overexpression of *SlMAPK1* enhances the drought resistance of tomato plants.^23^ In rice, OsMAPK3-OsbHLH002-OsTPP1 pathway is crucial for triggering cold tolerance, OsMAPK3 could interact with and phosphorylate the OsbHLH002 protein, and OsbHLH002/OsICE1 positively regulates cold signaling via targeting OsTPP1,^24^ and overexpression of *OsMAPK5* can enhance plant tolerance to drought, salt, and cold stresses.^25,26^ Tomato *SlMPK3* is a low-temperature stress response gene, and the overexpression of *SlMPK3* causes higher seed germination, longer root length, and stronger resistance to cold stress in transgenic tobacco.^27^ In maize, ZmMKK1 is a positive regulatory protein induced by high-salinity stress that improves salinity tolerance in plants.^28^ In addition, some MAPK cascade pathways that respond to abiotic stresses have been characterized, such as the AtMEKK1-AtMKK2/AtMEK1-AtMAPK4/AtMAPK6 pathway that enhances cold resistance and salt tolerance in *Arabidopsis thaliana*.^29^ MAPKKK18-MAPKK3 positively regulates tolerance to drought stress in *Arabidopsis thaliana*.^30^ OsMAPKKK63-OsMAPKK1-OsMAPK4 cascade can enhance salt tolerance in rice.^2^ And the MKK7/MKK9-MPK6 pathway has been implicated to play crucial roles in plant response to salt stress.^31^

As a pivotal component that links intracellular and extracellular signaling, the MAPK cascade has been widely reported to be involved in phytohormone anabolic and signaling pathways, etc. For instance, MPK3 could be induced by ABA in *Arabidopsis thaliana*.^32^ Tomato *SlMPK6–1* and *SlMPK6–2* have been demonstrated to be positive regulators of jasmonate (JA) biosynthesis and signaling pathways.^33^ Hydrogen peroxide (H_2_O_2_), a common secondary messenger in plants, often acts as a signaling regulator upstream or downstream of the MAPK signaling pathway, mediating signal transmission.^34^ An earlier study revealed that H_2_O_2_ was a potent activator of MAPKs in *Arabidopsis thaliana* leaf cells.^35^ and can activate the expression of MAPK cascade genes such as *MPK1/MPK2*, *MPK3/MPK6*.^35–37^

Foxtail millet (*Setaria italica*) is widely grown in China, Russia, India, Africa, and the Americas and has a long history of cultivation, making it one of the oldest food crops in the world.^38^ Foxtail millet is also a drought-tolerant and salt-tolerant crop with the characteristic of water saving, requiring much less water than maize and wheat, and therefore millet foxtail is often used as an important model species for stress biology research.^39–41^ MAPK cascade genes have been extensively studied in a variety of plants, but they have not been systematically identified in foxtail millet; therefore, in this study, we performed a comprehensive analysis of *Setaria italica* MAPKKKs, MAPKKs, and MAPKs by genome-wide screening and identification, including phylogenetic analysis, gene structure analysis, conserved motifs construction, chromosomal location, and promoter sequence analysis. The expression of foxtail millet *MAPKKs* and *MAPKs* under abiotic stresses such as salt, cold and drought, plant hormones such as abscisic acid (ABA), methyl jasmonate (MeJA), melatonin (MT), and exogenous H_2_O_2_ treatment were also studied by qRT-PCR. These data will pave the way for the identification and evolution of the MAPK cascade gene family in *Setaria italica* and provide valuable reference for further studies of the MAPK cascades.

## 2. Materials and methods

### 2.1. *Genome-wide identification of MAPK cascade genes in* Setaria italica

Download the whole-genome annotation information and sequence information of foxtail millet (*Setaria italica*) from the EnsemblPlants database (http://plants.ensembl.org/index.html), use the MAPK cascade proteins in *Arabidopsis thaliana* (TAIR database: https://www.arabidopsis.org/), *Brachypodium distachyon* (PlantGDA database: http://www.plantgdb.org/BdGDB/) and *Oryza sativa* (RGAP database: http://rice.uga.edu/) as templates (Tab. S1), and use the BLAST program in the TBtools software for preliminary alignment.^42^ The homologous MAPK cascade genes were retrieved from the whole-genome library of foxtail millet, and the secondary alignment was performed in the NCBI BLASTp program (https://blast.ncbi.nlm.nih.gov/). The conserved domains of the candidate MAPKs cascade proteins were identified in the Pfam database (http://pfam.xfam.org/), the NCBI CDD Search program (https://www.ncbi.nlm.nih.gov/cdd) and the SMART website (http://smart.embl-heidelberg.de/).^13,43^ Finally, redundant sequences and incomplete sequences were removed to identify the final candidate foxtail millet MAPK cascade genes. The protein theoretical molecular weight and isoelectric point (pI) of the MAPK cascade proteins were predicted by ExPASy (http://au.expasy.org/tools). Subcellular localization of the MAPK cascade proteins of foxtail millet was analyzed by ProtComp 9.0 (http://linux1.softberry.com/berry.phtml).^44,45^

### 2.2. Multiple sequence alignment and phylogenetic analysis

The protein sequences for MAPK cascade genes from foxtail millet were aligned by DNAMAN 9 software. Phylogenetic trees were constructed by MEGA 7 software with bootstrap of 1000 replicates (Neighboring Joining (NJ) method), and the resulting tree was edited in iTOL v6 website (https://itol.embl.de/).^46^ In addition, the names of foxtail millet MAPK cascade genes started with the abbreviation *Si* of the scientific name of *Setaria italica*, and they were numbered according to the homology between the MAPK cascade genes of *Setaria italica* and those of *Arabidopsis thaliana*, *Oryza sativa*, and *Brachypodium distachyon*.

### 2.3. Gene structure, conserved motifs, and cis-acting regulatory elements analysis

The structural information of MAPK cascade genes in foxtail millet was obtained from the Gff3 annotation file of *Setaria italica* genome, and visual statistical analysis was performed by TBtools software. Conserved motifs were analyzed by the MEME website (http://meme-suite.org/tools/meme); In addition, the upstream 2kb sequence of the MAPK cascade genes of foxtail millet was extracted, and the cis-acting regulatory elements were predicted by the PlantCARE database (http://bioinformatics.psb.ugent.be/webtools/plantcare/html/), and the above results were visualized and analyzed by TBtools software.

### 2.4. Chromosome localization and collinearity analysis

Based on the annotation information of the *Setaria italica* genome, the MapGene2Chromosom v2 tool (http://mg2c.iask.in/mg2c_v2.0/) was used to draw the chromosomal distribution map of the *SiMAPK* cascade genes. The colinearity of MAPK cascade genes within and between species was also analyzed using the MCScanX toolkit,^47^ and visual graphs were obtained using TBtools software. Nonsynonymous substitution rate (Ka), synonymous substitution rate (Ks) and Ka/Ks ratios were calculated by the TBtools software.^42^

### 2.5. RNA-Seq analysis

Transcript information of SiMAPK and SiMAPKK genes in different organs of foxtail millet was downloaded from the *Setaria italica* database (SIFGD: http://structuralbiology.cau.edu.cn/SIFGD/index.html), and the transcript level was defined as FPKM (Fragments Per Kilobase of exon per Million fragments mapped). The protein interaction networks of SiMAPKs and SiMAPKKs were analyzed by the STRING system (https://string-db.org/cgi).

### 2.6. Plant material and treatment

In this study, “Henggu 11” foxtail millet seeds were used as the experimental material (provided by Institute of Dry Farming, Academy of Agricultural and Forestry Sciences, Hebei, China). The seeds with full grains and the same size were selected and planted in the mixture of nutrient soil and vermiculite (3:1). The plants were incubated in a chamber with a light intensity of 150 μmol∙m^−2^∙s^−1^ and a photoperiod of 16 h/8 h (light/dark) and a temperature of 25°C. Control soil moisture by daily watering. After 30 d, seedlings with uniform growth were selected and subjected to different treatments: (1) Control treatment (CK): normal growth seedlings; (2) Low-temperature treatment: the normally growing seedlings were placed in a low-temperature incubator at 4°C for 6 h; (3) Salt treatment: seedlings were treated with 150 mL of 200 mM NaCl solution for 6 h; (4) Drought treatment: seedlings were treated with 150 mL of 20% PEG6000 solution for 6 h; (5) MT treatment: the leaves of seedlings were sprayed with 150 μM MT solution until the leaves were infiltrated and treated for 3 h; (6) MeJA treatment: the leaves of seedlings were sprayed with 100 μM MeJA solution until the leaves were infiltrated and treated for 3 h; (7) ABA treatment: the leaves of seedlings were sprayed with 100 μM ABA solution until the leaves were infiltrated and treated for 3 h; (8) H_2_O_2_ treatment: the leaves of seedlings were sprayed with 10 mM H_2_O_2_ solution until the leaves were infiltrated and treated for 3 h. The fourth leaf was taken for the samples from the above treatment. Samples were frozen in liquid nitrogen and stored at −80°C for RNA extraction. Each treatment was replicated three times.

### 2.7. Real-time fluorescence quantitative PCR (qRT-PCR)

Referring to the method of Zhang et al.,^48^ the MiniBEST Plant RNA Extraction Kit (TaKaRa, Japan) was used to extract the total RNA of foxtail millet and Reverse Transcription Kit (TaKaRa, Japan) was used to reverse it for cDNA. Real-time quantitative PCR amplification was performed by SYBR® Premix Ex Taq™ (TaKaRa, Japan). The quantitative reaction system (20 μL) contained 10 μL of SYBR® Premix Ex Taq™, 0.8 μL each of the upstream and downstream primers, 6.4 μL of ddH_2_O, and 2 μL of template cDNA. The amplification program was as follows: pre-denaturation at 95°C for 30 s, denaturation at 95°C for 5 s, annealing at 60°C for 30 s, 40 cycles; the temperature in the dissolution stage was set to 95°C for 15 s, 60°C for 1 min, and 95°C for 15 s. PCR amplification primers were listed in Tab. S2. Three biological replicates were performed for each gene, and the relative expression of genes was calculated by the 2^−ΔΔCt^ method.

## 3. Result

### 3.1. Identification of MAPK cascade genes in foxtail mille

Through Blastp screening and PfamScan database analysis, 93 candidate genes were retrieved, including 15 *SiMAPKs* genes, 10 *SiMAPKKs* genes, and 68 *SiMAPKKK*s genes (Table 1), all the proteins encoded by these genes contain serine/threonine protein kinase-like domains (PF00069). According to the homology between foxtail millet and Poaceae (*Oryza sativa*, *Brachypodium distachyon*), we named the members of the foxtail millet MAPK cascade family, respectively, and the names are shown in Table 1. The basic physicochemical property analysis showed that the CDS length of *SiMAPKs* ranged from 1107 to 1833 bp, the molecular weight of SiMAPKs protein ranged from 42.29 to 69.63 kDa, and the isoelectric point ranged from 5.44 to 9.34. Except for SiMAPK21–2, which is localized in the cytoplasm, all other SiMAPKs are localized in the nucleus. The CDS lengths of *SiMAPKKs* ranged from 975 to 1569 bp, the molecular weights of SiMAPKKs protein ranged from 34.93 to 58.41 kDa, and the isoelectric point ranged from 5.47 to 9.45. SiMAPKKs are mainly localized in the cytoplasm and partially distributed in the plasma membrane (SiMAPKK3–2), mitochondria (SiMAPKK3–1, SiMAPKK10–1, SiMAPKK10–4) and peroxisomes (SiMAPKK10–3). The CDS lengths of *SiMAPKKKs* ranged from 630 to 3357 bp, the molecular weights of SiMAPKKKs protein ranged from 23.59 to 123.65 kDa, and the isoelectric point ranged from 4.3 to 9.71. SiMAPKKKs are mostly localized to the cytoplasm, nucleus, plasma membrane, and mitochondria.

**Table 1.** Basic information of MAPK cascade gene family members in foxtail millet.

Gene name	Gene ID	Protein information			Group	CDS (bp)	Subcellular localization	Chromosome
		PI	MW (kDa)	AA				
**MAPK**								
*SiMAPK3*	SETIT_036218mg	5.46	43.43	375	A	1125	Nucleus	ChroIX: 49447133–49452474 (+)
*SiMAPK4*	SETIT_036240mg	5.96	42.29	372	B	1116	Nucleus	ChroIX: 40427082–40430995 (-)
*SiMAPK6*	SETIT_006611mg	5.44	44.47	393	A	1179	Nucleus	ChroIV: 5381844–5387729 (+)
*SiMAPK7*	SETIT_006708mg	6.63	42.34	369	C	1107	Nucleus	ChroIV: 36733811–36738034 (-)
*SiMAPK11*	SETIT_013899mg	6.44	44.10	390	B	1170	Nucleus	ChroVI: 4549915–4553473 (-)
*SiMAPK14*	SETIT_017554mg	6.53	42.41	370	C	1110	Nucleus	ChroI: 7886461–7890224 (+)
*SiMAPK16–1*	SETIT_026197mg	8.79	61.17	535	D	1605	Nucleus	ChroVIII: 14012876–14019153 (-)
*SiMAPK16–2*	SETIT_021645mg	8.83	63.46	557	D	1671	Nucleus	ChroIII: 3700371–3706333 (+)
*SiMAPK17–1*	SETIT_006144mg	6.64	65.25	574	D	1722	Nucleus	ChroIV: 39101238–39106354 (+)
*SiMAPK17–2*	SETIT_016957mg	7.67	57.85	506	D	1518	Nucleus	ChroI: 8369629–8375826 (+)
*SiMAPK20–1*	SETIT_000725mg	9.14	69.63	611	D	1833	Nucleus	ChroV: 29846765–29852096 (-)
*SiMAPK20–2*	SETIT_000788mg	9.34	67.37	589	D	1767	Nucleus	ChroV: 32398562–32404773 (+)
*SiMAPK20–3*	SETIT_021560mg	9.2	67.26	591	D	1773	Nucleus	ChroIII: 10338871–10343433 (-)
*SiMAPK21–1*	SETIT_021565mg	6.74	66.86	590	D	1770	Nucleus	ChroIII: 9482394–9487115 (-)
*SiMAPK21–2*	SETIT_004793mg	8.3	54.72	480	D	1440	Cytoplasmic	ChroV: 30468187–30473047 (-)
**MAPKK**								
*SiMAPKK1*	SETIT_006786mg	5.72	58.41	523	B	1569	Cytoplasmic	Chro IV: 2443324–2446915 (-)
*SiMAPKK3–1*	SETIT_015676mg	8.68	37.24	341	C	1023	Mitochondrial	Chro VI: 28354293–28359769 (-)
*SiMAPKK3–2*	SETIT_016904mg	9.45	39.55	366	C	1098	Plasma membrane	Chro I: 37055070–37064379 (-)
*SiMAPKK4–1*	SETIT_002102mg	9.21	37.39	344	C	1032	Cytoplasmic	Chro V: 29299482–29301068 (+)
*SiMAPKK4–2*	SETIT_017572mg	5.57	39.95	355	A	1065	Cytoplasmic	Chro I: 40030490–40031926 (-)
*SiMAPKK5*	SETIT_006813mg	5.47	39.88	355	A	1065	Cytoplasmic	Chro IV: 3194809–3196335 (-)
*SiMAPKK6–1*	SETIT_022487mg	8.65	52.62	465	B	1395	Cytoplasmic	Chro III: 3943434–3947087 (-)
*SiMAPKK6–2*	SETIT_002024mg	6.36	39,06	350	A	1050	Cytoplasmic	Chro V: 35559395–35563839 (-)
*SiMAPKK10–1*	SETIT_036560mg	6.08	35.18	331	D	993	Cytoplasmic	Chro IX: 52473551–52475044 (+)
*SiMAPKK10–2*	SETIT_031963mg	7.2	34.93	325	D	975	Mitochondrial	Chro II: 27338771–27339748 (+)
**MAPKKK**								
*SiRAF1*	SETIT_016154mg	5.4	123.65	1117	Raf	3351	Cytoplasmic	Chro I: 39992146–39998092 (+)
*SiRAF2*	SETIT_005722mg	5.74	112.02	1055	Raf	3165	Cytoplasmic	Chro IV: 3869240–3873680 (-)
*SiRAF3*	SETIT_005743mg	5.43	117.35	1076	Raf	3228	Cytoplasmic	Chro IV: 8837774–8847258 (-)
*SiRAF4*	SETIT_016157mg	5.33	122.71	1109	Raf	3327	Cytoplasmic	Chro I: 37818484–37828116 (-)
*SiRAF5*	SETIT_034063mg	5.96	110.60	1005	Raf	3015	Plasma membrane	Chro IX: 55753452–55760899 (-)
*SiRAF6*	SETIT_005733mg	5.6	117.96	1101	Raf	3303	Plasma membrane	Chro IV: 31811633–31817207 (-)
*SiRAF7*	SETIT_016359mg	6.77	87.63	786	Raf	2358	Plasma membrane	Chro I: 1846576–1857977 (-)
*SiRAF8*	SETIT_034087mg	6.04	107.67	975	Raf	2925	Cytoplasmic	Chro IX: 19178122–19185997 (+)
*SiRAF9*	SETIT_021297mg	7.65	84.00	756	Raf	2268	Nuclear	Chro III: 42822482–42829672 (-)
*SiRAF10*	SETIT_029013mg	6.57	84.12	770	Raf	2310	Mitochondrial	Chro III: 15337559–15344974 (-)
*SiRAF11*	SETIT_016333mg	5.87	87.77	807	Raf	2421	Cytoplasmic	Chro I: 25695280–25701652 (-)
*SiRAF12*	SETIT_009672mg	8.22	65.05	590	Raf	1770	Cytoplasmic	Chro VII: 29640523–29645603 (+)
*SiRAF13*	SETIT_034839mg	5.41	65.16	587	Raf	1761	Cytoplasmic	Chro IX: 49068027–49071411 (+)
*SiRAF14*	SETIT_016301mg	5.37	83.00	766	Raf	2298	Mitochondrial	Chro I: 3298526–3306225 (-)
*SiRAF15*	SETIT_028893mg	5.46	96.88	872	Raf	2616	Nuclear	Chro II: 45663808–45667702 (-)
*SiRAF16*	SETIT_0306602mg	9.51	23.59	210	Raf	630	Mitochondrial	Chro II: 12053079–12054247 (+)
*SiRAF17*	SETIT_016766mg	5.71	52.01	469	Raf	1407	Cytoplasmic	Chro I: 9665305–9669596 (+)
*SiRAF18*	SETIT_017053mg	8.88	53.95	485	Raf	1455	Mitochondrial	Chro I: 30424559–30429727 (-)
*SiRAF19*	SETIT_003916mg	9.64	44.87	399	Raf	1197	Mitochondrial	Chro V: 43522754–43525439 (+)
*SiRAF20*	SETIT_015231mg	8.35	33.47	309	Raf	927	Plasma membrane	Chro VI1624566–1625492 (-)
*SiRAF21*	SETIT_017293mg	6.64	47.02	425	Raf	1275	Plasma membrane	Chro I: 6393777–6396752 (+)
*SiRAF22*	SETIT_010212mg	6.89	46.13	417	Raf	1251	Plasma membrane	Chro VII: 29482000–29486075 (-)
*SiRAF23*	SETIT_029452mg	6.34	59.58	534	Raf	1602	Cytoplasmic	Chro II: 40264168–40273428 (-)
*SiRAF24*	SETIT_001285mg	6.54	53.40	474	Raf	1422	Mitochondrial	Chro V: 36816851–36820917 (-)
*SiRAF25*	SETIT_036191mg	8.37	41.67	378	Raf	1134	Cytoplasmic	Chro IX: 10695090–10699462 (-)
*SiRAF26*	SETIT_035834mg	8.65	46.61	425	Raf	1275	Plasma membrane	Chro IX: 2116617–2120734 (+)
*SiRAF27*	SETIT_017658mg	7.7	39.54	350	Raf	1050	Plasma membrane	Chro I: 29601963–29607296 (+)
*SiRAF28*	SETIT_001833mg	8.18	42.18	382	Raf	1146	Cytoplasmic	Chro V: 30366972–30370933 (+)
*SiRAF29*	SETIT_001885mg	9.03	41.82	374	Raf	1122	Plasma membrane	Chro V: 6168525–6172461 (-)
*SiRAF30*	SETIT_008355mg	9.23	26.92	236	Raf	708	Plasma membrane	Chro IV: 32423565–32424386 (-)
*SiRAF31*	SETIT_029825mg	6.88	49.40	449	Raf	1347	Plasma membrane	Chro II: 46421957–46426344 (-)
*SiRAF32*	SETIT_013509mg	5.61	52.40	463	Raf	1389	Plasma membrane	Chro VI: 231890–237558 (-)
*SiRAF33*	SETIT_035970mg	8.38	44.33	403	Raf	1209	Plasma membrane	Chro IX: 5722282–5725159 (-)
*SiRAF34*	SETIT_027523mg	6.38	79.76	707	Raf	2121	Nuclear	Chro VIII: 38492027–38499141 (+)
*SiRAF35*	SETIT_021033mg	5.55	122.59	1119	Raf	3357	Nuclear	Chro III: 47748936–47754498 (+)
*SiRAF36*	SETIT_009795mg	5.15	58.32	533	Raf	1599	Cytoplasmic	Chro VII: 33646173–33653823 (-)
*SiRAF37*	SETIT_026192mg	5.04	58.81	544	Raf	1632	Cytoplasmic	Chro VIII: 3342619–3349422 (+)
*SiRAF38*	SETIT_024868mg	5.41	63.06	565	Raf	1695	Cytoplasmic	Chro III: 329098–334636 (+)
*SiZIK1*	SETIT_021438mg	4.95	72.69	647	ZIK	1941	Nuclear	Chro III: 4308763–4313699 (-)
*SiZIK2*	SETIT_016704mg	5.15	66.40	597	ZIK	1791	Cytoplasmic	Chro I: 34272225–34275215 (+)
*SiZIK3*	SETIT_029371mg	4.95	62.51	566	ZIK	1698	Cytoplasmic	Chro II: 5036074–5039034 (-)
*SiZIK4*	SETIT_026086mg	5.22	52.89	474	ZIK	1422	Cytoplasmic	Chro VIII: 817643–820823 (+)
*SiZIK5*	SETIT_009574mg	5.99	70.13	627	ZIK	1881	Cytoplasmic	Chro VII: 34110652–34115801 (-)
*SiZIK6*	SETIT_027310mg	5.51	50.05	448	ZIK	1344	Cytoplasmic	Chro VIII: 3102569–3105020 (-)
*SiZIK7*	SETIT_032842mg	8.2	42.02	378	ZIK	1134	Cytoplasmic	Chro II: 44387740–44389619 (+)
*SiZIK8*	SETIT_029157mg	5.69	74.93	659	ZIK	1977	Nuclear	Chro II: 43907396–43910350 (+)
*SiZIK9*	SETIT_011766mg	6.74	41.44	364	ZIK	1092	Cytoplasmic	Chro VII: 33692048–33693934 (+)
*SiMAPKKK1*	SETIT_009321mg	9.7	97.34	893	MEKK	2679	Cytoplasmic	Chro VII: 26989609–26999711 (-)
*SiMAPKKK2*	SETIT_016275mg	9.68	96.41	888	MEKK	2664	Nuclear	Chro I: 33826548–33833410 (-)
*SiMAPKKK3*	SETIT_034335mg	9.63	83.26	773	MEKK	2319	Nuclear	Chro IX: 4745273–4752261 (+)
*SiMAPKKK4*	SETIT_014949mg	6.04	67.14	615	MEKK	1845	Nuclear	Chro VI: 26801354–26806981 (-)
*SiMAPKKK5*	SETIT_029131mg	5.97	73.97	680	MEKK	2040	Cytoplasmic	Chro II: 29118577–29123708 (-)
*SiMAPKKK6*	SETIT_029029mg	9.55	81.93	752	MEKK	2256	Plasma membrane	Chro II: 903382–909243 (-)
*SiMAPKKK7*	SETIT_026073mg	9.37	71.68	671	MEKK	2013	Cytoplasmic	Chro VIII: 13440726–13445207 (-)
*SiMAPKKK8*	SETIT_039120mg	9.19	94.32	876	MEKK	2628	Cytoplasmic	Chro IX: 7803458–7809446 (+)
*SiMAPKKK9*	SETIT_034802mg	6.03	64.65	594	MEKK	1782	Nuclear	Chro IX: 50233622–50238161 (-)
*SiMAPKKK10*	SETIT_016544mg	9.71	73.68	680	MEKK	2040	Mitochondrial	Chro I: 27522373–27527779 (-)
*SiMAPKKK11*	SETIT_009480mg	8.92	78.32	728	MEKK	2184	Nuclear	Chro VII: 20605071–20610788 (-)
*SiMAPKKK12*	SETIT_004003mg	4.73	45.56	440	MEKK	1320	Golgi	Chro V: 34353442–34354761 (+)
*SiMAPKKK13*	SETIT_020144mg	5.4	35.89	340	MEKK	1020	Mitochondrial	Chro I: 16103557–16104576 (+)
*SiMAPKKK14*	SETIT_005153mg	4.96	43.14	407	MEKK	1221	Mitochondrial	Chro V: 34407339–34408654 (-)
*SiMAPKKK15*	SETIT_001317mg	4.57	49.68	469	MEKK	1407	Plasma membrane	Chro V: 34364069–34365574 (+)
*SiMAPKKK16*	SETIT_003823mg	5.12	36.04	339	MEKK	1017	Golgi	Chro V: 34385910–34386958 (-)
*SiMAPKKK17*	SETIT_004745mg	4.89	47.14	445	MEKK	1335	Mitochondrial	Chro V: 34376644–34378884 (-)
*SiMAPKKK18*	SETIT_021980mg	4.95	48.00	463	MEKK	1389	Mitochondrial	Chro III: 11699845–11701763 (+)
*SiMAPKKK19*	SETIT_025330mg	4.3	44.30	418	MEKK	1254	Cytoplasmic	Chro III: 11706406–11707662 (+)
*SiMAPKKK20*	SETIT_039013mg	5.99	44.54	436	MEKK	1308	Extracellular	Chro IX: 49146680–49147987 (-)
*SiMAPKKK21*	SETIT_017260mg	5.82	47.25	431	MEKK	1293	Nuclear	Chro I: 39218007–39222646 (+)

1. The ID, CDS, and chromosome information of the 93 foxtail millet MAPK genes in the table were obtained from the foxtail millet （*Setaria italica*） genome database (http://plants.ensembl.org/index.html); Protein information was obtained from ExPASy (http://au.expasy.org/tools); Subcellular localization was obtained from ProtComp 9.0 (http://linux1.softberry.com/berry.phtml); The naming and grouping information was determined based on subsequent experiments in this study.

2. Chromosome location indicates the location information of genes on chromosomes (including the chromosomal location, start location, and end location). “+” indicates the sense strand and “-” indicates the antisense strand.

### 3.2. Phylogenetic relationships of proteins encoded by SiMAPK cascade genes

**Figure 1.**
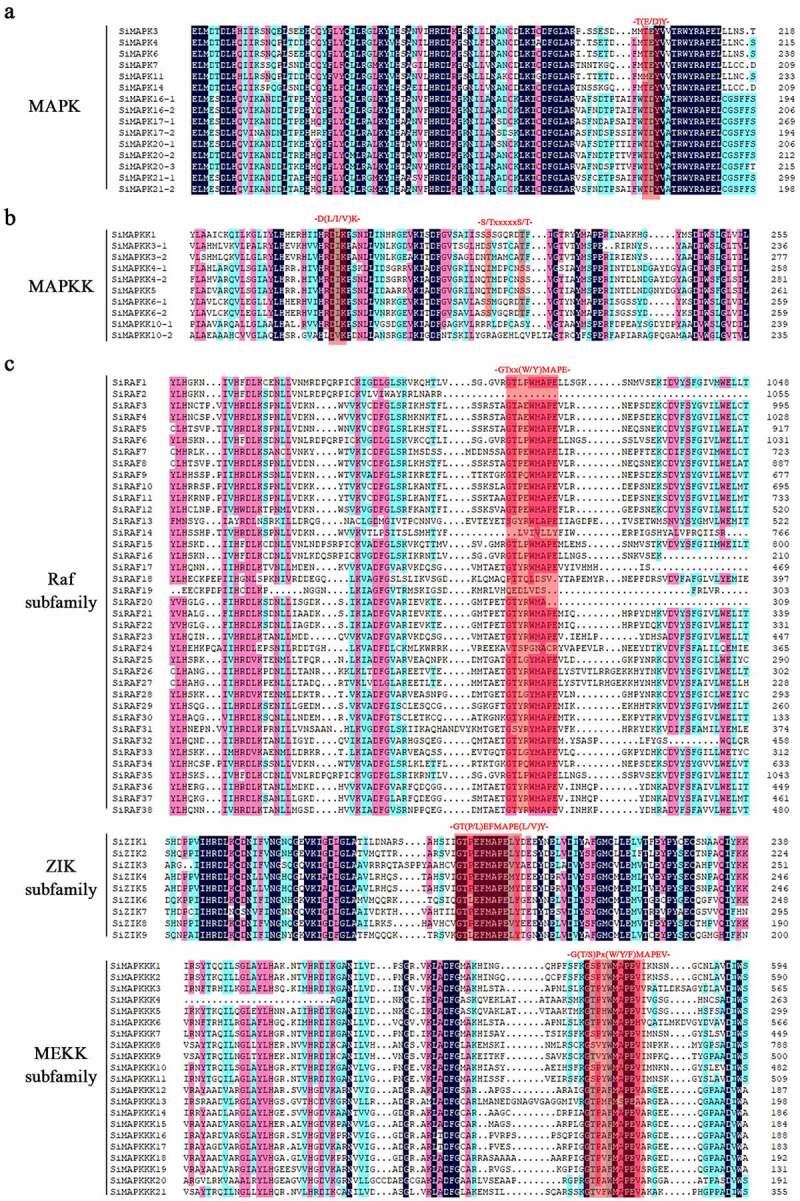
Multiple sequence alignment of proteins encoded by SiMAPK cascade genes.

The amino acid sequence of MAPKs in foxtail millet were compared by DNAMAN 8 (Figure 1). MAPKs are activated when both tyrosine and threonine residues in the -T(E/D)Y- motif are phosphorylated by the bispecific kinases MAPKKs.^1^ It was found that all 15 SiMAPKs had -T(E/D)Y- conserved motif, among which 5 SiMAPKs (SiMAPK3, SiMAPK4, SiMAPK6, SiMAPK7, and SiMAPK14) contained the -TEY conserved motif, 1 SiMAPK (SiMAPK11) had the -MEY- motif instead of the -TEY-motif, and 9 SiMAPKs had the -TDY-conserved motif (Figure 1a). MAPKKs were only accepted if they displayed the consensus sequences for dual-specificity protein kinases, including the conserved aspartate and lysine residues within the active site motif, -D(L/I/V) K-, and the plant-specific phosphorylation target site motif, -S/T-XXXXX-S/T-, within the activation loop.^11^ All 10 SiMAPKKs had the -D(L/I/V)K conserved motif, of which all sequences except SiMAPKK10–1 and SiMAPKK10–2 had the -S/T-XXXXX-S/T- phosphorylation motif (Figure 1b). The 68 SiMAPKKKs were divided into three subtypes according to conserved motifs: Raf, ZIK, and MEKK (Figure 1c), MEKK-like proteins contain less conserved protein structures, while ZIK-like and Raf-like proteins have a KD (kinase domain) structural domain at the C-terminus and an RD (regulatory domain) structural domain at the N-terminus.^49, 50^ It was found that 38 SiMAPKKKs containing the conserved motif -GTxx(W/Y)MAPE- belonged to the Raf isotype, 9 SiMAPKKKs containing the conserved motif -GTPEFMAPE(L/V/M)(Y/F/L)- belonged to ZIK isotype, 21 SiMAPKKKs containing the conserved motif -G(T/S)Px(W/F/Y)MAPEV- belonged to MEKK isotype. In addition, the phylogenetic tree of 93 foxtail millet MAPK cascade proteins was constructed by MEGA7, and it was found that the phylogenetic tree was basically consistent with the sequence alignment results, and it was mainly divided into six clades, of which the SiMAPKKKs had four branches and the Raf isoforms had two branches (Fig. S1).

**Figure 2.**
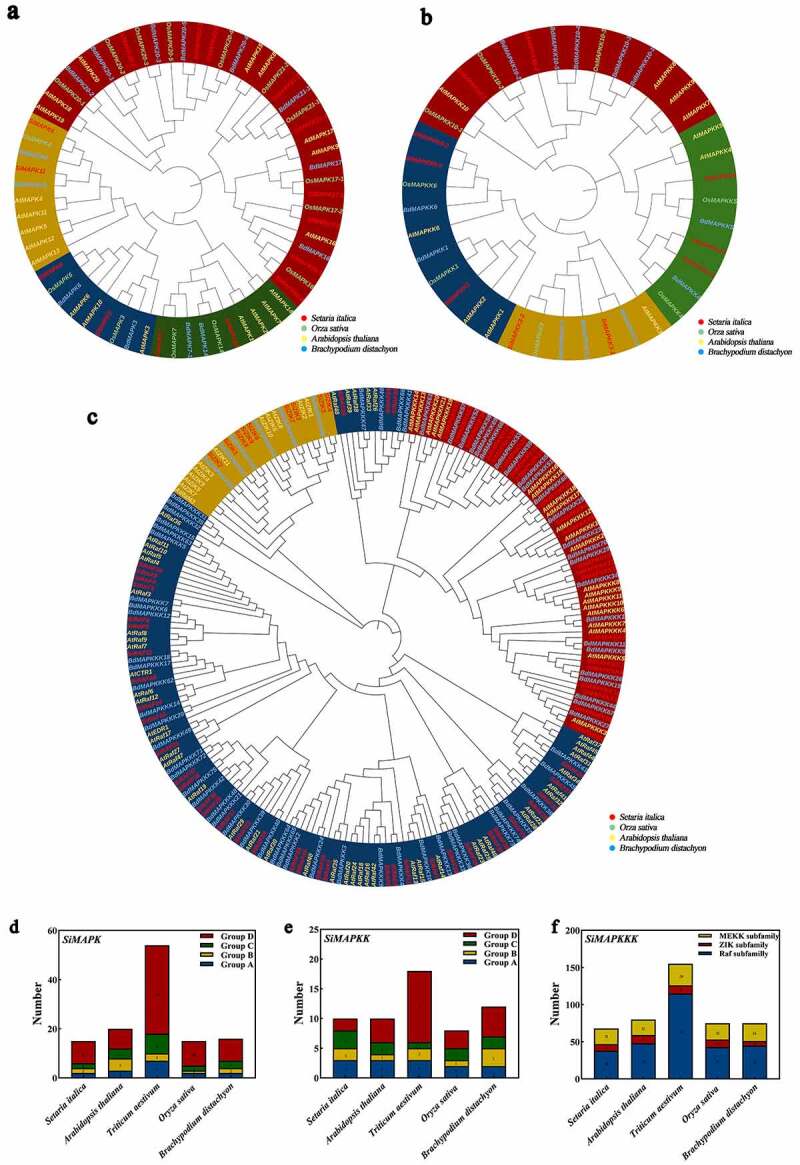
Phylogenetic relationship and taxonomic statistics of MAPK cascade genes in foxtail millet and other species. (a, d. Phylogenetic tree and taxonomic statistics of MAPK genes; b, e. Phylogenetic tree and taxonomic statistics of MAPKK genes; c, f. Phylogenetic tree and taxonomic statistics of MAPKKK genes; In a, b, d, e, blue represents group A, yellow represents group B, green represents group C, and red represents group D; In a and b, four species of *Setaria italica*, *Arabidopsis thaliana*, *Oryza sativa*, and *Brachypodium distachyon* were used to construct phylogenetic trees; In c and f, three species of *Setaria italica*, *Arabidopsis thaliana*, and *Brachypodium distachyon* were used to construct phylogenetic trees, where yellow represents the MEKK subgroup, red represents the ZIK subgroup, and blue represents the Raf subgroup.)

The phylogenetic tree of MAPKs, MAPKKs, and MAPKKKs in *Setaria italica*, *Arabidopsis thaliana*, *Oryza sativa*, and *Brachypodium distachyon* were constructed in MEGA 7 by neighbor-joining method (NJ) (Figure 2). It was found that the MAPK cascade proteins in *Setaria italica* were more closely related to that in *Brachypodium distachyon* in terms of homologous evolution. The SiMAPK cascade proteins were classified according to phylogenetic tree branching, and it was found that SiMAPKs could be classified into four groups: A (SiMAPK3, 6), B (SiMAPK4, 11), C (SiMAPK7, 14), and D (SiMAPK16–1, 16–2, 17–1, 17–2, 20–1, 20–2, 20–3, 21–1, 21–2) (Figure 2a); SiMAPKKs were classified into four groups: A (SiMAPKK1, 6–1, 6–2), B (SiMAPKK3–1, 3–2), C (SiMAPKK4–1, 4–2, 5), and D (SiMAPKK10–1, 10–2) (Figure 2b); SiMAPKKKs were classified into three subtypes: Raf, ZIK, and MEKK (Figure 2c). In addition, according to the gene number statistics (Figure 2d–f), it was found that the number and classification of MAPK cascade genes in foxtail millet were basically similar to those of other species except wheat (tetraploid).

### 3.3. Conserved motifs and gene structure analysis

**Figure 3.**
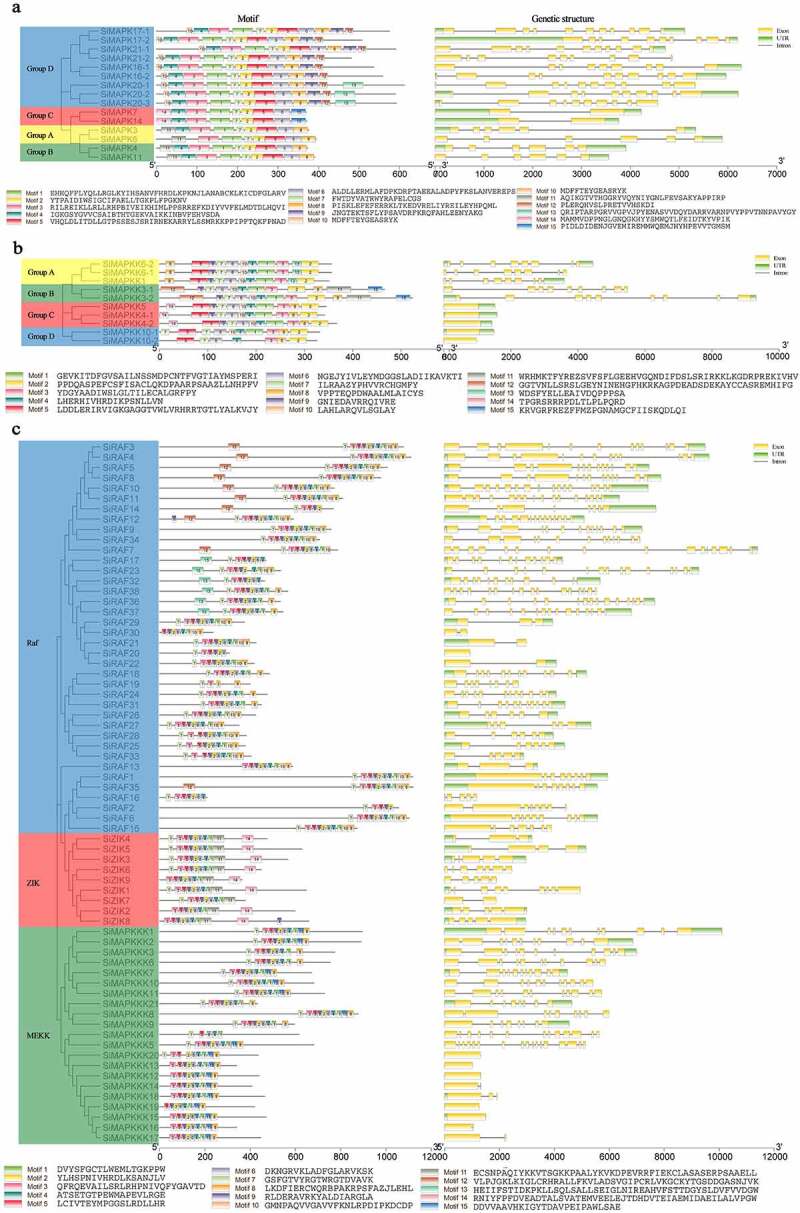
Conserved motifs and gene structure analysis of the SiMAPK cascade gene family (a. Conserved motifs and gene structure analysis of the *SiMAPKs*; b. Conserved motifs and gene structure analysis of the *SiMAPKKs*; c. Conserved motifs and gene structure analysis of the *SiMAPKKKs*).

In the conserved motif analysis, the conserved motif of the search program was set to 15 motifs (Fig. S2), and the size of each motif was between 10 and 50 amino acids. The analysis results were shown in Figure 3. In the motif analysis results of SiMAPKs (Figure 3a), all SiMAPKs contained motifs 1, 2, 3, 5, 6, and 7, among which motif 7 contained MAPK phosphorylation site -T(E/D)Y-motif. In SiMAPKKs, all SiMAPKKs shared six motifs (motifs 1, 2, 3, 5, 6, 8) (Figure 3b). In SiMAPKKK, the motif distribution was different in different MAPKKK isoforms, but most SiMAPKKK proteins have similar motif composition (Figure 3c). The exon-intron structure of the SiMAPK cascade genes was shown in Figure 3, the number of exons in the *SiMAPKs* gene was between 2 and 11. There were six exons in the SiMAPKs of groups A and B, respectively, and the length and arrangement of exons were relatively regular. There were two longer exons in the SiMAPKs of group C, and the non-coding regions were longer. The number of exons of SiMAPKs was highest in group D, ranging from 9 to 11 (Figure 3a). Similar results could be observed in *Cicer arietinum*, *Solanum lycopersicum*, and *Brachypodium distachyon*. Figure 3b showed that the number of exons in the *SiMAPKKs* gene was between 1 and 9, and the number of exons in groups A and B was between 8 and 9. However, there was only one exon in groups C and D, and no intron structure, and all members except SiMAPKK10–2 had non-coding regions. The gene structure of SiMAPKKK showed that most SiMAPKKKs had multiple exons and introns, and the coding regions of six SiMEKK genes did not contain introns (Figure 3c). From the above analysis, it was found that there are similar motifs and exon/intron distribution patterns among genes that are evolutionarily closely related (Figure 3).

### 3.4. Chromosomal location, gene duplication, and collinearity analysis

**Figure 4.**
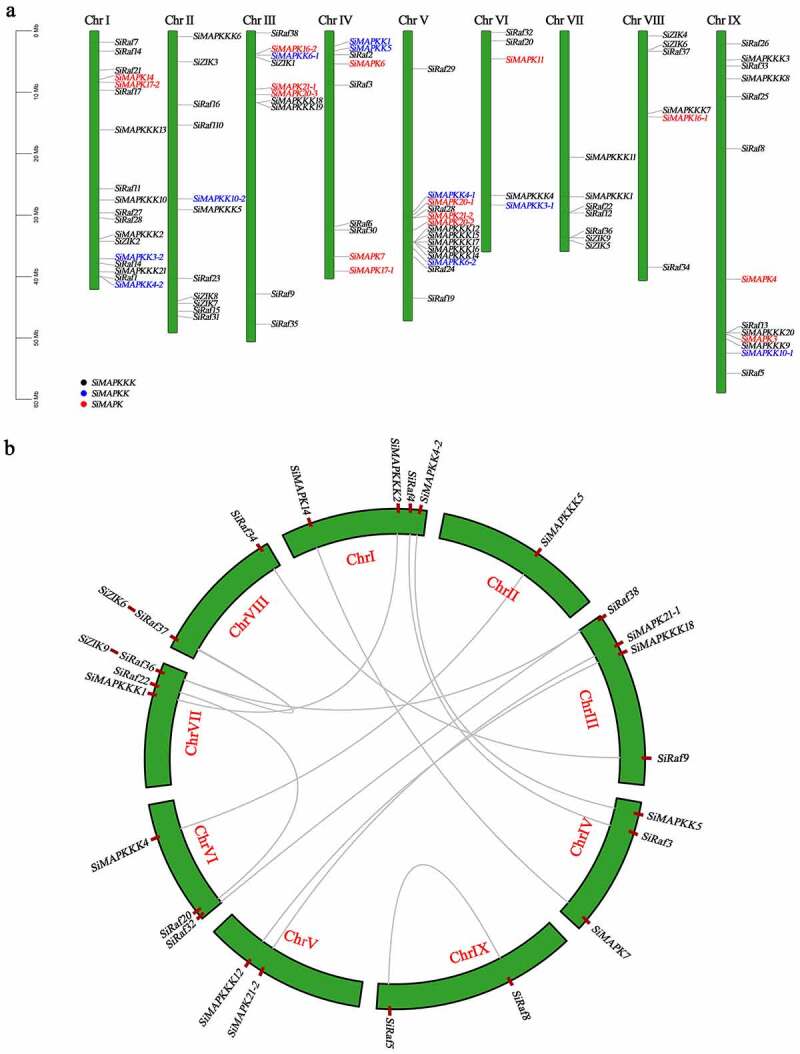
Chromosomal distribution and intraspecific collinearity analysis of SiMAPK cascade genes (a. The location of foxtail millet SiMAPK cascade genes on the chromosome; b. Analysis of gene duplication events in the SiMAPK cascade gene family).

The location of foxtail millet SiMAPK cascade genes on the chromosome is shown in Figure 4a, 93 SiMAPK cascade genes were located on the 9 chromosomes of foxtail millet, and the distribution was uneven. The results of gene duplication event analysis clarified the amplification mechanism of the SiMAPK cascade gene family, and a total of 14 gene pairs were identified in the SiMAPK cascade gene family (Figure 4b, Tab. S3). Among them, there were 2 and 1 segmental duplication events in *SiMAPKs* and *SiMAPKKs*, respectively, and 10 segmental duplication events and 1 tandem duplication event in *SiMAPKKKs*. In order to detect the selection effect in the process of gene divergence after replication, the Ka/Ks ratio of the replication pair was further calculated, and the results showed that the Ka/Ks ratio of the MAPK cascade genes ranged from 0.0385 to 0.4287 (Ka/Ks < 1), the average value was 0.2117, indicating that they experienced purifying selection pressure during foxtail millet evolution (Tab. S3).

**Figure 5.**
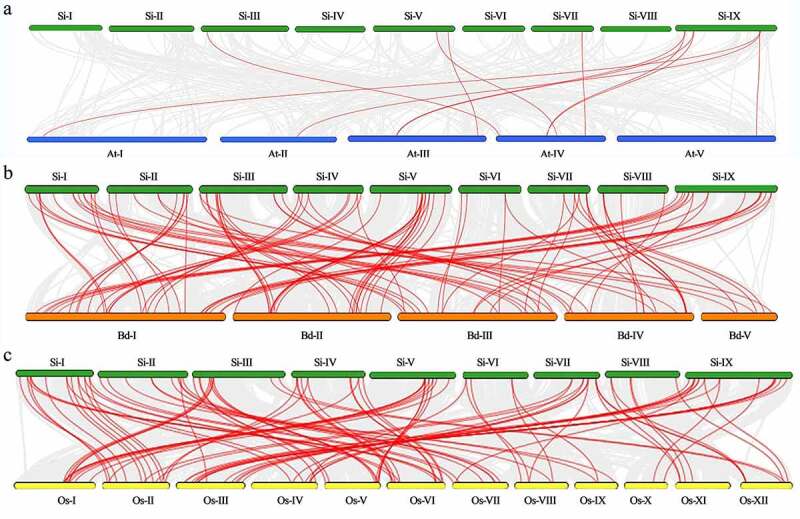
Interspecies collinearity analysis of MAPK cascade genes in foxtail millet and three different species (a, b, c represents the collinearity of MAPK cascade genes between *Setaria italica* and *Arabidopsis thaliana*, *Brachypodium distachyon* and *Oryza sativa*, respectively, where the collinear blocks generated by foxtail millet and other plant genomes are indicated by gray lines in the background, while the collinear MAPK cascade genes are paired with red lines.).

**Figure 6.**
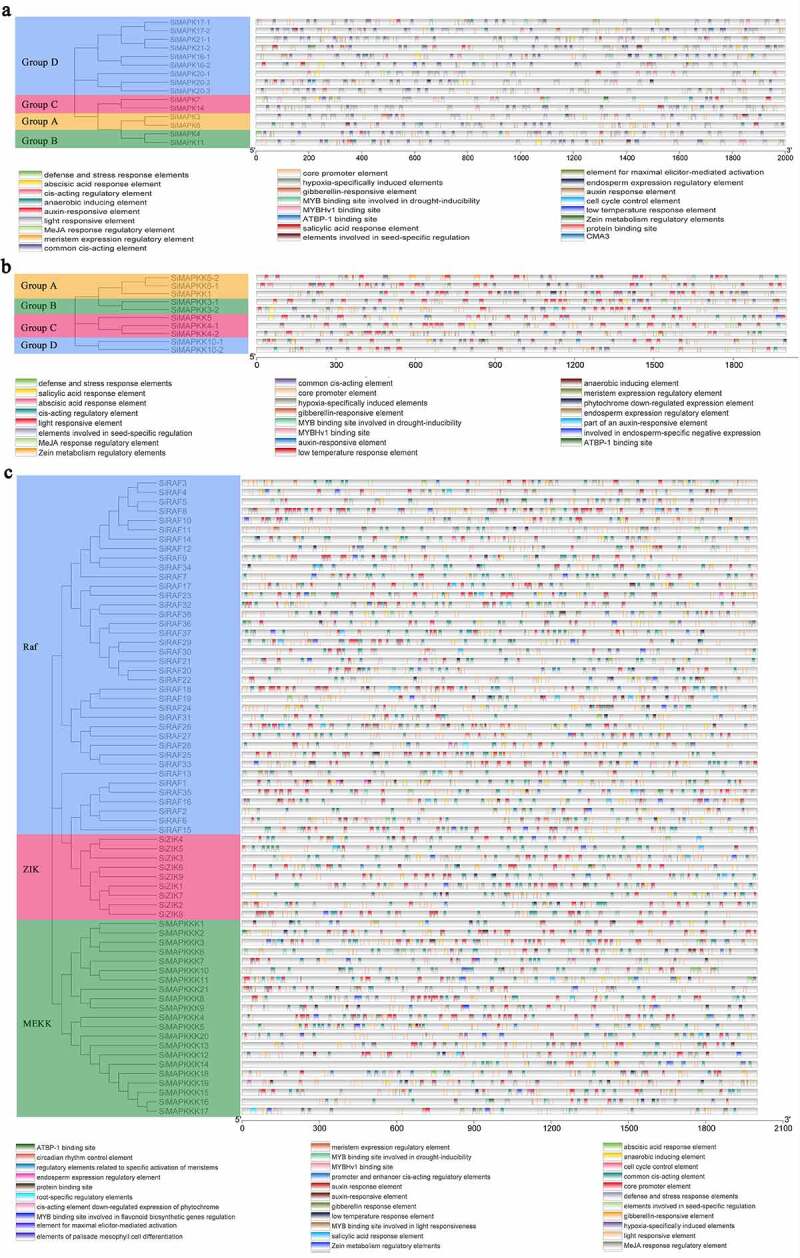
Analysis of cis-acting elements of SiMAPK cascade genes (a. Analysis of cis-acting elements of the SiMAPKs; b. Analysis of cis-acting elements of the SiMAPKKs; c. Analysis of cis-acting elements of the SiMAPKKKs).

In addition, as shown in Figure 5, according to the comparative analysis of collinearity between *Setaria italica* and other species (*Arabidopsis thaliana*, *Oryza sativa*, and *Brachypodium distachyon*), there were 11, 89, and 94 orthologous counterparts of the foxtail millet MAPK cascade genes in *Arabidopsis thaliana*, *Oryza sativa*, and *Brachypodium distachyon*, respectively (Tab. S4, S5, S6). Among them, the average Ka/Ks values between *Setaria italica* and *Arabidopsis thaliana*, *Oryza sativa*, and *Brachypodium distachyon* were 0.1995, 0.1725, and 0.0075, respectively, indicating that these gene pairs between *Setaria italica*, *Oryza sativa*, and *Brachypodium distachyon* should have experienced extensive purifying selection. Furthermore, most of the SiMAPK cascade genes exhibited synteny bias toward specific chromosomes in *Oryza sativa* and *Brachypodium distachyon*, suggesting that chromosomal rearrangement events such as duplications and inversions affect the distribution of MAPK genes in the genome.

### 3.5. Predictive analysis of cis-acting elements

The 2 kb sequence at the upstream of the transcription initiation site of the SiMAPK cascade genes was extracted, the distribution of cis-elements of each SiMAPK cascade gene was predicted through the PLACE database, and as a result, many cis-acting elements that responded to plant hormones and environmental stress were identified (Figure 6, Tab. S7). The promoter regions of most genes contained plant hormone response elements, such as 89 genes containing ABA response elements in their promoter regions, 50 genes containing auxin response elements in their promoter regions, and 86 genes containing MeJA response elements. In addition, the promoter regions of some SiMAPK cascade genes contained cis-elements that responded to environmental stress. For example, the promoter regions of 25 genes including *SiMAPK4* contained stress and defense response elements, and the promoter regions of 44 genes had low-temperature response elements and the promoter regions of 49 genes contained MYB transcription factor-binding sites that may be involved in drought stress. The above results suggested that most of the genes in the SiMAPK cascade play crucial roles in hormone signaling pathways and stress responses.

### 3.6. Expression patterns of each *SiMAPK* cascade gene between different tissues

**Figure 7.**
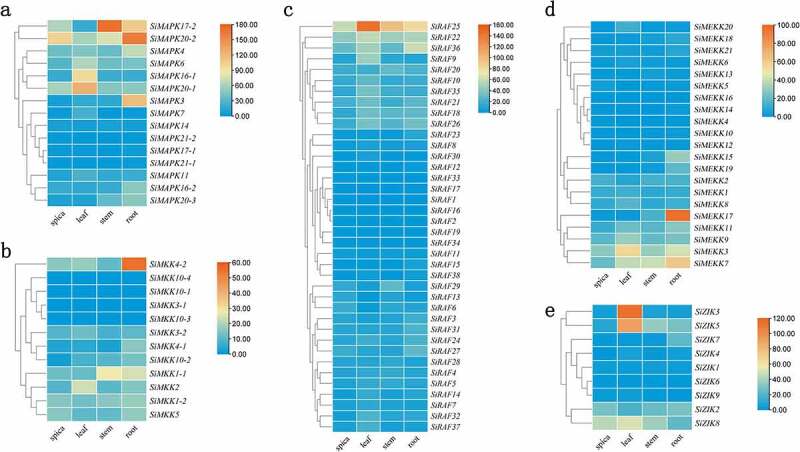
Expression levels of SiMAPK cascade genes in different tissues (a. Expression levels of *SiMAPKs* in different tissues; b. Expression levels of *SiMAPKKs* in different tissues; c. Expression levels of *SiRafs* in different tissues d. Expression levels of *SiMEKKs* in different tissues; e. Expression levels of *SiZIKs* in different tissues).

RNA-Seq data downloaded from the *Setaria italica* Gene Bank showed that SiMAPK cascade genes were expressed in multiple organs (root, stem, leaf, and spica) of foxtail millet (Figure 7, plotted with FPKM values). The expression levels of these MPAK cascade genes varied greatly. Among SiMAPKs, *SiMAPK17–2*, *SiMAPK20–2*, *SiMAPK16–1*, *SiMAPK20–1*, *SiMAPK3*, and other genes showed higher expression levels in various organs. Among SiMAPKKs, *SiMAPKK4–2*, *SiMAPKK6–2*, *SiMAPKK1* were highly expressed in roots, stems, and leaves. Among SiMAPKKKs, *SiRaf25*, *SiMAPKKK3*, *SiZIK3*, *SiZIK5* were highly expressed in leaves, *SiRaf25*, *SiRaf36*, *SiMAPKKK17*, *SiMAPKKK3*, *SiMAPKKK7* were highly expressed in roots, and *SiRaf25, ZIK5*, *ZIK8*, and *SiMAPKKK7* were highly expressed in stems.

### 3.7. Expression levels of SiMAPKs and SiMAPKKs genes under exogenous hormone and H_2_O_2_ treatment

**Figure 8.**
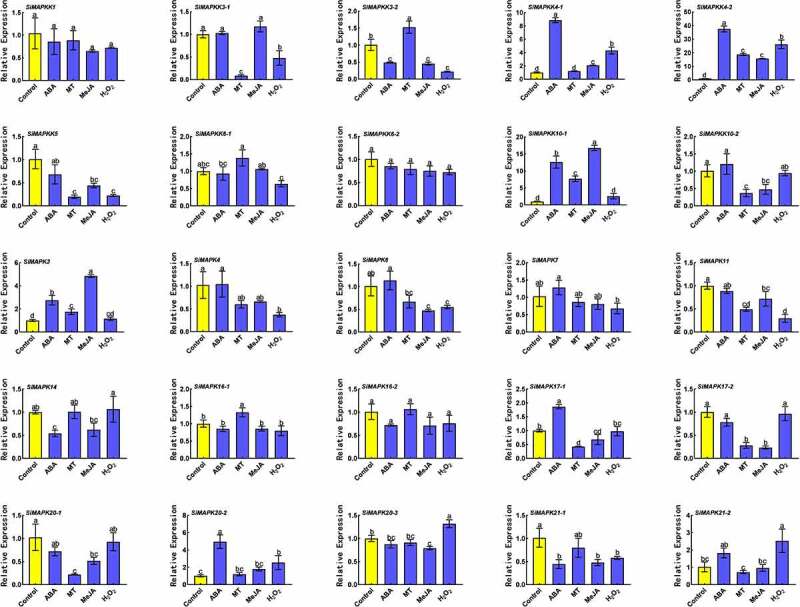
Expression levels of SiMAPKs and SiMAPKKs genes under ABA, MT, MeJA, and H_2_O_2_ treatments.

Present studies have confirmed that the MAPK cascade pathway interacts with phytohormone signaling and the secondary messenger H_2_O_2_-mediated signaling pathways to form a complex signal regulatory network. Therefore, in this study, the expression levels of SiMAPKs and SiMAPKKs genes under the treatment of exogenous H_2_O_2_ and exogenous phytohormones (ABA, MT, and MeJA) were determined, and it was found that most genes were induced after treatment with exogenous phytohormones and H_2_O_2_ (Figure 8). For example, *SiMAPKK4–1*, *SiMAPKK4–2*, *SiMAPKK10–1*, *SiMAPK3*, and *SiMAPK17–1* were induced up-regulated under ABA treatment. *SiMAPKK3–2, SiMAPKK4–2, SiMAPKK6–1, SiMAPKK10–1, SiMAPK3*, and *SiMAPK16–1* were significantly up-regulated under MT treatment. *SiMAPKK3–1, SiMAPKK4–2, SiMAPKK10–1*, and *SiMAPK3* were induced expression by exogenous MeJA. *SiMAPKK4–1, SiMAPKK4–2, SiMAPK20–2, SiMAPK20–3*, and *SiMAPK21–2* were significantly up-regulated under exogenous H_2_O_2_ treatment. However, most genes were inhibited by exogenous phytohormones and H_2_O_2_, such as *SiMAPKK5*, *SiMAPK11*, *SiMAPK20–1*, etc. In addition, some genes were also found to be induced by various hormones, such as *SiMAPKK4–1, SiMAPKK4–2, SiMAPKK10–1*, and so on. The above results indicated that exogenous H_2_O_2_ and phytohormones had certain regulatory effects on the expression of SiMAPKs and SiMAPKKs gene in foxtail millet, and changes in external substances could affect the MAPK cascade signal transduction in foxtail millet.

### 3.8. Expression levels of *SiMapks* and *SiMapkks* genes under abiotic stress

**Figure 9.**
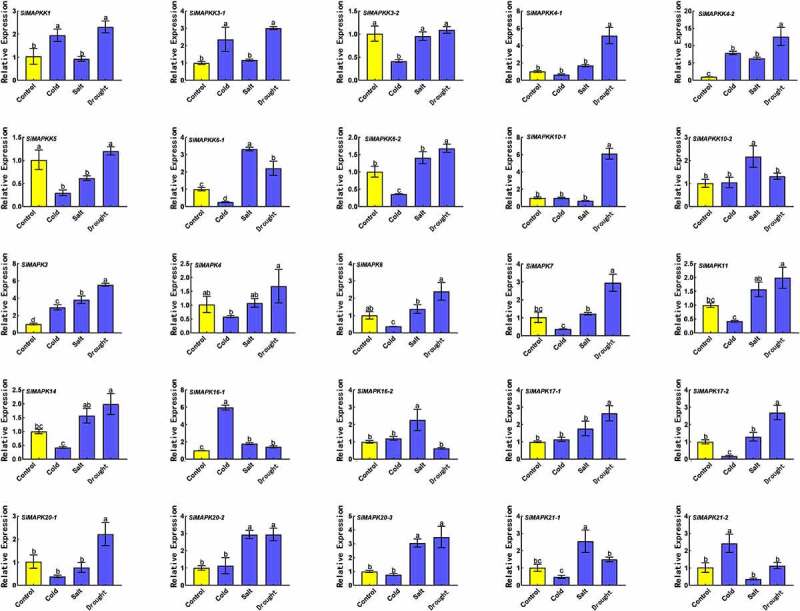
Expression levels of SiMAPKs and SiMAPKKs gene under low temperature, salt, and drought stress.

As a key molecule linking extracellular and intracellular signal transduction, MAPK cascades had been widely reported to be involved in plant abiotic stress responses. Figure 9 showed the expression of SiMAPKs and SiMAPKKs genes in foxtail millet under cold, salt, and simulated drought treatments. The results showed that *SiMAPK3* and *SiMAPKK4–2* could be induced under cold, salt, and simulated drought treatments. Twenty and 10 genes were significantly up-regulated under drought treatment and salt treatment, respectively, while the expression of 10 genes was significantly inhibited by cold treatment. Among them, the extensively studied *SiMAPK3* was significantly up-regulated under the three stresses, while *SiMAPKK6–1*, *SiMAPKK6–2*, *SiMAPK4*, *SiMAPK6*, *SiMAPK7*, *SiMAPK11*, *SiMAPK14*, *SiMAPK17–2*, and *SiMAPK20–1* were up-regulated under drought treatment and significantly down-regulated under low-temperature treatment.

### 3.9. Protein interaction network analysis of SiMAPKs and SiMapkks

**Figure 10.**
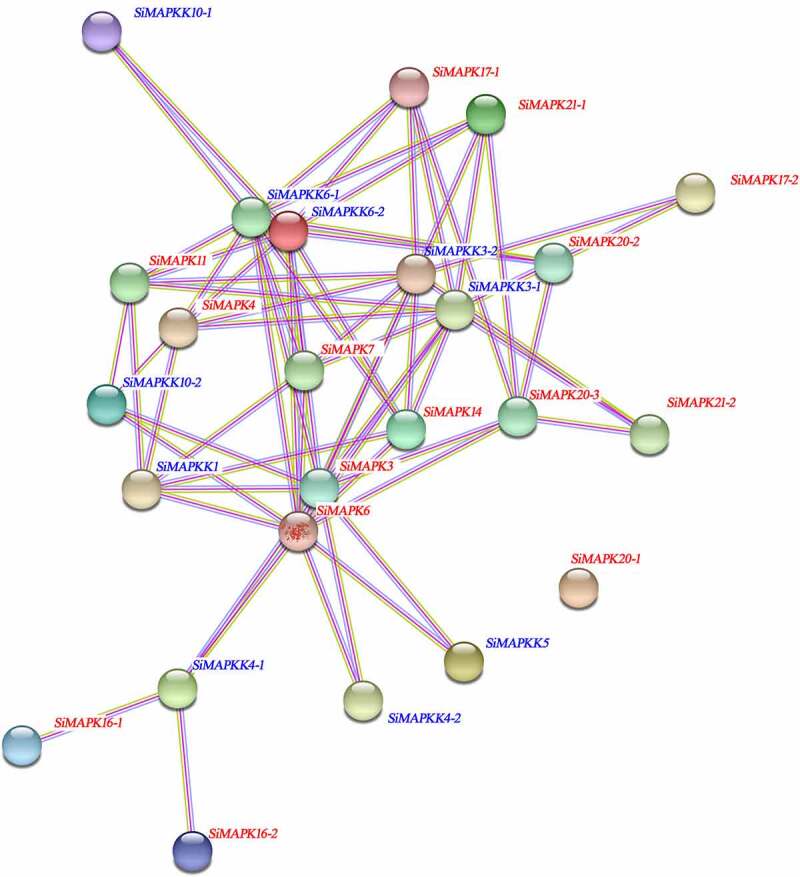
Protein interaction network of SiMAPKs and SiMAPKKs.

In order to explore the potential biological functions of foxtail millet SiMAPKs and SiMAPKK proteins, we used STRING software to predict the protein interaction network of foxtail millet SiMAPKs and SiMAPKK proteins. As shown in Figure 10, the interaction network analysis found that a total of 75 protein pairs had interactions between 24 SiMAPKs and SiMAPKKs proteins (except SiMAPK20–1). Among the SiMAPKs in group A, SiMAPK3 interacted with 10 proteins, SiMAPK6 interacted with 9 proteins, and SiMAPK3 and SiMAPK6 could interact with 7 identical SiMAPKKs (SiMAPKK1, 3–1, 4–1, 4–2, 5, 6–1 and 6–2). Among the SiMAPKs in group B, SiMAPK4 and SiMAPK11 interacted with six common proteins. Among the SiMAPKs in group C, SiMAPK7 interacted with eight proteins, SiMAPK14 interacted with six proteins, of which six proteins were identical; The above results indicated that SiMAPKs proteins of the same subfamily could interact with the same protein in different subfamilies, but there was no interaction between the same subfamily. In addition, among SiMAPKKs proteins, SiMAPKK3–1, 3–2, 6–1 and 6–2 all interacted with multiple SiMAPKs proteins, and there was basically no interaction between SiMAPKKs proteins. The above results indicated that there were frequently protein interactions between SiMAPKs and SiMAPKKs, while there were basically no interactions in the same group. In conclusion, this finding will provide some valuable information for further study of MAPK cascade family functions.

## 4. Discussion

Currently, the MAPK cascade gene family had been extensively identified in *Arabidopsis thaliana*,^5,7^ *Brachypodium distachyon*,^11^ *Gossypium spp*,^18^ *Solanum lycopersicum*,^16^ *Oryza sativa*,^10^ and other species. However, few studies concerning the MAPK cascade gene family in *Setaria italica* have been reported so far. Based on the identified sequences of MAPK cascade genes in *Brachypodium distachyon*, *Arabidopsis thaliana*, and *Oryza sativa*, a total of 68 SiMAPKKKs, 10 SiMAPKKs, and 15 SiMAPKs were identified through searching the foxtail millet genome database. The MAPKKK family is the largest component of the MAPK cascade and can be divided into three subtypes: MEKK, Raf, and ZIK.^16^ MAPKK is a double phosphorylase, and all MAPKK except the MAPKK10 homologue have the -S/T-XXXXX-S/T- motif in the activation loop and are generally classified into four groups.^5^ MAPKs, as downstream components of the MAPK cascade pathway, can be activated by MAPKKs phosphorylation during signal transduction. Generally, MAPKs have a conserved -T(E/D)Y- motif and can be classified into four groups,^51^ of which groups A-C have a -TEY- motif (MAPK3 contains a -MEY- motif), also known as the -TEY- subtype, and the group D of MAPKs is usually evolutionarily distant and has the -TDY- motif.^52^ In this study, according to the sequence alignment results and phylogenetic tree analysis, the MAPK cascade genes of foxtail millet were classified and found that there are 38 Rafs, 21 MEKKs, and 9 ZIKs in SiMAPKKKs. Among SiMAPKKs, there were three in group A, two in group B, three in group C, and two in group D. Among SiMAPKs, there were two in group A, two in group B, two in group C, and nine in group D. According to the analysis results of gene structure and conserved motifs, it is found that most of the SiMAPK cascade genes in the same group have similar gene structures and motif distribution. Based on the results of chromosomal location analysis of MAPK cascade genes in foxtail millet, it was found that SiMAPKKKs were distributed in all nine chromosomes of foxtail millet, SiMAPKKs were distributed in seven chromosomes (except chromosome VI and chromosome VII), and SiMAPKs were distributed in eight chromosomes (except chromosome VII), and the results also showed that the MAPK cascade genes of foxtail millet were unevenly distributed on the chromosomes. Gene duplication events are important in plant genome variation and will lead to the generation of new genes and genetic regulatory pathways, and gene duplication (including tandem gene duplication and segmental gene duplication) is a major driver of gene family expansion.^53^ In the present study, segmental duplications were found to be the main driving force for gene expansion of the SiMAPK cascade, and the Ka/Ks ratios of MAPK cascade genes within the genome of *Setaria italica* and between the genomes of *Setaria italica* and different species (*Arabidopsis thaliana*, *Oryza sativa*, and *Brachypodium distachyon*) were all less than 1, indicating that these genes experienced strong purifying selection pressure during evolution.^54^

In higher plants, the MAPK cascade pathway was not only involved in cell division, apoptosis, and plant growth and development but also in hormone signaling, biotic, and abiotic stress responses.^19,55^ In *Arabidopsis*, the MKK2 pathway mediated cold and salt stress signaling is critical for plant stress response.^29^ *MAPK6* rapidly activated the Na^+^/H^+^ antiporter (SOS1), thereby reducing Na^+^ ion toxicity in plants under salt stress.^56^ *AtMAPK3*, *AtMAPK4*, and *AtMAPK6* can be induced by low temperature, salt, and mechanical damage and thus can be involved in plant response to environmental stresses;^57–59^ In addition, ZmMKK4 was also revealed to be a positive regulator of salt tolerance and cold resistance in plants, and overexpression of *ZmMKK4* in *Arabidopsis* conferred tolerance to cold stress and salt stress.^60^ Maize ZmMKK1 induced by high salinity and alkali stress can act as a positive regulatory protein to improve salinity and alkalinity tolerance of plants.^28^ In this study, we analyzed the expression of foxtail millet SiMAPKKs and SiMAPKs gene by qRT-PCR and found that *SiMAPKK4–2*, *6–1*, *10–2* and *SiMAPK3, 4, 11, 14, 16–1, 16–2, 20–2, 20–3, 21–1* etc. were significantly up-regulated under salt treatment. *SiMAPKK1, 4–1, 4–2, 6–1, 6–2, 10–1* and *SiMAPK3, 4, 7, 11, 14*, 20–2, etc., were significantly up-regulated under simulated drought treatment. The expression of six genes including *SiMAPKK1, 3–1, 4–2* and *SiMAPK3, 16–1, 21–2* were significantly up-regulated under low-temperature treatment. These results will provide some reference information for the subsequent exploration of the function of MAPK cascade genes in foxtail millet.

In recent years, the crosstalk mechanism between MAPK cascades and phytohormone signaling pathways attracted much attention. Studies had shown that plant hormones such as ABA,^61^ auxin,^62^ jasmonic acid,^33^ ethylene,^63^ brassinolide,^64^ and MT^65^ were associated with the expression of MAPKs cascade genes and the regulation of plant growth by MAPK.^55^ For example, in *Arabidopsis thaliana*, there were 19 MAPK family genes including *AtMPK1*,^37^ *AtMPK2*,^66^ *AtMPK3*,^32,67^ *AtMPK7*, and *AtMKK9*^68^ were transcriptionally regulated by ABA. In addition, some studies had shown that ABA could regulate the MAP3K17/18-MKK3-MPK1/2/7/14 cascade pathway and affect the signal transduction under environmental stress in plant.^69^ Jasmonic acid was an important phytohormone during plant growth and development. It had been reported that *SlMPK6–1* and *SlMPK6–2* in tomato could positively regulate the biosynthesis of jasmonic acid and inhibited the expression of jasmonic acid-dependent defense genes.^33^ Furthermore, jasmonic acid treatment induced the expression of MAPK family genes in various plants, such as *CsMPK6, CsMPK9–1, CsMPK20–1, CsMPK20–2, CsMKK4, CsMKK6*, and *CsMEKK21–1* in cucumber,^70^ and *GrMPK2, GrMPK3, GrMPK5–1, GrMPK18, GrMPK19, GrMPK20, GrMPK22, GrMPK23, GrMPK24, GrMPK25, GrMPK27, GrMPK28*, etc., in cotton.^71^ As a new type of plant hormone, MT has been studied a lot in recent years. Studies showed that exogenous MT could activate the downstream bZIP60 transcription factor through the OXI1/MAPKKK3–MAPKK4/5/7/9–MAPK3/6 pathway and regulate the expression of BIP2, BIP3, and CNX1 genes, thereby reducing endoplasmic reticulum stress injury.^72,73^ In addition, MT and its metabolites could also participate in the regulation of redox homeostasis in plants by activating the MAPK pathway.^74^ H_2_O_2_ was a potent activator of MAPK cascades in *Arabidopsis thaliana*^35^ and could induce the expression of MAPK cascade genes such as *MPK1/MPK2*, *MPK3/MPK6*.^35–37^ In this study, in order to further understand the possible crosstalk relationship between MAPK cascade genes and phytohormones in foxtail millet, we investigated the distribution of cis-acting elements in the promoter regions of SiMAPK cascade genes, and found that most gene promoters have cis-elements related to plant hormone response elements, especially jasmonic acid response elements. This suggested that most SiMAPK cascade genes may be involved in phytohormone-mediated plant growth and stress response. To better understand the effect of exogenous phytohormones on the expression of SiMAPK cascade genes, we further analyzed the expression levels of SiMAPK and SiMAPKK genes under exogenous phytohormone treatment. It was found that five SiMAPKs and three SiMAPKKs genes were significantly up-regulated, and three SiMAPKs and one SiMAPKKs genes were down-regulated under exogenous ABA treatment. Analysis of cis-acting elements in the promoter regions of MAPK family genes revealed that almost all MAPK family genes have jasmonic acid response elements, and after jasmonic acid treatment, *SiMAPKK4–1, 4–2, 10–1* and *SiMAPK3* were significantly up-regulated, the expression of *SiMAPKK3–2, 5, 10–2* and *SiMAPK6, 11, 14, 17–1, 17–2, 20–1, 20–3* and *21–1* were inhibited. *SiMAPKK3–2, 4–2, 10–1* and *SiMAPK3, 16–1, 20–2* were found to be significantly up-regulated after exogenous application of MT. Four genes including *SiMAPKK3–1, 5* and *SiMAPK20–1, 21–1* were significantly down-regulated under the treatment of exogenous MT. Seven *SiMAPKs* and *SiMAPKKs* genes were noticed to be induced and eight genes were repressed after exogenous H_2_O_2_ treatment. The above results indicated that exogenous phytohormones or H_2_O_2_ had certain regulatory effects on the expression of *SiMAPKs* and *SiMAPKKs* genes in foxtail millet, and the changes of exogenous treatment substances would largely affect the signal transduction of the MAPK cascade pathway. The above results will provide some clues for the subsequent exploration of MAPK cascades mediating signal transduction pathways during plant growth and stress response.

As a typical signal transduction pathway in higher plants, the MAPK cascade pathway transmits stimulus signals step by step according to the principle of signal cascade amplification, forming a complex signal regulation network, thereby regulating plant growth and development and stress response processes.^75^ For example, the AtMEKK1-AtMKK2/AtMEK1-AtMAPK4/AtMAPK6 pathway could improve *Arabidopsis thaliana* resistance to low temperature and high salt stress,^29^ and MAPKKK18-MAPKK3 could positively regulate drought tolerance in *Arabidopsis thaliana*.^30^ The rice OsMKK6-OsMPK3 pathway was an important low-temperature signaling pathway that could regulate rice tolerance to cold stress,^76^ and the OsMAPKKK63-OsMAPKK1-OsMAPK4 pathway could positively regulate rice salt tolerance.^2^ In this study, we analyzed the possible interactions between SiMAPKs and SiMAPKKs proteins in foxtail millet through the protein interaction network, and found that 75 protein pairs had interactions between 24 SiMAPKs and SiMAPKKs proteins. In further analysis, it was found that the interaction of MAPK cascade proteins often occurs between different families (MAPKs and MAPKKs), and the same subfamily usually has the same interacting proteins. And according to the protein interaction network analysis, it was found that there was interaction between SiMAPKK3–2 and SiMAPK14, SiMAPKK3–2 and SiMAPK7, SiMAPKK3–2, 4–1, 4–2 and SiMAPK3, 6. The above results showed the complexity and functional diversity of the MAPK cascade pathway. From a certain perspective, this implicates that the MAPK signaling cascade pathway has strong stability and self-regulation ability. However, it also implies that it is difficult to specifically define the roles that MAPK cascade genes and their pathways fulfill in specific biological processes. In addition, some MAPK cascade genes have functional redundancy (such as *MAPK3* and *MAPK6*) and regulatory diversity (such as *MAPKKs*), which undoubtedly enhances the difficulty of exploring the function and regulatory mechanism of the MAPK cascade pathway. In this study, the functional information of the MAPK cascade genes in foxtail millet was analyzed from the aspects of screening, basic information analysis, evolutionary selection, and expression analysis of related genes of the MAPK cascade family in foxtail millet. The analysis of the functional information of MAPK cascade genes in foxtail millet provides some valuable guidelines for the subsequent exploration of the function of the MAPK cascade pathway.

## 5. Conclusion

This study is the first time to study the MAPK cascade genes in foxtail millet at the genome level. A total of 15 SiMAPKs, 10 SiMAPKKs and 68 SiMAPKKKs genes were obtained, and the identified results were analyzed by multiple sequence alignment, phylogenetic relationship construction, intron-exon structure construction, conserved motif analysis, etc. In addition, the expansion of MAPK cascade genes in foxtail millet was found to be dependent on segmental and tandem repeat events, and according to gene collinearity analysis, it was found that the MAPK cascade gene pairs between *Setaria italica* and *Arabidopsis thaliana*, *Oryza sativa, and Brachypodium distachyon* may have undergone extensive purification selection. Based on the RNA-Seq expression profiles of the SiMAPK cascade genes in different tissues, we selected some genes in the SiMAPK cascades and analyzed the expression levels of these SiMAPK cascade genes under hormone/H_2_O_2_ and abiotic stresses, and this will contribute to further research on the MAPK cascade signaling pathway in foxtail millet.

## Abbreviations

MAPKs: Mitogen-activated protein kinases
MAPKKs: Mitogen-activated protein kinase kinases
MAPKKKs: Mitogen-activated protein kinase kinase kinases
Si: *Setaria italica*
JA: Jasmonate
ABA: Abscisic acid
MeJA: Methyl jasmonate
MT: Melatonin
H_2_O_2_: Hydrogen peroxide
NJ: Neighboring joining
Ka: Nonsynonymous substitution rate
Ks: Synonymous substitution rate
CDS: Coding Sequence

## Supplementary Material

Supplemental Material
