# Supplementary material for: Identification and expression analysis of MAPK cascade gene family in foxtail millet (Setaria italica)
Source: Plant Signal Behav. 2023 Aug 16;18(1):2246228. doi: 10.1080/15592324.2023.2246228 (PMC10435010; doi:10.1080/15592324.2023.2246228)
Supplement: Supplemental Material [file KPSB_A_2246228_SM9810.zip › Table S1.docx]

Table S1 MAPK cascade proteins IDs of *Arabidopsis thaliana*, *Oryza sativa*, and *Brachypodium distachyon*

| *Arabidopsis thaliana* | | *Oryza sativa* | | *Brachypodium distachyon* | |
| --- | --- | --- | --- | --- | --- |
| Name | ID | Name | ID | Name | ID |
| MAPK | | | | | |
| *AtMAPK1* | At1g10210 | *OsMAPK3* | LOC_Os03g17700.1 | *BdMAPK3* | Bradi1g65810 |
| *AtMAPK2* | At1g59580 | *OsMAPK4* | LOC_Os10g38950.1 | *BdMAPK4* | Bradi3g32000 |
| *AtMAPK3* | At3g45640 | *OsMAPK6* | LOC_Os06g06090.1 | *BdMAPK6* | Bradi1g49100 |
| *AtMAPK4* | At4g01370 | *OsMAPK7* | LOC_Os06g48590.1 | *BdMAPK7-1* | Bradi1g34030 |
| *AtMAPK5* | At4g11330 | *OsMAPK14* | LOC_Os02g05480.1 | *BdMAPK7-2* | Bradi4g24912 |
| *AtMAPK6* | At2g43790 | *OsMAPK16* | LOC_Os11g17080.1 | *BdMAPK11* | Bradi3g16560 |
| *AtMAPK7* | At2g18170 | *OsMAPK17-1* | LOC_Os06g49430.1 | *BdMAPK14* | Bradi3g03780 |
| *AtMAPK8* | At1g18150 | *OsMAPK17-2* | LOC_Os02g04230.3 | *BdMAPK16* | Bradi2g36470 |
| *AtMAPK9* | At3g18040 | *OsMAPK20-1* | LOC_Os01g43910.1 | *BdMAPK17* | Bradi1g34700 |
| *AtMAPK10* | At3g59790 | *OsMAPK20-2* | LOC_Os05g50560.1 | *BdMAPK20-1* | Bradi2g44350 |
| *AtMAPK11* | At1g01560 | *OsMAPK20-3* | LOC_Os06g26340.1 | *BdMAPK20-2* | Bradi2g15317 |
| *AtMAPK12* | At2g46070 | *OsMAPK20-4* | LOC_Os01g47530.1 | *BdMAPK20-3* | Bradi1g41780 |
| *AtMAPK13* | At1g07880 | *OsMAPK20-5* | LOC_Os05g49140.1 | *BdMAPK20-4* | Bradi2g45870 |
| *AtMAPK14* | At4g36450 | *OsMAPK21-1* | LOC_Os05g50120.1 | *BdMAPK20-5* | Bradi2g16337 |
| *AtMAPK15* | At1g73670 | *OsMAPK21-2* | LOC_Os01g45620.1 | *BdMAPK21-1* | Bradi2g15620 |
| *AtMAPK16* | At5g19010 |  |  | *BdMAPK21-2* | Bradi2g45010 |
| *AtMAPK17* | At2g01450 |  |  |  |  |
| *AtMAPK18* | At1g53510 |  |  |  |  |
| *AtMAPK19* | At3g14720 |  |  |  |  |
| *AtMAPK20* | At2g42880 |  |  |  |  |
| MAPKK | | | | | |
| *AtMAPKK1* | At4g26070 | *OsMAPKK1* | LOS_Os06g05520 | *BdMAPKK1* | Bradi1g51000 |
| *AtMAPKK2* | At4g29810 | *OsMAPKK3* | LOS_Os06g27890 | *BdMAPKK3-1* | Bradi1g41860 |
| *AtMAPKK3* | At5g40440 | *OsMAPKK4* | LOS_Os02g54600 | *BdMAPKK3-2* | Bradi4g39490 |
| *AtMAPKK4* | At1g51660 | *OsMAPKK5* | LOS_Os06g09180 | *BdMAPKK3-3* | Bradi3g11260 |
| *AtMAPKK5* | At3g21220 | *OsMAPKK6* | LOS_Os01g32660 | *BdMAPKK4* | Bradi3g53650 |
| *AtMAPKK6* | At5g56580 | *OsMAPKK10-1* | LOS_Os02g46760 | *BdMAPKK5* | Bradi1g46880 |
| *AtMAPKK7* | At1g18350 | *OsMAPKK10-2* | LOS_Os03g12390 | *BdMAPKK6* | Bradi1g75150 |
| *AtMAPKK8* | At3g06230 | *OsMAPKK10-3* | LOS_Os03g50550 | *BdMAPKK10-1* | Bradi1g11525 |
| *AtMAPKK9* | At1g73500 |  |  | *BdMAPKK10-2* | Bradi1g69400 |
| *AtMAPKK10* | At1g32320 |  |  | *BdMAPKK10-3* | Bradi1g10800 |
|  |  |  |  | *BdMAPKK10-4* | Bradi1g10770 |
|  |  |  |  | *BdMAPKK10-5* | Bradi1g10790 |
| MAPKKK | | | | | |
| *AtMAPKKK1* | At1g09000 | *OsMAPKKK1* | LOC_Os03g06410 | *BdMAPKKK1* | Bradi5g24870 |
| *AtMAPKKK2* | At1g54960 | *OsMAPKKK2* | LOC_Os10g29540 | *BdMAPKKK2* | Bradi1g28950 |
| *AtMAPKKK3* | At1g53570 | *OsMAPKKK3* | LOC_Os02g32610 | *BdMAPKKK4* | Bradi1g47570 |
| *AtMAPKKK4* | At1g63700 | *OsMAPKKK4* | LOC_Os02g12810 | *BdMAPKKK3* | Bradi3g60210 |
| *AtMAPKKK5* | At5g66850 | *OsMAPKKK5* | LOC_Os12g37570 | *BdMAPKKK5* | Bradi3g59510 |
| *AtMAPKKK6* | At3g07980 | *OsMAPKKK6* | LOC_Os02g50970 | *BdMAPKKK6* | Bradi1g74480 |
| *AtMAPKKK7* | At3g13530 | *OsMAPKKK7* | LOC_Os06g12590 | *BdMAPKKK7* | Bradi1g45040 |
| *AtMAPKKK8* | At4g08500 | *OsMAPKKK8* | LOC_Os11g10100 | *BdMAPKKK8* | Bradi5g18180 |
| *AtMAPKKK9* | At4g08480 | *OsMAPKKK9* | LOC_Os02g44642 | *BdMAPKKK9* | Bradi1g30720 |
| *AtMAPKKK10* | At4g08470 | *OsMAPKKK10* | LOC_Os04g47240 | *BdMAPKKK10* | Bradi2g46340 |
| *AtMAPKKK11* | At4g12020 | *OsMAPKKK11* | LOC_Os07g02780 | *BdMAPKKK11* | Bradi3g51380 |
| *AtMAPKKK12* | At3g06030 | *OsMAPKKK12* | LOC_Os09g39320 | *BdMAPKKK12* | Bradi3g27120 |
| *AtMAPKKK13* | AT1G07150 | *OsMAPKKK13* | LOC_Os09g21510 | *BdMAPKKK13* | Bradi3g09170 |
| *AtMAPKKK14* | AT2G30040 | *OsMAPKKK14* | LOC_Os04g52140 | *BdMAPKKK14* | Bradi3g08260 |
| *AtMAPKKK15* | AT5G55090 | *OsMAPKKK15* | LOC_Os08g32600 | *BdMAPKKK15* | Bradi4g04470 |
| *AtMAPKKK16* | AT4G26890 | *OsMAPKKK16* | LOC_Os04g35700 | *BdMAPKKK16* | Bradi1g23970 |
| *AtMAPKKK17* | AT2G32510 | *OsMAPKKK17* | LOC_Os09g37230 | *BdMAPKKK17* | Bradi4g38400 |
| *AtMAPKKK18* | AT1G05100 | *OsMAPKKK18* | LOC_Os03g55560 | *BdMAPKKK18* | Bradi3g44710 |
| *AtMAPKKK19* | AT5G67080 | *OsMAPKKK19* | LOC_Os02g35010 | *BdMAPKKK19* | Bradi1g07650 |
| *AtMAPKKK20* | AT3G50310 | *OsMAPKKK20* | LOC_Os07g38530 | *BdMAPKKK20* | Bradi5g21330 |
| *AtMAPKKK21* | AT4G36950 | *OsMAPKKK21* | LOC_Os07g25680 | *BdMAPKKK21* | Bradi4g36880 |
| *AtCTR1* | At5g03730 | *OsMAPKKK22* | LOC_Os03g49640 | *BdMAPKKK22* | Bradi2g39350 |
| *AtEDR1* | At1g08720 | *OsMAPKKK23* | LOC_Os12g40279 | *BdMAPKKK23* | Bradi4g29500 |
| *AtRaf3* | At5g11850 | *OsMAPKKK24* | LOC_Os04g56530 | *BdMAPKKK24* | Bradi1g60340 |
| *AtRaf4* | At1g18160 | *OsMAPKKK25* | LOC_Os02g38080 | *BdMAPKKK25* | Bradi3g36080 |
| *AtRaf5* | At1g73660 | *OsMAPKKK26* | LOC_Os07g29330 | *BdMAPKKK26* | Bradi1g58810 |
| *AtRaf6* | At4g24480 | *OsMAPKKK27* | LOC_Os03g43760 | *BdMAPKKK27* | Bradi4g22760 |
| *AtRaf7* | At3g06620 | *OsMAPKKK28* | LOC_Os03g15570 | *BdMAPKKK28* | Bradi3g51460 |
| *AtRaf8* | At3g06630 | *OsMAPKKK29* | LOC_Os02g45130 | *BdMAPKKK29* | Bradi1g10970 |
| *AtRaf9* | At3g06640 | *OsMAPKKK30* | LOC_Os02g02780 | *BdMAPKKK30* | Bradi3g01850 |
| *AtRaf10* | At5g49470 | *OsMAPKKK31* | LOC_Os01g45380 | *BdMAPKKK31* | Bradi2g06260 |
| *AtRaf11* | At1g67890 | *OsMAPKKK32* | LOC_Os08g12750 | *BdMAPKKK32* | Bradi2g19590 |
| *AtRaf12* | At4g23050 | *OsMAPKKK33* | LOC_Os02g07790 | *BdMAPKKK33* | Bradi3g48360 |
| *AtRaf13* | At2g31010 | *OsMAPKKK34* | LOC_Os05g50190 | *BdMAPKKK34* | Bradi1g67400 |
| *AtRaf14* | At2g42630 | *OsMAPKKK35* | LOC_Os02g54510 | *BdMAPKKK35* | Bradi2g49700 |
| *AtRaf15* | At3g58640 | *OsMAPKKK36* | LOC_Os05g01780 | *BdMAPKKK36* | Bradi2g57470 |
| *AtRaf16* | At1g04700 | *OsMAPKKK37* | LOC_Os04g51950 | *BdMAPKKK37* | Bradi3g05520 |
| *AtRaf17* | At1g14000 | *OsMAPKKK38* | LOC_Os06g45300 | *BdMAPKKK38* | Bradi3g18150 |
| *AtRaf18* | At1g16270 | *OsMAPKKK39* | LOC_Os06g08280 | *BdMAPKKK39* | Bradi1g28110 |
| *AtRaf19* | At1g62400 | *OsMAPKKK40* | LOC_Os01g48330 | *BdMAPKKK40* | Bradi2g49790 |
| *AtRaf20* | At1g79570 | *OsMAPKKK41* | LOC_Os06g43840 | *BdMAPKKK41* | Bradi1g14000 |
| *AtRaf21* | At2g17700 | *OsMAPKKK42* | LOC_Os03g60150 | *BdMAPKKK42* | Bradi1g35350 |
| *AtRaf22* | At2g24360 | *OsMAPKKK43* | LOC_Os06g50920 | *BdMAPKKK43* | Bradi1g04080 |
| *AtRaf23* | At2g31800 | *OsMAPKKK44* | LOC_Os02g14530 | *BdMAPKKK44* | Bradi5g10670 |
| *AtRaf24* | At2g35050 | *OsMAPKKK45* | LOC_Os06g43030 | *BdMAPKKK45* | Bradi3g47600 |
| *AtRaf25* | At2g43850 | *OsMAPKKK46* | LOC_Os11g06140 | *BdMAPKKK46* | Bradi2g44910 |
| *AtRaf26* | At4g14780 | *OsMAPKKK47* | LOC_Os07g08750 | *BdMAPKKK47* | Bradi2g15560 |
| *AtRaf27* | At4g18950 | *OsMAPKKK48* | LOC_Os01g01740 | *BdMAPKKK48* | Bradi2g00670 |
| *AtRaf28* | At4g31170 | *OsMAPKKK49* | LOC_Os05g44290 | *BdMAPKKK49* | Bradi1g20390 |
| *AtRaf29* | At4g35780 | *OsMAPKKK50* | LOC_Os12g02250 | *BdMAPKKK50* | Bradi4g44430 |
| *AtRaf30* | At4g38470 | *OsMAPKKK51* | LOC_Os01g54350 | *BdMAPKKK51* | Bradi2g47510 |
| *AtRaf31* | At5g01850 | *OsMAPKKK52* | LOC_Os12g06490 | *BdMAPKKK52* | Bradi2g47480 |
| *AtRaf32* | At5g40540 | *OsMAPKKK53* | LOC_Os11g02305 | *BdMAPKKK53* | Bradi2g47490 |
| *AtRaf33* | At5g50000 | *OsMAPKKK54* | LOC_Os03g28300 | *BdMAPKKK54* | Bradi1g23320 |
| *AtRaf34* | At5g50180 | *OsMAPKKK55* | LOC_Os01g50400 | *BdMAPKKK55* | Bradi2g47500 |
| *AtRaf35* | At5g57610 | *OsMAPKKK56* | LOC_Os05g01780 | *BdMAPKKK56* | Bradi4g41940 |
| *AtRaf36* | At5g58950 | *OsMAPKKK57* | LOC_Os05g46750 | *BdMAPKKK57* | Bradi3g10890 |
| *AtRaf37* | At5g66710 | *OsMAPKKK58* | LOC_Os03g39150 | *BdMAPKKK58* | Bradi2g17820 |
| *AtRaf38* | At3g01490 | *OsMAPKKK59* | LOC_Os12g41260 | *BdMAPKKK59* | Bradi2g17830 |
| *AtRaf39* | At3g22750 | *OsMAPKKK60* | LOC_Os03g53410 | *BdMAPKKK60* | Bradi1g65500 |
| *AtRaf40* | At3g24720 | *OsMAPKKK61* | LOC_Os01g10450 | *BdMAPKKK61* | Bradi1g41850 |
| *AtRaf41* | At3g27560 | *OsMAPKKK62* | LOC_Os01g50420 | *BdMAPKKK62* | Bradi4g09990 |
| *AtRaf42* | At3g46920 | *OsMAPKKK63* | LOC_Os01g50370 | *BdMAPKKK63* | Bradi3g45660 |
| *AtRaf43* | At3g46930 | *OsMAPKKK64* | LOC_Os07g39520 | *BdMAPKKK64* | Bradi4g02900 |
| *AtRaf44* | At3g50720 | *OsMAPKKK65* | LOC_Os07g43900 | *BdMAPKKK65* | Bradi1g00580 |
| *AtRaf45* | At3g50730 | *OsMAPKKK66* | LOC_Os10g04010 | *BdMAPKKK66* | Bradi1g14010 |
| *AtRaf46* | At3g59830 | *OsMAPKKK67* | LOC_Os10g04000 | *BdMAPKKK67* | Bradi3g45790 |
| *AtRaf47* | At3g58760 | *OsMAPKKK68* | LOC_Os12g30570 | *BdMAPKKK68* | Bradi2g17800 |
| *AtRaf48* | At3g63260 | *OsMAPKKK69* | LOC_Os05g46760 | *BdMAPKKK69* | Bradi2g17840 |
| *AtZIK1* | AT3G51630 | *OsMAPKKK70* | LOC_Os01g50410 | *BdMAPKKK70* | Bradi3g57740 |
| *AtZIK2* | AT5G58350 | *OsMAPKKK71* | LOC_Os02g21700 | *BdMAPKKK71* | Bradi4g24830 |
| *AtZIK3* | At3g22420 | *OsMAPKKK72* | LOC_Os01g54480 | *BdMAPKKK72* | Bradi4g24840 |
| *AtZIK4* | AT3G04910 | *OsMAPKKK73* | LOC_Os03g18170 | *BdMAPKKK73* | Bradi3g13050 |
| *AtZIK5* | AT3G18750 | *OsMAPKKK74* | LOC_Os01g66860 | *BdMAPKKK74* | Bradi3g13060 |
| *AtZIK6* | AT5G41990 | *OsMAPKKK75* | LOC_Os02g39560 | *BdMAPKKK75* | Bradi4g41870 |
| *AtZIK7* | AT1G49160 |  |  |  |  |
| *AtZIK8* | AT5G55560 |  |  |  |  |
| *AtZIK9* | AT5G28080 |  |  |  |  |
| *AtZIK10* | AT1G64630 |  |  |  |  |
| *AtZIK11* | AT3G48260 |  |  |  |  |

Note: The screening template for the MAPK family genes in foxtail millet (*Setaria italica*) was shown in the table, they were respectively obtained from the genome-wide databases of *Arabidopsis thaliana* (TAIR database: https://www.arabidopsis.org/), *Oryza sativa* (RGAP database: http://rice.uga.edu/), and *Brachypodium distachyon* (PlantGDA database: http://www.plantgdb.org/BdGDB/).
