# Supplementary material for: Identification and expression analysis of MAPK cascade gene family in foxtail millet (Setaria italica)
Source: Plant Signal Behav. 2023 Aug 16;18(1):2246228. doi: 10.1080/15592324.2023.2246228 (PMC10435010; doi:10.1080/15592324.2023.2246228)
Supplement: Supplemental Material [file KPSB_A_2246228_SM9810.zip › Table S2.docx]

Table S2 Primers for quantitative real-time PCR (qRT-PCR)

| Gene | Upstream primer | Downstream primer |
| --- | --- | --- |
| *SiActin* | CAGTGGACGCACAACAGGTAT | AGCAAGGTCAAGACGGAGAA |
| *SiMAPK3* | CCAGTACAACATCTTCGGCA | AAGTTCATCACCGAGCAGAC |
| *SiMAPK4* | GTATCAGTTGCTACGAGGGC | GCATCCAACTGACCAGACAT |
| *SiMAPK6* | GTGGTCTGTGGGCTGTAT | TTTGGTGTCCCGATGAG |
| *SiMAPK7* | ATGTATGGTCTGTTGGCTGC | AGAGGATGTGCTTGTGGGTA |
| *SiMAPK11* | AAGGACATCATACGCCCACC | AAGACATTCGCCGAGTGGAC |
| *SiMAPK14* | ACCTCAGGGGCTTTCCAATG | GTCACAGCAGAGGAGCAACT |
| *SiMAPK16-1* | CAACCAATGTCACGGAGGAT | ACCACCTGCTTGAGGAACT |
| *SiMAPK16-2* | GACCAGAAGAAGAAGGCACC | AGAGCAAACCACACCATAGC |
| *SiMAPK17-1* | CCAACTTCTTCGTGCTCT | GCCACATAATCCGTCCA |
| *SiMAPK17-2* | CGTTTACTTGCGTTTGACCC | TTGTGCTGAAGGCTCCATT |
| *SiMAPK20-1* | CCACAAAGAGTGCCAACAGC | AGTTCATTGCCAACCTCCGT |
| *SiMAPK20-2* | GCCAAGGTAGTGCCACAGAT | ACGCAGCTCTTTCCATGACA |
| *SiMAPK20-3* | GACCAGAAGAAGAAGGCACC | TATGGGTGTCCAGAGCAGAG |
| *SiMAPK21-1* | TGATGGGAACCAGATGACCT | TCGCTTATGCCATCAGTCAC |
| *SiMAPK21-2* | GGGACCTCAAGCCCAAGA | CGCTGAAGGCGTATCGT |
| *SiMAPKK1* | TTTTCCGTTTCCTCCCTGTG | GTGAAAACTGGTCCGATGGT |
| *SiMAPKK3-1* | TTCGTGTGTCTGGGTCATTC | TTCCTCTCTAAAACGACCGAC |
| *SiMAPKK3-2* | CGAGAAGGAGAAGAGGCAAC | TGCTCCCTGGAACTCAACTA |
| *SiMAPKK4-1* | GAACTTCATCAGCCTGTGCC | CTCGTCAGTCACTCTCATGT |
| *SiMAPKK4-2* | AGCCCTCCAACCTCCTGAT | CCGGCGTAGCCCATCGTA |
| *SiMAPKK5* | TCTCGAGTTCTACCTTGGCA | CTGATGAACCCCCTGAACTC |
| *SiMAPKK6-1* | AAGGATGGTGAACTACGGCT | ACCTTCAGGTTTGTCGATTG |
| *SiMAPKK6-2* | TCAACCGTAAAGGGGAAGTC | CGACAATAGCCTCCAGTAGT |
| *SiMAPKK10-1* | GCCTACTCCGGCGACTACGA | CGACGAACGGGTGTTCCAG |
| *SiMAPKK10-2* | ATCGGCGACTTCAACACTTC | GCAGACGCCTTCTTCACATC |
| *SiMAPKK5* | CAAGCCATCCAACCTCCTCA | TAGTTGCCGTCGTTGAGGTC |
| *SiMAPKK10-2* | CGGCGACTTCAACACTTCCA | ACCTCTCGGGGCTGAAGTAG |

Note: PCR amplification primers were designed using primer 5.0 and Primer-BLAST (https://www.ncbi.nlm.nih.gov/tools/primer-blast/) and were synthesized by AuGCT Biotechnology (Beijing, China).
