# Supplementary material for: Identification and expression analysis of MAPK cascade gene family in foxtail millet (Setaria italica)
Source: Plant Signal Behav. 2023 Aug 16;18(1):2246228. doi: 10.1080/15592324.2023.2246228 (PMC10435010; doi:10.1080/15592324.2023.2246228)
Supplement: Supplemental Material [file KPSB_A_2246228_SM9810.zip › Table S3.docx]

Table S3 Ka/Ks ratios of duplicated MAPK cascade gene pairs in *Setaria italic*

| Gene ID1 | Gene Name1 | Gene ID2 | Gene Name2 | Duplication | Ka | Ks | Ka/Ks | Selection pressure |
| --- | --- | --- | --- | --- | --- | --- | --- | --- |
| SETIT_017554mg | *SiMAPK14* | SETIT_006708mg | *SiMAPK7* | Segmental | 0.038132267 | 0.990393803 | 0.038502126 | Purifying selection |
| SETIT_021565mg | *SiMAPK21-1* | SETIT_004793mg | *SiMAPK21-2* | Segmental | 0.104721432 | 1.45879268 | 0.071786371 | Purifying selection |
| SETIT_017572mg | *SiMAPKK4-2* | SETIT_006813mg | *SiMAPKK5* | Segmental | 0.109453548 | 0.446815382 | 0.244963698 | Purifying selection |
| SETIT_016157mg | *SiRAF4* | SETIT_005743mg | *SiRAF3* | Segmental | 0.205184557 | 0.941063294 | 0.21803481 | Purifying selection |
| SETIT_034087mg | *SiRAF8* | SETIT_034063mg | *SiRAF5* | Segmental | 0.247742243 | 0.939413008 | 0.263720261 | Purifying selection |
| SETIT_021297mg | *SiRAF9* | SETIT_027523mg | *SiRAF34* | Segmental | 0.245384973 | 0.795953945 | 0.308290416 | Purifying selection |
| SETIT_015231mg | *SiRAF20* | SETIT_010212mg | *SiRAF22* | Segmental | 0.085215802 | 1.894097781 | 0.044990181 | Purifying selection |
| SETIT_024868mg | *SiRAF38* | SETIT_013509mg | *SiRAF32* | Segmental | 0.341426482 | 0.971518172 | 0.351436023 | Purifying selection |
| SETIT_024868mg | *SiRAF38* | SETIT_009795mg | *SiRAF36* | Segmental | 0.356494887 | 2.19482752 | 0.162425012 | Purifying selection |
| SETIT_009795mg | *SiRAF36* | SETIT_026192mg | *SiRAF37* | Segmental | 0.168546026 | 0.713781869 | 0.236131 | Purifying selection |
| SETIT_011766mg | *SiZIK9* | SETIT_027310mg | *SiZIK6* | Segmental | 0.273904152 | 1.398086014 | 0.195913663 | Purifying selection |
| SETIT_016275mg | *SiMAPKKK2* | SETIT_009321mg | *SiMAPKKK1* | Segmental | 0.140283028 | 0.953241542 | 0.147164199 | Purifying selection |
| SETIT_029131mg | *SiMAPKKK5* | SETIT_014949mg | *SiMAPKKK4* | Segmental | 0.228050375 | 0.904530654 | 0.252120118 | Purifying selection |
| SETIT_021980mg | *SiMAPKKK18* | SETIT_004003mg | *SiMAPKKK12* | Segmental | 0.278706385 | 0.650134356 | 0.428690443 | Purifying selection |

Note: The data in the table were analyzed and calculated using TBtools software and MCScanX toolkit; the gene information was obtained from the genome-wide database of foxtail millet (*Setaria italica*).
