# Supplementary material for: Identification and expression analysis of MAPK cascade gene family in foxtail millet (Setaria italica)
Source: Plant Signal Behav. 2023 Aug 16;18(1):2246228. doi: 10.1080/15592324.2023.2246228 (PMC10435010; doi:10.1080/15592324.2023.2246228)
Supplement: Supplemental Material [file KPSB_A_2246228_SM9810.zip › Table S4.docx]

Table S4 The orthologous relationship between *Setaria italica* and *Arabidopsis thaliana* and the type of selection pressure.

| Gene ID1 | Gene ID2 | Ka | Ks | Ka/Ks | Selection pressure |
| --- | --- | --- | --- | --- | --- |
| SETIT_003916mg | AT3G59830.1 | 0.30638037 | NaN | NaN | NaN |
| SETIT_001285mg | AT4G18950.1 | 0.333875927 | NaN | NaN | NaN |
| SETIT_010212mg | AT4G31170.3 | 0.183685167 | NaN | NaN | NaN |
| SETIT_021645mg | AT4G01595.1 | 0.308635334 | NaN | NaN | NaN |
| SETIT_039013mg | AT1G07150.1 | 0.60979051 | NaN | NaN | NaN |
| SETIT_039013mg | AT2G30040.1 | 0.700025628 | NaN | NaN | NaN |
| SETIT_035970mg | AT3G22750.1 | 0.336983578 | NaN | NaN | NaN |
| SETIT_036191mg | AT3G22750.1 | 0.210337977 | 2.5556904 | 0.082301823 | Purifying selection |
| SETIT_036191mg | AT4G14780.1 | 0.227872776 | NaN | NaN | NaN |
| SETIT_035970mg | AT4G14780.1 | 0.321437768 | NaN | NaN | NaN |
| SETIT_034839mg | AT5G58520.1 | 0.362149795 | NaN | NaN | NaN |

Note: The data in the table were analyzed and calculated using TBtools software and MCScanX toolkit; the gene information was obtained from the genome-wide databases of foxtail millet (*Setaria italica*) and *Arabidopsis thaliana.*
