# Supplementary material for: Identification and expression analysis of MAPK cascade gene family in foxtail millet (Setaria italica)
Source: Plant Signal Behav. 2023 Aug 16;18(1):2246228. doi: 10.1080/15592324.2023.2246228 (PMC10435010; doi:10.1080/15592324.2023.2246228)
Supplement: Supplemental Material [file KPSB_A_2246228_SM9810.zip › Table S5.docx]

Table S5 The orthologous relationship between *Setaria italica* and *Oryza sativa* and the type of selection pressure

| Gene ID1 | Gene ID2 | Ka | Ks | Ka/Ks | Selection pressure |
| --- | --- | --- | --- | --- | --- |
| SETIT_028893mg | Os03t0401100-01 | 0.42697172 | 1.003262705 | 0.425583168 | Purifying selection |
| SETIT_029452mg | Os07t0475900-01 | 0.089531812 | 0.515541936 | 0.17366543 | Purifying selection |
| SETIT_029157mg | Os07t0572800-01 | 0.085831043 | 0.693148446 | 0.123827794 | Purifying selection |
| SETIT_032842mg | Os07t0584100-01 | 0.107351953 | 0.273032266 | 0.393184126 | Purifying selection |
| SETIT_029825mg | Os07t0632800-01 | 0.030466769 | 0.457032973 | 0.066662081 | Purifying selection |
| SETIT_029029mg | Os07t0119000-01 | 0.157033512 | 0.479511393 | 0.327486509 | Purifying selection |
| SETIT_029371mg | Os07t0185000-01 | 0.229862456 | 0.396533423 | 0.579679903 | Purifying selection |
| SETIT_029131mg | Os08t0421800-01 | 0.169540513 | 1.046384236 | 0.162025103 | Purifying selection |
| SETIT_029131mg | Os09t0383300-01 | 0.12103062 | 0.604767577 | 0.200127494 | Purifying selection |
| SETIT_029013mg | Os09t0566550-01 | 0.075460351 | 0.528563488 | 0.142764971 | Purifying selection |
| SETIT_015231mg | Os04t0608900-01 | 0.093061127 | 1.578335854 | 0.058961549 | Purifying selection |
| SETIT_014949mg | Os08t0421800-01 | 0.165410624 | 0.636005428 | 0.260077378 | Purifying selection |
| SETIT_013899mg | Os08t0157000-00 | 0.048640357 | 0.623655148 | 0.077992393 | Purifying selection |
| SETIT_015231mg | Os08t0224100-01 | 0.063461737 | 0.881869745 | 0.07196271 | Purifying selection |
| SETIT_014949mg | Os09t0383300-01 | 0.29638523 | 1.006483162 | 0.294476094 | Purifying selection |
| SETIT_000788mg | Os01t0665200-01 | 0.057530825 | 0.569274104 | 0.101059973 | Purifying selection |
| SETIT_001285mg | Os01t0748600-02 | 0.069665711 | 0.40060844 | 0.173899757 | Purifying selection |
| SETIT_003916mg | Os01t0892800-02 | 0.134823738 | 0.496760526 | 0.2714059 | Purifying selection |
| SETIT_000725mg | Os01t0629900-01 | 0.034637126 | 0.561760763 | 0.061658145 | Purifying selection |
| SETIT_001833mg | Os01t0641000-01 | 0.05837305 | 0.422593781 | 0.138130405 | Purifying selection |
| SETIT_004793mg | Os01t0643800-01 | 0.075896861 | 0.504987639 | 0.150294493 | Purifying selection |
| SETIT_000788mg | Os05t0566400-01 | 0.094660631 | 0.938463655 | 0.100867658 | Purifying selection |
| SETIT_004793mg | Os05t0576800-01 | 0.110829776 | 1.780091365 | 0.062260723 | Purifying selection |
| SETIT_001833mg | Os05t0577700-01 | 0.102851885 | 0.962713054 | 0.106835452 | Purifying selection |
| SETIT_011766mg | Os11t0160300-01 | 0.273187466 | 1.226560049 | 0.222726532 | Purifying selection |
| SETIT_009795mg | Os12t0163800-01 | 0.075598281 | 0.530770478 | 0.142431209 | Purifying selection |
| SETIT_011766mg | Os12t0162100-02 | 0.157388718 | 0.696483655 | 0.225976184 | Purifying selection |
| SETIT_009480mg | Os02t0555900-00 | 0.363804025 | 1.132851655 | 0.321140039 | Purifying selection |
| SETIT_009321mg | Os02t0666300-01 | 0.165610546 | 0.913599366 | 0.181272615 | Purifying selection |
| SETIT_009480mg | Os04t0437600-01 | 0.133650955 | 0.567734911 | 0.235410844 | Purifying selection |
| SETIT_009321mg | Os04t0559800-01 | 0.079860536 | 0.554870657 | 0.143926401 | Purifying selection |
| SETIT_010212mg | Os04t0608900-01 | 0.031485281 | 0.715232614 | 0.044021037 | Purifying selection |
| SETIT_009672mg | Os04t0610900-01 | 0.079968803 | 0.573777319 | 0.13937254 | Purifying selection |
| SETIT_010212mg | Os08t0224100-01 | 0.069626295 | 0.912623219 | 0.076292487 | Purifying selection |
| SETIT_021565mg | Os01t0643800-01 | 0.108341835 | 1.353706575 | 0.08003347 | Purifying selection |
| SETIT_021560mg | Os01t0665200-01 | 0.118980484 | 0.92813614 | 0.128192922 | Purifying selection |
| SETIT_021033mg | Os12t0594300-00 | 0.172476668 | 0.563199048 | 0.306244602 | Purifying selection |
| SETIT_024868mg | Os04t0686650-00 | 0.153462281 | 0.718425668 | 0.213609129 | Purifying selection |
| SETIT_021645mg | Os05t0143500-02 | 0.052498335 | 0.560265128 | 0.093702664 | Purifying selection |
| SETIT_021560mg | Os05t0566400-01 | 0.081344929 | 0.553217659 | 0.147039646 | Purifying selection |
| SETIT_021565mg | Os05t0576800-01 | 0.074533603 | 0.57827215 | 0.128890182 | Purifying selection |
| SETIT_021438mg | Os05t0108300-01 | 0.112527118 | 0.765203045 | 0.147055241 | Purifying selection |
| SETIT_020144mg | Os02t0322400-00 | 0.139359446 | 0.412481233 | 0.337856452 | Purifying selection |
| SETIT_016544mg | Os02t0555900-00 | 0.320979021 | 0.823696979 | 0.38968095 | Purifying selection |
| SETIT_017053mg | Os02t0608500-01 | 0.088088493 | 0.522807344 | 0.168491308 | Purifying selection |
| SETIT_016275mg | Os02t0666300-01 | 0.090404008 | 0.492549635 | 0.18354294 | Purifying selection |
| SETIT_016157mg | Os02t0743500-02 | 0.109888646 | 0.550863204 | 0.199484455 | Purifying selection |
| SETIT_017572mg | Os02t0787300-01 | 0.049514651 | 0.295343237 | 0.167651211 | Purifying selection |
| SETIT_017554mg | Os02t0148100-01 | 0.013639435 | 0.692917729 | 0.019684061 | Purifying selection |
| SETIT_017293mg | Os02t0174200-01 | 0.037034765 | 0.583839508 | 0.063433126 | Purifying selection |
| SETIT_016359mg | Os02t0241600-01 | 0.09578029 | 0.600438627 | 0.159517202 | Purifying selection |
| SETIT_016275mg | Os04t0559800-01 | 0.131780951 | 0.910432464 | 0.144745443 | Purifying selection |
| SETIT_016544mg | Os04t0437600-01 | 0.300600018 | 0.911100913 | 0.329930542 | Purifying selection |
| SETIT_017293mg | Os06t0663400-01 | 0.087321986 | 0.902765898 | 0.096727165 | Purifying selection |
| SETIT_017554mg | Os06t0699400-01 | 0.037579823 | 1.147781337 | 0.032741274 | Purifying selection |
| SETIT_016957mg | Os06t0708000-01 | 0.148957722 | 0.881852555 | 0.168914543 | Purifying selection |
| SETIT_017572mg | Os06t0191300-01 | 0.100395992 | 0.354548249 | 0.283165949 | Purifying selection |
| SETIT_016157mg | Os06t0232100-02 | 0.140823238 | 0.890557988 | 0.158129217 | Purifying selection |
| SETIT_027310mg | Os11t0160300-01 | 0.156453833 | 0.902645772 | 0.173328051 | Purifying selection |
| SETIT_026197mg | Os11t0271100-01 | 0.064488343 | 0.667675819 | 0.096586309 | Purifying selection |
| SETIT_026073mg | Os11t0207200-01 | 0.090891866 | 0.427747397 | 0.212489583 | Purifying selection |
| SETIT_027310mg | Os12t0162100-02 | 0.276319049 | 1.131468695 | 0.244212722 | Purifying selection |
| SETIT_006813mg | Os02t0787300-01 | 0.069482734 | 0.42187146 | 0.164701196 | Purifying selection |
| SETIT_006708mg | Os02t0148100-01 | 0.035616792 | 1.145432377 | 0.031094626 | Purifying selection |
| SETIT_005743mg | Os02t0743500-02 | 0.215359851 | 0.904512421 | 0.238094963 | Purifying selection |
| SETIT_005743mg | Os06t0232100-02 | 0.091236427 | 0.577611077 | 0.157954774 | Purifying selection |
| SETIT_006786mg | Os06t0147800-01 | 0.096887134 | 0.608328336 | 0.159267831 | Purifying selection |
| SETIT_006144mg | Os06t0708000-01 | 0.048571959 | 0.486444799 | 0.099850917 | Purifying selection |
| SETIT_008355mg | Os06t0636600-01 | 0.019978984 | 0.221563575 | 0.090172691 | Purifying selection |
| SETIT_005733mg | Os06t0646400-01 | 0.097875827 | 0.536212895 | 0.182531654 | Purifying selection |
| SETIT_006611mg | Os06t0154500-01 | 0.03238187 | 0.405330408 | 0.07989006 | Purifying selection |
| SETIT_006813mg | Os06t0191300-01 | 0.085425143 | 0.473864201 | 0.180273469 | Purifying selection |
| SETIT_006708mg | Os06t0699400-01 | 0.010718722 | 0.649972968 | 0.016491027 | Purifying selection |
| SETIT_034087mg | Os10t0430900-01 | 0.160648263 | 0.511044478 | 0.314352801 | Purifying selection |
| SETIT_036240mg | Os10t0533600-01 | 0.02188186 | 0.43291988 | 0.050544826 | Purifying selection |
| SETIT_036191mg | Os12t0605900-01 | 0.238202814 | 0.92369945 | 0.257879134 | Purifying selection |
| SETIT_034802mg | Os03t0262200-00 | 0.121819016 | 0.585208822 | 0.208163329 | Purifying selection |
| SETIT_034087mg | Os03t0160100-01 | 0.229122987 | 0.794521476 | 0.288378596 | Purifying selection |
| SETIT_039120mg | Os03t0262200-00 | 0.290205338 | 1.52677662 | 0.190077143 | Purifying selection |
| SETIT_036191mg | Os03t0638800-01 | 0.023589168 | 0.52715914 | 0.044747717 | Purifying selection |
| SETIT_039120mg | Os03t0703400-01 | 0.09376117 | 0.573712892 | 0.163428731 | Purifying selection |
| SETIT_035970mg | Os03t0745700-00 | 0.125425406 | 0.491835776 | 0.255014807 | Purifying selection |
| SETIT_034335mg | Os03t0764300-01 | 0.121351103 | 0.62004201 | 0.195714325 | Purifying selection |
| SETIT_035834mg | Os03t0816100-01 | 0.041057481 | 0.672081782 | 0.061090008 | Purifying selection |
| SETIT_034063mg | Os03t0160100-01 | 0.085230404 | 0.502107726 | 0.169745256 | Purifying selection |
| SETIT_036560mg | Os03t0225100-02 | 0.051606693 | 0.288700711 | 0.17875499 | Purifying selection |
| SETIT_036218mg | Os03t0285800-02 | 0.030109412 | 0.388554024 | 0.077490928 | Purifying selection |
| SETIT_034839mg | Os03t0295600-01 | 0.063971594 | 0.549269817 | 0.116466612 | Purifying selection |
| SETIT_034335mg | Os07t0119000-01 | 0.26531468 | 1.055161959 | 0.251444509 | Purifying selection |

Note: The data in the table were analyzed and calculated using TBtools software and MCScanX toolkit; the gene information was obtained from the genome-wide databases of foxtail millet (*Setaria italica*) and *Oryza sativa.*
