# Supplementary material for: Identification and expression analysis of MAPK cascade gene family in foxtail millet (Setaria italica)
Source: Plant Signal Behav. 2023 Aug 16;18(1):2246228. doi: 10.1080/15592324.2023.2246228 (PMC10435010; doi:10.1080/15592324.2023.2246228)
Supplement: Supplemental Material [file KPSB_A_2246228_SM9810.zip › Table S6.docx]

Table S6 The orthologous relationship between *Setaria italica* and *Brachypodium distachyon* and the type of selection pressure

| Gene ID1 | Gene ID2 | Ka | Ks | Ka/Ks | Selection pressure |
| --- | --- | --- | --- | --- | --- |
| SETIT_028893mg | BRADI_1g60340v3 | 0.353010116 | 0.986764737 | 0.357744965 | Purifying selection |
| SETIT_029029mg | BRADI_1g58810v3 | 0.138570144 | 0.483675585 | 0.286493981 | Purifying selection |
| SETIT_032842mg | BRADI_1g23320v3 | 0.058305025 | 0.333983262 | 0.174574691 | Purifying selection |
| SETIT_029157mg | BRADI_1g23970v3 | 0.088321314 | 0.617190443 | 0.143102206 | Purifying selection |
| SETIT_029371mg | BRADI_1g55245v3 | 0.270107827 | 0.572181556 | 0.472066644 | Purifying selection |
| SETIT_029825mg | BRADI_1g20390v3 | 0.045242617 | 0.364295836 | 0.124191968 | Purifying selection |
| SETIT_029452mg | BRADI_1g28110v3 | 0.094530004 | 0.509406054 | 0.185569062 | Purifying selection |
| SETIT_029131mg | BRADI_3g36080v3 | 0.188813941 | 1.020921901 | 0.18494455 | Purifying selection |
| SETIT_029131mg | BRADI_4g29500v3 | 0.079594614 | 0.577484085 | 0.137829971 | Purifying selection |
| SETIT_029013mg | BRADI_4g38400v3 | 0.077287733 | 0.50875704 | 0.151914818 | Purifying selection |
| SETIT_013899mg | BRADI_3g16560v3 | 0.044043965 | 0.726397463 | 0.060633423 | Purifying selection |
| SETIT_014949mg | BRADI_3g36080v3 | 0.185661726 | 0.674888045 | 0.275100037 | Purifying selection |
| SETIT_015231mg | BRADI_3g18150v3 | 0.048809729 | 0.764657824 | 0.063832118 | Purifying selection |
| SETIT_014949mg | BRADI_4g29500v3 | 0.220344206 | 0.975208321 | 0.225945781 | Purifying selection |
| SETIT_000788mg | BRADI_2g45870v3 | 0.055874295 | 0.602826073 | 0.092687257 | Purifying selection |
| SETIT_004003mg | BRADI_2g47480v3 | 0.19217691 | 0.498274737 | 0.385684634 | Purifying selection |
| SETIT_001285mg | BRADI_2g49790v3 | 0.084986585 | 0.475325179 | 0.178796724 | Purifying selection |
| SETIT_003916mg | BRADI_2g57470v3 | 0.152260492 | 0.44808374 | 0.339803653 | Purifying selection |
| SETIT_000725mg | BRADI_2g44350v3 | 0.040936849 | 0.607943886 | 0.067336558 | Purifying selection |
| SETIT_004793mg | BRADI_2g45006v3 | 0.06905803 | 0.507099629 | 0.136182372 | Purifying selection |
| SETIT_001885mg | BRADI_2g00670v3 | 0.054569516 | 0.463468994 | 0.117741459 | Purifying selection |
| SETIT_000788mg | BRADI_2g16337v3 | 0.099827543 | 1.001075536 | 0.099720291 | Purifying selection |
| SETIT_004003mg | BRADI_2g17820v3 | 0.351426382 | 0.847275109 | 0.414772461 | Purifying selection |
| SETIT_001833mg | BRADI_2g15560v3 | 0.091148957 | 0.652223613 | 0.139751084 | Purifying selection |
| SETIT_004793mg | BRADI_2g15620v3 | 0.096077745 | 1.81867204 | 0.052828516 | Purifying selection |
| SETIT_000725mg | BRADI_2g15317v3 | 0.146650349 | 0.883855951 | 0.165921096 | Purifying selection |
| SETIT_000725mg | BRADI_2g45870v3 | 0.205059647 | 1.910514894 | 0.107332137 | Purifying selection |
| SETIT_009480mg | BRADI_3g45790v3 | 0.290460968 | 0.900623991 | 0.322510804 | Purifying selection |
| SETIT_010212mg | BRADI_3g18150v3 | 0.06751058 | 1.017574974 | 0.066344576 | Purifying selection |
| SETIT_009321mg | BRADI_3g51380v3 | 0.162517465 | 0.895846939 | 0.181412089 | Purifying selection |
| SETIT_009795mg | BRADI_4g41870v3 | 0.081747678 | 0.469195414 | 0.17422949 | Purifying selection |
| SETIT_011766mg | BRADI_4g41940v3 | 0.173851211 | 0.752191356 | 0.231126307 | Purifying selection |
| SETIT_009795mg | BRADI_4g24840v3 | 0.277490994 | 0.72113937 | 0.384795236 | Purifying selection |
| SETIT_009321mg | BRADI_5g18180v3 | 0.085453956 | 0.510878242 | 0.167268732 | Purifying selection |
| SETIT_009672mg | BRADI_5g21330v3 | 0.098334989 | 0.624433103 | 0.157478821 | Purifying selection |
| SETIT_009480mg | BRADI_5g10670v3 | 0.11647385 | 0.672302946 | 0.173246079 | Purifying selection |
| SETIT_021565mg | BRADI_2g15620v3 | 0.08412821 | 0.486066345 | 0.173079685 | Purifying selection |
| SETIT_021980mg | BRADI_2g17820v3 | 0.297692676 | 0.799879525 | 0.372171891 | Purifying selection |
| SETIT_021565mg | BRADI_2g45006v3 | 0.120149513 | 1.362217288 | 0.08820143 | Purifying selection |
| SETIT_021980mg | BRADI_2g47480v3 | 0.298792284 | 0.873064734 | 0.342233826 | Purifying selection |
| SETIT_021438mg | BRADI_2g39347v3 | 0.107678015 | 0.717639283 | 0.150044761 | Purifying selection |
| SETIT_021560mg | BRADI_2g15317v3 | 0.238063338 | 1.952248924 | 0.121943127 | Purifying selection |
| SETIT_021645mg | BRADI_2g36470v3 | 0.067522775 | 0.549817945 | 0.122809333 | Purifying selection |
| SETIT_021560mg | BRADI_2g16337v3 | 0.098229767 | 0.594156065 | 0.165326541 | Purifying selection |
| SETIT_024868mg | BRADI_3g13050v3 | 0.334890567 | 1.115386613 | 0.300246177 | Purifying selection |
| SETIT_021297mg | BRADI_4g09990v3 | 0.261373713 | 0.940910318 | 0.277788125 | Purifying selection |
| SETIT_021033mg | BRADI_4g02900v3 | 0.186432798 | 0.531433315 | 0.350811272 | Purifying selection |
| SETIT_021297mg | BRADI_4g04470v3 | 0.100312309 | 0.56420545 | 0.177793939 | Purifying selection |
| SETIT_024868mg | BRADI_5g26917v3 | 0.19457558 | 0.572086194 | 0.340115846 | Purifying selection |
| SETIT_016157mg | BRADI_1g45040v3 | 0.196618497 | 0.897719105 | 0.219020065 | Purifying selection |
| SETIT_017572mg | BRADI_1g46880v3 | 0.11902543 | 0.417001029 | 0.285431982 | Purifying selection |
| SETIT_017554mg | BRADI_1g34030v3 | 0.051060179 | 0.944602205 | 0.054054689 | Purifying selection |
| SETIT_016957mg | BRADI_1g34700v3 | 0.166884157 | 0.881993081 | 0.189212547 | Purifying selection |
| SETIT_016544mg | BRADI_3g45790v3 | 0.234887474 | 0.658123544 | 0.356904834 | Purifying selection |
| SETIT_017053mg | BRADI_3g48360v3 | 0.091732612 | 0.447886175 | 0.204812332 | Purifying selection |
| SETIT_017554mg | BRADI_3g03780v3 | 0.017918431 | 0.534279126 | 0.033537585 | Purifying selection |
| SETIT_017293mg | BRADI_3g05520v3 | 0.045929992 | 0.580963998 | 0.079058242 | Purifying selection |
| SETIT_016157mg | BRADI_3g59510v3 | 0.113590298 | 0.525488385 | 0.216161386 | Purifying selection |
| SETIT_016359mg | BRADI_3g09170v3 | 0.092766513 | 0.572329884 | 0.16208574 | Purifying selection |
| SETIT_016275mg | BRADI_3g51380v3 | 0.093895881 | 0.440760962 | 0.213031302 | Purifying selection |
| SETIT_020144mg | BRADI_3g10887v3 | 0.171309498 | 0.539342 | 0.317626845 | Purifying selection |
| SETIT_016275mg | BRADI_5g18180v3 | 0.126461981 | 0.754563014 | 0.167596316 | Purifying selection |
| SETIT_016544mg | BRADI_5g10670v3 | 0.264763332 | 0.957652225 | 0.276471275 | Purifying selection |
| SETIT_026073mg | BRADI_4g22760v3 | 0.113274372 | 0.502351222 | 0.225488398 | Purifying selection |
| SETIT_026192mg | BRADI_4g41870v3 | 0.157661722 | 0.639848712 | 0.246404687 | Purifying selection |
| SETIT_027310mg | BRADI_4g41940v3 | 0.277205457 | 1.084074973 | 0.255706906 | Purifying selection |
| SETIT_027523mg | BRADI_4g09990v3 | 0.171105384 | 0.542180173 | 0.315587682 | Purifying selection |
| SETIT_026192mg | BRADI_4g24830v3 | 0.286952804 | 0.487547696 | 0.588563552 | Purifying selection |
| SETIT_008355mg | BRADI_1g35350v3 | 0.037531884 | 0.323541595 | 0.116003274 | Purifying selection |
| SETIT_006813mg | BRADI_1g46880v3 | 0.076481413 | 0.419460879 | 0.182332649 | Purifying selection |
| SETIT_006611mg | BRADI_1g49100v3 | 0.018093148 | 0.557966321 | 0.032426954 | Purifying selection |
| SETIT_006144mg | BRADI_1g34700v3 | 0.068516926 | 0.524694585 | 0.130584397 | Purifying selection |
| SETIT_005743mg | BRADI_1g45040v3 | 0.116584386 | 0.494242293 | 0.235885087 | Purifying selection |
| SETIT_005733mg | BRADI_1g30720v3 | 0.112608431 | 0.524789091 | 0.214578452 | Purifying selection |
| SETIT_006708mg | BRADI_1g34030v3 | 0.029483391 | 0.523071324 | 0.05636591 | Purifying selection |
| SETIT_005743mg | BRADI_3g59510v3 | 0.189808573 | 0.908208571 | 0.208992272 | Purifying selection |
| SETIT_006708mg | BRADI_3g03780v3 | 0.040005443 | 0.848477744 | 0.047149666 | Purifying selection |
| SETIT_006813mg | BRADI_3g53650v3 | 0.070152454 | 0.416171315 | 0.168566289 | Purifying selection |
| SETIT_036560mg | BRADI_1g69400v3 | 0.059143931 | 0.35816013 | 0.16513265 | Purifying selection |
| SETIT_034063mg | BRADI_1g74480v3 | 0.095074431 | 0.47859863 | 0.198651699 | Purifying selection |
| SETIT_034839mg | BRADI_1g65300v3 | 0.065356495 | 0.529788236 | 0.123363433 | Purifying selection |
| SETIT_036218mg | BRADI_1g65810v3 | 0.035869788 | 0.386094023 | 0.092904282 | Purifying selection |
| SETIT_034335mg | BRADI_1g07650v3 | 0.131391901 | 0.60816693 | 0.216045783 | Purifying selection |
| SETIT_035970mg | BRADI_1g08865v3 | 0.159238228 | 0.446403751 | 0.356713463 | Purifying selection |
| SETIT_036191mg | BRADI_1g14000v3 | 0.037479146 | 0.553227805 | 0.067746316 | Purifying selection |
| SETIT_035834mg | BRADI_1g04080v3 | 0.050967473 | 0.525492452 | 0.096989923 | Purifying selection |
| SETIT_034802mg | BRADI_1g67397v3 | 0.1258437 | 0.541075103 | 0.232580836 | Purifying selection |
| SETIT_039120mg | BRADI_1g10970v3 | 0.078650108 | 0.593522587 | 0.132514094 | Purifying selection |
| SETIT_034087mg | BRADI_1g74480v3 | 0.233648957 | 0.848471202 | 0.275376414 | Purifying selection |
| SETIT_039120mg | BRADI_1g67397v3 | 0.33984052 | 1.704582195 | 0.199368808 | Purifying selection |
| SETIT_034063mg | BRADI_3g27120v3 | 0.243009217 | 1.045342603 | 0.23246849 | Purifying selection |
| SETIT_036240mg | BRADI_3g32000v3 | 0.028391572 | 0.483279743 | 0.058747697 | Purifying selection |
| SETIT_034087mg | BRADI_3g27120v3 | 0.142513649 | 0.528212578 | 0.269803588 | Purifying selection |
| SETIT_036191mg | BRADI_4g02367v3 | 0.235607147 | 0.988414147 | 0.238368854 | Purifying selection |

Note: The data in the table were analyzed and calculated using TBtools software and MCScanX toolkit; the gene information was obtained from the genome-wide databases of foxtail millet (*Setaria italica*) and *Brachypodium distachyon.*
