# Supplementary material for: Identification and expression analysis of MAPK cascade gene family in foxtail millet (Setaria italica)
Source: Plant Signal Behav. 2023 Aug 16;18(1):2246228. doi: 10.1080/15592324.2023.2246228 (PMC10435010; doi:10.1080/15592324.2023.2246228)
Supplement: Supplemental Material [file KPSB_A_2246228_SM9810.zip › Table S7.docx]

| Table S7 Information of cis-elements in the promoter region of SiMAPK cascade gene family members | | | | | | |
| --- | --- | --- | --- | --- | --- | --- |
| Gene name | Gene ID | Cis-element | Sequence | Start | End | Function |
| SiMAPK3 | SETIT_036218mg | MSA-like | (T/C)C(T/C)AACGG(T/C)(T/C)A | 1187 | 1205 | cell cycle control element |
| SiMAPK3 | SETIT_036218mg | TCA-element | TCAGAAGAGG | 1837 | 1855 | salicylic acid response element |
| SiMAPK3 | SETIT_036218mg | ABRE | ACGTG | 891 | 901 | abscisic acid response element |
| SiMAPK3 | SETIT_036218mg | ABRE | ACGTG | 1089 | 1099 | abscisic acid response element |
| SiMAPK3 | SETIT_036218mg | ABRE | CACGTG | 1649 | 1661 | abscisic acid response element |
| SiMAPK3 | SETIT_036218mg | ABRE | ACGTG | 1651 | 1661 | abscisic acid response element |
| SiMAPK3 | SETIT_036218mg | A-box | CCGTCC | 549 | 561 | cis-acting regulatory element |
| SiMAPK3 | SETIT_036218mg | A-box | CCGTCC | 1356 | 1368 | cis-acting regulatory element |
| SiMAPK3 | SETIT_036218mg | G-Box | CACGTT | 889 | 901 | light responsive element |
| SiMAPK3 | SETIT_036218mg | G-Box | CACGTG | 1649 | 1661 | light responsive element |
| SiMAPK3 | SETIT_036218mg | G-box | CACGAC | 947 | 959 | light responsive element |
| SiMAPK3 | SETIT_036218mg | G-box | CACGTC | 1088 | 1100 | light responsive element |
| SiMAPK3 | SETIT_036218mg | G-box | ACACGTGT | 1646 | 1662 | light responsive element |
| SiMAPK3 | SETIT_036218mg | G-box | CACGTG | 1649 | 1661 | light responsive element |
| SiMAPK3 | SETIT_036218mg | CGTCA-motif | CGTCA | 1238 | 1248 | MeJA response regulatory element |
| SiMAPK3 | SETIT_036218mg | CGTCA-motif | CGTCA | 1634 | 1644 | MeJA response regulatory element |
| SiMAPK3 | SETIT_036218mg | TGACG-motif | TGACG | 1238 | 1248 | MeJA response regulatory element |
| SiMAPK3 | SETIT_036218mg | TGACG-motif | TGACG | 1634 | 1644 | MeJA response regulatory element |
| SiMAPK3 | SETIT_036218mg | O2-site | GATGATGTGG | 1169 | 1187 | Zein metabolism regulatory elements |
| SiMAPK3 | SETIT_036218mg | CAAT-box | CAAAT | 83 | 93 | common cis-acting element |
| SiMAPK3 | SETIT_036218mg | CAAT-box | CCAAT | 198 | 208 | common cis-acting element |
| SiMAPK3 | SETIT_036218mg | CAAT-box | CAAAT | 359 | 369 | common cis-acting element |
| SiMAPK3 | SETIT_036218mg | CAAT-box | CCAAT | 478 | 488 | common cis-acting element |
| SiMAPK3 | SETIT_036218mg | CAAT-box | CCAAT | 493 | 503 | common cis-acting element |
| SiMAPK3 | SETIT_036218mg | CAAT-box | CCAAT | 505 | 515 | common cis-acting element |
| SiMAPK3 | SETIT_036218mg | CAAT-box | CAAAT | 527 | 537 | common cis-acting element |
| SiMAPK3 | SETIT_036218mg | CAAT-box | CAAAT | 1567 | 1577 | common cis-acting element |
| SiMAPK3 | SETIT_036218mg | CAAT-box | CAAAT | 1851 | 1861 | common cis-acting element |
| SiMAPK3 | SETIT_036218mg | CAAT-box | CAAAT | 1900 | 1910 | common cis-acting element |
| SiMAPK3 | SETIT_036218mg | TATA-box | TATAA | 247 | 257 | core promoter element |
| SiMAPK3 | SETIT_036218mg | TATA-box | TATA | 249 | 257 | core promoter element |
| SiMAPK3 | SETIT_036218mg | TATA-box | TATACA | 251 | 263 | core promoter element |
| SiMAPK3 | SETIT_036218mg | TATA-box | TATA | 255 | 263 | core promoter element |
| SiMAPK3 | SETIT_036218mg | TATA-box | TATA | 405 | 413 | core promoter element |
| SiMAPK3 | SETIT_036218mg | TATA-box | TACAAAA | 800 | 814 | core promoter element |
| SiMAPK3 | SETIT_036218mg | TATA-box | TATA | 1694 | 1702 | core promoter element |
| SiMAPK3 | SETIT_036218mg | TATA-box | TATAAAA | 1814 | 1828 | core promoter element |
| SiMAPK3 | SETIT_036218mg | TATA-box | taTATAAAtc | 1813 | 1831 | core promoter element |
| SiMAPK3 | SETIT_036218mg | TATA-box | TATAA | 1818 | 1828 | core promoter element |
| SiMAPK3 | SETIT_036218mg | TATA-box | TATA | 1820 | 1828 | core promoter element |
| SiMAPK3 | SETIT_036218mg | GC-motif | CCCCCG | 1401 | 1413 | hypoxia-specifically induced elements |
| SiMAPK3 | SETIT_036218mg | GC-motif | CCCCCG | 1753 | 1765 | hypoxia-specifically induced elements |
| SiMAPK3 | SETIT_036218mg | Sp1 | GGGCGG | 1181 | 1193 | light responsive element |
| SiMAPK3 | SETIT_036218mg | Sp1 | GGGCGG | 1318 | 1330 | light responsive element |
| SiMAPK3 | SETIT_036218mg | Sp1 | GGGCGG | 1532 | 1544 | light responsive element |
| SiMAPK3 | SETIT_036218mg | Sp1 | GGGCGG | 1805 | 1817 | light responsive element |
| SiMAPK3 | SETIT_036218mg | CCAAT-box | CAACGG | 1192 | 1204 | MYBHv1 binding site |
| SiMAPK3 | SETIT_036218mg | CCAAT-box | CAACGG | 1305 | 1317 | MYBHv1 binding site |
| SiMAPK3 | SETIT_036218mg | ATCT-motif | AATCTAATCC | 753 | 771 | light responsive element |
| SiMAPK3 | SETIT_036218mg | Box 4 | ATTAAT | 340 | 352 | light responsive element |
| SiMAPK3 | SETIT_036218mg | I-box | gGATAAGGTG | 249 | 267 | light responsive element |
| SiMAPK3 | SETIT_036218mg | I-box | AAGATAAGGCT | 282 | 302 | light responsive element |
| SiMAPK3 | SETIT_036218mg | GATA-motif | AAGATAAGATT | 282 | 302 | light responsive element |
| SiMAPK3 | SETIT_036218mg | GATA-motif | GATAGGG | 737 | 751 | light responsive element |
| SiMAPK3 | SETIT_036218mg | Box III | atCATTTTCACt | 475 | 497 | protein binding site |
| SiMAPK4 | SETIT_036240mg | TGA-element | AACGAC | 393 | 405 | auxin-responsive element |
| SiMAPK4 | SETIT_036240mg | TGA-element | AACGAC | 1299 | 1311 | auxin-responsive element |
| SiMAPK4 | SETIT_036240mg | TC-rich repeats | GTTTTCTTAC | 3 | 21 | defense and stress response elements |
| SiMAPK4 | SETIT_036240mg | ACE | CTAACGTATT | 799 | 817 | light responsive element |
| SiMAPK4 | SETIT_036240mg | LTR | CCGAAA | 158 | 170 | low temperature response element |
| SiMAPK4 | SETIT_036240mg | LTR | CCGAAA | 354 | 366 | low temperature response element |
| SiMAPK4 | SETIT_036240mg | ABRE | ACGTG | 1028 | 1038 | abscisic acid response element |
| SiMAPK4 | SETIT_036240mg | ABRE | CACGTG | 1363 | 1375 | abscisic acid response element |
| SiMAPK4 | SETIT_036240mg | ABRE | ACGTG | 1365 | 1375 | abscisic acid response element |
| SiMAPK4 | SETIT_036240mg | ABRE | ACGTG | 1399 | 1409 | abscisic acid response element |
| SiMAPK4 | SETIT_036240mg | ABRE | TACGGTC | 1416 | 1430 | abscisic acid response element |
| SiMAPK4 | SETIT_036240mg | ARE | AAACCA | 642 | 654 | anaerobic inducing element |
| SiMAPK4 | SETIT_036240mg | ARE | AAACCA | 919 | 931 | anaerobic inducing element |
| SiMAPK4 | SETIT_036240mg | G-box | CACGAC | 1147 | 1159 | light responsive element |
| SiMAPK4 | SETIT_036240mg | G-box | CACGTG | 1363 | 1375 | light responsive element |
| SiMAPK4 | SETIT_036240mg | G-box | TACGTG | 1398 | 1410 | light responsive element |
| SiMAPK4 | SETIT_036240mg | G-Box | CACGTT | 1026 | 1038 | light responsive element |
| SiMAPK4 | SETIT_036240mg | G-Box | CACGTGAAA | 1357 | 1375 | light responsive element |
| SiMAPK4 | SETIT_036240mg | G-Box | CACGTG | 1363 | 1375 | light responsive element |
| SiMAPK4 | SETIT_036240mg | CGTCA-motif | CGTCA | 1201 | 1211 | MeJA response regulatory element |
| SiMAPK4 | SETIT_036240mg | TGACG-motif | TGACG | 1201 | 1211 | MeJA response regulatory element |
| SiMAPK4 | SETIT_036240mg | CAT-box | GCCACT | 944 | 956 | meristem expression regulatory element |
| SiMAPK4 | SETIT_036240mg | CAAT-box | CAAAT | 29 | 39 | common cis-acting element |
| SiMAPK4 | SETIT_036240mg | CAAT-box | CCAAT | 322 | 332 | common cis-acting element |
| SiMAPK4 | SETIT_036240mg | CAAT-box | CAAAT | 476 | 486 | common cis-acting element |
| SiMAPK4 | SETIT_036240mg | CAAT-box | CAAAT | 508 | 518 | common cis-acting element |
| SiMAPK4 | SETIT_036240mg | CAAT-box | CAAAT | 705 | 715 | common cis-acting element |
| SiMAPK4 | SETIT_036240mg | CAAT-box | CAAAT | 741 | 751 | common cis-acting element |
| SiMAPK4 | SETIT_036240mg | CAAT-box | CAACCAACTCC | 1049 | 1069 | common cis-acting element |
| SiMAPK4 | SETIT_036240mg | CAAT-box | TGCCAAC | 1102 | 1116 | common cis-acting element |
| SiMAPK4 | SETIT_036240mg | CAAT-box | CCAAT | 1394 | 1404 | common cis-acting element |
| SiMAPK4 | SETIT_036240mg | CAAT-box | CAAAT | 1462 | 1472 | common cis-acting element |
| SiMAPK4 | SETIT_036240mg | CAAT-box | CCAAT | 1605 | 1615 | common cis-acting element |
| SiMAPK4 | SETIT_036240mg | CAAT-box | CCAAT | 1907 | 1917 | common cis-acting element |
| SiMAPK4 | SETIT_036240mg | TATA-box | TATATAA | 53 | 67 | core promoter element |
| SiMAPK4 | SETIT_036240mg | TATA-box | TATATA | 55 | 67 | core promoter element |
| SiMAPK4 | SETIT_036240mg | TATA-box | ATATAA | 56 | 68 | core promoter element |
| SiMAPK4 | SETIT_036240mg | TATA-box | TATA | 59 | 67 | core promoter element |
| SiMAPK4 | SETIT_036240mg | TATA-box | TATA | 554 | 562 | core promoter element |
| SiMAPK4 | SETIT_036240mg | TATA-box | TATATAA | 588 | 602 | core promoter element |
| SiMAPK4 | SETIT_036240mg | TATA-box | TATATA | 590 | 602 | core promoter element |
| SiMAPK4 | SETIT_036240mg | TATA-box | TATA | 594 | 602 | core promoter element |
| SiMAPK4 | SETIT_036240mg | TATA-box | TATATAA | 1340 | 1354 | core promoter element |
| SiMAPK4 | SETIT_036240mg | TATA-box | TATATA | 1342 | 1354 | core promoter element |
| SiMAPK4 | SETIT_036240mg | TATA-box | ATATAT | 1343 | 1355 | core promoter element |
| SiMAPK4 | SETIT_036240mg | TATA-box | TATA | 1346 | 1354 | core promoter element |
| SiMAPK4 | SETIT_036240mg | TATA-box | ccTATAAAaa | 1529 | 1547 | core promoter element |
| SiMAPK4 | SETIT_036240mg | TATA-box | TATA | 1536 | 1544 | core promoter element |
| SiMAPK4 | SETIT_036240mg | TATA-box | TATA | 1596 | 1604 | core promoter element |
| SiMAPK4 | SETIT_036240mg | GC-motif | CCCCCG | 1707 | 1719 | hypoxia-specifically induced elements |
| SiMAPK4 | SETIT_036240mg | P-box | CCTTTTG | 1063 | 1077 | gibberellin-responsive element |
| SiMAPK4 | SETIT_036240mg | Sp1 | GGGCGG | 1567 | 1579 | light responsive element |
| SiMAPK4 | SETIT_036240mg | Sp1 | GGGCGG | 1744 | 1756 | light responsive element |
| SiMAPK4 | SETIT_036240mg | Sp1 | GGGCGG | 1748 | 1760 | light responsive element |
| SiMAPK4 | SETIT_036240mg | Sp1 | GGGCGG | 1826 | 1838 | light responsive element |
| SiMAPK4 | SETIT_036240mg | 3-AF3 binding site | CACTATCTAAC | 409 | 429 | CMA3 |
| SiMAPK4 | SETIT_036240mg | ATC-motif | AGTAATCT | 532 | 548 | light responsive element |
| SiMAPK4 | SETIT_036240mg | ATCT-motif | AATCTAATCC | 611 | 629 | light responsive element |
| SiMAPK4 | SETIT_036240mg | I-box | atGATAAGGTC | 1228 | 1248 | light responsive element |
| SiMAPK4 | SETIT_036240mg | TCT-motif | TCTTAC | 413 | 425 | light responsive element |
| SiMAPK4 | SETIT_036240mg | TCT-motif | TCTTAC | 1041 | 1053 | light responsive element |
| SiMAPK4 | SETIT_036240mg | AE-box | AGAAACTT | 857 | 873 | light responsive element |
| SiMAPK6 | SETIT_006611mg | TCA-element | CCATCTTTTT | 1881 | 1899 | salicylic acid response element |
| SiMAPK6 | SETIT_006611mg | ABRE | ACGTG | 249 | 259 | abscisic acid response element |
| SiMAPK6 | SETIT_006611mg | ABRE | CACGTG | 679 | 691 | abscisic acid response element |
| SiMAPK6 | SETIT_006611mg | ABRE | ACGTG | 681 | 691 | abscisic acid response element |
| SiMAPK6 | SETIT_006611mg | ABRE | GCCGCGTGGC | 1699 | 1717 | abscisic acid response element |
| SiMAPK6 | SETIT_006611mg | ABRE | CACGTG | 1704 | 1716 | abscisic acid response element |
| SiMAPK6 | SETIT_006611mg | ABRE | ACGTG | 1706 | 1716 | abscisic acid response element |
| SiMAPK6 | SETIT_006611mg | ARE | AAACCA | 61 | 73 | anaerobic inducing element |
| SiMAPK6 | SETIT_006611mg | ARE | AAACCA | 619 | 631 | anaerobic inducing element |
| SiMAPK6 | SETIT_006611mg | ARE | AAACCA | 1833 | 1845 | anaerobic inducing element |
| SiMAPK6 | SETIT_006611mg | G-Box | CACGTG | 679 | 691 | light responsive element |
| SiMAPK6 | SETIT_006611mg | G-Box | CACGTG | 1704 | 1716 | light responsive element |
| SiMAPK6 | SETIT_006611mg | G-box | TACGTG | 248 | 260 | light responsive element |
| SiMAPK6 | SETIT_006611mg | G-box | CACGTG | 679 | 691 | light responsive element |
| SiMAPK6 | SETIT_006611mg | G-box | tgACACGTGGCA | 1696 | 1718 | light responsive element |
| SiMAPK6 | SETIT_006611mg | G-box | GCCACGTGGA | 1699 | 1717 | light responsive element |
| SiMAPK6 | SETIT_006611mg | G-box | CACGTG | 1704 | 1716 | light responsive element |
| SiMAPK6 | SETIT_006611mg | RY-element | CATGCATG | 339 | 355 | elements involved in seed-specific regulation |
| SiMAPK6 | SETIT_006611mg | TGACG-motif | TGACG | 91 | 101 | MeJA response regulatory element |
| SiMAPK6 | SETIT_006611mg | CGTCA-motif | CGTCA | 91 | 101 | MeJA response regulatory element |
| SiMAPK6 | SETIT_006611mg | CAT-box | GCCACT | 1413 | 1425 | meristem expression regulatory element |
| SiMAPK6 | SETIT_006611mg | CAAT-box | CCAAT | 133 | 143 | common cis-acting element |
| SiMAPK6 | SETIT_006611mg | CAAT-box | CAAAT | 370 | 380 | common cis-acting element |
| SiMAPK6 | SETIT_006611mg | CAAT-box | CAAAT | 455 | 465 | common cis-acting element |
| SiMAPK6 | SETIT_006611mg | CAAT-box | CAAAT | 554 | 564 | common cis-acting element |
| SiMAPK6 | SETIT_006611mg | CAAT-box | CAAAT | 775 | 785 | common cis-acting element |
| SiMAPK6 | SETIT_006611mg | CAAT-box | CAAAT | 839 | 849 | common cis-acting element |
| SiMAPK6 | SETIT_006611mg | CAAT-box | CAAAT | 922 | 932 | common cis-acting element |
| SiMAPK6 | SETIT_006611mg | CAAT-box | CCAAT | 1014 | 1024 | common cis-acting element |
| SiMAPK6 | SETIT_006611mg | CAAT-box | CCAAT | 1047 | 1057 | common cis-acting element |
| SiMAPK6 | SETIT_006611mg | CAAT-box | CCAAT | 1074 | 1084 | common cis-acting element |
| SiMAPK6 | SETIT_006611mg | CAAT-box | CAAAT | 1104 | 1114 | common cis-acting element |
| SiMAPK6 | SETIT_006611mg | CAAT-box | CAAAT | 1369 | 1379 | common cis-acting element |
| SiMAPK6 | SETIT_006611mg | CAAT-box | CAAAT | 1497 | 1507 | common cis-acting element |
| SiMAPK6 | SETIT_006611mg | CAAT-box | CAAAT | 1591 | 1601 | common cis-acting element |
| SiMAPK6 | SETIT_006611mg | CAAT-box | CAAAT | 1985 | 1995 | common cis-acting element |
| SiMAPK6 | SETIT_006611mg | TATA-box | TATA | 434 | 442 | core promoter element |
| SiMAPK6 | SETIT_006611mg | TATA-box | TAAAGATT | 434 | 450 | core promoter element |
| SiMAPK6 | SETIT_006611mg | TATA-box | TATA | 690 | 698 | core promoter element |
| SiMAPK6 | SETIT_006611mg | TATA-box | TACAAAA | 821 | 835 | core promoter element |
| SiMAPK6 | SETIT_006611mg | TATA-box | ATTATA | 852 | 864 | core promoter element |
| SiMAPK6 | SETIT_006611mg | TATA-box | TATAA | 854 | 864 | core promoter element |
| SiMAPK6 | SETIT_006611mg | TATA-box | TATA | 856 | 864 | core promoter element |
| SiMAPK6 | SETIT_006611mg | TATA-box | ATTATA | 976 | 988 | core promoter element |
| SiMAPK6 | SETIT_006611mg | TATA-box | TATAA | 978 | 988 | core promoter element |
| SiMAPK6 | SETIT_006611mg | TATA-box | TATA | 980 | 988 | core promoter element |
| SiMAPK6 | SETIT_006611mg | TATA-box | TATA | 1297 | 1305 | core promoter element |
| SiMAPK6 | SETIT_006611mg | TATA-box | TATA | 1304 | 1312 | core promoter element |
| SiMAPK6 | SETIT_006611mg | TATA-box | ATATAT | 1345 | 1357 | core promoter element |
| SiMAPK6 | SETIT_006611mg | TATA-box | TATA | 1348 | 1356 | core promoter element |
| SiMAPK6 | SETIT_006611mg | TATA-box | ATTATA | 1375 | 1387 | core promoter element |
| SiMAPK6 | SETIT_006611mg | TATA-box | TATAA | 1377 | 1387 | core promoter element |
| SiMAPK6 | SETIT_006611mg | TATA-box | TATA | 1379 | 1387 | core promoter element |
| SiMAPK6 | SETIT_006611mg | TATA-box | ATTATA | 1431 | 1443 | core promoter element |
| SiMAPK6 | SETIT_006611mg | TATA-box | TATAA | 1433 | 1443 | core promoter element |
| SiMAPK6 | SETIT_006611mg | TATA-box | TATA | 1435 | 1443 | core promoter element |
| SiMAPK6 | SETIT_006611mg | TATA-box | TATAAGAA | 1464 | 1480 | core promoter element |
| SiMAPK6 | SETIT_006611mg | TATA-box | TATAA | 1470 | 1480 | core promoter element |
| SiMAPK6 | SETIT_006611mg | TATA-box | TATA | 1472 | 1480 | core promoter element |
| SiMAPK6 | SETIT_006611mg | TATA-box | ATTATA | 1554 | 1566 | core promoter element |
| SiMAPK6 | SETIT_006611mg | TATA-box | TATAA | 1556 | 1566 | core promoter element |
| SiMAPK6 | SETIT_006611mg | TATA-box | TATA | 1558 | 1566 | core promoter element |
| SiMAPK6 | SETIT_006611mg | Sp1 | GGGCGG | 1961 | 1973 | light responsive element |
| SiMAPK6 | SETIT_006611mg | MBS | CAACTG | 175 | 187 | MYB binding site involved in drought-inducibility |
| SiMAPK6 | SETIT_006611mg | Box II | CCACGTGGC | 1699 | 1717 | light responsive element |
| SiMAPK6 | SETIT_006611mg | Box II | CCACGTGGC | 1700 | 1718 | light responsive element |
| SiMAPK6 | SETIT_006611mg | chs-CMA2a | TCACTTGA | 602 | 618 | light responsive element |
| SiMAPK6 | SETIT_006611mg | I-box | TGATAATGT | 825 | 843 | light responsive element |
| SiMAPK6 | SETIT_006611mg | AE-box | AGAAACAA | 793 | 809 | light responsive element |
| SiMAPK7 | SETIT_006708mg | TGA-element | AACGAC | 1736 | 1748 | auxin-responsive element |
| SiMAPK7 | SETIT_006708mg | TCA-element | CCATCTTTTT | 1928 | 1946 | salicylic acid response element |
| SiMAPK7 | SETIT_006708mg | ABRE | CGCACGTGTC | 26 | 44 | abscisic acid response element |
| SiMAPK7 | SETIT_006708mg | ABRE | CACGTG | 31 | 43 | abscisic acid response element |
| SiMAPK7 | SETIT_006708mg | ABRE | ACGTG | 33 | 43 | abscisic acid response element |
| SiMAPK7 | SETIT_006708mg | ABRE | AACCCGG | 225 | 239 | abscisic acid response element |
| SiMAPK7 | SETIT_006708mg | ABRE | ACGTG | 290 | 300 | abscisic acid response element |
| SiMAPK7 | SETIT_006708mg | A-box | CCGTCC | 177 | 189 | cis-acting regulatory element |
| SiMAPK7 | SETIT_006708mg | A-box | CCGTCC | 887 | 899 | cis-acting regulatory element |
| SiMAPK7 | SETIT_006708mg | ARE | AAACCA | 1638 | 1650 | anaerobic inducing element |
| SiMAPK7 | SETIT_006708mg | G-box | CACGTG | 31 | 43 | light responsive element |
| SiMAPK7 | SETIT_006708mg | G-box | CACGTC | 289 | 301 | light responsive element |
| SiMAPK7 | SETIT_006708mg | G-box | CACGAC | 307 | 319 | light responsive element |
| SiMAPK7 | SETIT_006708mg | G-box | ACACGTG(G/t)CACC | 708.5 | 729.5 | light responsive element |
| SiMAPK7 | SETIT_006708mg | G-Box | CACGTG | 31 | 43 | light responsive element |
| SiMAPK7 | SETIT_006708mg | CGTCA-motif | CGTCA | 287 | 297 | MeJA response regulatory element |
| SiMAPK7 | SETIT_006708mg | CGTCA-motif | CGTCA | 298 | 308 | MeJA response regulatory element |
| SiMAPK7 | SETIT_006708mg | CGTCA-motif | CGTCA | 691 | 701 | MeJA response regulatory element |
| SiMAPK7 | SETIT_006708mg | CGTCA-motif | CGTCA | 1789 | 1799 | MeJA response regulatory element |
| SiMAPK7 | SETIT_006708mg | TGACG-motif | TGACG | 287 | 297 | MeJA response regulatory element |
| SiMAPK7 | SETIT_006708mg | TGACG-motif | TGACG | 298 | 308 | MeJA response regulatory element |
| SiMAPK7 | SETIT_006708mg | TGACG-motif | TGACG | 691 | 701 | MeJA response regulatory element |
| SiMAPK7 | SETIT_006708mg | TGACG-motif | TGACG | 1789 | 1799 | MeJA response regulatory element |
| SiMAPK7 | SETIT_006708mg | O2-site | GATGATGTGG | 1314 | 1332 | Zein metabolism regulatory elements |
| SiMAPK7 | SETIT_006708mg | CAAT-box | CAACCAACTCC | 244 | 264 | common cis-acting element |
| SiMAPK7 | SETIT_006708mg | CAAT-box | CAAAT | 1698 | 1708 | common cis-acting element |
| SiMAPK7 | SETIT_006708mg | CAAT-box | CAAAT | 1714 | 1724 | common cis-acting element |
| SiMAPK7 | SETIT_006708mg | GC-motif | CCCCCG | 174 | 186 | hypoxia-specifically induced elements |
| SiMAPK7 | SETIT_006708mg | GC-motif | CCCCCG | 277 | 289 | hypoxia-specifically induced elements |
| SiMAPK7 | SETIT_006708mg | GC-motif | CCCCCG | 906 | 918 | hypoxia-specifically induced elements |
| SiMAPK7 | SETIT_006708mg | P-box | CCTTTTG | 1075 | 1089 | gibberellin-responsive element |
| SiMAPK7 | SETIT_006708mg | Sp1 | GGGCGG | 239 | 251 | light responsive element |
| SiMAPK7 | SETIT_006708mg | Sp1 | GGGCGG | 555 | 567 | light responsive element |
| SiMAPK7 | SETIT_006708mg | Sp1 | GGGCGG | 570 | 582 | light responsive element |
| SiMAPK7 | SETIT_006708mg | Sp1 | GGGCGG | 574 | 586 | light responsive element |
| SiMAPK7 | SETIT_006708mg | Sp1 | GGGCGG | 700 | 712 | light responsive element |
| SiMAPK7 | SETIT_006708mg | Sp1 | GGGCGG | 827 | 839 | light responsive element |
| SiMAPK7 | SETIT_006708mg | Sp1 | GGGCGG | 1099 | 1111 | light responsive element |
| SiMAPK7 | SETIT_006708mg | MBS | CAACTG | 1089 | 1101 | MYB binding site involved in drought-inducibility |
| SiMAPK7 | SETIT_006708mg | MBS | CAACTG | 1423 | 1435 | MYB binding site involved in drought-inducibility |
| SiMAPK7 | SETIT_006708mg | MBS | CAACTG | 1544 | 1556 | MYB binding site involved in drought-inducibility |
| SiMAPK7 | SETIT_006708mg | CCAAT-box | CAACGG | 84 | 96 | MYBHv1 binding site |
| SiMAPK7 | SETIT_006708mg | CCAAT-box | CAACGG | 671 | 683 | MYBHv1 binding site |
| SiMAPK7 | SETIT_006708mg | AE-box | AGAAACTT | 1840 | 1856 | light responsive element |
| SiMAPK11 | SETIT_013899mg | TGA-element | AACGAC | 354 | 366 | auxin-responsive element |
| SiMAPK11 | SETIT_013899mg | TATC-box | TATCCCA | 343 | 357 | gibberellin-responsive element |
| SiMAPK11 | SETIT_013899mg | ABRE | ACGTG | 135 | 145 | abscisic acid response element |
| SiMAPK11 | SETIT_013899mg | ABRE | GCCGCGTGGC | 1058 | 1078 | abscisic acid response element |
| SiMAPK11 | SETIT_013899mg | ABRE | CGTACGTGCA | 1975 | 1993 | abscisic acid response element |
| SiMAPK11 | SETIT_013899mg | ABRE | ACGTG | 1982 | 1992 | abscisic acid response element |
| SiMAPK11 | SETIT_013899mg | A-box | CCGTCC | 1827 | 1839 | cis-acting regulatory element |
| SiMAPK11 | SETIT_013899mg | A-box | CCGTCC | 1841 | 1853 | cis-acting regulatory element |
| SiMAPK11 | SETIT_013899mg | ARE | AAACCA | 260 | 272 | anaerobic inducing element |
| SiMAPK11 | SETIT_013899mg | ARE | AAACCA | 367 | 379 | anaerobic inducing element |
| SiMAPK11 | SETIT_013899mg | ARE | AAACCA | 438 | 450 | anaerobic inducing element |
| SiMAPK11 | SETIT_013899mg | ARE | AAACCA | 1436 | 1448 | anaerobic inducing element |
| SiMAPK11 | SETIT_013899mg | G-box | TACGTG | 134 | 146 | light responsive element |
| SiMAPK11 | SETIT_013899mg | G-box | TACGTG | 1980 | 1992 | light responsive element |
| SiMAPK11 | SETIT_013899mg | RY-element | CATGCATG | 1400 | 1416 | elements involved in seed-specific regulation |
| SiMAPK11 | SETIT_013899mg | TGACG-motif | TGACG | 1692 | 1702 | MeJA response regulatory element |
| SiMAPK11 | SETIT_013899mg | CGTCA-motif | CGTCA | 1692 | 1702 | MeJA response regulatory element |
| SiMAPK11 | SETIT_013899mg | CAAT-box | CAAAT | 83 | 93 | common cis-acting element |
| SiMAPK11 | SETIT_013899mg | CAAT-box | CAAAT | 242 | 252 | common cis-acting element |
| SiMAPK11 | SETIT_013899mg | CAAT-box | CAAAT | 329 | 339 | common cis-acting element |
| SiMAPK11 | SETIT_013899mg | CAAT-box | CCAAT | 442 | 452 | common cis-acting element |
| SiMAPK11 | SETIT_013899mg | CAAT-box | CAAAT | 496 | 506 | common cis-acting element |
| SiMAPK11 | SETIT_013899mg | CAAT-box | CAAAT | 571 | 581 | common cis-acting element |
| SiMAPK11 | SETIT_013899mg | CAAT-box | CAAAT | 655 | 665 | common cis-acting element |
| SiMAPK11 | SETIT_013899mg | CAAT-box | CAAAT | 1146 | 1156 | common cis-acting element |
| SiMAPK11 | SETIT_013899mg | CAAT-box | CAAAT | 1160 | 1170 | common cis-acting element |
| SiMAPK11 | SETIT_013899mg | CAAT-box | CAAAT | 1192 | 1202 | common cis-acting element |
| SiMAPK11 | SETIT_013899mg | CAAT-box | CCAAT | 1245 | 1255 | common cis-acting element |
| SiMAPK11 | SETIT_013899mg | CAAT-box | CAAAT | 1300 | 1310 | common cis-acting element |
| SiMAPK11 | SETIT_013899mg | CAAT-box | CAAAT | 1431 | 1441 | common cis-acting element |
| SiMAPK11 | SETIT_013899mg | CAAT-box | CAACCAACTCC | 1668 | 1688 | common cis-acting element |
| SiMAPK11 | SETIT_013899mg | TATA-box | TATTTAAA | 22 | 38 | core promoter element |
| SiMAPK11 | SETIT_013899mg | TATA-box | ATATAA | 305 | 317 | core promoter element |
| SiMAPK11 | SETIT_013899mg | TATA-box | TATA | 308 | 316 | core promoter element |
| SiMAPK11 | SETIT_013899mg | TATA-box | ATATAT | 334 | 346 | core promoter element |
| SiMAPK11 | SETIT_013899mg | TATA-box | TATA | 337 | 345 | core promoter element |
| SiMAPK11 | SETIT_013899mg | TATA-box | TATAAAA | 469 | 483 | core promoter element |
| SiMAPK11 | SETIT_013899mg | TATA-box | TATAAA | 471 | 483 | core promoter element |
| SiMAPK11 | SETIT_013899mg | TATA-box | TATATAA | 471 | 485 | core promoter element |
| SiMAPK11 | SETIT_013899mg | TATA-box | TATATA | 473 | 485 | core promoter element |
| SiMAPK11 | SETIT_013899mg | TATA-box | ATATAA | 474 | 486 | core promoter element |
| SiMAPK11 | SETIT_013899mg | TATA-box | TATA | 477 | 485 | core promoter element |
| SiMAPK11 | SETIT_013899mg | TATA-box | TATAA | 619 | 629 | core promoter element |
| SiMAPK11 | SETIT_013899mg | TATA-box | TATA | 621 | 629 | core promoter element |
| SiMAPK11 | SETIT_013899mg | TATA-box | ATTATA | 1122 | 1134 | core promoter element |
| SiMAPK11 | SETIT_013899mg | TATA-box | TATAA | 1124 | 1134 | core promoter element |
| SiMAPK11 | SETIT_013899mg | TATA-box | TATA | 1126 | 1134 | core promoter element |
| SiMAPK11 | SETIT_013899mg | TATA-box | TATA | 1272 | 1280 | core promoter element |
| SiMAPK11 | SETIT_013899mg | TATA-box | ATATAT | 1286 | 1298 | core promoter element |
| SiMAPK11 | SETIT_013899mg | TATA-box | TATA | 1289 | 1297 | core promoter element |
| SiMAPK11 | SETIT_013899mg | TATA-box | TATA | 1298 | 1306 | core promoter element |
| SiMAPK11 | SETIT_013899mg | GT1-motif | GGTTAAT | 983 | 997 | light responsive element |
| SiMAPK11 | SETIT_013899mg | MBSI | aaaAaaC(G/C)GTTA | 271.5 | 292.5 | MYB binding site involved in flavonoid biosynthetic genes regulation |
| SiMAPK11 | SETIT_013899mg | MBSI | aaaAaaC(G/C)GTTA | 304.5 | 325.5 | MYB binding site involved in flavonoid biosynthetic genes regulation |
| SiMAPK11 | SETIT_013899mg | TCCC-motif | TCTCCCT | 1580 | 1594 | light responsive element |
| SiMAPK11 | SETIT_013899mg | TCCC-motif | TCTCCCT | 1916 | 1930 | light responsive element |
| SiMAPK11 | SETIT_013899mg | LAMP-element | CTTTATCA | 713 | 729 | light responsive element |
| SiMAPK11 | SETIT_013899mg | I-box | GTATAAGGCC | 1116 | 1134 | light responsive element |
| SiMAPK11 | SETIT_013899mg | I-box | AGATAAGG | 1755 | 1771 | light responsive element |
| SiMAPK14 | SETIT_017554mg | ACE | GCGACGTACC | 488 | 506 | light responsive element |
| SiMAPK14 | SETIT_017554mg | TCA-element | TCAGAAGAGG | 1518 | 1536 | salicylic acid response element |
| SiMAPK14 | SETIT_017554mg | ABRE | CACGTG | 268 | 280 | abscisic acid response element |
| SiMAPK14 | SETIT_017554mg | ABRE | ACGTG | 270 | 280 | abscisic acid response element |
| SiMAPK14 | SETIT_017554mg | ABRE | ACGTG | 323 | 333 | abscisic acid response element |
| SiMAPK14 | SETIT_017554mg | ABRE | ACGTG | 1577 | 1587 | abscisic acid response element |
| SiMAPK14 | SETIT_017554mg | A-box | CCGTCC | 832 | 844 | cis-acting regulatory element |
| SiMAPK14 | SETIT_017554mg | ARE | AAACCA | 1083 | 1095 | anaerobic inducing element |
| SiMAPK14 | SETIT_017554mg | ARE | AAACCA | 1680 | 1692 | anaerobic inducing element |
| SiMAPK14 | SETIT_017554mg | G-box | CACGTG | 268 | 280 | light responsive element |
| SiMAPK14 | SETIT_017554mg | G-box | CACGTC | 322 | 334 | light responsive element |
| SiMAPK14 | SETIT_017554mg | G-box | CACGAC | 358 | 370 | light responsive element |
| SiMAPK14 | SETIT_017554mg | G-box | CACGAC | 509 | 521 | light responsive element |
| SiMAPK14 | SETIT_017554mg | G-Box | CACGTG | 268 | 280 | light responsive element |
| SiMAPK14 | SETIT_017554mg | G-Box | CACGTT | 1576 | 1588 | light responsive element |
| SiMAPK14 | SETIT_017554mg | CGTCA-motif | CGTCA | 163 | 173 | MeJA response regulatory element |
| SiMAPK14 | SETIT_017554mg | CGTCA-motif | CGTCA | 325 | 335 | MeJA response regulatory element |
| SiMAPK14 | SETIT_017554mg | TGACG-motif | TGACG | 163 | 173 | MeJA response regulatory element |
| SiMAPK14 | SETIT_017554mg | TGACG-motif | TGACG | 325 | 335 | MeJA response regulatory element |
| SiMAPK14 | SETIT_017554mg | CAAT-box | CAAAT | -4 | 6 | common cis-acting element |
| SiMAPK14 | SETIT_017554mg | CAAT-box | CAAAT | 39 | 49 | common cis-acting element |
| SiMAPK14 | SETIT_017554mg | CAAT-box | CAAAT | 81 | 91 | common cis-acting element |
| SiMAPK14 | SETIT_017554mg | CAAT-box | CCAAT | 342 | 352 | common cis-acting element |
| SiMAPK14 | SETIT_017554mg | CAAT-box | CCAAT | 946 | 956 | common cis-acting element |
| SiMAPK14 | SETIT_017554mg | CAAT-box | CAAAT | 971 | 981 | common cis-acting element |
| SiMAPK14 | SETIT_017554mg | CAAT-box | CCAAT | 1094 | 1104 | common cis-acting element |
| SiMAPK14 | SETIT_017554mg | CAAT-box | CAAAT | 1169 | 1179 | common cis-acting element |
| SiMAPK14 | SETIT_017554mg | CAAT-box | CAAAT | 1301 | 1311 | common cis-acting element |
| SiMAPK14 | SETIT_017554mg | CAAT-box | CCAAT | 1532 | 1542 | common cis-acting element |
| SiMAPK14 | SETIT_017554mg | CAAT-box | CAAAT | 1589 | 1599 | common cis-acting element |
| SiMAPK14 | SETIT_017554mg | CAAT-box | CCAAT | 1727 | 1737 | common cis-acting element |
| SiMAPK14 | SETIT_017554mg | CAAT-box | CAAAT | 1756 | 1766 | common cis-acting element |
| SiMAPK14 | SETIT_017554mg | CAAT-box | CAAAT | 1912 | 1922 | common cis-acting element |
| SiMAPK14 | SETIT_017554mg | TATA-box | ATTATA | 110 | 122 | core promoter element |
| SiMAPK14 | SETIT_017554mg | TATA-box | TATAA | 112 | 122 | core promoter element |
| SiMAPK14 | SETIT_017554mg | TATA-box | TATA | 114 | 122 | core promoter element |
| SiMAPK14 | SETIT_017554mg | TATA-box | ATTATA | 134 | 146 | core promoter element |
| SiMAPK14 | SETIT_017554mg | TATA-box | TATAA | 136 | 146 | core promoter element |
| SiMAPK14 | SETIT_017554mg | TATA-box | TATA | 138 | 146 | core promoter element |
| SiMAPK14 | SETIT_017554mg | TATA-box | TATA | 1141 | 1149 | core promoter element |
| SiMAPK14 | SETIT_017554mg | TATA-box | TACAAAA | 1451 | 1465 | core promoter element |
| SiMAPK14 | SETIT_017554mg | TATA-box | ATTATA | 1647 | 1659 | core promoter element |
| SiMAPK14 | SETIT_017554mg | TATA-box | TATAA | 1649 | 1659 | core promoter element |
| SiMAPK14 | SETIT_017554mg | TATA-box | TATA | 1651 | 1659 | core promoter element |
| SiMAPK14 | SETIT_017554mg | TATA-box | TATA | 1667 | 1675 | core promoter element |
| SiMAPK14 | SETIT_017554mg | TATA-box | TATA | 1733 | 1741 | core promoter element |
| SiMAPK14 | SETIT_017554mg | TATA-box | TATAA | 1878 | 1888 | core promoter element |
| SiMAPK14 | SETIT_017554mg | TATA-box | TATA | 1880 | 1888 | core promoter element |
| SiMAPK14 | SETIT_017554mg | TATA-box | TATAAAT | 1916 | 1930 | core promoter element |
| SiMAPK14 | SETIT_017554mg | TATA-box | TATAAA | 1918 | 1930 | core promoter element |
| SiMAPK14 | SETIT_017554mg | TATA-box | TATAA | 1920 | 1930 | core promoter element |
| SiMAPK14 | SETIT_017554mg | TATA-box | TATA | 1922 | 1930 | core promoter element |
| SiMAPK14 | SETIT_017554mg | TATA-box | TATATAA | 1954 | 1968 | core promoter element |
| SiMAPK14 | SETIT_017554mg | TATA-box | TATATA | 1956 | 1968 | core promoter element |
| SiMAPK14 | SETIT_017554mg | TATA-box | TATA | 1960 | 1968 | core promoter element |
| SiMAPK14 | SETIT_017554mg | GC-motif | CCCCCG | 542 | 554 | hypoxia-specifically induced elements |
| SiMAPK14 | SETIT_017554mg | GC-motif | CCCCCG | 1007 | 1019 | hypoxia-specifically induced elements |
| SiMAPK14 | SETIT_017554mg | Sp1 | GGGCGG | 371 | 383 | light responsive element |
| SiMAPK14 | SETIT_017554mg | Sp1 | GGGCGG | 448 | 460 | light responsive element |
| SiMAPK14 | SETIT_017554mg | Sp1 | GGGCGG | 724 | 736 | light responsive element |
| SiMAPK14 | SETIT_017554mg | Sp1 | GGGCGG | 729 | 741 | light responsive element |
| SiMAPK14 | SETIT_017554mg | GT1-motif | GTGTGTGAA | 188 | 206 | light responsive element |
| SiMAPK14 | SETIT_017554mg | MBS | CAACTG | 1615 | 1627 | MYB binding site involved in drought-inducibility |
| SiMAPK14 | SETIT_017554mg | Box 4 | ATTAAT | 1561 | 1573 | light responsive element |
| SiMAPK14 | SETIT_017554mg | I-box | TAGATAACC | 1853 | 1871 | light responsive element |
| SiMAPK14 | SETIT_017554mg | GATA-motif | GATAGGG | 1022 | 1036 | light responsive element |
| SiMAPK16-1 | SETIT_026197mg | ABRE | ACGTG | 298 | 308 | abscisic acid response element |
| SiMAPK16-1 | SETIT_026197mg | ABRE | CGCACGTGTC | 603 | 621 | abscisic acid response element |
| SiMAPK16-1 | SETIT_026197mg | ABRE | ACGTG | 1236 | 1246 | abscisic acid response element |
| SiMAPK16-1 | SETIT_026197mg | A-box | CCGTCC | 197 | 209 | cis-acting regulatory element |
| SiMAPK16-1 | SETIT_026197mg | A-box | CCGTCC | 764 | 776 | cis-acting regulatory element |
| SiMAPK16-1 | SETIT_026197mg | ARE | AAACCA | 382 | 394 | anaerobic inducing element |
| SiMAPK16-1 | SETIT_026197mg | ARE | AAACCA | 403 | 415 | anaerobic inducing element |
| SiMAPK16-1 | SETIT_026197mg | ARE | AAACCA | 409 | 421 | anaerobic inducing element |
| SiMAPK16-1 | SETIT_026197mg | ARE | AAACCA | 1486 | 1498 | anaerobic inducing element |
| SiMAPK16-1 | SETIT_026197mg | ARE | AAACCA | 1661 | 1673 | anaerobic inducing element |
| SiMAPK16-1 | SETIT_026197mg | ARE | AAACCA | 1869 | 1881 | anaerobic inducing element |
| SiMAPK16-1 | SETIT_026197mg | G-box | CACGTC | 296 | 308 | light responsive element |
| SiMAPK16-1 | SETIT_026197mg | G-box | TACGTG | 1235 | 1247 | light responsive element |
| SiMAPK16-1 | SETIT_026197mg | TGACG-motif | TGACG | 847 | 857 | MeJA response regulatory element |
| SiMAPK16-1 | SETIT_026197mg | TGACG-motif | TGACG | 1546 | 1556 | MeJA response regulatory element |
| SiMAPK16-1 | SETIT_026197mg | CGTCA-motif | CGTCA | 847 | 857 | MeJA response regulatory element |
| SiMAPK16-1 | SETIT_026197mg | CGTCA-motif | CGTCA | 1546 | 1556 | MeJA response regulatory element |
| SiMAPK16-1 | SETIT_026197mg | CAT-box | GCCACT | 1859 | 1871 | meristem expression regulatory element |
| SiMAPK16-1 | SETIT_026197mg | GCN4_motif | TGAGTCA | 1406 | 1420 | endosperm expression regulatory element |
| SiMAPK16-1 | SETIT_026197mg | CAAT-box | CAAAT | 127 | 137 | common cis-acting element |
| SiMAPK16-1 | SETIT_026197mg | CAAT-box | CCAAT | 304 | 314 | common cis-acting element |
| SiMAPK16-1 | SETIT_026197mg | CAAT-box | CAAAT | 380 | 390 | common cis-acting element |
| SiMAPK16-1 | SETIT_026197mg | CAAT-box | CAAAT | 414 | 424 | common cis-acting element |
| SiMAPK16-1 | SETIT_026197mg | CAAT-box | CCAAT | 431 | 441 | common cis-acting element |
| SiMAPK16-1 | SETIT_026197mg | CAAT-box | CCAAT | 488 | 498 | common cis-acting element |
| SiMAPK16-1 | SETIT_026197mg | CAAT-box | CCAAT | 735 | 745 | common cis-acting element |
| SiMAPK16-1 | SETIT_026197mg | CAAT-box | CCAAT | 958 | 968 | common cis-acting element |
| SiMAPK16-1 | SETIT_026197mg | CAAT-box | CAAAT | 1016 | 1026 | common cis-acting element |
| SiMAPK16-1 | SETIT_026197mg | CAAT-box | CCAAT | 1046 | 1056 | common cis-acting element |
| SiMAPK16-1 | SETIT_026197mg | CAAT-box | CAAAT | 1065 | 1075 | common cis-acting element |
| SiMAPK16-1 | SETIT_026197mg | CAAT-box | CCAAT | 1433 | 1443 | common cis-acting element |
| SiMAPK16-1 | SETIT_026197mg | CAAT-box | CAAAT | 1438 | 1448 | common cis-acting element |
| SiMAPK16-1 | SETIT_026197mg | CAAT-box | CAAAT | 1515 | 1525 | common cis-acting element |
| SiMAPK16-1 | SETIT_026197mg | CAAT-box | CAAAT | 1525 | 1535 | common cis-acting element |
| SiMAPK16-1 | SETIT_026197mg | CAAT-box | CAAAT | 1581 | 1591 | common cis-acting element |
| SiMAPK16-1 | SETIT_026197mg | CAAT-box | CAAAT | 1625 | 1635 | common cis-acting element |
| SiMAPK16-1 | SETIT_026197mg | CAAT-box | CAAAT | 1708 | 1718 | common cis-acting element |
| SiMAPK16-1 | SETIT_026197mg | CAAT-box | CAAAT | 1733 | 1743 | common cis-acting element |
| SiMAPK16-1 | SETIT_026197mg | CAAT-box | CAAAT | 1765 | 1775 | common cis-acting element |
| SiMAPK16-1 | SETIT_026197mg | TATA-box | TATAA | 19 | 29 | core promoter element |
| SiMAPK16-1 | SETIT_026197mg | TATA-box | TATA | 21 | 29 | core promoter element |
| SiMAPK16-1 | SETIT_026197mg | TATA-box | ATATAT | 636 | 648 | core promoter element |
| SiMAPK16-1 | SETIT_026197mg | TATA-box | TATA | 639 | 647 | core promoter element |
| SiMAPK16-1 | SETIT_026197mg | TATA-box | TATA | 911 | 919 | core promoter element |
| SiMAPK16-1 | SETIT_026197mg | TATA-box | TATATA | 1004 | 1016 | core promoter element |
| SiMAPK16-1 | SETIT_026197mg | TATA-box | ATATAT | 1005 | 1017 | core promoter element |
| SiMAPK16-1 | SETIT_026197mg | TATA-box | TATATA | 1006 | 1018 | core promoter element |
| SiMAPK16-1 | SETIT_026197mg | TATA-box | TATA | 1010 | 1018 | core promoter element |
| SiMAPK16-1 | SETIT_026197mg | TATA-box | TATACA | 1195 | 1207 | core promoter element |
| SiMAPK16-1 | SETIT_026197mg | TATA-box | TATA | 1199 | 1207 | core promoter element |
| SiMAPK16-1 | SETIT_026197mg | TATA-box | TATAAAT | 1819 | 1833 | core promoter element |
| SiMAPK16-1 | SETIT_026197mg | TATA-box | TATAAA | 1821 | 1833 | core promoter element |
| SiMAPK16-1 | SETIT_026197mg | TATA-box | TATAA | 1823 | 1833 | core promoter element |
| SiMAPK16-1 | SETIT_026197mg | TATA-box | TATA | 1825 | 1833 | core promoter element |
| SiMAPK16-1 | SETIT_026197mg | MBS | CAACTG | 1337 | 1349 | MYB binding site involved in drought-inducibility |
| SiMAPK16-1 | SETIT_026197mg | CCAAT-box | CAACGG | 481 | 493 | MYBHv1 binding site |
| SiMAPK16-1 | SETIT_026197mg | Box 4 | ATTAAT | 1916 | 1928 | light responsive element |
| SiMAPK16-1 | SETIT_026197mg | GATA-motif | AAGGATAAGG | 1321 | 1339 | light responsive element |
| SiMAPK16-1 | SETIT_026197mg | TCT-motif | TCTTAC | 435 | 447 | light responsive element |
| SiMAPK16-1 | SETIT_026197mg | AuxRE | TGTCTCAATAAG | 96 | 118 | auxin response element |
| SiMAPK16-1 | SETIT_026197mg | HD-Zip 3 | GTAAT(G/C)ATTAC | 1595.5 | 1614.5 | protein binding site |
| SiMAPK16-2 | SETIT_021645mg | MSA-like | TCCAACGGT | 397 | 415 | cell cycle control element |
| SiMAPK16-2 | SETIT_021645mg | LTR | CCGAAA | 595 | 607 | low temperature response element |
| SiMAPK16-2 | SETIT_021645mg | TCA-element | CCATCTTTTT | 168 | 186 | salicylic acid response element |
| SiMAPK16-2 | SETIT_021645mg | ABRE | GCAACGTGTC | 238 | 256 | abscisic acid response element |
| SiMAPK16-2 | SETIT_021645mg | ARE | AAACCA | 901 | 913 | anaerobic inducing element |
| SiMAPK16-2 | SETIT_021645mg | ARE | AAACCA | 1625 | 1637 | anaerobic inducing element |
| SiMAPK16-2 | SETIT_021645mg | AuxRR-core | GGTCCAT | 23 | 37 | auxin-responsive element |
| SiMAPK16-2 | SETIT_021645mg | G-box | CACGAC | 1217 | 1229 | light responsive element |
| SiMAPK16-2 | SETIT_021645mg | G-box | GCCACGTGGA | 1454 | 1472 | light responsive element |
| SiMAPK16-2 | SETIT_021645mg | CAT-box | GCCACT | 1634 | 1646 | meristem expression regulatory element |
| SiMAPK16-2 | SETIT_021645mg | CAAT-box | CAAAT | 113 | 123 | common cis-acting element |
| SiMAPK16-2 | SETIT_021645mg | CAAT-box | CAAAT | 324 | 334 | common cis-acting element |
| SiMAPK16-2 | SETIT_021645mg | CAAT-box | CCAAT | 396 | 406 | common cis-acting element |
| SiMAPK16-2 | SETIT_021645mg | CAAT-box | CAAAT | 458 | 468 | common cis-acting element |
| SiMAPK16-2 | SETIT_021645mg | CAAT-box | CAAAT | 520 | 530 | common cis-acting element |
| SiMAPK16-2 | SETIT_021645mg | CAAT-box | CAAAT | 628 | 638 | common cis-acting element |
| SiMAPK16-2 | SETIT_021645mg | CAAT-box | CCAAT | 684 | 694 | common cis-acting element |
| SiMAPK16-2 | SETIT_021645mg | CAAT-box | CCAAT | 690 | 700 | common cis-acting element |
| SiMAPK16-2 | SETIT_021645mg | CAAT-box | CAAAT | 755 | 765 | common cis-acting element |
| SiMAPK16-2 | SETIT_021645mg | CAAT-box | CCAAT | 818 | 828 | common cis-acting element |
| SiMAPK16-2 | SETIT_021645mg | CAAT-box | CAAAT | 970 | 980 | common cis-acting element |
| SiMAPK16-2 | SETIT_021645mg | CAAT-box | CAAAT | 1036 | 1046 | common cis-acting element |
| SiMAPK16-2 | SETIT_021645mg | CAAT-box | CCAAT | 1205 | 1215 | common cis-acting element |
| SiMAPK16-2 | SETIT_021645mg | CAAT-box | CCAAT | 1478 | 1488 | common cis-acting element |
| SiMAPK16-2 | SETIT_021645mg | CAAT-box | CCAAT | 1620 | 1630 | common cis-acting element |
| SiMAPK16-2 | SETIT_021645mg | TATA-box | ccTATAAAaa | 152 | 170 | core promoter element |
| SiMAPK16-2 | SETIT_021645mg | TATA-box | TATA | 191 | 199 | core promoter element |
| SiMAPK16-2 | SETIT_021645mg | TATA-box | ATTATA | 757 | 769 | core promoter element |
| SiMAPK16-2 | SETIT_021645mg | TATA-box | TATAA | 759 | 769 | core promoter element |
| SiMAPK16-2 | SETIT_021645mg | TATA-box | TATA | 761 | 769 | core promoter element |
| SiMAPK16-2 | SETIT_021645mg | TATA-box | ATATAA | 1614 | 1626 | core promoter element |
| SiMAPK16-2 | SETIT_021645mg | TATA-box | TATA | 1617 | 1625 | core promoter element |
| SiMAPK16-2 | SETIT_021645mg | GC-motif | CCCCCG | 1493 | 1505 | hypoxia-specifically induced elements |
| SiMAPK16-2 | SETIT_021645mg | P-box | CCTTTTG | 1481 | 1495 | gibberellin-responsive element |
| SiMAPK16-2 | SETIT_021645mg | Sp1 | GGGCGG | 1364 | 1376 | light responsive element |
| SiMAPK16-2 | SETIT_021645mg | Sp1 | GGGCGG | 1496 | 1508 | light responsive element |
| SiMAPK16-2 | SETIT_021645mg | Sp1 | GGGCGG | 1500 | 1512 | light responsive element |
| SiMAPK16-2 | SETIT_021645mg | Sp1 | GGGCGG | 1714 | 1726 | light responsive element |
| SiMAPK16-2 | SETIT_021645mg | Sp1 | GGGCGG | 1766 | 1778 | light responsive element |
| SiMAPK16-2 | SETIT_021645mg | MBS | CAACTG | 103 | 115 | MYB binding site involved in drought-inducibility |
| SiMAPK16-2 | SETIT_021645mg | MBS | CAACTG | 828 | 840 | MYB binding site involved in drought-inducibility |
| SiMAPK16-2 | SETIT_021645mg | MBS | CAACTG | 854 | 866 | MYB binding site involved in drought-inducibility |
| SiMAPK16-2 | SETIT_021645mg | CCAAT-box | CAACGG | 402 | 414 | MYBHv1 binding site |
| SiMAPK16-2 | SETIT_021645mg | CCAAT-box | CAACGG | 897 | 909 | MYBHv1 binding site |
| SiMAPK16-2 | SETIT_021645mg | Box 4 | ATTAAT | 948 | 960 | light responsive element |
| SiMAPK16-2 | SETIT_021645mg | TCCC-motif | TCTCCCT | 1650 | 1664 | light responsive element |
| SiMAPK16-2 | SETIT_021645mg | TCCC-motif | TCTCCCT | 1805 | 1819 | light responsive element |
| SiMAPK16-2 | SETIT_021645mg | chs-CMA2a | TCACTTGA | 1655 | 1671 | light responsive element |
| SiMAPK17-1 | SETIT_006144mg | TC-rich repeats | GTTTTCTTAC | 1274 | 1292 | defense and stress response elements |
| SiMAPK17-1 | SETIT_006144mg | ABRE | ACGTG | 756 | 766 | abscisic acid response element |
| SiMAPK17-1 | SETIT_006144mg | ABRE | ACGTG | 1068 | 1078 | abscisic acid response element |
| SiMAPK17-1 | SETIT_006144mg | ABRE | CACGTG | 1286 | 1298 | abscisic acid response element |
| SiMAPK17-1 | SETIT_006144mg | ABRE | ACGTG | 1288 | 1298 | abscisic acid response element |
| SiMAPK17-1 | SETIT_006144mg | ABRE | ACGTG | 1391 | 1401 | abscisic acid response element |
| SiMAPK17-1 | SETIT_006144mg | ABRE | ACGTG | 1661 | 1671 | abscisic acid response element |
| SiMAPK17-1 | SETIT_006144mg | ABRE | ACGTG | 1831 | 1841 | abscisic acid response element |
| SiMAPK17-1 | SETIT_006144mg | A-box | CCGTCC | 1854 | 1866 | cis-acting regulatory element |
| SiMAPK17-1 | SETIT_006144mg | ARE | AAACCA | 810 | 822 | anaerobic inducing element |
| SiMAPK17-1 | SETIT_006144mg | AuxRR-core | GGTCCAT | 532 | 546 | auxin-responsive element |
| SiMAPK17-1 | SETIT_006144mg | G-box | CACGTC | 754 | 766 | light responsive element |
| SiMAPK17-1 | SETIT_006144mg | G-box | TACGTG | 1067 | 1079 | light responsive element |
| SiMAPK17-1 | SETIT_006144mg | G-box | CACGTG | 1286 | 1298 | light responsive element |
| SiMAPK17-1 | SETIT_006144mg | G-box | CACGTC | 1830 | 1842 | light responsive element |
| SiMAPK17-1 | SETIT_006144mg | G-Box | CACGTG | 1286 | 1298 | light responsive element |
| SiMAPK17-1 | SETIT_006144mg | G-Box | CACGTT | 1389 | 1401 | light responsive element |
| SiMAPK17-1 | SETIT_006144mg | G-Box | CACGTT | 1660 | 1672 | light responsive element |
| SiMAPK17-1 | SETIT_006144mg | CGTCA-motif | CGTCA | 754 | 764 | MeJA response regulatory element |
| SiMAPK17-1 | SETIT_006144mg | CGTCA-motif | CGTCA | 1771 | 1781 | MeJA response regulatory element |
| SiMAPK17-1 | SETIT_006144mg | TGACG-motif | TGACG | 754 | 764 | MeJA response regulatory element |
| SiMAPK17-1 | SETIT_006144mg | TGACG-motif | TGACG | 1771 | 1781 | MeJA response regulatory element |
| SiMAPK17-1 | SETIT_006144mg | O2-site | GATGA(C/T)(A/G)TG(A/G) | 15.5 | 32.5 | Zein metabolism regulatory elements |
| SiMAPK17-1 | SETIT_006144mg | CAT-box | GCCACT | 1510 | 1522 | meristem expression regulatory element |
| SiMAPK17-1 | SETIT_006144mg | CAAT-box | CCAAT | 64 | 74 | common cis-acting element |
| SiMAPK17-1 | SETIT_006144mg | CAAT-box | CAAAT | 192 | 202 | common cis-acting element |
| SiMAPK17-1 | SETIT_006144mg | CAAT-box | CAAAT | 219 | 229 | common cis-acting element |
| SiMAPK17-1 | SETIT_006144mg | CAAT-box | CCAAT | 336 | 346 | common cis-acting element |
| SiMAPK17-1 | SETIT_006144mg | CAAT-box | CAACCAACTCC | 415 | 435 | common cis-acting element |
| SiMAPK17-1 | SETIT_006144mg | CAAT-box | CAAAT | 585 | 595 | common cis-acting element |
| SiMAPK17-1 | SETIT_006144mg | CAAT-box | CAAAT | 864 | 874 | common cis-acting element |
| SiMAPK17-1 | SETIT_006144mg | CAAT-box | CCAAT | 904 | 914 | common cis-acting element |
| SiMAPK17-1 | SETIT_006144mg | CAAT-box | CCAAT | 932 | 942 | common cis-acting element |
| SiMAPK17-1 | SETIT_006144mg | CAAT-box | TGCCAAC | 1084 | 1098 | common cis-acting element |
| SiMAPK17-1 | SETIT_006144mg | CAAT-box | CCAAT | 1710 | 1720 | common cis-acting element |
| SiMAPK17-1 | SETIT_006144mg | CAAT-box | CCAAT | 1778 | 1788 | common cis-acting element |
| SiMAPK17-1 | SETIT_006144mg | CAAT-box | CCAAT | 1965 | 1975 | common cis-acting element |
| SiMAPK17-1 | SETIT_006144mg | TATA-box | TATA | 120 | 128 | core promoter element |
| SiMAPK17-1 | SETIT_006144mg | TATA-box | TACAAAA | 128 | 142 | core promoter element |
| SiMAPK17-1 | SETIT_006144mg | TATA-box | TATAAAA | 287 | 301 | core promoter element |
| SiMAPK17-1 | SETIT_006144mg | TATA-box | TATAAA | 289 | 301 | core promoter element |
| SiMAPK17-1 | SETIT_006144mg | TATA-box | TATAA | 291 | 301 | core promoter element |
| SiMAPK17-1 | SETIT_006144mg | TATA-box | TATA | 293 | 301 | core promoter element |
| SiMAPK17-1 | SETIT_006144mg | TATA-box | TACAAAA | 508 | 522 | core promoter element |
| SiMAPK17-1 | SETIT_006144mg | TATA-box | TACAAAA | 779 | 793 | core promoter element |
| SiMAPK17-1 | SETIT_006144mg | TATA-box | TATA | 971 | 979 | core promoter element |
| SiMAPK17-1 | SETIT_006144mg | TATA-box | TATA | 1032 | 1040 | core promoter element |
| SiMAPK17-1 | SETIT_006144mg | GC-motif | CCCCCG | 6 | 18 | hypoxia-specifically induced elements |
| SiMAPK17-1 | SETIT_006144mg | P-box | CCTTTTG | 794 | 808 | gibberellin-responsive element |
| SiMAPK17-1 | SETIT_006144mg | Sp1 | GGGCGG | 1922 | 1934 | light responsive element |
| SiMAPK17-1 | SETIT_006144mg | Sp1 | GGGCGG | 1927 | 1939 | light responsive element |
| SiMAPK17-1 | SETIT_006144mg | GT1-motif | GGTTAA | 356 | 368 | light responsive element |
| SiMAPK17-1 | SETIT_006144mg | MBS | CAACTG | 656 | 668 | MYB binding site involved in drought-inducibility |
| SiMAPK17-1 | SETIT_006144mg | MBS | CAACTG | 1015 | 1027 | MYB binding site involved in drought-inducibility |
| SiMAPK17-1 | SETIT_006144mg | CCAAT-box | CAACGG | 9 | 21 | MYBHv1 binding site |
| SiMAPK17-1 | SETIT_006144mg | CCAAT-box | CAACGG | 454 | 466 | MYBHv1 binding site |
| SiMAPK17-1 | SETIT_006144mg | Box 4 | ATTAAT | 787 | 799 | light responsive element |
| SiMAPK17-1 | SETIT_006144mg | ATCT-motif | AATCTAATCC | 1771 | 1789 | light responsive element |
| SiMAPK17-2 | SETIT_016957mg | AT-rich element | ATAGAAATCAA | 1709 | 1729 | ATBP-1 binding site |
| SiMAPK17-2 | SETIT_016957mg | TATC-box | TATCCCA | 1694 | 1708 | gibberellin-responsive element |
| SiMAPK17-2 | SETIT_016957mg | TCA-element | CCATCTTTTT | 140 | 158 | salicylic acid response element |
| SiMAPK17-2 | SETIT_016957mg | A-box | CCGTCC | 1000 | 1012 | cis-acting regulatory element |
| SiMAPK17-2 | SETIT_016957mg | ARE | AAACCA | 660 | 672 | anaerobic inducing element |
| SiMAPK17-2 | SETIT_016957mg | ARE | AAACCA | 864 | 876 | anaerobic inducing element |
| SiMAPK17-2 | SETIT_016957mg | CGTCA-motif | CGTCA | 1323 | 1333 | MeJA response regulatory element |
| SiMAPK17-2 | SETIT_016957mg | TGACG-motif | TGACG | 1323 | 1333 | MeJA response regulatory element |
| SiMAPK17-2 | SETIT_016957mg | CAT-box | GCCACT | 1361 | 1373 | meristem expression regulatory element |
| SiMAPK17-2 | SETIT_016957mg | CAAT-box | CCAAT | 57 | 67 | common cis-acting element |
| SiMAPK17-2 | SETIT_016957mg | CAAT-box | CAAAT | 77 | 87 | common cis-acting element |
| SiMAPK17-2 | SETIT_016957mg | CAAT-box | CCAAT | 137 | 147 | common cis-acting element |
| SiMAPK17-2 | SETIT_016957mg | CAAT-box | CCAAT | 169 | 179 | common cis-acting element |
| SiMAPK17-2 | SETIT_016957mg | CAAT-box | CAAAT | 351 | 361 | common cis-acting element |
| SiMAPK17-2 | SETIT_016957mg | CAAT-box | TGCCAAC | 401 | 415 | common cis-acting element |
| SiMAPK17-2 | SETIT_016957mg | CAAT-box | CAAAT | 445 | 455 | common cis-acting element |
| SiMAPK17-2 | SETIT_016957mg | CAAT-box | CAAAT | 561 | 571 | common cis-acting element |
| SiMAPK17-2 | SETIT_016957mg | CAAT-box | CCAAT | 704 | 714 | common cis-acting element |
| SiMAPK17-2 | SETIT_016957mg | CAAT-box | CCAAT | 759 | 769 | common cis-acting element |
| SiMAPK17-2 | SETIT_016957mg | CAAT-box | CCAAT | 1035 | 1045 | common cis-acting element |
| SiMAPK17-2 | SETIT_016957mg | CAAT-box | CCAAT | 1493 | 1503 | common cis-acting element |
| SiMAPK17-2 | SETIT_016957mg | CAAT-box | TGCCAAC | 1547 | 1561 | common cis-acting element |
| SiMAPK17-2 | SETIT_016957mg | CAAT-box | CAAAT | 1657 | 1667 | common cis-acting element |
| SiMAPK17-2 | SETIT_016957mg | CAAT-box | CCAAT | 1754 | 1764 | common cis-acting element |
| SiMAPK17-2 | SETIT_016957mg | CAAT-box | CCAAT | 1765 | 1775 | common cis-acting element |
| SiMAPK17-2 | SETIT_016957mg | CAAT-box | CAAAT | 1846 | 1856 | common cis-acting element |
| SiMAPK17-2 | SETIT_016957mg | TATA-box | TATA | 235 | 243 | core promoter element |
| SiMAPK17-2 | SETIT_016957mg | TATA-box | TACAAAA | 261 | 275 | core promoter element |
| SiMAPK17-2 | SETIT_016957mg | TATA-box | TATA | 367 | 375 | core promoter element |
| SiMAPK17-2 | SETIT_016957mg | TATA-box | TATA | 400 | 408 | core promoter element |
| SiMAPK17-2 | SETIT_016957mg | TATA-box | TACAAAA | 633 | 647 | core promoter element |
| SiMAPK17-2 | SETIT_016957mg | TATA-box | TATA | 824 | 832 | core promoter element |
| SiMAPK17-2 | SETIT_016957mg | TATA-box | TATACA | 1287 | 1299 | core promoter element |
| SiMAPK17-2 | SETIT_016957mg | TATA-box | TATA | 1291 | 1299 | core promoter element |
| SiMAPK17-2 | SETIT_016957mg | TATA-box | TATTTAAA | 1385 | 1401 | core promoter element |
| SiMAPK17-2 | SETIT_016957mg | TATA-box | TATA | 1617 | 1625 | core promoter element |
| SiMAPK17-2 | SETIT_016957mg | TATA-box | ATATAT | 1699 | 1711 | core promoter element |
| SiMAPK17-2 | SETIT_016957mg | TATA-box | TATA | 1702 | 1710 | core promoter element |
| SiMAPK17-2 | SETIT_016957mg | TATA-box | TATA | 1826 | 1834 | core promoter element |
| SiMAPK17-2 | SETIT_016957mg | TATA-box | TATAAA | 1978 | 1990 | core promoter element |
| SiMAPK17-2 | SETIT_016957mg | TATA-box | TATAA | 1980 | 1990 | core promoter element |
| SiMAPK17-2 | SETIT_016957mg | TATA-box | TATA | 1982 | 1990 | core promoter element |
| SiMAPK17-2 | SETIT_016957mg | P-box | CCTTTTG | 1135 | 1149 | gibberellin-responsive element |
| SiMAPK17-2 | SETIT_016957mg | GT1-motif | GGTTAA | 568 | 580 | light responsive element |
| SiMAPK17-2 | SETIT_016957mg | MRE | AACCTAA | 569 | 583 | light responsive element |
| SiMAPK17-2 | SETIT_016957mg | ATC-motif | AGTAATCT | 1235 | 1251 | light responsive element |
| SiMAPK17-2 | SETIT_016957mg | Box 4 | ATTAAT | 385 | 397 | light responsive element |
| SiMAPK17-2 | SETIT_016957mg | TCT-motif | TCTTAC | 1526 | 1538 | light responsive element |
| SiMAPK17-2 | SETIT_016957mg | TCT-motif | TCTTAC | 1688 | 1700 | light responsive element |
| SiMAPK17-2 | SETIT_016957mg | I-box | ccttatcct | 106 | 124 | light responsive element |
| SiMAPK17-2 | SETIT_016957mg | I-box | GATAAGGGT | 108 | 126 | light responsive element |
| SiMAPK17-2 | SETIT_016957mg | GA-motif | ATAGATAA | 232 | 248 | light responsive element |
| SiMAPK17-2 | SETIT_016957mg | GATA-motif | AAGGATAAGG | 105 | 123 | light responsive element |
| SiMAPK17-2 | SETIT_016957mg | GATA-motif | AAGATAAGATT | 1236 | 1256 | light responsive element |
| SiMAPK17-2 | SETIT_016957mg | GATA-motif | AAGGATAAGG | 1400 | 1418 | light responsive element |
| SiMAPK17-2 | SETIT_016957mg | LS7 | CAGATTTATTTTTA | 702 | 728 | light responsive element |
| SiMAPK17-2 | SETIT_016957mg | LS7 | CAGATTTATTTTTA | 757 | 783 | light responsive element |
| SiMAPK17-2 | SETIT_016957mg | AE-box | AGAAACAA | 1862 | 1878 | light responsive element |
| SiMAPK17-2 | SETIT_016957mg | AE-box | AGAAACAA | 1880 | 1896 | light responsive element |
| SiMAPK20-1 | SETIT_000725mg | TCA-element | CCATCTTTTT | 1285 | 1303 | salicylic acid response element |
| SiMAPK20-1 | SETIT_000725mg | ABRE | GCCGCGTGGC | 222 | 240 | abscisic acid response element |
| SiMAPK20-1 | SETIT_000725mg | ABRE | CGCACGTGTC | 962 | 980 | abscisic acid response element |
| SiMAPK20-1 | SETIT_000725mg | ABRE | CACGTG | 967 | 979 | abscisic acid response element |
| SiMAPK20-1 | SETIT_000725mg | ABRE | ACGTG | 969 | 979 | abscisic acid response element |
| SiMAPK20-1 | SETIT_000725mg | ABRE | GCCGCGTGGC | 1880 | 1898 | abscisic acid response element |
| SiMAPK20-1 | SETIT_000725mg | A-box | CCGTCC | 195 | 207 | cis-acting regulatory element |
| SiMAPK20-1 | SETIT_000725mg | A-box | CCGTCC | 1437 | 1449 | cis-acting regulatory element |
| SiMAPK20-1 | SETIT_000725mg | A-box | CCGTCC | 1502 | 1514 | cis-acting regulatory element |
| SiMAPK20-1 | SETIT_000725mg | A-box | CCGTCC | 1571 | 1583 | cis-acting regulatory element |
| SiMAPK20-1 | SETIT_000725mg | A-box | CCGTCC | 1954 | 1966 | cis-acting regulatory element |
| SiMAPK20-1 | SETIT_000725mg | G-Box | CACGTG | 967 | 979 | light responsive element |
| SiMAPK20-1 | SETIT_000725mg | G-box | CACGAC | 415 | 427 | light responsive element |
| SiMAPK20-1 | SETIT_000725mg | G-box | CACGTG | 967 | 979 | light responsive element |
| SiMAPK20-1 | SETIT_000725mg | G-box | CACGAC | 1475 | 1487 | light responsive element |
| SiMAPK20-1 | SETIT_000725mg | CGTCA-motif | CGTCA | 474 | 484 | MeJA response regulatory element |
| SiMAPK20-1 | SETIT_000725mg | TGACG-motif | TGACG | 474 | 484 | MeJA response regulatory element |
| SiMAPK20-1 | SETIT_000725mg | CAAT-box | CCAAT | 1879 | 1889 | common cis-acting element |
| SiMAPK20-1 | SETIT_000725mg | TATA-box | TACAAAA | 1021 | 1035 | core promoter element |
| SiMAPK20-1 | SETIT_000725mg | GC-motif | CCCCCG | 532 | 544 | hypoxia-specifically induced elements |
| SiMAPK20-1 | SETIT_000725mg | GC-motif | CCCCCG | 606 | 618 | hypoxia-specifically induced elements |
| SiMAPK20-1 | SETIT_000725mg | GC-motif | CCCCCG | 650 | 662 | hypoxia-specifically induced elements |
| SiMAPK20-1 | SETIT_000725mg | GC-motif | CCCCCG | 675 | 687 | hypoxia-specifically induced elements |
| SiMAPK20-1 | SETIT_000725mg | GC-motif | CCCCCG | 681 | 693 | hypoxia-specifically induced elements |
| SiMAPK20-1 | SETIT_000725mg | GC-motif | CCCCCG | 1818 | 1830 | hypoxia-specifically induced elements |
| SiMAPK20-1 | SETIT_000725mg | Sp1 | GGGCGG | 658 | 670 | light responsive element |
| SiMAPK20-1 | SETIT_000725mg | Sp1 | GGGCGG | 678 | 690 | light responsive element |
| SiMAPK20-1 | SETIT_000725mg | Sp1 | GGGCGG | 711 | 723 | light responsive element |
| SiMAPK20-1 | SETIT_000725mg | Sp1 | GGGCGG | 766 | 778 | light responsive element |
| SiMAPK20-1 | SETIT_000725mg | Sp1 | GGGCGG | 780 | 792 | light responsive element |
| SiMAPK20-1 | SETIT_000725mg | Sp1 | GGGCGG | 1344 | 1356 | light responsive element |
| SiMAPK20-1 | SETIT_000725mg | Sp1 | GGGCGG | 1481 | 1493 | light responsive element |
| SiMAPK20-1 | SETIT_000725mg | Sp1 | GGGCGG | 1801 | 1813 | light responsive element |
| SiMAPK20-1 | SETIT_000725mg | Sp1 | GGGCGG | 1805 | 1817 | light responsive element |
| SiMAPK20-1 | SETIT_000725mg | GT1-motif | GGTTAA | 1355 | 1367 | light responsive element |
| SiMAPK20-1 | SETIT_000725mg | MBS | CAACTG | 496 | 508 | MYB binding site involved in drought-inducibility |
| SiMAPK20-1 | SETIT_000725mg | CCAAT-box | CAACGG | 748 | 760 | MYBHv1 binding site |
| SiMAPK20-1 | SETIT_000725mg | I-box | cGATAAGGCG | 592 | 610 | light responsive element |
| SiMAPK20-1 | SETIT_000725mg | TCCC-motif | TCTCCCT | 1614 | 1628 | light responsive element |
| SiMAPK20-1 | SETIT_000725mg | GTGGC-motif | CAGCGTGTGGC | 534 | 554 | light responsive element |
| SiMAPK20-2 | SETIT_000788mg | TGA-element | AACGAC | 68 | 80 | auxin-responsive element |
| SiMAPK20-2 | SETIT_000788mg | TC-rich repeats | ATTCTCTAAC | 747 | 765 | defense and stress response elements |
| SiMAPK20-2 | SETIT_000788mg | TATC-box | TATCCCA | 332 | 346 | gibberellin-responsive element |
| SiMAPK20-2 | SETIT_000788mg | LTR | CCGAAA | 196 | 208 | low temperature response element |
| SiMAPK20-2 | SETIT_000788mg | ABRE | ACGTG | 269 | 279 | abscisic acid response element |
| SiMAPK20-2 | SETIT_000788mg | ABRE | CACGTG | 740 | 752 | abscisic acid response element |
| SiMAPK20-2 | SETIT_000788mg | ABRE | ACGTG | 742 | 752 | abscisic acid response element |
| SiMAPK20-2 | SETIT_000788mg | ABRE | AACCCGG | 784 | 798 | abscisic acid response element |
| SiMAPK20-2 | SETIT_000788mg | ABRE | ACGTG | 990 | 1000 | abscisic acid response element |
| SiMAPK20-2 | SETIT_000788mg | ABRE | ACGTG | 1247 | 1257 | abscisic acid response element |
| SiMAPK20-2 | SETIT_000788mg | A-box | CCGTCC | 1353 | 1365 | cis-acting regulatory element |
| SiMAPK20-2 | SETIT_000788mg | A-box | CCGTCC | 1838 | 1850 | cis-acting regulatory element |
| SiMAPK20-2 | SETIT_000788mg | ARE | AAACCA | 213 | 225 | anaerobic inducing element |
| SiMAPK20-2 | SETIT_000788mg | G-box | CACGTG | 740 | 752 | light responsive element |
| SiMAPK20-2 | SETIT_000788mg | G-box | TACGTG | 988 | 1000 | light responsive element |
| SiMAPK20-2 | SETIT_000788mg | G-Box | CACGTT | 267 | 279 | light responsive element |
| SiMAPK20-2 | SETIT_000788mg | G-Box | CACGTG | 740 | 752 | light responsive element |
| SiMAPK20-2 | SETIT_000788mg | G-Box | CACGTT | 1245 | 1257 | light responsive element |
| SiMAPK20-2 | SETIT_000788mg | GCN4_motif | TGAGTCA | 647 | 661 | endosperm expression regulatory element |
| SiMAPK20-2 | SETIT_000788mg | GCN4_motif | TGAGTCA | 1488 | 1502 | endosperm expression regulatory element |
| SiMAPK20-2 | SETIT_000788mg | CAAT-box | CCAAT | 34 | 44 | common cis-acting element |
| SiMAPK20-2 | SETIT_000788mg | CAAT-box | CAAAT | 95 | 105 | common cis-acting element |
| SiMAPK20-2 | SETIT_000788mg | CAAT-box | CAAAT | 98 | 108 | common cis-acting element |
| SiMAPK20-2 | SETIT_000788mg | CAAT-box | CCAAT | 168 | 178 | common cis-acting element |
| SiMAPK20-2 | SETIT_000788mg | CAAT-box | CAAAT | 331 | 341 | common cis-acting element |
| SiMAPK20-2 | SETIT_000788mg | CAAT-box | CCAAT | 438 | 448 | common cis-acting element |
| SiMAPK20-2 | SETIT_000788mg | CAAT-box | CCAAT | 507 | 517 | common cis-acting element |
| SiMAPK20-2 | SETIT_000788mg | CAAT-box | CCAAT | 533 | 543 | common cis-acting element |
| SiMAPK20-2 | SETIT_000788mg | CAAT-box | CAAAT | 693 | 703 | common cis-acting element |
| SiMAPK20-2 | SETIT_000788mg | CAAT-box | CCAAT | 726 | 736 | common cis-acting element |
| SiMAPK20-2 | SETIT_000788mg | CAAT-box | CAAAT | 1939 | 1949 | common cis-acting element |
| SiMAPK20-2 | SETIT_000788mg | TATA-box | TATAA | 53 | 63 | core promoter element |
| SiMAPK20-2 | SETIT_000788mg | TATA-box | TATA | 55 | 63 | core promoter element |
| SiMAPK20-2 | SETIT_000788mg | TATA-box | TATAAA | 140 | 152 | core promoter element |
| SiMAPK20-2 | SETIT_000788mg | TATA-box | TATAA | 142 | 152 | core promoter element |
| SiMAPK20-2 | SETIT_000788mg | TATA-box | TATA | 144 | 152 | core promoter element |
| SiMAPK20-2 | SETIT_000788mg | TATA-box | TACAAAA | 391 | 405 | core promoter element |
| SiMAPK20-2 | SETIT_000788mg | TATA-box | ATTATA | 816 | 828 | core promoter element |
| SiMAPK20-2 | SETIT_000788mg | TATA-box | TATAA | 818 | 828 | core promoter element |
| SiMAPK20-2 | SETIT_000788mg | TATA-box | TATA | 820 | 828 | core promoter element |
| SiMAPK20-2 | SETIT_000788mg | TATA-box | TATA | 934 | 942 | core promoter element |
| SiMAPK20-2 | SETIT_000788mg | TATA-box | TATA | 985 | 993 | core promoter element |
| SiMAPK20-2 | SETIT_000788mg | GARE-motif | TCTGTTG | 260 | 274 | gibberellin-responsive element |
| SiMAPK20-2 | SETIT_000788mg | GARE-motif | TCTGTTG | 362 | 376 | gibberellin-responsive element |
| SiMAPK20-2 | SETIT_000788mg | P-box | CCTTTTG | -4 | 10 | gibberellin-responsive element |
| SiMAPK20-2 | SETIT_000788mg | GT1-motif | GGTTAAT | 161 | 175 | light responsive element |
| SiMAPK20-2 | SETIT_000788mg | GT1-motif | GGTTAA | 1444 | 1456 | light responsive element |
| SiMAPK20-2 | SETIT_000788mg | 3-AF1 binding site | TAAGAGAGGAA | 938 | 958 | light responsive element |
| SiMAPK20-2 | SETIT_000788mg | Sp1 | GGGCGG | 1318 | 1330 | light responsive element |
| SiMAPK20-2 | SETIT_000788mg | Sp1 | GGGCGG | 1338 | 1350 | light responsive element |
| SiMAPK20-2 | SETIT_000788mg | Sp1 | GGGCGG | 1801 | 1813 | light responsive element |
| SiMAPK20-2 | SETIT_000788mg | Sp1 | GGGCGG | 1902 | 1914 | light responsive element |
| SiMAPK20-2 | SETIT_000788mg | Sp1 | GGGCGG | 1906 | 1918 | light responsive element |
| SiMAPK20-2 | SETIT_000788mg | CCAAT-box | CAACGG | 158 | 170 | MYBHv1 binding site |
| SiMAPK20-2 | SETIT_000788mg | Box 4 | ATTAAT | 255 | 267 | light responsive element |
| SiMAPK20-2 | SETIT_000788mg | Box 4 | ATTAAT | 599 | 611 | light responsive element |
| SiMAPK20-2 | SETIT_000788mg | I-box | atGATAAGGTC | 1127 | 1147 | light responsive element |
| SiMAPK20-3 | SETIT_021560mg | ACE | GACACGTATG | 963 | 981 | light responsive element |
| SiMAPK20-3 | SETIT_021560mg | ABRE | TACGTGTC | 964 | 980 | abscisic acid response element |
| SiMAPK20-3 | SETIT_021560mg | ABRE | ACGTG | 969 | 979 | abscisic acid response element |
| SiMAPK20-3 | SETIT_021560mg | ABRE | GCAACGTGTC | 1703 | 1721 | abscisic acid response element |
| SiMAPK20-3 | SETIT_021560mg | ABRE | ACGTG | 1710 | 1720 | abscisic acid response element |
| SiMAPK20-3 | SETIT_021560mg | A-box | CCGTCC | 1757 | 1769 | cis-acting regulatory element |
| SiMAPK20-3 | SETIT_021560mg | G-Box | TCCACATGGCA | 1219 | 1239 | light responsive element |
| SiMAPK20-3 | SETIT_021560mg | G-Box | CACGTT | 1708 | 1720 | light responsive element |
| SiMAPK20-3 | SETIT_021560mg | G-box | CACGAC | 934 | 946 | light responsive element |
| SiMAPK20-3 | SETIT_021560mg | G-box | TACGTG | 968 | 980 | light responsive element |
| SiMAPK20-3 | SETIT_021560mg | TGACG-motif | TGACG | 912 | 922 | MeJA response regulatory element |
| SiMAPK20-3 | SETIT_021560mg | TGACG-motif | TGACG | 1018 | 1028 | MeJA response regulatory element |
| SiMAPK20-3 | SETIT_021560mg | TGACG-motif | TGACG | 1612 | 1622 | MeJA response regulatory element |
| SiMAPK20-3 | SETIT_021560mg | TGACG-motif | TGACG | 1991 | 2001 | MeJA response regulatory element |
| SiMAPK20-3 | SETIT_021560mg | CGTCA-motif | CGTCA | 912 | 922 | MeJA response regulatory element |
| SiMAPK20-3 | SETIT_021560mg | CGTCA-motif | CGTCA | 1018 | 1028 | MeJA response regulatory element |
| SiMAPK20-3 | SETIT_021560mg | CGTCA-motif | CGTCA | 1612 | 1622 | MeJA response regulatory element |
| SiMAPK20-3 | SETIT_021560mg | CGTCA-motif | CGTCA | 1991 | 2001 | MeJA response regulatory element |
| SiMAPK20-3 | SETIT_021560mg | CAAT-box | CAAAT | 20 | 30 | common cis-acting element |
| SiMAPK20-3 | SETIT_021560mg | CAAT-box | CAAAT | 192 | 202 | common cis-acting element |
| SiMAPK20-3 | SETIT_021560mg | CAAT-box | CCAAT | 214 | 224 | common cis-acting element |
| SiMAPK20-3 | SETIT_021560mg | CAAT-box | CCAAT | 387 | 397 | common cis-acting element |
| SiMAPK20-3 | SETIT_021560mg | CAAT-box | CCAAT | 465 | 475 | common cis-acting element |
| SiMAPK20-3 | SETIT_021560mg | CAAT-box | CAAAT | 566 | 576 | common cis-acting element |
| SiMAPK20-3 | SETIT_021560mg | CAAT-box | CCAAT | 574 | 584 | common cis-acting element |
| SiMAPK20-3 | SETIT_021560mg | CAAT-box | CCAAT | 610 | 620 | common cis-acting element |
| SiMAPK20-3 | SETIT_021560mg | CAAT-box | CAAAT | 640 | 650 | common cis-acting element |
| SiMAPK20-3 | SETIT_021560mg | CAAT-box | CAAAT | 1061 | 1071 | common cis-acting element |
| SiMAPK20-3 | SETIT_021560mg | CAAT-box | CAAAT | 1673 | 1683 | common cis-acting element |
| SiMAPK20-3 | SETIT_021560mg | CAAT-box | CCAAT | 1940 | 1950 | common cis-acting element |
| SiMAPK20-3 | SETIT_021560mg | TATA-box | TATA | 29 | 37 | core promoter element |
| SiMAPK20-3 | SETIT_021560mg | TATA-box | TACAAAA | 103 | 117 | core promoter element |
| SiMAPK20-3 | SETIT_021560mg | TATA-box | ATATAA | 400 | 412 | core promoter element |
| SiMAPK20-3 | SETIT_021560mg | TATA-box | TATA | 403 | 411 | core promoter element |
| SiMAPK20-3 | SETIT_021560mg | TATA-box | TATACA | 733 | 745 | core promoter element |
| SiMAPK20-3 | SETIT_021560mg | TATA-box | TATA | 737 | 745 | core promoter element |
| SiMAPK20-3 | SETIT_021560mg | TATA-box | TATA | 797 | 805 | core promoter element |
| SiMAPK20-3 | SETIT_021560mg | TATA-box | TATA | 974 | 982 | core promoter element |
| SiMAPK20-3 | SETIT_021560mg | TATA-box | TATATA | 1245 | 1257 | core promoter element |
| SiMAPK20-3 | SETIT_021560mg | TATA-box | ATATAA | 1246 | 1258 | core promoter element |
| SiMAPK20-3 | SETIT_021560mg | TATA-box | TATA | 1249 | 1257 | core promoter element |
| SiMAPK20-3 | SETIT_021560mg | TATA-box | ATATAA | 1841 | 1853 | core promoter element |
| SiMAPK20-3 | SETIT_021560mg | TATA-box | TATA | 1844 | 1852 | core promoter element |
| SiMAPK20-3 | SETIT_021560mg | GC-motif | CCCCCG | 1821 | 1833 | hypoxia-specifically induced elements |
| SiMAPK20-3 | SETIT_021560mg | GARE-motif | TCTGTTG | 253 | 267 | gibberellin-responsive element |
| SiMAPK20-3 | SETIT_021560mg | Sp1 | GGGCGG | 1403 | 1415 | light responsive element |
| SiMAPK20-3 | SETIT_021560mg | CCAAT-box | CAACGG | 1051 | 1063 | MYBHv1 binding site |
| SiMAPK20-3 | SETIT_021560mg | Box 4 | ATTAAT | 555 | 567 | light responsive element |
| SiMAPK20-3 | SETIT_021560mg | I-box | GTATAAGGCC | 731 | 749 | light responsive element |
| SiMAPK20-3 | SETIT_021560mg | TCT-motif | TCTTAC | 1188 | 1200 | light responsive element |
| SiMAPK20-3 | SETIT_021560mg | GATA-motif | AAGATAAGATT | 144 | 164 | light responsive element |
| SiMAPK20-3 | SETIT_021560mg | GATA-motif | GATAGGA | 1630 | 1644 | light responsive element |
| SiMAPK20-3 | SETIT_021560mg | chs-CMA2a | TCACTTGA | 316 | 332 | light responsive element |
| SiMAPK21-1 | SETIT_021565mg | TCA-element | CCATCTTTTT | 1288 | 1306 | salicylic acid response element |
| SiMAPK21-1 | SETIT_021565mg | ABRE | ACGTG | 379 | 389 | abscisic acid response element |
| SiMAPK21-1 | SETIT_021565mg | ABRE | ACGTG | 522 | 532 | abscisic acid response element |
| SiMAPK21-1 | SETIT_021565mg | ABRE | ACGTG | 926 | 936 | abscisic acid response element |
| SiMAPK21-1 | SETIT_021565mg | ABRE | TACGGTC | 1191 | 1205 | abscisic acid response element |
| SiMAPK21-1 | SETIT_021565mg | ABRE | TACGGTC | 1548 | 1562 | abscisic acid response element |
| SiMAPK21-1 | SETIT_021565mg | ABRE | ACGTG | 1673 | 1683 | abscisic acid response element |
| SiMAPK21-1 | SETIT_021565mg | A-box | CCGTCC | 602 | 614 | cis-acting regulatory element |
| SiMAPK21-1 | SETIT_021565mg | A-box | CCGTCC | 1702 | 1714 | cis-acting regulatory element |
| SiMAPK21-1 | SETIT_021565mg | A-box | CCGTCC | 1903 | 1915 | cis-acting regulatory element |
| SiMAPK21-1 | SETIT_021565mg | G-box | CACGTC | 378 | 390 | light responsive element |
| SiMAPK21-1 | SETIT_021565mg | G-box | CACGTC | 520 | 532 | light responsive element |
| SiMAPK21-1 | SETIT_021565mg | G-box | CACGTC | 1671 | 1683 | light responsive element |
| SiMAPK21-1 | SETIT_021565mg | G-Box | CACGTT | 924 | 936 | light responsive element |
| SiMAPK21-1 | SETIT_021565mg | CGTCA-motif | CGTCA | 520 | 530 | MeJA response regulatory element |
| SiMAPK21-1 | SETIT_021565mg | CGTCA-motif | CGTCA | 929 | 939 | MeJA response regulatory element |
| SiMAPK21-1 | SETIT_021565mg | CGTCA-motif | CGTCA | 1189 | 1199 | MeJA response regulatory element |
| SiMAPK21-1 | SETIT_021565mg | TGACG-motif | TGACG | 520 | 530 | MeJA response regulatory element |
| SiMAPK21-1 | SETIT_021565mg | TGACG-motif | TGACG | 929 | 939 | MeJA response regulatory element |
| SiMAPK21-1 | SETIT_021565mg | TGACG-motif | TGACG | 1189 | 1199 | MeJA response regulatory element |
| SiMAPK21-1 | SETIT_021565mg | CAT-box | GCCACT | 1838 | 1850 | meristem expression regulatory element |
| SiMAPK21-1 | SETIT_021565mg | CAAT-box | CCAAT | 105 | 115 | common cis-acting element |
| SiMAPK21-1 | SETIT_021565mg | CAAT-box | CAAAT | 108 | 118 | common cis-acting element |
| SiMAPK21-1 | SETIT_021565mg | CAAT-box | CAAAT | 168 | 178 | common cis-acting element |
| SiMAPK21-1 | SETIT_021565mg | CAAT-box | CAAAT | 195 | 205 | common cis-acting element |
| SiMAPK21-1 | SETIT_021565mg | CAAT-box | CCAAT | 292 | 302 | common cis-acting element |
| SiMAPK21-1 | SETIT_021565mg | CAAT-box | CAAAT | 354 | 364 | common cis-acting element |
| SiMAPK21-1 | SETIT_021565mg | CAAT-box | CCAAT | 482 | 492 | common cis-acting element |
| SiMAPK21-1 | SETIT_021565mg | CAAT-box | CCAAT | 669 | 679 | common cis-acting element |
| SiMAPK21-1 | SETIT_021565mg | CAAT-box | CCAAT | 918 | 928 | common cis-acting element |
| SiMAPK21-1 | SETIT_021565mg | CAAT-box | TGCCAAC | 973 | 987 | common cis-acting element |
| SiMAPK21-1 | SETIT_021565mg | CAAT-box | CCAAT | 1056 | 1066 | common cis-acting element |
| SiMAPK21-1 | SETIT_021565mg | CAAT-box | CAAAT | 1283 | 1293 | common cis-acting element |
| SiMAPK21-1 | SETIT_021565mg | CAAT-box | CAAAT | 1420 | 1430 | common cis-acting element |
| SiMAPK21-1 | SETIT_021565mg | CAAT-box | CAAAT | 1576 | 1586 | common cis-acting element |
| SiMAPK21-1 | SETIT_021565mg | CAAT-box | CAAAT | 1579 | 1589 | common cis-acting element |
| SiMAPK21-1 | SETIT_021565mg | CAAT-box | CCAAT | 1693 | 1703 | common cis-acting element |
| SiMAPK21-1 | SETIT_021565mg | TATA-box | TATAAAA | 242 | 256 | core promoter element |
| SiMAPK21-1 | SETIT_021565mg | TATA-box | TATAAA | 244 | 256 | core promoter element |
| SiMAPK21-1 | SETIT_021565mg | TATA-box | TATAA | 246 | 256 | core promoter element |
| SiMAPK21-1 | SETIT_021565mg | TATA-box | TATA | 248 | 256 | core promoter element |
| SiMAPK21-1 | SETIT_021565mg | TATA-box | TATAAAA | 303 | 317 | core promoter element |
| SiMAPK21-1 | SETIT_021565mg | TATA-box | TATAAA | 305 | 317 | core promoter element |
| SiMAPK21-1 | SETIT_021565mg | TATA-box | TATAA | 307 | 317 | core promoter element |
| SiMAPK21-1 | SETIT_021565mg | TATA-box | TATA | 309 | 317 | core promoter element |
| SiMAPK21-1 | SETIT_021565mg | TATA-box | ATATAA | 321 | 333 | core promoter element |
| SiMAPK21-1 | SETIT_021565mg | TATA-box | TATA | 324 | 332 | core promoter element |
| SiMAPK21-1 | SETIT_021565mg | TATA-box | ATATAA | 326 | 338 | core promoter element |
| SiMAPK21-1 | SETIT_021565mg | TATA-box | TATA | 329 | 337 | core promoter element |
| SiMAPK21-1 | SETIT_021565mg | TATA-box | TATAAAA | 1170 | 1184 | core promoter element |
| SiMAPK21-1 | SETIT_021565mg | TATA-box | TATAAA | 1172 | 1184 | core promoter element |
| SiMAPK21-1 | SETIT_021565mg | TATA-box | TATATAA | 1172 | 1186 | core promoter element |
| SiMAPK21-1 | SETIT_021565mg | TATA-box | TATATA | 1174 | 1186 | core promoter element |
| SiMAPK21-1 | SETIT_021565mg | TATA-box | ATATAA | 1175 | 1187 | core promoter element |
| SiMAPK21-1 | SETIT_021565mg | TATA-box | TATA | 1178 | 1186 | core promoter element |
| SiMAPK21-1 | SETIT_021565mg | TATA-box | TATACA | 1285 | 1297 | core promoter element |
| SiMAPK21-1 | SETIT_021565mg | TATA-box | TATA | 1289 | 1297 | core promoter element |
| SiMAPK21-1 | SETIT_021565mg | TATA-box | TATATA | 1367 | 1379 | core promoter element |
| SiMAPK21-1 | SETIT_021565mg | TATA-box | TATA | 1371 | 1379 | core promoter element |
| SiMAPK21-1 | SETIT_021565mg | TATA-box | TATAAAT | 1396 | 1410 | core promoter element |
| SiMAPK21-1 | SETIT_021565mg | TATA-box | TATAAA | 1398 | 1410 | core promoter element |
| SiMAPK21-1 | SETIT_021565mg | TATA-box | TATAA | 1400 | 1410 | core promoter element |
| SiMAPK21-1 | SETIT_021565mg | TATA-box | TATA | 1402 | 1410 | core promoter element |
| SiMAPK21-1 | SETIT_021565mg | TATA-box | ATATAT | 1470 | 1482 | core promoter element |
| SiMAPK21-1 | SETIT_021565mg | TATA-box | TATA | 1473 | 1481 | core promoter element |
| SiMAPK21-1 | SETIT_021565mg | GC-motif | CCCCCG | 898 | 910 | hypoxia-specifically induced elements |
| SiMAPK21-1 | SETIT_021565mg | GC-motif | CCCCCG | 1770 | 1782 | hypoxia-specifically induced elements |
| SiMAPK21-1 | SETIT_021565mg | GC-motif | CCCCCG | 1845 | 1857 | hypoxia-specifically induced elements |
| SiMAPK21-1 | SETIT_021565mg | GC-motif | CCCCCG | 1911 | 1923 | hypoxia-specifically induced elements |
| SiMAPK21-1 | SETIT_021565mg | GT1-motif | GGTTAA | 69 | 81 | light responsive element |
| SiMAPK21-1 | SETIT_021565mg | GT1-motif | GGTTAA | 82 | 94 | light responsive element |
| SiMAPK21-1 | SETIT_021565mg | GT1-motif | GGTTAAT | 1535 | 1549 | light responsive element |
| SiMAPK21-1 | SETIT_021565mg | Sp1 | GGGCGG | 1780 | 1792 | light responsive element |
| SiMAPK21-1 | SETIT_021565mg | MBS | CAACTG | 454 | 466 | MYB binding site involved in drought-inducibility |
| SiMAPK21-1 | SETIT_021565mg | MBS | CAACTG | 972 | 984 | MYB binding site involved in drought-inducibility |
| SiMAPK21-1 | SETIT_021565mg | TCT-motif | TCTTAC | 171 | 183 | light responsive element |
| SiMAPK21-1 | SETIT_021565mg | TCT-motif | TCTTAC | 491 | 503 | light responsive element |
| SiMAPK21-1 | SETIT_021565mg | I-box | ccttatcct | 1131 | 1149 | light responsive element |
| SiMAPK21-1 | SETIT_021565mg | chs-CMA1a | TTACTTAA | 1372 | 1388 | light responsive element |
| SiMAPK21-1 | SETIT_021565mg | GATA-motif | AAGGATAAGG | 1130 | 1148 | light responsive element |
| SiMAPK21-1 | SETIT_021565mg | GATA-motif | GATAGGA | 1223 | 1237 | light responsive element |
| SiMAPK21-1 | SETIT_021565mg | AE-box | AGAAACAA | 409 | 425 | light responsive element |
| SiMAPK21-2 | SETIT_004793mg | TGA-element | AACGAC | 779 | 791 | auxin-responsive element |
| SiMAPK21-2 | SETIT_004793mg | TATC-box | TATCCCA | 62 | 76 | gibberellin-responsive element |
| SiMAPK21-2 | SETIT_004793mg | TATC-box | TATCCCA | 319 | 333 | gibberellin-responsive element |
| SiMAPK21-2 | SETIT_004793mg | TCA-element | CCATCTTTTT | 64 | 82 | salicylic acid response element |
| SiMAPK21-2 | SETIT_004793mg | ABRE | CGCACGTGTC | 1615 | 1633 | abscisic acid response element |
| SiMAPK21-2 | SETIT_004793mg | ABRE | CACGTG | 1620 | 1632 | abscisic acid response element |
| SiMAPK21-2 | SETIT_004793mg | ABRE | ACGTG | 1622 | 1632 | abscisic acid response element |
| SiMAPK21-2 | SETIT_004793mg | ABRE | ACGTG | 1652 | 1662 | abscisic acid response element |
| SiMAPK21-2 | SETIT_004793mg | ABRE | CGCACGTGTC | 1927 | 1945 | abscisic acid response element |
| SiMAPK21-2 | SETIT_004793mg | ABRE | CACGTG | 1932 | 1944 | abscisic acid response element |
| SiMAPK21-2 | SETIT_004793mg | ABRE | ACGTG | 1934 | 1944 | abscisic acid response element |
| SiMAPK21-2 | SETIT_004793mg | A-box | CCGTCC | 801 | 813 | cis-acting regulatory element |
| SiMAPK21-2 | SETIT_004793mg | A-box | CCGTCC | 1974 | 1986 | cis-acting regulatory element |
| SiMAPK21-2 | SETIT_004793mg | ARE | AAACCA | 197 | 209 | anaerobic inducing element |
| SiMAPK21-2 | SETIT_004793mg | G-box | CACGAC | 338 | 350 | light responsive element |
| SiMAPK21-2 | SETIT_004793mg | G-box | CACGTG | 1620 | 1632 | light responsive element |
| SiMAPK21-2 | SETIT_004793mg | G-box | TACGTG | 1651 | 1663 | light responsive element |
| SiMAPK21-2 | SETIT_004793mg | G-box | CACGTG | 1932 | 1944 | light responsive element |
| SiMAPK21-2 | SETIT_004793mg | G-box | GCCACGTGGA | 1974 | 1992 | light responsive element |
| SiMAPK21-2 | SETIT_004793mg | G-Box | CACGTG | 1620 | 1632 | light responsive element |
| SiMAPK21-2 | SETIT_004793mg | G-Box | CACGTG | 1932 | 1944 | light responsive element |
| SiMAPK21-2 | SETIT_004793mg | RY-element | CATGCATG | 1439 | 1455 | elements involved in seed-specific regulation |
| SiMAPK21-2 | SETIT_004793mg | RY-element | CATGCATG | 1443 | 1459 | elements involved in seed-specific regulation |
| SiMAPK21-2 | SETIT_004793mg | TGACG-motif | TGACG | 123 | 133 | MeJA response regulatory element |
| SiMAPK21-2 | SETIT_004793mg | TGACG-motif | TGACG | 1729 | 1739 | MeJA response regulatory element |
| SiMAPK21-2 | SETIT_004793mg | CGTCA-motif | CGTCA | 123 | 133 | MeJA response regulatory element |
| SiMAPK21-2 | SETIT_004793mg | CGTCA-motif | CGTCA | 1729 | 1739 | MeJA response regulatory element |
| SiMAPK21-2 | SETIT_004793mg | CAT-box | GCCACT | 1815 | 1827 | meristem expression regulatory element |
| SiMAPK21-2 | SETIT_004793mg | CAAT-box | CCAAT | 268 | 278 | common cis-acting element |
| SiMAPK21-2 | SETIT_004793mg | CAAT-box | CCAAT | 283 | 293 | common cis-acting element |
| SiMAPK21-2 | SETIT_004793mg | CAAT-box | CAAAT | 352 | 362 | common cis-acting element |
| SiMAPK21-2 | SETIT_004793mg | CAAT-box | CAAAT | 540 | 550 | common cis-acting element |
| SiMAPK21-2 | SETIT_004793mg | CAAT-box | CAAAT | 560 | 570 | common cis-acting element |
| SiMAPK21-2 | SETIT_004793mg | CAAT-box | CCAAT | 628 | 638 | common cis-acting element |
| SiMAPK21-2 | SETIT_004793mg | CAAT-box | CAAAT | 637 | 647 | common cis-acting element |
| SiMAPK21-2 | SETIT_004793mg | CAAT-box | CAAAT | 1044 | 1054 | common cis-acting element |
| SiMAPK21-2 | SETIT_004793mg | CAAT-box | CAAAT | 1130 | 1140 | common cis-acting element |
| SiMAPK21-2 | SETIT_004793mg | CAAT-box | CAAAT | 1165 | 1175 | common cis-acting element |
| SiMAPK21-2 | SETIT_004793mg | CAAT-box | CAAAT | 1186 | 1196 | common cis-acting element |
| SiMAPK21-2 | SETIT_004793mg | CAAT-box | CAAAT | 1383 | 1393 | common cis-acting element |
| SiMAPK21-2 | SETIT_004793mg | CAAT-box | CAAAT | 1396 | 1406 | common cis-acting element |
| SiMAPK21-2 | SETIT_004793mg | CAAT-box | TGCCAAC | 1926 | 1940 | common cis-acting element |
| SiMAPK21-2 | SETIT_004793mg | CAAT-box | CAAAT | 1958 | 1968 | common cis-acting element |
| SiMAPK21-2 | SETIT_004793mg | TATA-box | ATTATA | 23 | 35 | core promoter element |
| SiMAPK21-2 | SETIT_004793mg | TATA-box | TATAA | 25 | 35 | core promoter element |
| SiMAPK21-2 | SETIT_004793mg | TATA-box | TATA | 27 | 35 | core promoter element |
| SiMAPK21-2 | SETIT_004793mg | TATA-box | TATA | 141 | 149 | core promoter element |
| SiMAPK21-2 | SETIT_004793mg | TATA-box | TATA | 810 | 818 | core promoter element |
| SiMAPK21-2 | SETIT_004793mg | TATA-box | TATA | 1228 | 1236 | core promoter element |
| SiMAPK21-2 | SETIT_004793mg | TATA-box | TACAAAA | 1322 | 1336 | core promoter element |
| SiMAPK21-2 | SETIT_004793mg | TATA-box | ATATAT | 1334 | 1346 | core promoter element |
| SiMAPK21-2 | SETIT_004793mg | TATA-box | TATATA | 1335 | 1347 | core promoter element |
| SiMAPK21-2 | SETIT_004793mg | TATA-box | ATATAT | 1336 | 1348 | core promoter element |
| SiMAPK21-2 | SETIT_004793mg | TATA-box | TATA | 1339 | 1347 | core promoter element |
| SiMAPK21-2 | SETIT_004793mg | TATA-box | TATA | 1364 | 1372 | core promoter element |
| SiMAPK21-2 | SETIT_004793mg | TATA-box | TATA | 1369 | 1377 | core promoter element |
| SiMAPK21-2 | SETIT_004793mg | AT-rich sequence | TAAAATACT | 207 | 225 | element for maximal elicitor-mediated activation |
| SiMAPK21-2 | SETIT_004793mg | GC-motif | CCCCCG | 1788 | 1800 | hypoxia-specifically induced elements |
| SiMAPK21-2 | SETIT_004793mg | GC-motif | CCCCCG | 1843 | 1855 | hypoxia-specifically induced elements |
| SiMAPK21-2 | SETIT_004793mg | P-box | CCTTTTG | 668 | 682 | gibberellin-responsive element |
| SiMAPK21-2 | SETIT_004793mg | Sp1 | GGGCGG | 1664 | 1676 | light responsive element |
| SiMAPK21-2 | SETIT_004793mg | Sp1 | GGGCGG | 1767 | 1779 | light responsive element |
| SiMAPK21-2 | SETIT_004793mg | Sp1 | GGGCGG | 1846 | 1858 | light responsive element |
| SiMAPK21-2 | SETIT_004793mg | Box 4 | ATTAAT | 1090 | 1102 | light responsive element |
| SiMAPK21-2 | SETIT_004793mg | Box 4 | ATTAAT | 1315 | 1327 | light responsive element |
| SiMAPK21-2 | SETIT_004793mg | AT1-motif | AATTATTTTTTATT | 1311 | 1337 | light responsive element |
| SiMAPK21-2 | SETIT_004793mg | AE-box | AGAAACAA | 917 | 933 | light responsive element |
| SiMAPK21-2 | SETIT_004793mg | AE-box | AGAAACTT | 1143 | 1159 | light responsive element |
| SiMAPKK1 | SETIT_006786mg | TGA-element | AACGAC | 979 | 991 | auxin-responsive element |
| SiMAPKK1 | SETIT_006786mg | LTR | CCGAAA | 1750 | 1762 | low temperature response element |
| SiMAPKK1 | SETIT_006786mg | Unnamed_1 | GGATTTTACAGT | 1110 | 1132 | phytochrome down-regulated expression element |
| SiMAPKK1 | SETIT_006786mg | ABRE | ACGTG | 788 | 798 | abscisic acid response element |
| SiMAPKK1 | SETIT_006786mg | ABRE | ACGTG | 973 | 983 | abscisic acid response element |
| SiMAPKK1 | SETIT_006786mg | ABRE | ACGTG | 1287 | 1297 | abscisic acid response element |
| SiMAPKK1 | SETIT_006786mg | ABRE | CACGTG | 1357 | 1369 | abscisic acid response element |
| SiMAPKK1 | SETIT_006786mg | ABRE | ACGTG | 1359 | 1369 | abscisic acid response element |
| SiMAPKK1 | SETIT_006786mg | ABRE | ACGTG | 1644 | 1654 | abscisic acid response element |
| SiMAPKK1 | SETIT_006786mg | ABRE | CGCACGTGTC | 1709 | 1727 | abscisic acid response element |
| SiMAPKK1 | SETIT_006786mg | ABRE | CACGTG | 1714 | 1726 | abscisic acid response element |
| SiMAPKK1 | SETIT_006786mg | ABRE | ACGTG | 1716 | 1726 | abscisic acid response element |
| SiMAPKK1 | SETIT_006786mg | A-box | CCGTCC | 465 | 477 | cis-acting regulatory element |
| SiMAPKK1 | SETIT_006786mg | A-box | CCGTCC | 1262 | 1274 | cis-acting regulatory element |
| SiMAPKK1 | SETIT_006786mg | ARE | AAACCA | 909 | 921 | anaerobic inducing element |
| SiMAPKK1 | SETIT_006786mg | ARE | AAACCA | 1419 | 1431 | anaerobic inducing element |
| SiMAPKK1 | SETIT_006786mg | G-box | CACGAC | 482 | 494 | light responsive element |
| SiMAPKK1 | SETIT_006786mg | G-box | TACGTG | 787 | 799 | light responsive element |
| SiMAPKK1 | SETIT_006786mg | G-box | TACGTG | 971 | 983 | light responsive element |
| SiMAPKK1 | SETIT_006786mg | G-box | GCCACGTGGA | 1281 | 1299 | light responsive element |
| SiMAPKK1 | SETIT_006786mg | G-box | CACGTG | 1357 | 1369 | light responsive element |
| SiMAPKK1 | SETIT_006786mg | G-box | TACGTG | 1643 | 1655 | light responsive element |
| SiMAPKK1 | SETIT_006786mg | G-box | CACGTG | 1714 | 1726 | light responsive element |
| SiMAPKK1 | SETIT_006786mg | G-Box | CACGTT | 1286 | 1298 | light responsive element |
| SiMAPKK1 | SETIT_006786mg | G-Box | CACGTG | 1357 | 1369 | light responsive element |
| SiMAPKK1 | SETIT_006786mg | G-Box | CACGTG | 1714 | 1726 | light responsive element |
| SiMAPKK1 | SETIT_006786mg | TGACG-motif | TGACG | 126 | 136 | MeJA response regulatory element |
| SiMAPKK1 | SETIT_006786mg | TGACG-motif | TGACG | 200 | 210 | MeJA response regulatory element |
| SiMAPKK1 | SETIT_006786mg | TGACG-motif | TGACG | 342 | 352 | MeJA response regulatory element |
| SiMAPKK1 | SETIT_006786mg | TGACG-motif | TGACG | 519 | 529 | MeJA response regulatory element |
| SiMAPKK1 | SETIT_006786mg | TGACG-motif | TGACG | 710 | 720 | MeJA response regulatory element |
| SiMAPKK1 | SETIT_006786mg | TGACG-motif | TGACG | 757 | 767 | MeJA response regulatory element |
| SiMAPKK1 | SETIT_006786mg | TGACG-motif | TGACG | 999 | 1009 | MeJA response regulatory element |
| SiMAPKK1 | SETIT_006786mg | TGACG-motif | TGACG | 1922 | 1932 | MeJA response regulatory element |
| SiMAPKK1 | SETIT_006786mg | CGTCA-motif | CGTCA | 126 | 136 | MeJA response regulatory element |
| SiMAPKK1 | SETIT_006786mg | CGTCA-motif | CGTCA | 200 | 210 | MeJA response regulatory element |
| SiMAPKK1 | SETIT_006786mg | CGTCA-motif | CGTCA | 342 | 352 | MeJA response regulatory element |
| SiMAPKK1 | SETIT_006786mg | CGTCA-motif | CGTCA | 519 | 529 | MeJA response regulatory element |
| SiMAPKK1 | SETIT_006786mg | CGTCA-motif | CGTCA | 710 | 720 | MeJA response regulatory element |
| SiMAPKK1 | SETIT_006786mg | CGTCA-motif | CGTCA | 757 | 767 | MeJA response regulatory element |
| SiMAPKK1 | SETIT_006786mg | CGTCA-motif | CGTCA | 999 | 1009 | MeJA response regulatory element |
| SiMAPKK1 | SETIT_006786mg | CGTCA-motif | CGTCA | 1922 | 1932 | MeJA response regulatory element |
| SiMAPKK1 | SETIT_006786mg | CAAT-box | CCAAT | 191 | 201 | common cis-acting element |
| SiMAPKK1 | SETIT_006786mg | CAAT-box | CAAAT | 226 | 236 | common cis-acting element |
| SiMAPKK1 | SETIT_006786mg | CAAT-box | CAAAT | 641 | 651 | common cis-acting element |
| SiMAPKK1 | SETIT_006786mg | CAAT-box | CAAAT | 714 | 724 | common cis-acting element |
| SiMAPKK1 | SETIT_006786mg | CAAT-box | CCAAT | 935 | 945 | common cis-acting element |
| SiMAPKK1 | SETIT_006786mg | CAAT-box | CAAAT | 951 | 961 | common cis-acting element |
| SiMAPKK1 | SETIT_006786mg | CAAT-box | CAAAT | 1145 | 1155 | common cis-acting element |
| SiMAPKK1 | SETIT_006786mg | CAAT-box | CCAAT | 1159 | 1169 | common cis-acting element |
| SiMAPKK1 | SETIT_006786mg | CAAT-box | CCAAT | 1238 | 1248 | common cis-acting element |
| SiMAPKK1 | SETIT_006786mg | CAAT-box | CCAAT | 1349 | 1359 | common cis-acting element |
| SiMAPKK1 | SETIT_006786mg | CAAT-box | CCAAT | 1371 | 1381 | common cis-acting element |
| SiMAPKK1 | SETIT_006786mg | CAAT-box | CAAAT | 1402 | 1412 | common cis-acting element |
| SiMAPKK1 | SETIT_006786mg | CAAT-box | CAAAT | 1562 | 1572 | common cis-acting element |
| SiMAPKK1 | SETIT_006786mg | CAAT-box | CAAAT | 1589 | 1599 | common cis-acting element |
| SiMAPKK1 | SETIT_006786mg | CAAT-box | CAAAT | 1787 | 1797 | common cis-acting element |
| SiMAPKK1 | SETIT_006786mg | CAAT-box | CCAAT | 1829 | 1839 | common cis-acting element |
| SiMAPKK1 | SETIT_006786mg | CAAT-box | TGCCAAC | 1840 | 1854 | common cis-acting element |
| SiMAPKK1 | SETIT_006786mg | TATA-box | ATATAT | 156 | 168 | core promoter element |
| SiMAPKK1 | SETIT_006786mg | TATA-box | TATA | 159 | 167 | core promoter element |
| SiMAPKK1 | SETIT_006786mg | TATA-box | TACAAAA | 1151 | 1165 | core promoter element |
| SiMAPKK1 | SETIT_006786mg | TATA-box | TATAAATA | 1488 | 1504 | core promoter element |
| SiMAPKK1 | SETIT_006786mg | TATA-box | TATAAAT | 1490 | 1504 | core promoter element |
| SiMAPKK1 | SETIT_006786mg | TATA-box | TATAAA | 1492 | 1504 | core promoter element |
| SiMAPKK1 | SETIT_006786mg | TATA-box | TATAA | 1494 | 1504 | core promoter element |
| SiMAPKK1 | SETIT_006786mg | TATA-box | TATA | 1496 | 1504 | core promoter element |
| SiMAPKK1 | SETIT_006786mg | TATA-box | TACAAAA | 1657 | 1671 | core promoter element |
| SiMAPKK1 | SETIT_006786mg | GC-motif | CCCCCG | 1941 | 1953 | hypoxia-specifically induced elements |
| SiMAPKK1 | SETIT_006786mg | P-box | CCTTTTG | 237 | 251 | gibberellin-responsive element |
| SiMAPKK1 | SETIT_006786mg | P-box | CCTTTTG | 1057 | 1071 | gibberellin-responsive element |
| SiMAPKK1 | SETIT_006786mg | P-box | CCTTTTG | 1807 | 1821 | gibberellin-responsive element |
| SiMAPKK1 | SETIT_006786mg | GT1-motif | GGTTAA | 583 | 595 | light responsive element |
| SiMAPKK1 | SETIT_006786mg | Sp1 | GGGCGG | 676 | 688 | light responsive element |
| SiMAPKK1 | SETIT_006786mg | Box 4 | ATTAAT | 1603 | 1615 | light responsive element |
| SiMAPKK1 | SETIT_006786mg | GTGGC-motif | CATCGTGTGGC | 692 | 712 | light responsive element |
| SiMAPKK1 | SETIT_006786mg | TCCC-motif | TCTCCCT | 875 | 889 | light responsive element |
| SiMAPKK1 | SETIT_006786mg | I-box | TGATAATGT | 1561 | 1579 | light responsive element |
| SiMAPKK3-1 | SETIT_015676mg | TC-rich repeats | GTTTTCTTAC | 1530 | 1548 | defense and stress response elements |
| SiMAPKK3-1 | SETIT_015676mg | ACE | GCGACGTACC | 1286 | 1304 | light responsive element |
| SiMAPKK3-1 | SETIT_015676mg | TCA-element | CCATCTTTTT | 1382 | 1400 | salicylic acid response element |
| SiMAPKK3-1 | SETIT_015676mg | ABRE | ACGTG | 461 | 471 | abscisic acid response element |
| SiMAPKK3-1 | SETIT_015676mg | ABRE | ACGTG | 1088 | 1098 | abscisic acid response element |
| SiMAPKK3-1 | SETIT_015676mg | ABRE | ACGTG | 1237 | 1247 | abscisic acid response element |
| SiMAPKK3-1 | SETIT_015676mg | ABRE | ACGTG | 1326 | 1336 | abscisic acid response element |
| SiMAPKK3-1 | SETIT_015676mg | ABRE | ACGTG | 1369 | 1379 | abscisic acid response element |
| SiMAPKK3-1 | SETIT_015676mg | A-box | CCGTCC | 210 | 222 | cis-acting regulatory element |
| SiMAPKK3-1 | SETIT_015676mg | G-Box | CACGTT | 1235 | 1247 | light responsive element |
| SiMAPKK3-1 | SETIT_015676mg | G-box | TACGTG | 460 | 472 | light responsive element |
| SiMAPKK3-1 | SETIT_015676mg | G-box | TACGTG | 1087 | 1099 | light responsive element |
| SiMAPKK3-1 | SETIT_015676mg | G-box | CACGTC | 1324 | 1336 | light responsive element |
| SiMAPKK3-1 | SETIT_015676mg | G-box | CACGTC | 1368 | 1380 | light responsive element |
| SiMAPKK3-1 | SETIT_015676mg | CGTCA-motif | CGTCA | 263 | 273 | MeJA response regulatory element |
| SiMAPKK3-1 | SETIT_015676mg | CGTCA-motif | CGTCA | 931 | 941 | MeJA response regulatory element |
| SiMAPKK3-1 | SETIT_015676mg | CGTCA-motif | CGTCA | 1137 | 1147 | MeJA response regulatory element |
| SiMAPKK3-1 | SETIT_015676mg | CGTCA-motif | CGTCA | 1172 | 1182 | MeJA response regulatory element |
| SiMAPKK3-1 | SETIT_015676mg | CGTCA-motif | CGTCA | 1255 | 1265 | MeJA response regulatory element |
| SiMAPKK3-1 | SETIT_015676mg | CGTCA-motif | CGTCA | 1321 | 1331 | MeJA response regulatory element |
| SiMAPKK3-1 | SETIT_015676mg | TGACG-motif | TGACG | 263 | 273 | MeJA response regulatory element |
| SiMAPKK3-1 | SETIT_015676mg | TGACG-motif | TGACG | 931 | 941 | MeJA response regulatory element |
| SiMAPKK3-1 | SETIT_015676mg | TGACG-motif | TGACG | 1137 | 1147 | MeJA response regulatory element |
| SiMAPKK3-1 | SETIT_015676mg | TGACG-motif | TGACG | 1172 | 1182 | MeJA response regulatory element |
| SiMAPKK3-1 | SETIT_015676mg | TGACG-motif | TGACG | 1255 | 1265 | MeJA response regulatory element |
| SiMAPKK3-1 | SETIT_015676mg | TGACG-motif | TGACG | 1321 | 1331 | MeJA response regulatory element |
| SiMAPKK3-1 | SETIT_015676mg | CAT-box | GCCACT | 1949 | 1961 | meristem expression regulatory element |
| SiMAPKK3-1 | SETIT_015676mg | GCN4_motif | TGAGTCA | 540 | 554 | endosperm expression regulatory element |
| SiMAPKK3-1 | SETIT_015676mg | CAAT-box | CCAAT | 279 | 289 | common cis-acting element |
| SiMAPKK3-1 | SETIT_015676mg | CAAT-box | CAAAT | 409 | 419 | common cis-acting element |
| SiMAPKK3-1 | SETIT_015676mg | CAAT-box | CCAAT | 631 | 641 | common cis-acting element |
| SiMAPKK3-1 | SETIT_015676mg | CAAT-box | CCAAT | 779 | 789 | common cis-acting element |
| SiMAPKK3-1 | SETIT_015676mg | CAAT-box | CAAAT | 1007 | 1017 | common cis-acting element |
| SiMAPKK3-1 | SETIT_015676mg | CAAT-box | CAAAT | 1283 | 1293 | common cis-acting element |
| SiMAPKK3-1 | SETIT_015676mg | CAAT-box | CCAAT | 1419 | 1429 | common cis-acting element |
| SiMAPKK3-1 | SETIT_015676mg | CAAT-box | CCAAT | 1437 | 1447 | common cis-acting element |
| SiMAPKK3-1 | SETIT_015676mg | CAAT-box | CAAAT | 1471 | 1481 | common cis-acting element |
| SiMAPKK3-1 | SETIT_015676mg | CAAT-box | CCAAT | 1702 | 1712 | common cis-acting element |
| SiMAPKK3-1 | SETIT_015676mg | CAAT-box | CAAAT | 1873 | 1883 | common cis-acting element |
| SiMAPKK3-1 | SETIT_015676mg | TATA-box | ATATAT | 689 | 701 | core promoter element |
| SiMAPKK3-1 | SETIT_015676mg | TATA-box | TATA | 692 | 700 | core promoter element |
| SiMAPKK3-1 | SETIT_015676mg | TATA-box | ATATAT | 1577 | 1589 | core promoter element |
| SiMAPKK3-1 | SETIT_015676mg | TATA-box | TATATA | 1578 | 1590 | core promoter element |
| SiMAPKK3-1 | SETIT_015676mg | TATA-box | TATA | 1582 | 1590 | core promoter element |
| SiMAPKK3-1 | SETIT_015676mg | TATA-box | TACAAAA | 1641 | 1655 | core promoter element |
| SiMAPKK3-1 | SETIT_015676mg | TATA-box | TATA | 1681 | 1689 | core promoter element |
| SiMAPKK3-1 | SETIT_015676mg | TATA-box | ATATAA | 1721 | 1733 | core promoter element |
| SiMAPKK3-1 | SETIT_015676mg | TATA-box | TATA | 1724 | 1732 | core promoter element |
| SiMAPKK3-1 | SETIT_015676mg | TATA-box | TATA | 1772 | 1780 | core promoter element |
| SiMAPKK3-1 | SETIT_015676mg | TATA-box | ATATAT | 1842 | 1854 | core promoter element |
| SiMAPKK3-1 | SETIT_015676mg | TATA-box | TATATA | 1843 | 1855 | core promoter element |
| SiMAPKK3-1 | SETIT_015676mg | TATA-box | TATA | 1847 | 1855 | core promoter element |
| SiMAPKK3-1 | SETIT_015676mg | GC-motif | CCCCCG | 1340 | 1352 | hypoxia-specifically induced elements |
| SiMAPKK3-1 | SETIT_015676mg | P-box | CCTTTTG | 1796 | 1810 | gibberellin-responsive element |
| SiMAPKK3-1 | SETIT_015676mg | GARE-motif | TCTGTTG | 1158 | 1172 | gibberellin-responsive element |
| SiMAPKK3-1 | SETIT_015676mg | Sp1 | GGGCGG | 64 | 76 | light responsive element |
| SiMAPKK3-1 | SETIT_015676mg | Sp1 | GGGCGG | 1141 | 1153 | light responsive element |
| SiMAPKK3-1 | SETIT_015676mg | 3-AF1 binding site | TAAGAGAGGAA | 116 | 136 | light responsive element |
| SiMAPKK3-1 | SETIT_015676mg | GT1-motif | GGTTAA | 1961 | 1973 | light responsive element |
| SiMAPKK3-1 | SETIT_015676mg | MBS | CAACTG | 671 | 683 | MYB binding site involved in drought-inducibility |
| SiMAPKK3-1 | SETIT_015676mg | CCAAT-box | CAACGG | 661 | 673 | MYBHv1 binding site |
| SiMAPKK3-1 | SETIT_015676mg | ATCT-motif | AATCTAATCC | 624 | 642 | light responsive element |
| SiMAPKK3-1 | SETIT_015676mg | ATC-motif | AGTAATCT | 320 | 336 | light responsive element |
| SiMAPKK3-1 | SETIT_015676mg | TCT-motif | TCTTAC | 126 | 138 | light responsive element |
| SiMAPKK3-1 | SETIT_015676mg | TCT-motif | TCTTAC | 786 | 798 | light responsive element |
| SiMAPKK3-1 | SETIT_015676mg | TCT-motif | TCTTAC | 1447 | 1459 | light responsive element |
| SiMAPKK3-1 | SETIT_015676mg | TCCC-motif | TCTCCCT | 117 | 131 | light responsive element |
| SiMAPKK3-1 | SETIT_015676mg | GTGGC-motif | CATCGTGTGGC | 1110 | 1130 | light responsive element |
| SiMAPKK3-1 | SETIT_015676mg | AE-box | AGAAACAA | 841 | 857 | light responsive element |
| SiMAPKK3-2 | SETIT_016904mg | TCA-element | CCATCTTTTT | 49 | 69 | salicylic acid response element |
| SiMAPKK3-2 | SETIT_016904mg | ABRE | ACGTG | 7 | 17 | abscisic acid response element |
| SiMAPKK3-2 | SETIT_016904mg | ABRE | ACGTG | 1155 | 1165 | abscisic acid response element |
| SiMAPKK3-2 | SETIT_016904mg | ABRE | AACCCGG | 1953 | 1967 | abscisic acid response element |
| SiMAPKK3-2 | SETIT_016904mg | A-box | CCGTCC | 264 | 276 | cis-acting regulatory element |
| SiMAPKK3-2 | SETIT_016904mg | A-box | CCGTCC | 477 | 489 | cis-acting regulatory element |
| SiMAPKK3-2 | SETIT_016904mg | A-box | CCGTCC | 1229 | 1241 | cis-acting regulatory element |
| SiMAPKK3-2 | SETIT_016904mg | A-box | CCGTCC | 1572 | 1584 | cis-acting regulatory element |
| SiMAPKK3-2 | SETIT_016904mg | G-Box | CACGTT | 1153 | 1165 | light responsive element |
| SiMAPKK3-2 | SETIT_016904mg | G-box | TACGTG | 6 | 18 | light responsive element |
| SiMAPKK3-2 | SETIT_016904mg | CGTCA-motif | CGTCA | 1128 | 1138 | MeJA response regulatory element |
| SiMAPKK3-2 | SETIT_016904mg | CGTCA-motif | CGTCA | 1665 | 1675 | MeJA response regulatory element |
| SiMAPKK3-2 | SETIT_016904mg | TGACG-motif | TGACG | 1128 | 1138 | MeJA response regulatory element |
| SiMAPKK3-2 | SETIT_016904mg | TGACG-motif | TGACG | 1665 | 1675 | MeJA response regulatory element |
| SiMAPKK3-2 | SETIT_016904mg | O2-site | GATGATGTGG | 307 | 325 | Zein metabolism regulatory elements |
| SiMAPKK3-2 | SETIT_016904mg | O2-site | GATGACATGG | 623 | 641 | Zein metabolism regulatory elements |
| SiMAPKK3-2 | SETIT_016904mg | CAAT-box | TGCCAAC | 135 | 149 | common cis-acting element |
| SiMAPKK3-2 | SETIT_016904mg | CAAT-box | CAAAT | 142 | 152 | common cis-acting element |
| SiMAPKK3-2 | SETIT_016904mg | CAAT-box | CAAAT | 401 | 411 | common cis-acting element |
| SiMAPKK3-2 | SETIT_016904mg | CAAT-box | CAAAT | 442 | 452 | common cis-acting element |
| SiMAPKK3-2 | SETIT_016904mg | CAAT-box | CAAAT | 539 | 549 | common cis-acting element |
| SiMAPKK3-2 | SETIT_016904mg | CAAT-box | CAAAT | 1037 | 1047 | common cis-acting element |
| SiMAPKK3-2 | SETIT_016904mg | CAAT-box | TGCAAATCT | 1072 | 1090 | common cis-acting element |
| SiMAPKK3-2 | SETIT_016904mg | CAAT-box | CAAAT | 1078 | 1088 | common cis-acting element |
| SiMAPKK3-2 | SETIT_016904mg | TATA-box | TATA | 240 | 248 | core promoter element |
| SiMAPKK3-2 | SETIT_016904mg | TATA-box | TATA | 254 | 262 | core promoter element |
| SiMAPKK3-2 | SETIT_016904mg | TATA-box | TATAA | 275 | 285 | core promoter element |
| SiMAPKK3-2 | SETIT_016904mg | TATA-box | TATA | 277 | 285 | core promoter element |
| SiMAPKK3-2 | SETIT_016904mg | TATA-box | TATA | 349 | 357 | core promoter element |
| SiMAPKK3-2 | SETIT_016904mg | TATA-box | TATACA | 357 | 369 | core promoter element |
| SiMAPKK3-2 | SETIT_016904mg | TATA-box | TATA | 361 | 369 | core promoter element |
| SiMAPKK3-2 | SETIT_016904mg | TATA-box | TATA | 373 | 381 | core promoter element |
| SiMAPKK3-2 | SETIT_016904mg | TATA-box | TACAAAA | 504 | 518 | core promoter element |
| SiMAPKK3-2 | SETIT_016904mg | TATA-box | TATACA | 619 | 631 | core promoter element |
| SiMAPKK3-2 | SETIT_016904mg | TATA-box | TATA | 623 | 631 | core promoter element |
| SiMAPKK3-2 | SETIT_016904mg | TATA-box | TATACA | 649 | 661 | core promoter element |
| SiMAPKK3-2 | SETIT_016904mg | TATA-box | TATA | 653 | 661 | core promoter element |
| SiMAPKK3-2 | SETIT_016904mg | TATA-box | TATA | 745 | 753 | core promoter element |
| SiMAPKK3-2 | SETIT_016904mg | TATA-box | ccTATAAAaa | 764 | 782 | core promoter element |
| SiMAPKK3-2 | SETIT_016904mg | TATA-box | ATTATA | 877 | 889 | core promoter element |
| SiMAPKK3-2 | SETIT_016904mg | TATA-box | TATAA | 879 | 889 | core promoter element |
| SiMAPKK3-2 | SETIT_016904mg | TATA-box | TATA | 881 | 889 | core promoter element |
| SiMAPKK3-2 | SETIT_016904mg | TATA-box | TATA | 1690 | 1698 | core promoter element |
| SiMAPKK3-2 | SETIT_016904mg | GC-motif | CCCCCG | 1353 | 1365 | hypoxia-specifically induced elements |
| SiMAPKK3-2 | SETIT_016904mg | Sp1 | GGGCGG | 1273 | 1285 | light responsive element |
| SiMAPKK3-2 | SETIT_016904mg | Sp1 | GGGCGG | 1302 | 1314 | light responsive element |
| SiMAPKK3-2 | SETIT_016904mg | Sp1 | GGGCGG | 1336 | 1348 | light responsive element |
| SiMAPKK3-2 | SETIT_016904mg | Sp1 | GGGCGG | 1356 | 1368 | light responsive element |
| SiMAPKK3-2 | SETIT_016904mg | Sp1 | GGGCGG | 1372 | 1384 | light responsive element |
| SiMAPKK3-2 | SETIT_016904mg | Sp1 | GGGCGG | 1401 | 1413 | light responsive element |
| SiMAPKK3-2 | SETIT_016904mg | Sp1 | GGGCGG | 1460 | 1472 | light responsive element |
| SiMAPKK3-2 | SETIT_016904mg | Sp1 | GGGCGG | 1477 | 1489 | light responsive element |
| SiMAPKK3-2 | SETIT_016904mg | MBS | CAACTG | 32 | 44 | MYB binding site involved in drought-inducibility |
| SiMAPKK3-2 | SETIT_016904mg | GATT-motif | CTCCTGATTAGC | 844 | 866 | light responsive element |
| SiMAPKK3-2 | SETIT_016904mg | TCT-motif | TCTTAC | 1590 | 1602 | light responsive element |
| SiMAPKK3-2 | SETIT_016904mg | TGA-box | TGACGTAA | 1122 | 1138 | part of an auxin-responsive element |
| SiMAPKK4-1 | SETIT_002102mg | TC-rich repeats | ATTCTCTAAC | 513 | 531 | defense and stress response elements |
| SiMAPKK4-1 | SETIT_002102mg | TC-rich repeats | ATTCTCTAAC | 1010 | 1028 | defense and stress response elements |
| SiMAPKK4-1 | SETIT_002102mg | ACE | GCGACGTACC | 255 | 273 | light responsive element |
| SiMAPKK4-1 | SETIT_002102mg | LTR | CCGAAA | 1817 | 1829 | low temperature response element |
| SiMAPKK4-1 | SETIT_002102mg | TCA-element | CCATCTTTTT | 805 | 823 | salicylic acid response element |
| SiMAPKK4-1 | SETIT_002102mg | ABRE | CGTACGTGCA | 479 | 497 | abscisic acid response element |
| SiMAPKK4-1 | SETIT_002102mg | ABRE | ACGTG | 485 | 495 | abscisic acid response element |
| SiMAPKK4-1 | SETIT_002102mg | ABRE | AACCCGG | 668 | 682 | abscisic acid response element |
| SiMAPKK4-1 | SETIT_002102mg | ABRE | ACGTG | 695 | 705 | abscisic acid response element |
| SiMAPKK4-1 | SETIT_002102mg | ABRE | ACGTG | 1476 | 1486 | abscisic acid response element |
| SiMAPKK4-1 | SETIT_002102mg | ABRE | ACGTG | 1891 | 1901 | abscisic acid response element |
| SiMAPKK4-1 | SETIT_002102mg | A-box | CCGTCC | 327 | 339 | cis-acting regulatory element |
| SiMAPKK4-1 | SETIT_002102mg | A-box | CCGTCC | 419 | 431 | cis-acting regulatory element |
| SiMAPKK4-1 | SETIT_002102mg | A-box | CCGTCC | 1964 | 1976 | cis-acting regulatory element |
| SiMAPKK4-1 | SETIT_002102mg | ARE | AAACCA | 876 | 888 | anaerobic inducing element |
| SiMAPKK4-1 | SETIT_002102mg | G-box | CACGAC | 92 | 104 | light responsive element |
| SiMAPKK4-1 | SETIT_002102mg | G-box | TACGTG | 484 | 496 | light responsive element |
| SiMAPKK4-1 | SETIT_002102mg | G-box | CACGAC | 653 | 665 | light responsive element |
| SiMAPKK4-1 | SETIT_002102mg | G-box | CACGTC | 693 | 705 | light responsive element |
| SiMAPKK4-1 | SETIT_002102mg | G-box | TACGTG | 1475 | 1487 | light responsive element |
| SiMAPKK4-1 | SETIT_002102mg | G-Box | CACGTT | 1889 | 1901 | light responsive element |
| SiMAPKK4-1 | SETIT_002102mg | TGACG-motif | TGACG | 194 | 204 | MeJA response regulatory element |
| SiMAPKK4-1 | SETIT_002102mg | TGACG-motif | TGACG | 208 | 218 | MeJA response regulatory element |
| SiMAPKK4-1 | SETIT_002102mg | TGACG-motif | TGACG | 1772 | 1782 | MeJA response regulatory element |
| SiMAPKK4-1 | SETIT_002102mg | TGACG-motif | TGACG | 1779 | 1789 | MeJA response regulatory element |
| SiMAPKK4-1 | SETIT_002102mg | TGACG-motif | TGACG | 1800 | 1810 | MeJA response regulatory element |
| SiMAPKK4-1 | SETIT_002102mg | CGTCA-motif | CGTCA | 194 | 204 | MeJA response regulatory element |
| SiMAPKK4-1 | SETIT_002102mg | CGTCA-motif | CGTCA | 208 | 218 | MeJA response regulatory element |
| SiMAPKK4-1 | SETIT_002102mg | CGTCA-motif | CGTCA | 1772 | 1782 | MeJA response regulatory element |
| SiMAPKK4-1 | SETIT_002102mg | CGTCA-motif | CGTCA | 1779 | 1789 | MeJA response regulatory element |
| SiMAPKK4-1 | SETIT_002102mg | CGTCA-motif | CGTCA | 1800 | 1810 | MeJA response regulatory element |
| SiMAPKK4-1 | SETIT_002102mg | CAT-box | GCCACT | 1531 | 1543 | meristem expression regulatory element |
| SiMAPKK4-1 | SETIT_002102mg | GCN4_motif | TGAGTCA | 627 | 641 | endosperm expression regulatory element |
| SiMAPKK4-1 | SETIT_002102mg | CAAT-box | CCAAT | 88 | 98 | common cis-acting element |
| SiMAPKK4-1 | SETIT_002102mg | CAAT-box | CCAAT | 578 | 588 | common cis-acting element |
| SiMAPKK4-1 | SETIT_002102mg | CAAT-box | CCAAT | 1022 | 1032 | common cis-acting element |
| SiMAPKK4-1 | SETIT_002102mg | CAAT-box | CCAAT | 1260 | 1270 | common cis-acting element |
| SiMAPKK4-1 | SETIT_002102mg | CAAT-box | CCAAT | 1435 | 1445 | common cis-acting element |
| SiMAPKK4-1 | SETIT_002102mg | CAAT-box | CAAAT | 1584 | 1594 | common cis-acting element |
| SiMAPKK4-1 | SETIT_002102mg | TATA-box | TATATA | 1050 | 1062 | core promoter element |
| SiMAPKK4-1 | SETIT_002102mg | TATA-box | TATA | 1054 | 1062 | core promoter element |
| SiMAPKK4-1 | SETIT_002102mg | TATA-box | ccTATAAAaa | 1115 | 1133 | core promoter element |
| SiMAPKK4-1 | SETIT_002102mg | TATA-box | TACATAAA | 1178 | 1194 | core promoter element |
| SiMAPKK4-1 | SETIT_002102mg | TATA-box | TACATAAA | 1195 | 1211 | core promoter element |
| SiMAPKK4-1 | SETIT_002102mg | TATA-box | TATAA | 1556 | 1566 | core promoter element |
| SiMAPKK4-1 | SETIT_002102mg | TATA-box | TATA | 1558 | 1566 | core promoter element |
| SiMAPKK4-1 | SETIT_002102mg | TATA-box | TATAAA | 1656 | 1668 | core promoter element |
| SiMAPKK4-1 | SETIT_002102mg | TATA-box | TATAA | 1658 | 1668 | core promoter element |
| SiMAPKK4-1 | SETIT_002102mg | TATA-box | TATA | 1660 | 1668 | core promoter element |
| SiMAPKK4-1 | SETIT_002102mg | TATA-box | ATATAT | 1692 | 1704 | core promoter element |
| SiMAPKK4-1 | SETIT_002102mg | TATA-box | TATA | 1695 | 1703 | core promoter element |
| SiMAPKK4-1 | SETIT_002102mg | GC-motif | CCCCCG | 315 | 327 | hypoxia-specifically induced elements |
| SiMAPKK4-1 | SETIT_002102mg | Sp1 | GGGCGG | 1391 | 1403 | light responsive element |
| SiMAPKK4-1 | SETIT_002102mg | Sp1 | GGGCGG | 1645 | 1657 | light responsive element |
| SiMAPKK4-1 | SETIT_002102mg | ATCT-motif | AATCTAATCC | 1299 | 1317 | light responsive element |
| SiMAPKK4-1 | SETIT_002102mg | CAG-motif | GAAAGGCAGAC | 627 | 647 | light responsive element |
| SiMAPKK4-1 | SETIT_002102mg | CAG-motif | GAAAGGCAGAC | 1477 | 1497 | light responsive element |
| SiMAPKK4-1 | SETIT_002102mg | GATA-motif | GATAGGA | 676 | 690 | light responsive element |
| SiMAPKK4-1 | SETIT_002102mg | GATA-motif | AAGGATAAGG | 1109 | 1127 | light responsive element |
| SiMAPKK4-1 | SETIT_002102mg | AE-box | AGAAACTT | 762 | 778 | light responsive element |
| SiMAPKK4-1 | SETIT_002102mg | AE-box | AGAAACAA | 769 | 785 | light responsive element |
| SiMAPKK4-2 | SETIT_017572mg | TGA-element | AACGAC | 1085 | 1097 | auxin-responsive element |
| SiMAPKK4-2 | SETIT_017572mg | AT-rich element | ATAGAAATCAA | 474 | 494 | ATBP-1 binding site |
| SiMAPKK4-2 | SETIT_017572mg | TC-rich repeats | GTTTTCTTAC | 452 | 470 | defense and stress response elements |
| SiMAPKK4-2 | SETIT_017572mg | ACE | CTAACGTATT | 1209 | 1227 | light responsive element |
| SiMAPKK4-2 | SETIT_017572mg | SARE | TTCGACCATCTT | 1498 | 1520 | salicylic acid response element |
| SiMAPKK4-2 | SETIT_017572mg | ABRE | ACGTG | 389 | 399 | abscisic acid response element |
| SiMAPKK4-2 | SETIT_017572mg | ABRE | CACGTG | 502 | 514 | abscisic acid response element |
| SiMAPKK4-2 | SETIT_017572mg | ABRE | ACGTG | 504 | 514 | abscisic acid response element |
| SiMAPKK4-2 | SETIT_017572mg | ABRE | TACGGTC | 1363 | 1377 | abscisic acid response element |
| SiMAPKK4-2 | SETIT_017572mg | ABRE | ACGTG | 1725 | 1735 | abscisic acid response element |
| SiMAPKK4-2 | SETIT_017572mg | ABRE | ACGTG | 1856 | 1866 | abscisic acid response element |
| SiMAPKK4-2 | SETIT_017572mg | A-box | CCGTCC | 1894 | 1906 | cis-acting regulatory element |
| SiMAPKK4-2 | SETIT_017572mg | ARE | AAACCA | 1530 | 1542 | anaerobic inducing element |
| SiMAPKK4-2 | SETIT_017572mg | G-Box | CACGTG | 502 | 514 | light responsive element |
| SiMAPKK4-2 | SETIT_017572mg | G-Box | CACGTT | 1854 | 1866 | light responsive element |
| SiMAPKK4-2 | SETIT_017572mg | G-box | CACGTC | 387 | 399 | light responsive element |
| SiMAPKK4-2 | SETIT_017572mg | G-box | CACGTG | 502 | 514 | light responsive element |
| SiMAPKK4-2 | SETIT_017572mg | G-box | CACGTC | 1724 | 1736 | light responsive element |
| SiMAPKK4-2 | SETIT_017572mg | CGTCA-motif | CGTCA | 387 | 397 | MeJA response regulatory element |
| SiMAPKK4-2 | SETIT_017572mg | CGTCA-motif | CGTCA | 963 | 973 | MeJA response regulatory element |
| SiMAPKK4-2 | SETIT_017572mg | CGTCA-motif | CGTCA | 974 | 984 | MeJA response regulatory element |
| SiMAPKK4-2 | SETIT_017572mg | CGTCA-motif | CGTCA | 1499 | 1509 | MeJA response regulatory element |
| SiMAPKK4-2 | SETIT_017572mg | CGTCA-motif | CGTCA | 1859 | 1869 | MeJA response regulatory element |
| SiMAPKK4-2 | SETIT_017572mg | TGACG-motif | TGACG | 387 | 397 | MeJA response regulatory element |
| SiMAPKK4-2 | SETIT_017572mg | TGACG-motif | TGACG | 963 | 973 | MeJA response regulatory element |
| SiMAPKK4-2 | SETIT_017572mg | TGACG-motif | TGACG | 974 | 984 | MeJA response regulatory element |
| SiMAPKK4-2 | SETIT_017572mg | TGACG-motif | TGACG | 1499 | 1509 | MeJA response regulatory element |
| SiMAPKK4-2 | SETIT_017572mg | TGACG-motif | TGACG | 1859 | 1869 | MeJA response regulatory element |
| SiMAPKK4-2 | SETIT_017572mg | GCN4_motif | TGAGTCA | 1316 | 1330 | endosperm expression regulatory element |
| SiMAPKK4-2 | SETIT_017572mg | CAAT-box | CAAAT | 30 | 40 | common cis-acting element |
| SiMAPKK4-2 | SETIT_017572mg | CAAT-box | CCAAT | 278 | 288 | common cis-acting element |
| SiMAPKK4-2 | SETIT_017572mg | CAAT-box | CAAAT | 404 | 414 | common cis-acting element |
| SiMAPKK4-2 | SETIT_017572mg | CAAT-box | CCAAT | 425 | 435 | common cis-acting element |
| SiMAPKK4-2 | SETIT_017572mg | CAAT-box | CAAAT | 639 | 649 | common cis-acting element |
| SiMAPKK4-2 | SETIT_017572mg | CAAT-box | CAAAT | 812 | 822 | common cis-acting element |
| SiMAPKK4-2 | SETIT_017572mg | CAAT-box | CAAAT | 885 | 895 | common cis-acting element |
| SiMAPKK4-2 | SETIT_017572mg | CAAT-box | CCAAT | 982 | 992 | common cis-acting element |
| SiMAPKK4-2 | SETIT_017572mg | CAAT-box | CAAAT | 1156 | 1166 | common cis-acting element |
| SiMAPKK4-2 | SETIT_017572mg | CAAT-box | CAAAT | 1211 | 1221 | common cis-acting element |
| SiMAPKK4-2 | SETIT_017572mg | CAAT-box | CCAAT | 1873 | 1883 | common cis-acting element |
| SiMAPKK4-2 | SETIT_017572mg | TATA-box | ATTATA | 70 | 82 | core promoter element |
| SiMAPKK4-2 | SETIT_017572mg | TATA-box | TATAA | 72 | 82 | core promoter element |
| SiMAPKK4-2 | SETIT_017572mg | TATA-box | TATA | 74 | 82 | core promoter element |
| SiMAPKK4-2 | SETIT_017572mg | TATA-box | TACAAAA | 93 | 107 | core promoter element |
| SiMAPKK4-2 | SETIT_017572mg | TATA-box | ccTATAAAaa | 156 | 174 | core promoter element |
| SiMAPKK4-2 | SETIT_017572mg | TATA-box | ATTATA | 240 | 252 | core promoter element |
| SiMAPKK4-2 | SETIT_017572mg | TATA-box | TATAA | 242 | 252 | core promoter element |
| SiMAPKK4-2 | SETIT_017572mg | TATA-box | TATA | 244 | 252 | core promoter element |
| SiMAPKK4-2 | SETIT_017572mg | TATA-box | TATA | 334 | 342 | core promoter element |
| SiMAPKK4-2 | SETIT_017572mg | TATA-box | TATAAAA | 341 | 355 | core promoter element |
| SiMAPKK4-2 | SETIT_017572mg | TATA-box | TATAAA | 343 | 355 | core promoter element |
| SiMAPKK4-2 | SETIT_017572mg | TATA-box | TATAA | 345 | 355 | core promoter element |
| SiMAPKK4-2 | SETIT_017572mg | TATA-box | TATA | 347 | 355 | core promoter element |
| SiMAPKK4-2 | SETIT_017572mg | TATA-box | TATA | 488 | 496 | core promoter element |
| SiMAPKK4-2 | SETIT_017572mg | TATA-box | TATACA | 582 | 594 | core promoter element |
| SiMAPKK4-2 | SETIT_017572mg | TATA-box | TATA | 586 | 594 | core promoter element |
| SiMAPKK4-2 | SETIT_017572mg | TATA-box | ATATAT | 591 | 603 | core promoter element |
| SiMAPKK4-2 | SETIT_017572mg | TATA-box | TATATA | 592 | 604 | core promoter element |
| SiMAPKK4-2 | SETIT_017572mg | TATA-box | ATATAT | 593 | 605 | core promoter element |
| SiMAPKK4-2 | SETIT_017572mg | TATA-box | TATATA | 594 | 606 | core promoter element |
| SiMAPKK4-2 | SETIT_017572mg | TATA-box | TATA | 598 | 606 | core promoter element |
| SiMAPKK4-2 | SETIT_017572mg | TATA-box | TATA | 610 | 618 | core promoter element |
| SiMAPKK4-2 | SETIT_017572mg | TATA-box | ATATAT | 631 | 643 | core promoter element |
| SiMAPKK4-2 | SETIT_017572mg | TATA-box | TATATA | 632 | 644 | core promoter element |
| SiMAPKK4-2 | SETIT_017572mg | TATA-box | ATATAA | 633 | 645 | core promoter element |
| SiMAPKK4-2 | SETIT_017572mg | TATA-box | TATA | 636 | 644 | core promoter element |
| SiMAPKK4-2 | SETIT_017572mg | TATA-box | TATA | 704 | 712 | core promoter element |
| SiMAPKK4-2 | SETIT_017572mg | TATA-box | ATATAT | 709 | 721 | core promoter element |
| SiMAPKK4-2 | SETIT_017572mg | TATA-box | TATATA | 710 | 722 | core promoter element |
| SiMAPKK4-2 | SETIT_017572mg | TATA-box | ATATAT | 711 | 723 | core promoter element |
| SiMAPKK4-2 | SETIT_017572mg | TATA-box | TATATA | 712 | 724 | core promoter element |
| SiMAPKK4-2 | SETIT_017572mg | TATA-box | TATA | 716 | 724 | core promoter element |
| SiMAPKK4-2 | SETIT_017572mg | TATA-box | TATA | 728 | 736 | core promoter element |
| SiMAPKK4-2 | SETIT_017572mg | TATA-box | ATATAT | 749 | 761 | core promoter element |
| SiMAPKK4-2 | SETIT_017572mg | TATA-box | TATATA | 750 | 762 | core promoter element |
| SiMAPKK4-2 | SETIT_017572mg | TATA-box | ATATAA | 751 | 763 | core promoter element |
| SiMAPKK4-2 | SETIT_017572mg | TATA-box | TATA | 754 | 762 | core promoter element |
| SiMAPKK4-2 | SETIT_017572mg | TATA-box | TATAA | 890 | 900 | core promoter element |
| SiMAPKK4-2 | SETIT_017572mg | TATA-box | TATA | 892 | 900 | core promoter element |
| SiMAPKK4-2 | SETIT_017572mg | TATA-box | ATTATA | 1323 | 1335 | core promoter element |
| SiMAPKK4-2 | SETIT_017572mg | TATA-box | TATAA | 1325 | 1335 | core promoter element |
| SiMAPKK4-2 | SETIT_017572mg | TATA-box | TATA | 1327 | 1335 | core promoter element |
| SiMAPKK4-2 | SETIT_017572mg | TATA-box | TATA | 1460 | 1468 | core promoter element |
| SiMAPKK4-2 | SETIT_017572mg | TATA-box | TATAAA | 1533 | 1545 | core promoter element |
| SiMAPKK4-2 | SETIT_017572mg | TATA-box | TATAA | 1535 | 1545 | core promoter element |
| SiMAPKK4-2 | SETIT_017572mg | TATA-box | TATA | 1537 | 1545 | core promoter element |
| SiMAPKK4-2 | SETIT_017572mg | TATA-box | TATA | 1573 | 1581 | core promoter element |
| SiMAPKK4-2 | SETIT_017572mg | TATA-box | TATA | 1599 | 1607 | core promoter element |
| SiMAPKK4-2 | SETIT_017572mg | TATA-box | ccTATAAAaa | 1768 | 1786 | core promoter element |
| SiMAPKK4-2 | SETIT_017572mg | TATA-box | TATA | 1775 | 1783 | core promoter element |
| SiMAPKK4-2 | SETIT_017572mg | HD-Zip 1 | CAAT(A/T)ATTG | 469.5 | 486.5 | elements of palisade mesophyll cell differentiation |
| SiMAPKK4-2 | SETIT_017572mg | P-box | CCTTTTG | 1704 | 1718 | gibberellin-responsive element |
| SiMAPKK4-2 | SETIT_017572mg | GT1-motif | GGTTAA | 351 | 363 | light responsive element |
| SiMAPKK4-2 | SETIT_017572mg | GATA-motif | AAGATAAGATT | 448 | 468 | light responsive element |
| SiMAPKK4-2 | SETIT_017572mg | GATA-motif | GATAGGA | 1518 | 1532 | light responsive element |
| SiMAPKK4-2 | SETIT_017572mg | GATT-motif | CTCCTGATTAGC | 112 | 134 | light responsive element |
| SiMAPKK4-2 | SETIT_017572mg | GATT-motif | CTCCTGATTGGA | 413 | 435 | light responsive element |
| SiMAPKK4-2 | SETIT_017572mg | TCT-motif | TCTTAC | 459 | 471 | light responsive element |
| SiMAPKK5 | SETIT_006813mg | ACE | GCGACGTACC | 1083 | 1101 | light responsive element |
| SiMAPKK5 | SETIT_006813mg | TCA-element | CCATCTTTTT | 45 | 65 | salicylic acid response element |
| SiMAPKK5 | SETIT_006813mg | TCA-element | CCATCTTTTT | 513 | 531 | salicylic acid response element |
| SiMAPKK5 | SETIT_006813mg | ABRE | ACGTG | 218 | 228 | abscisic acid response element |
| SiMAPKK5 | SETIT_006813mg | ABRE | CGCACGTGTC | 792 | 810 | abscisic acid response element |
| SiMAPKK5 | SETIT_006813mg | ABRE | CACGTG | 831 | 843 | abscisic acid response element |
| SiMAPKK5 | SETIT_006813mg | ABRE | ACGTG | 833 | 843 | abscisic acid response element |
| SiMAPKK5 | SETIT_006813mg | ABRE | GCAACGTGTC | 939 | 957 | abscisic acid response element |
| SiMAPKK5 | SETIT_006813mg | ABRE | ACGTG | 945 | 955 | abscisic acid response element |
| SiMAPKK5 | SETIT_006813mg | ABRE | CACGTG | 1011 | 1023 | abscisic acid response element |
| SiMAPKK5 | SETIT_006813mg | ABRE | ACGTG | 1013 | 1023 | abscisic acid response element |
| SiMAPKK5 | SETIT_006813mg | ABRE | ACGTG | 1145 | 1155 | abscisic acid response element |
| SiMAPKK5 | SETIT_006813mg | ABRE | CGCACGTGTC | 1163 | 1181 | abscisic acid response element |
| SiMAPKK5 | SETIT_006813mg | ABRE | CACGTG | 1168 | 1180 | abscisic acid response element |
| SiMAPKK5 | SETIT_006813mg | ABRE | ACGTG | 1170 | 1180 | abscisic acid response element |
| SiMAPKK5 | SETIT_006813mg | ABRE | ACGTG | 1568 | 1578 | abscisic acid response element |
| SiMAPKK5 | SETIT_006813mg | ABRE | ACGTG | 1597 | 1607 | abscisic acid response element |
| SiMAPKK5 | SETIT_006813mg | A-box | CCGTCC | 1735 | 1747 | cis-acting regulatory element |
| SiMAPKK5 | SETIT_006813mg | A-box | CCGTCC | 1837 | 1849 | cis-acting regulatory element |
| SiMAPKK5 | SETIT_006813mg | G-Box | CACGTG | 831 | 843 | light responsive element |
| SiMAPKK5 | SETIT_006813mg | G-Box | CACGTG | 1011 | 1023 | light responsive element |
| SiMAPKK5 | SETIT_006813mg | G-Box | CACGTG | 1168 | 1180 | light responsive element |
| SiMAPKK5 | SETIT_006813mg | G-Box | CACGTT | 1567 | 1579 | light responsive element |
| SiMAPKK5 | SETIT_006813mg | G-box | CACGTC | 216 | 228 | light responsive element |
| SiMAPKK5 | SETIT_006813mg | G-box | CACGTG | 831 | 843 | light responsive element |
| SiMAPKK5 | SETIT_006813mg | G-box | CACGTC | 944 | 956 | light responsive element |
| SiMAPKK5 | SETIT_006813mg | G-box | CACGTG | 1011 | 1023 | light responsive element |
| SiMAPKK5 | SETIT_006813mg | G-box | CACGTC | 1144 | 1156 | light responsive element |
| SiMAPKK5 | SETIT_006813mg | G-box | CACGTG | 1168 | 1180 | light responsive element |
| SiMAPKK5 | SETIT_006813mg | G-box | CACGAC | 1492 | 1504 | light responsive element |
| SiMAPKK5 | SETIT_006813mg | G-box | TACGTG | 1596 | 1608 | light responsive element |
| SiMAPKK5 | SETIT_006813mg | G-box | CACGAC | 1895 | 1907 | light responsive element |
| SiMAPKK5 | SETIT_006813mg | CGTCA-motif | CGTCA | 1430 | 1440 | MeJA response regulatory element |
| SiMAPKK5 | SETIT_006813mg | CGTCA-motif | CGTCA | 1626 | 1636 | MeJA response regulatory element |
| SiMAPKK5 | SETIT_006813mg | CGTCA-motif | CGTCA | 1793 | 1803 | MeJA response regulatory element |
| SiMAPKK5 | SETIT_006813mg | TGACG-motif | TGACG | 1430 | 1440 | MeJA response regulatory element |
| SiMAPKK5 | SETIT_006813mg | TGACG-motif | TGACG | 1626 | 1636 | MeJA response regulatory element |
| SiMAPKK5 | SETIT_006813mg | TGACG-motif | TGACG | 1793 | 1803 | MeJA response regulatory element |
| SiMAPKK5 | SETIT_006813mg | O2-site | GATGACATGG | 410 | 428 | Zein metabolism regulatory elements |
| SiMAPKK5 | SETIT_006813mg | CAT-box | GCCACT | 648 | 660 | meristem expression regulatory element |
| SiMAPKK5 | SETIT_006813mg | CAAT-box | CCAAT | 170 | 180 | common cis-acting element |
| SiMAPKK5 | SETIT_006813mg | CAAT-box | TGCCAAC | 188 | 202 | common cis-acting element |
| SiMAPKK5 | SETIT_006813mg | CAAT-box | CAAAT | 404 | 414 | common cis-acting element |
| SiMAPKK5 | SETIT_006813mg | CAAT-box | CAAAT | 1159 | 1169 | common cis-acting element |
| SiMAPKK5 | SETIT_006813mg | CAAT-box | CAAAT | 1420 | 1430 | common cis-acting element |
| SiMAPKK5 | SETIT_006813mg | CAAT-box | CCAAT | 1871 | 1881 | common cis-acting element |
| SiMAPKK5 | SETIT_006813mg | TATA-box | TATA | 263 | 271 | core promoter element |
| SiMAPKK5 | SETIT_006813mg | TATA-box | TATAA | 464 | 474 | core promoter element |
| SiMAPKK5 | SETIT_006813mg | TATA-box | TATA | 466 | 474 | core promoter element |
| SiMAPKK5 | SETIT_006813mg | TATA-box | TATAA | 863 | 873 | core promoter element |
| SiMAPKK5 | SETIT_006813mg | TATA-box | TATA | 865 | 873 | core promoter element |
| SiMAPKK5 | SETIT_006813mg | TATA-box | TATAAA | 891 | 903 | core promoter element |
| SiMAPKK5 | SETIT_006813mg | TATA-box | TATAA | 893 | 903 | core promoter element |
| SiMAPKK5 | SETIT_006813mg | TATA-box | TATA | 895 | 903 | core promoter element |
| SiMAPKK5 | SETIT_006813mg | TATA-box | ATATAT | 1041 | 1053 | core promoter element |
| SiMAPKK5 | SETIT_006813mg | TATA-box | TATA | 1044 | 1052 | core promoter element |
| SiMAPKK5 | SETIT_006813mg | TATA-box | TATA | 1256 | 1264 | core promoter element |
| SiMAPKK5 | SETIT_006813mg | TATA-box | ATTATA | 1257 | 1269 | core promoter element |
| SiMAPKK5 | SETIT_006813mg | TATA-box | TATAA | 1259 | 1269 | core promoter element |
| SiMAPKK5 | SETIT_006813mg | TATA-box | TATA | 1261 | 1269 | core promoter element |
| SiMAPKK5 | SETIT_006813mg | TATA-box | TATAA | 1390 | 1400 | core promoter element |
| SiMAPKK5 | SETIT_006813mg | TATA-box | TATA | 1392 | 1400 | core promoter element |
| SiMAPKK5 | SETIT_006813mg | TATA-box | TATA | 1411 | 1419 | core promoter element |
| SiMAPKK5 | SETIT_006813mg | AACA_motif | TAACAAACTCCA | 1961 | 1983 | involved in endosperm-specific negative expression |
| SiMAPKK5 | SETIT_006813mg | Sp1 | GGGCGG | 1983 | 1995 | light responsive element |
| SiMAPKK5 | SETIT_006813mg | GT1-motif | GGTTAAT | 314 | 328 | light responsive element |
| SiMAPKK5 | SETIT_006813mg | Box 4 | ATTAAT | 304 | 316 | light responsive element |
| SiMAPKK5 | SETIT_006813mg | TCT-motif | TCTTAC | 105 | 117 | light responsive element |
| SiMAPKK5 | SETIT_006813mg | AE-box | AGAAACAA | 95 | 111 | light responsive element |
| SiMAPKK5 | SETIT_006813mg | TGA-element | AACGAC | 635 | 647 | auxin-responsive element |
| SiMAPKK6-1 | SETIT_022487mg | TC-rich repeats | GTTTTCTTAC | 1289 | 1307 | defense and stress response elements |
| SiMAPKK6-1 | SETIT_022487mg | TC-rich repeats | GTTTTCTTAC | 1418 | 1436 | defense and stress response elements |
| SiMAPKK6-1 | SETIT_022487mg | TCA-element | CCATCTTTTT | 338 | 356 | salicylic acid response element |
| SiMAPKK6-1 | SETIT_022487mg | ABRE | ACGTG | 356 | 366 | abscisic acid response element |
| SiMAPKK6-1 | SETIT_022487mg | ABRE | ACGTG | 885 | 895 | abscisic acid response element |
| SiMAPKK6-1 | SETIT_022487mg | ABRE | CACGTG | 1008 | 1020 | abscisic acid response element |
| SiMAPKK6-1 | SETIT_022487mg | ABRE | ACGTG | 1010 | 1020 | abscisic acid response element |
| SiMAPKK6-1 | SETIT_022487mg | ABRE | ACGTG | 1449 | 1459 | abscisic acid response element |
| SiMAPKK6-1 | SETIT_022487mg | ABRE | CACGTG | 1732 | 1744 | abscisic acid response element |
| SiMAPKK6-1 | SETIT_022487mg | ABRE | ACGTG | 1734 | 1744 | abscisic acid response element |
| SiMAPKK6-1 | SETIT_022487mg | ABRE | TACGGTC | 1747 | 1761 | abscisic acid response element |
| SiMAPKK6-1 | SETIT_022487mg | A-box | CCGTCC | 1958 | 1970 | cis-acting regulatory element |
| SiMAPKK6-1 | SETIT_022487mg | G-Box | CACGTG | 1008 | 1020 | light responsive element |
| SiMAPKK6-1 | SETIT_022487mg | G-Box | CACGTG | 1732 | 1744 | light responsive element |
| SiMAPKK6-1 | SETIT_022487mg | G-box | TACGTG | 354 | 366 | light responsive element |
| SiMAPKK6-1 | SETIT_022487mg | G-box | CACGTC | 883 | 895 | light responsive element |
| SiMAPKK6-1 | SETIT_022487mg | G-box | CACGTG | 1008 | 1020 | light responsive element |
| SiMAPKK6-1 | SETIT_022487mg | G-box | TACGTG | 1447 | 1459 | light responsive element |
| SiMAPKK6-1 | SETIT_022487mg | G-box | CACGTG | 1732 | 1744 | light responsive element |
| SiMAPKK6-1 | SETIT_022487mg | RY-element | CATGCATG | 310 | 326 | elements involved in seed-specific regulation |
| SiMAPKK6-1 | SETIT_022487mg | TGACG-motif | TGACG | 493 | 503 | MeJA response regulatory element |
| SiMAPKK6-1 | SETIT_022487mg | TGACG-motif | TGACG | 1013 | 1023 | MeJA response regulatory element |
| SiMAPKK6-1 | SETIT_022487mg | CGTCA-motif | CGTCA | 493 | 503 | MeJA response regulatory element |
| SiMAPKK6-1 | SETIT_022487mg | CGTCA-motif | CGTCA | 1013 | 1023 | MeJA response regulatory element |
| SiMAPKK6-1 | SETIT_022487mg | O2-site | GATGACATGG | 577 | 595 | Zein metabolism regulatory elements |
| SiMAPKK6-1 | SETIT_022487mg | O2-site | GATGACATGG | 582 | 600 | Zein metabolism regulatory elements |
| SiMAPKK6-1 | SETIT_022487mg | O2-site | GATGA(C/T)(A/G)TG(A/G) | 700 | 716 | Zein metabolism regulatory elements |
| SiMAPKK6-1 | SETIT_022487mg | O2-site | GATGATGTGG | 822 | 842 | Zein metabolism regulatory elements |
| SiMAPKK6-1 | SETIT_022487mg | CAAT-box | CCAAT | 333 | 343 | common cis-acting element |
| SiMAPKK6-1 | SETIT_022487mg | CAAT-box | TGCAAATCT | 478 | 496 | common cis-acting element |
| SiMAPKK6-1 | SETIT_022487mg | CAAT-box | CAAAT | 484 | 494 | common cis-acting element |
| SiMAPKK6-1 | SETIT_022487mg | CAAT-box | CCAAT | 722 | 732 | common cis-acting element |
| SiMAPKK6-1 | SETIT_022487mg | CAAT-box | CCAAT | 1072 | 1082 | common cis-acting element |
| SiMAPKK6-1 | SETIT_022487mg | CAAT-box | CAAAT | 1103 | 1113 | common cis-acting element |
| SiMAPKK6-1 | SETIT_022487mg | CAAT-box | CAAAT | 1262 | 1272 | common cis-acting element |
| SiMAPKK6-1 | SETIT_022487mg | CAAT-box | CAAAT | 1280 | 1290 | common cis-acting element |
| SiMAPKK6-1 | SETIT_022487mg | CAAT-box | CAAAT | 1432 | 1442 | common cis-acting element |
| SiMAPKK6-1 | SETIT_022487mg | CAAT-box | CCAAT | 1543 | 1553 | common cis-acting element |
| SiMAPKK6-1 | SETIT_022487mg | CAAT-box | CAAAT | 1713 | 1723 | common cis-acting element |
| SiMAPKK6-1 | SETIT_022487mg | CAAT-box | CAAAT | 1768 | 1778 | common cis-acting element |
| SiMAPKK6-1 | SETIT_022487mg | TATA-box | TACATAAA | 346 | 362 | core promoter element |
| SiMAPKK6-1 | SETIT_022487mg | TATA-box | TATACA | 641 | 653 | core promoter element |
| SiMAPKK6-1 | SETIT_022487mg | TATA-box | TATA | 645 | 653 | core promoter element |
| SiMAPKK6-1 | SETIT_022487mg | TATA-box | TATA | 695 | 703 | core promoter element |
| SiMAPKK6-1 | SETIT_022487mg | TATA-box | TACAAAA | 1116 | 1130 | core promoter element |
| SiMAPKK6-1 | SETIT_022487mg | TATA-box | TACATAAA | 1468 | 1484 | core promoter element |
| SiMAPKK6-1 | SETIT_022487mg | TATA-box | TATACA | 1546 | 1558 | core promoter element |
| SiMAPKK6-1 | SETIT_022487mg | TATA-box | TATA | 1550 | 1558 | core promoter element |
| SiMAPKK6-1 | SETIT_022487mg | TATA-box | TATA | 1628 | 1636 | core promoter element |
| SiMAPKK6-1 | SETIT_022487mg | GC-motif | CCCCCG | 1899 | 1911 | hypoxia-specifically induced elements |
| SiMAPKK6-1 | SETIT_022487mg | GARE-motif | TCTGTTG | 1531 | 1545 | gibberellin-responsive element |
| SiMAPKK6-1 | SETIT_022487mg | Sp1 | GGGCGG | 31 | 43 | light responsive element |
| SiMAPKK6-1 | SETIT_022487mg | 3-AF1 binding site | TAAGAGAGGAA | 971 | 991 | light responsive element |
| SiMAPKK6-1 | SETIT_022487mg | MBS | CAACTG | 1644 | 1656 | MYB binding site involved in drought-inducibility |
| SiMAPKK6-1 | SETIT_022487mg | CCAAT-box | CAACGG | 226 | 238 | MYBHv1 binding site |
| SiMAPKK6-1 | SETIT_022487mg | I-box | cCATATCCAAT | 711 | 731 | light responsive element |
| SiMAPKK6-1 | SETIT_022487mg | TCCC-motif | TCTCCCT | 72 | 86 | light responsive element |
| SiMAPKK6-1 | SETIT_022487mg | TCT-motif | TCTTAC | 1296 | 1308 | light responsive element |
| SiMAPKK6-1 | SETIT_022487mg | TCT-motif | TCTTAC | 1421 | 1433 | light responsive element |
| SiMAPKK6-1 | SETIT_022487mg | AE-box | AGAAACAA | 388 | 404 | light responsive element |
| SiMAPKK6-2 | SETIT_002024mg | TGA-element | AACGAC | 212 | 224 | auxin-responsive element |
| SiMAPKK6-2 | SETIT_002024mg | LTR | CCGAAA | 1612 | 1624 | low temperature response element |
| SiMAPKK6-2 | SETIT_002024mg | ABRE | GCAACGTGTC | 43 | 61 | abscisic acid response element |
| SiMAPKK6-2 | SETIT_002024mg | ARE | AAACCA | 387 | 399 | anaerobic inducing element |
| SiMAPKK6-2 | SETIT_002024mg | ARE | AAACCA | 1096 | 1108 | anaerobic inducing element |
| SiMAPKK6-2 | SETIT_002024mg | CGTCA-motif | CGTCA | 1717 | 1727 | MeJA response regulatory element |
| SiMAPKK6-2 | SETIT_002024mg | CGTCA-motif | CGTCA | 1797 | 1807 | MeJA response regulatory element |
| SiMAPKK6-2 | SETIT_002024mg | TGACG-motif | TGACG | 1717 | 1727 | MeJA response regulatory element |
| SiMAPKK6-2 | SETIT_002024mg | TGACG-motif | TGACG | 1797 | 1807 | MeJA response regulatory element |
| SiMAPKK6-2 | SETIT_002024mg | CAT-box | GCCACT | 607 | 619 | meristem expression regulatory element |
| SiMAPKK6-2 | SETIT_002024mg | CAT-box | GCCACT | 721 | 733 | meristem expression regulatory element |
| SiMAPKK6-2 | SETIT_002024mg | CAAT-box | CAAAT | 249 | 259 | common cis-acting element |
| SiMAPKK6-2 | SETIT_002024mg | CAAT-box | CAAAT | 430 | 440 | common cis-acting element |
| SiMAPKK6-2 | SETIT_002024mg | CAAT-box | CAAAT | 447 | 457 | common cis-acting element |
| SiMAPKK6-2 | SETIT_002024mg | CAAT-box | CCAAT | 473 | 483 | common cis-acting element |
| SiMAPKK6-2 | SETIT_002024mg | CAAT-box | CCAAT | 623 | 633 | common cis-acting element |
| SiMAPKK6-2 | SETIT_002024mg | CAAT-box | CAAAT | 818 | 828 | common cis-acting element |
| SiMAPKK6-2 | SETIT_002024mg | CAAT-box | CCAAT | 893 | 903 | common cis-acting element |
| SiMAPKK6-2 | SETIT_002024mg | CAAT-box | CCAAT | 1137 | 1147 | common cis-acting element |
| SiMAPKK6-2 | SETIT_002024mg | CAAT-box | CAAAT | 1167 | 1177 | common cis-acting element |
| SiMAPKK6-2 | SETIT_002024mg | CAAT-box | CAAAT | 1431 | 1441 | common cis-acting element |
| SiMAPKK6-2 | SETIT_002024mg | CAAT-box | CAAAT | 1484 | 1494 | common cis-acting element |
| SiMAPKK6-2 | SETIT_002024mg | CAAT-box | CAAAT | 1589 | 1599 | common cis-acting element |
| SiMAPKK6-2 | SETIT_002024mg | CAAT-box | CAAAT | 1643 | 1653 | common cis-acting element |
| SiMAPKK6-2 | SETIT_002024mg | TATA-box | TATA | 127 | 135 | core promoter element |
| SiMAPKK6-2 | SETIT_002024mg | TATA-box | tcTATAAATAgg | 392 | 414 | core promoter element |
| SiMAPKK6-2 | SETIT_002024mg | TATA-box | TATA | 401 | 409 | core promoter element |
| SiMAPKK6-2 | SETIT_002024mg | TATA-box | ATTATA | 432 | 444 | core promoter element |
| SiMAPKK6-2 | SETIT_002024mg | TATA-box | TATAA | 434 | 444 | core promoter element |
| SiMAPKK6-2 | SETIT_002024mg | TATA-box | TATA | 436 | 444 | core promoter element |
| SiMAPKK6-2 | SETIT_002024mg | TATA-box | TATAAA | 507 | 519 | core promoter element |
| SiMAPKK6-2 | SETIT_002024mg | TATA-box | TATAA | 509 | 519 | core promoter element |
| SiMAPKK6-2 | SETIT_002024mg | TATA-box | TATA | 511 | 519 | core promoter element |
| SiMAPKK6-2 | SETIT_002024mg | TATA-box | TATATA | 527 | 539 | core promoter element |
| SiMAPKK6-2 | SETIT_002024mg | TATA-box | ATATAT | 528 | 540 | core promoter element |
| SiMAPKK6-2 | SETIT_002024mg | TATA-box | TATATA | 529 | 541 | core promoter element |
| SiMAPKK6-2 | SETIT_002024mg | TATA-box | TATA | 533 | 541 | core promoter element |
| SiMAPKK6-2 | SETIT_002024mg | TATA-box | TATA | 704 | 712 | core promoter element |
| SiMAPKK6-2 | SETIT_002024mg | TATA-box | TATA | 1027 | 1035 | core promoter element |
| SiMAPKK6-2 | SETIT_002024mg | TATA-box | TATAAGAA | 1209 | 1225 | core promoter element |
| SiMAPKK6-2 | SETIT_002024mg | TATA-box | TATAA | 1215 | 1225 | core promoter element |
| SiMAPKK6-2 | SETIT_002024mg | TATA-box | TATA | 1217 | 1225 | core promoter element |
| SiMAPKK6-2 | SETIT_002024mg | TATA-box | TATAAAA | 1222 | 1236 | core promoter element |
| SiMAPKK6-2 | SETIT_002024mg | TATA-box | TATAAA | 1224 | 1236 | core promoter element |
| SiMAPKK6-2 | SETIT_002024mg | TATA-box | TATAA | 1226 | 1236 | core promoter element |
| SiMAPKK6-2 | SETIT_002024mg | TATA-box | TATA | 1228 | 1236 | core promoter element |
| SiMAPKK6-2 | SETIT_002024mg | TATA-box | TATA | 1558 | 1566 | core promoter element |
| SiMAPKK6-2 | SETIT_002024mg | Sp1 | GGGCGG | 1829 | 1841 | light responsive element |
| SiMAPKK6-2 | SETIT_002024mg | GT1-motif | GGTTAAT | 278 | 292 | light responsive element |
| SiMAPKK6-2 | SETIT_002024mg | GT1-motif | GGTTAA | 280 | 292 | light responsive element |
| SiMAPKK6-2 | SETIT_002024mg | Box 4 | ATTAAT | 820 | 832 | light responsive element |
| SiMAPKK6-2 | SETIT_002024mg | Box 4 | ATTAAT | 829 | 841 | light responsive element |
| SiMAPKK6-2 | SETIT_002024mg | Box 4 | ATTAAT | 858 | 870 | light responsive element |
| SiMAPKK6-2 | SETIT_002024mg | ATCT-motif | AATCTAATCC | 597 | 615 | light responsive element |
| SiMAPKK6-2 | SETIT_002024mg | GA-motif | ATAGATAA | 15 | 31 | light responsive element |
| SiMAPKK6-2 | SETIT_002024mg | GATA-motif | GATAGGA | 198 | 212 | light responsive element |
| SiMAPKK6-2 | SETIT_002024mg | GATA-motif | GATAGGG | 1409 | 1423 | light responsive element |
| SiMAPKK6-2 | SETIT_002024mg | GATA-motif | GATAGGA | 1935 | 1949 | light responsive element |
| SiMAPKK6-2 | SETIT_002024mg | TCCC-motif | TCTCCCT | 1412 | 1426 | light responsive element |
| SiMAPKK6-2 | SETIT_002024mg | TCCC-motif | TCTCCCT | 1968 | 1982 | light responsive element |
| SiMAPKK6-2 | SETIT_002024mg | LAMP-element | CTTTATCA | 539 | 555 | light responsive element |
| SiMAPKK6-2 | SETIT_002024mg | TCT-motif | TCTTAC | 412 | 424 | light responsive element |
| SiMAPKK6-2 | SETIT_002024mg | AE-box | AGAAACAA | 231 | 247 | light responsive element |
| SiMAPKK10-1 | SETIT_019527mg | MSA-like | (T/C)C(T/C)AACGG(T/C)(T/C)A | 289.5 | 306.5 | cell cycle control element |
| SiMAPKK10-1 | SETIT_019527mg | ABRE | ACGTG | 883 | 893 | abscisic acid response element |
| SiMAPKK10-1 | SETIT_019527mg | ARE | AAACCA | 1381 | 1393 | anaerobic inducing element |
| SiMAPKK10-1 | SETIT_019527mg | ARE | AAACCA | 1627 | 1639 | anaerobic inducing element |
| SiMAPKK10-1 | SETIT_019527mg | circadian | CAAAGATATC | 915 | 933 | circadian rhythm control element |
| SiMAPKK10-1 | SETIT_019527mg | G-Box | CACGTT | 881 | 893 | light responsive element |
| SiMAPKK10-1 | SETIT_019527mg | O2-site | GATGATGTGG | 437 | 455 | Zein metabolism regulatory elements |
| SiMAPKK10-1 | SETIT_019527mg | CAAT-box | CAAAT | 34 | 44 | common cis-acting element |
| SiMAPKK10-1 | SETIT_019527mg | CAAT-box | CAAAT | 128 | 138 | common cis-acting element |
| SiMAPKK10-1 | SETIT_019527mg | CAAT-box | CAAAT | 240 | 250 | common cis-acting element |
| SiMAPKK10-1 | SETIT_019527mg | CAAT-box | CAAAT | 249 | 259 | common cis-acting element |
| SiMAPKK10-1 | SETIT_019527mg | CAAT-box | CAAAT | 313 | 323 | common cis-acting element |
| SiMAPKK10-1 | SETIT_019527mg | CAAT-box | CCAAT | 345 | 355 | common cis-acting element |
| SiMAPKK10-1 | SETIT_019527mg | CAAT-box | CAAAT | 417 | 427 | common cis-acting element |
| SiMAPKK10-1 | SETIT_019527mg | CAAT-box | CCAAT | 431 | 441 | common cis-acting element |
| SiMAPKK10-1 | SETIT_019527mg | CAAT-box | CCAAT | 681 | 691 | common cis-acting element |
| SiMAPKK10-1 | SETIT_019527mg | CAAT-box | CCAAT | 899 | 909 | common cis-acting element |
| SiMAPKK10-1 | SETIT_019527mg | CAAT-box | CAAAT | 1186 | 1196 | common cis-acting element |
| SiMAPKK10-1 | SETIT_019527mg | CAAT-box | CCAAT | 1912 | 1922 | common cis-acting element |
| SiMAPKK10-1 | SETIT_019527mg | TATA-box | ATATAT | 211 | 223 | core promoter element |
| SiMAPKK10-1 | SETIT_019527mg | TATA-box | TATA | 214 | 222 | core promoter element |
| SiMAPKK10-1 | SETIT_019527mg | TATA-box | ATTATA | 459 | 471 | core promoter element |
| SiMAPKK10-1 | SETIT_019527mg | TATA-box | TATAA | 461 | 471 | core promoter element |
| SiMAPKK10-1 | SETIT_019527mg | TATA-box | TATA | 463 | 471 | core promoter element |
| SiMAPKK10-1 | SETIT_019527mg | TATA-box | TATATA | 1351 | 1363 | core promoter element |
| SiMAPKK10-1 | SETIT_019527mg | TATA-box | ATATAT | 1352 | 1364 | core promoter element |
| SiMAPKK10-1 | SETIT_019527mg | TATA-box | TATA | 1355 | 1363 | core promoter element |
| SiMAPKK10-1 | SETIT_019527mg | TATA-box | TATA | 1371 | 1379 | core promoter element |
| SiMAPKK10-1 | SETIT_019527mg | GT1-motif | GGTTAAT | 337 | 351 | light responsive element |
| SiMAPKK10-1 | SETIT_019527mg | GT1-motif | GGTTAA | 339 | 351 | light responsive element |
| SiMAPKK10-1 | SETIT_019527mg | GT1-motif | GTGTGTGAA | 1947 | 1965 | light responsive element |
| SiMAPKK10-1 | SETIT_019527mg | MBS | CAACTG | 259 | 271 | MYB binding site involved in drought-inducibility |
| SiMAPKK10-1 | SETIT_019527mg | MBS | CAACTG | 686 | 698 | MYB binding site involved in drought-inducibility |
| SiMAPKK10-1 | SETIT_019527mg | TCT-motif | TCTTAC | 808 | 820 | light responsive element |
| SiMAPKK10-1 | SETIT_019527mg | GATA-motif | AAGATAAGATT | 797 | 817 | light responsive element |
| SiMAPKK10-1 | SETIT_019527mg | GTGGC-motif | CATCGTGTGGC | 1408 | 1428 | light responsive element |
| SiMAPKK10-1 | SETIT_019527mg | TCCC-motif | TCTCCCT | 787 | 801 | light responsive element |
| SiMAPKK10-1 | SETIT_019527mg | AE-box | AGAAACAA | 141 | 157 | light responsive element |
| SiMAPKK10-2 | SETIT_036560mg | TC-rich repeats | ATTCTCTAAC | 855 | 873 | defense and stress response elements |
| SiMAPKK10-2 | SETIT_036560mg | LTR | CCGAAA | 1037 | 1049 | low temperature response element |
| SiMAPKK10-2 | SETIT_036560mg | LTR | CCGAAA | 1595 | 1607 | low temperature response element |
| SiMAPKK10-2 | SETIT_036560mg | LTR | CCGAAA | 1802 | 1814 | low temperature response element |
| SiMAPKK10-2 | SETIT_036560mg | ABRE | ACGTG | 42 | 52 | abscisic acid response element |
| SiMAPKK10-2 | SETIT_036560mg | ABRE | ACGTG | 137 | 147 | abscisic acid response element |
| SiMAPKK10-2 | SETIT_036560mg | ABRE | CACGTG | 224 | 236 | abscisic acid response element |
| SiMAPKK10-2 | SETIT_036560mg | ABRE | ACGTG | 226 | 236 | abscisic acid response element |
| SiMAPKK10-2 | SETIT_036560mg | ABRE | ACGTG | 315 | 325 | abscisic acid response element |
| SiMAPKK10-2 | SETIT_036560mg | ABRE | ACGTG | 1423 | 1433 | abscisic acid response element |
| SiMAPKK10-2 | SETIT_036560mg | A-box | CCGTCC | 117 | 129 | cis-acting regulatory element |
| SiMAPKK10-2 | SETIT_036560mg | ARE | AAACCA | 850 | 862 | anaerobic inducing element |
| SiMAPKK10-2 | SETIT_036560mg | ARE | AAACCA | 1989 | 2001 | anaerobic inducing element |
| SiMAPKK10-2 | SETIT_036560mg | AuxRR-core | GGTCCAT | 1207 | 1221 | auxin-responsive element |
| SiMAPKK10-2 | SETIT_036560mg | G-box | CACGTC | 135 | 147 | light responsive element |
| SiMAPKK10-2 | SETIT_036560mg | G-box | CACGTG | 224 | 236 | light responsive element |
| SiMAPKK10-2 | SETIT_036560mg | G-box | CACGTC | 313 | 325 | light responsive element |
| SiMAPKK10-2 | SETIT_036560mg | G-box | TACGTG | 1421 | 1433 | light responsive element |
| SiMAPKK10-2 | SETIT_036560mg | G-Box | CACGTT | 40 | 52 | light responsive element |
| SiMAPKK10-2 | SETIT_036560mg | G-Box | CACGTG | 224 | 236 | light responsive element |
| SiMAPKK10-2 | SETIT_036560mg | TGACG-motif | TGACG | 135 | 145 | MeJA response regulatory element |
| SiMAPKK10-2 | SETIT_036560mg | CGTCA-motif | CGTCA | 135 | 145 | MeJA response regulatory element |
| SiMAPKK10-2 | SETIT_036560mg | O2-site | GATGA(C/T)(A/G)TG(A/G) | 130 | 146 | Zein metabolism regulatory elements |
| SiMAPKK10-2 | SETIT_036560mg | O2-site | GATGATGTGG | 645 | 663 | Zein metabolism regulatory elements |
| SiMAPKK10-2 | SETIT_036560mg | CAT-box | GCCACT | 24 | 36 | meristem expression regulatory element |
| SiMAPKK10-2 | SETIT_036560mg | CAT-box | GCCACT | 529 | 541 | meristem expression regulatory element |
| SiMAPKK10-2 | SETIT_036560mg | GCN4_motif | TGAGTCA | 1807 | 1821 | endosperm expression regulatory element |
| SiMAPKK10-2 | SETIT_036560mg | CAAT-box | CCAAT | 231 | 241 | common cis-acting element |
| SiMAPKK10-2 | SETIT_036560mg | CAAT-box | CCAAT | 667 | 677 | common cis-acting element |
| SiMAPKK10-2 | SETIT_036560mg | CAAT-box | CAAAT | 692 | 702 | common cis-acting element |
| SiMAPKK10-2 | SETIT_036560mg | CAAT-box | CCAAT | 765 | 775 | common cis-acting element |
| SiMAPKK10-2 | SETIT_036560mg | CAAT-box | CAAAT | 781 | 791 | common cis-acting element |
| SiMAPKK10-2 | SETIT_036560mg | CAAT-box | CAAAT | 839 | 849 | common cis-acting element |
| SiMAPKK10-2 | SETIT_036560mg | CAAT-box | CAAAT | 987 | 997 | common cis-acting element |
| SiMAPKK10-2 | SETIT_036560mg | CAAT-box | CCAAT | 1143 | 1153 | common cis-acting element |
| SiMAPKK10-2 | SETIT_036560mg | CAAT-box | CCAAT | 1340 | 1350 | common cis-acting element |
| SiMAPKK10-2 | SETIT_036560mg | TATA-box | TATA | 335 | 343 | core promoter element |
| SiMAPKK10-2 | SETIT_036560mg | TATA-box | TATACA | 1128 | 1140 | core promoter element |
| SiMAPKK10-2 | SETIT_036560mg | TATA-box | TATATA | 1130 | 1142 | core promoter element |
| SiMAPKK10-2 | SETIT_036560mg | TATA-box | TATA | 1134 | 1142 | core promoter element |
| SiMAPKK10-2 | SETIT_036560mg | TATA-box | ATTATA | 1622 | 1634 | core promoter element |
| SiMAPKK10-2 | SETIT_036560mg | TATA-box | TATAA | 1624 | 1634 | core promoter element |
| SiMAPKK10-2 | SETIT_036560mg | TATA-box | TATA | 1626 | 1634 | core promoter element |
| SiMAPKK10-2 | SETIT_036560mg | TATA-box | TATA | 1655 | 1663 | core promoter element |
| SiMAPKK10-2 | SETIT_036560mg | TATA-box | ATATAT | 1872 | 1884 | core promoter element |
| SiMAPKK10-2 | SETIT_036560mg | TATA-box | TATATA | 1873 | 1885 | core promoter element |
| SiMAPKK10-2 | SETIT_036560mg | TATA-box | TATA | 1877 | 1885 | core promoter element |
| SiMAPKK10-2 | SETIT_036560mg | GARE-motif | TCTGTTG | 58 | 72 | gibberellin-responsive element |
| SiMAPKK10-2 | SETIT_036560mg | GT1-motif | GGTTAA | 1511 | 1523 | light responsive element |
| SiMAPKK10-2 | SETIT_036560mg | TCT-motif | TCTTAC | 806 | 818 | light responsive element |
| SiMAPKK10-2 | SETIT_036560mg | GATA-motif | GATAGGA | 428 | 442 | light responsive element |
| SiMAPKK10-2 | SETIT_036560mg | GATA-motif | AAGATAAGATT | 1470 | 1490 | light responsive element |
| SiMAPKK10-3 | SETIT_004678mg | AT-rich element | ATAGAAATCAA | 1450 | 1470 | ATBP-1 binding site |
| SiMAPKK10-3 | SETIT_004678mg | LTR | CCGAAA | 416 | 428 | low temperature response element |
| SiMAPKK10-3 | SETIT_004678mg | LTR | CCGAAA | 1057 | 1069 | low temperature response element |
| SiMAPKK10-3 | SETIT_004678mg | ABRE | ACGTG | 490 | 500 | abscisic acid response element |
| SiMAPKK10-3 | SETIT_004678mg | ABRE | ACGTG | 1200 | 1210 | abscisic acid response element |
| SiMAPKK10-3 | SETIT_004678mg | ABRE | CACGTG | 1327 | 1339 | abscisic acid response element |
| SiMAPKK10-3 | SETIT_004678mg | ABRE | ACGTG | 1329 | 1339 | abscisic acid response element |
| SiMAPKK10-3 | SETIT_004678mg | G-box | CACGTC | 488 | 500 | light responsive element |
| SiMAPKK10-3 | SETIT_004678mg | G-box | TACGTG | 1199 | 1211 | light responsive element |
| SiMAPKK10-3 | SETIT_004678mg | G-box | CACGAC | 1254 | 1266 | light responsive element |
| SiMAPKK10-3 | SETIT_004678mg | G-box | TAACACGTAG | 1323 | 1341 | light responsive element |
| SiMAPKK10-3 | SETIT_004678mg | G-box | CACGTG | 1327 | 1339 | light responsive element |
| SiMAPKK10-3 | SETIT_004678mg | G-Box | CACGTG | 1327 | 1339 | light responsive element |
| SiMAPKK10-3 | SETIT_004678mg | TGACG-motif | TGACG | 256 | 266 | MeJA response regulatory element |
| SiMAPKK10-3 | SETIT_004678mg | TGACG-motif | TGACG | 345 | 355 | MeJA response regulatory element |
| SiMAPKK10-3 | SETIT_004678mg | TGACG-motif | TGACG | 404 | 414 | MeJA response regulatory element |
| SiMAPKK10-3 | SETIT_004678mg | CGTCA-motif | CGTCA | 256 | 266 | MeJA response regulatory element |
| SiMAPKK10-3 | SETIT_004678mg | CGTCA-motif | CGTCA | 345 | 355 | MeJA response regulatory element |
| SiMAPKK10-3 | SETIT_004678mg | CGTCA-motif | CGTCA | 404 | 414 | MeJA response regulatory element |
| SiMAPKK10-3 | SETIT_004678mg | CAT-box | GCCACT | 834 | 846 | meristem expression regulatory element |
| SiMAPKK10-3 | SETIT_004678mg | CAT-box | GCCACT | 1414 | 1426 | meristem expression regulatory element |
| SiMAPKK10-3 | SETIT_004678mg | CAT-box | GCCACT | 1752 | 1764 | meristem expression regulatory element |
| SiMAPKK10-3 | SETIT_004678mg | CAAT-box | CCAAT | 226 | 236 | common cis-acting element |
| SiMAPKK10-3 | SETIT_004678mg | CAAT-box | CCAAT | 325 | 335 | common cis-acting element |
| SiMAPKK10-3 | SETIT_004678mg | CAAT-box | CCAAT | 379 | 389 | common cis-acting element |
| SiMAPKK10-3 | SETIT_004678mg | CAAT-box | CAACCAACTCC | 455 | 475 | common cis-acting element |
| SiMAPKK10-3 | SETIT_004678mg | CAAT-box | CCAAT | 960 | 970 | common cis-acting element |
| SiMAPKK10-3 | SETIT_004678mg | CAAT-box | CAAAT | 1303 | 1313 | common cis-acting element |
| SiMAPKK10-3 | SETIT_004678mg | CAAT-box | CCAAT | 1363 | 1373 | common cis-acting element |
| SiMAPKK10-3 | SETIT_004678mg | CAAT-box | CCAAT | 1571 | 1581 | common cis-acting element |
| SiMAPKK10-3 | SETIT_004678mg | CAAT-box | CAAAT | 1863 | 1873 | common cis-acting element |
| SiMAPKK10-3 | SETIT_004678mg | CAAT-box | CCAAT | 1967 | 1977 | common cis-acting element |
| SiMAPKK10-3 | SETIT_004678mg | TATA-box | TATA | 1348 | 1356 | core promoter element |
| SiMAPKK10-3 | SETIT_004678mg | GC-motif | CCCCCG | 193 | 205 | hypoxia-specifically induced elements |
| SiMAPKK10-3 | SETIT_004678mg | GC-motif | CCCCCG | 687 | 699 | hypoxia-specifically induced elements |
| SiMAPKK10-3 | SETIT_004678mg | GT1-motif | GGTTAA | 986 | 998 | light responsive element |
| SiMAPKK10-3 | SETIT_004678mg | GT1-motif | GGTTAA | 988 | 1000 | light responsive element |
| SiMAPKK10-3 | SETIT_004678mg | GT1-motif | GGTTAA | 1010 | 1022 | light responsive element |
| SiMAPKK10-3 | SETIT_004678mg | Sp1 | GGGCGG | -3 | 9 | light responsive element |
| SiMAPKK10-3 | SETIT_004678mg | Sp1 | GGGCGG | 208 | 220 | light responsive element |
| SiMAPKK10-3 | SETIT_004678mg | Sp1 | GGGCGG | 633 | 645 | light responsive element |
| SiMAPKK10-3 | SETIT_004678mg | Sp1 | GGGCGG | 734 | 746 | light responsive element |
| SiMAPKK10-3 | SETIT_004678mg | Sp1 | GGGCGG | 1294 | 1306 | light responsive element |
| SiMAPKK10-3 | SETIT_004678mg | Sp1 | GGGCGG | 1878 | 1890 | light responsive element |
| SiMAPKK10-3 | SETIT_004678mg | TCCC-motif | TCTCCCT | 1425 | 1439 | light responsive element |
| SiMAPKK10-4 | SETIT_031963mg | LTR | CCGAAA | 1488 | 1500 | low temperature response element |
| SiMAPKK10-4 | SETIT_031963mg | ABRE | ACGTG | 137 | 147 | abscisic acid response element |
| SiMAPKK10-4 | SETIT_031963mg | ABRE | ACGTG | 451 | 461 | abscisic acid response element |
| SiMAPKK10-4 | SETIT_031963mg | A-box | CCGTCC | 66 | 78 | cis-acting regulatory element |
| SiMAPKK10-4 | SETIT_031963mg | A-box | CCGTCC | 1953 | 1965 | cis-acting regulatory element |
| SiMAPKK10-4 | SETIT_031963mg | G-box | CACGTC | 135 | 147 | light responsive element |
| SiMAPKK10-4 | SETIT_031963mg | G-box | GCCACGTGGA | 911 | 929 | light responsive element |
| SiMAPKK10-4 | SETIT_031963mg | G-Box | CACGTT | 450 | 462 | light responsive element |
| SiMAPKK10-4 | SETIT_031963mg | TGACG-motif | TGACG | 135 | 145 | MeJA response regulatory element |
| SiMAPKK10-4 | SETIT_031963mg | TGACG-motif | TGACG | 188 | 198 | MeJA response regulatory element |
| SiMAPKK10-4 | SETIT_031963mg | TGACG-motif | TGACG | 676 | 686 | MeJA response regulatory element |
| SiMAPKK10-4 | SETIT_031963mg | TGACG-motif | TGACG | 1242 | 1252 | MeJA response regulatory element |
| SiMAPKK10-4 | SETIT_031963mg | TGACG-motif | TGACG | 1275 | 1285 | MeJA response regulatory element |
| SiMAPKK10-4 | SETIT_031963mg | TGACG-motif | TGACG | 1508 | 1518 | MeJA response regulatory element |
| SiMAPKK10-4 | SETIT_031963mg | TGACG-motif | TGACG | 1769 | 1779 | MeJA response regulatory element |
| SiMAPKK10-4 | SETIT_031963mg | TGACG-motif | TGACG | 1814 | 1824 | MeJA response regulatory element |
| SiMAPKK10-4 | SETIT_031963mg | CGTCA-motif | CGTCA | 135 | 145 | MeJA response regulatory element |
| SiMAPKK10-4 | SETIT_031963mg | CGTCA-motif | CGTCA | 188 | 198 | MeJA response regulatory element |
| SiMAPKK10-4 | SETIT_031963mg | CGTCA-motif | CGTCA | 676 | 686 | MeJA response regulatory element |
| SiMAPKK10-4 | SETIT_031963mg | CGTCA-motif | CGTCA | 1242 | 1252 | MeJA response regulatory element |
| SiMAPKK10-4 | SETIT_031963mg | CGTCA-motif | CGTCA | 1275 | 1285 | MeJA response regulatory element |
| SiMAPKK10-4 | SETIT_031963mg | CGTCA-motif | CGTCA | 1508 | 1518 | MeJA response regulatory element |
| SiMAPKK10-4 | SETIT_031963mg | CGTCA-motif | CGTCA | 1769 | 1779 | MeJA response regulatory element |
| SiMAPKK10-4 | SETIT_031963mg | CGTCA-motif | CGTCA | 1814 | 1824 | MeJA response regulatory element |
| SiMAPKK10-4 | SETIT_031963mg | CAAT-box | CAAAT | 262 | 272 | common cis-acting element |
| SiMAPKK10-4 | SETIT_031963mg | CAAT-box | CAAAT | 276 | 286 | common cis-acting element |
| SiMAPKK10-4 | SETIT_031963mg | CAAT-box | CAAAT | 339 | 349 | common cis-acting element |
| SiMAPKK10-4 | SETIT_031963mg | CAAT-box | CAAAT | 482 | 492 | common cis-acting element |
| SiMAPKK10-4 | SETIT_031963mg | CAAT-box | CAAAT | 742 | 752 | common cis-acting element |
| SiMAPKK10-4 | SETIT_031963mg | CAAT-box | CAAAT | 808 | 818 | common cis-acting element |
| SiMAPKK10-4 | SETIT_031963mg | CAAT-box | CAAAT | 911 | 921 | common cis-acting element |
| SiMAPKK10-4 | SETIT_031963mg | CAAT-box | CAAAT | 1003 | 1013 | common cis-acting element |
| SiMAPKK10-4 | SETIT_031963mg | CAAT-box | CAAAT | 1087 | 1097 | common cis-acting element |
| SiMAPKK10-4 | SETIT_031963mg | CAAT-box | CCAAT | 1120 | 1130 | common cis-acting element |
| SiMAPKK10-4 | SETIT_031963mg | CAAT-box | CAAAT | 1293 | 1303 | common cis-acting element |
| SiMAPKK10-4 | SETIT_031963mg | CAAT-box | CAAAT | 1313 | 1323 | common cis-acting element |
| SiMAPKK10-4 | SETIT_031963mg | CAAT-box | CAAAT | 1316 | 1326 | common cis-acting element |
| SiMAPKK10-4 | SETIT_031963mg | CAAT-box | CAAAT | 1322 | 1332 | common cis-acting element |
| SiMAPKK10-4 | SETIT_031963mg | TATA-box | TATATA | 125 | 137 | core promoter element |
| SiMAPKK10-4 | SETIT_031963mg | TATA-box | ATATAT | 126 | 138 | core promoter element |
| SiMAPKK10-4 | SETIT_031963mg | TATA-box | TATA | 129 | 137 | core promoter element |
| SiMAPKK10-4 | SETIT_031963mg | TATA-box | TATACA | 426 | 438 | core promoter element |
| SiMAPKK10-4 | SETIT_031963mg | TATA-box | TATA | 430 | 438 | core promoter element |
| SiMAPKK10-4 | SETIT_031963mg | TATA-box | TATA | 462 | 470 | core promoter element |
| SiMAPKK10-4 | SETIT_031963mg | TATA-box | TATA | 949 | 957 | core promoter element |
| SiMAPKK10-4 | SETIT_031963mg | TATA-box | TATAA | 1179 | 1189 | core promoter element |
| SiMAPKK10-4 | SETIT_031963mg | TATA-box | TATA | 1181 | 1189 | core promoter element |
| SiMAPKK10-4 | SETIT_031963mg | TATA-box | TACAAAA | 1233 | 1247 | core promoter element |
| SiMAPKK10-4 | SETIT_031963mg | TATA-box | TATA | 1594 | 1602 | core promoter element |
| SiMAPKK10-4 | SETIT_031963mg | TATA-box | ATATAT | 1664 | 1676 | core promoter element |
| SiMAPKK10-4 | SETIT_031963mg | TATA-box | TATA | 1667 | 1675 | core promoter element |
| SiMAPKK10-4 | SETIT_031963mg | TATA-box | TATAA | 1872 | 1882 | core promoter element |
| SiMAPKK10-4 | SETIT_031963mg | TATA-box | TATA | 1874 | 1882 | core promoter element |
| SiMAPKK10-4 | SETIT_031963mg | P-box | CCTTTTG | 1595 | 1609 | gibberellin-responsive element |
| SiMAPKK10-4 | SETIT_031963mg | GT1-motif | GGTTAA | 164 | 176 | light responsive element |
| SiMAPKK10-4 | SETIT_031963mg | GT1-motif | GGTTAAT | 382 | 396 | light responsive element |
| SiMAPKK10-4 | SETIT_031963mg | GT1-motif | GGTTAA | 1263 | 1275 | light responsive element |
| SiMAPKK10-4 | SETIT_031963mg | Sp1 | GGGCGG | 1927 | 1939 | light responsive element |
| SiMAPKK10-4 | SETIT_031963mg | MBS | CAACTG | 147 | 159 | MYB binding site involved in drought-inducibility |
| SiMAPKK10-4 | SETIT_031963mg | Box 4 | ATTAAT | 492 | 504 | light responsive element |
| SiMAPKK10-4 | SETIT_031963mg | I-box | cCATATCCAAT | 529 | 549 | light responsive element |
| SiRAF1 | SETIT_016154mg | ABRE | ACGTG | 551 | 561 | abscisic acid response element |
| SiRAF1 | SETIT_016154mg | ABRE | GCCGCGTGGC | 701 | 719 | abscisic acid response element |
| SiRAF1 | SETIT_016154mg | ARE | AAACCA | 216 | 228 | anaerobic inducing element |
| SiRAF1 | SETIT_016154mg | ARE | AAACCA | 348 | 360 | anaerobic inducing element |
| SiRAF1 | SETIT_016154mg | ARE | AAACCA | 627 | 639 | anaerobic inducing element |
| SiRAF1 | SETIT_016154mg | ARE | AAACCA | 1176 | 1188 | anaerobic inducing element |
| SiRAF1 | SETIT_016154mg | ARE | AAACCA | 1308 | 1320 | anaerobic inducing element |
| SiRAF1 | SETIT_016154mg | TGA-element | AACGAC | 249 | 261 | auxin-responsive element |
| SiRAF1 | SETIT_016154mg | TGA-element | AACGAC | 381 | 393 | auxin-responsive element |
| SiRAF1 | SETIT_016154mg | TGA-element | AACGAC | 489 | 501 | auxin-responsive element |
| SiRAF1 | SETIT_016154mg | TGA-element | AACGAC | 588 | 600 | auxin-responsive element |
| SiRAF1 | SETIT_016154mg | CAAT-box | CAAAT | 99 | 109 | common cis-acting element |
| SiRAF1 | SETIT_016154mg | CAAT-box | CCAAT | 907 | 917 | common cis-acting element |
| SiRAF1 | SETIT_016154mg | CAAT-box | CCAAT | 1023 | 1033 | common cis-acting element |
| SiRAF1 | SETIT_016154mg | CAAT-box | CAAAT | 1044 | 1054 | common cis-acting element |
| SiRAF1 | SETIT_016154mg | CAAT-box | CAACCAACTCC | 1096 | 1116 | common cis-acting element |
| SiRAF1 | SETIT_016154mg | CAAT-box | CAAAT | 1717 | 1727 | common cis-acting element |
| SiRAF1 | SETIT_016154mg | CAAT-box | CAAAT | 1733 | 1743 | common cis-acting element |
| SiRAF1 | SETIT_016154mg | CAAT-box | CAAAT | 1752 | 1762 | common cis-acting element |
| SiRAF1 | SETIT_016154mg | CAAT-box | CCAAT | 1886 | 1896 | common cis-acting element |
| SiRAF1 | SETIT_016154mg | CAAT-box | CAAAT | 1913 | 1923 | common cis-acting element |
| SiRAF1 | SETIT_016154mg | TATA-box | ATTATA | 60 | 72 | core promoter element |
| SiRAF1 | SETIT_016154mg | TATA-box | TATAA | 62 | 72 | core promoter element |
| SiRAF1 | SETIT_016154mg | TATA-box | TATA | 64 | 72 | core promoter element |
| SiRAF1 | SETIT_016154mg | TATA-box | TATA | 204 | 212 | core promoter element |
| SiRAF1 | SETIT_016154mg | TATA-box | ATATAT | 333 | 345 | core promoter element |
| SiRAF1 | SETIT_016154mg | TATA-box | TATA | 336 | 344 | core promoter element |
| SiRAF1 | SETIT_016154mg | TATA-box | TACAAAA | 1520 | 1534 | core promoter element |
| SiRAF1 | SETIT_016154mg | TATA-box | TATA | 1929 | 1937 | core promoter element |
| SiRAF1 | SETIT_016154mg | GCN4_motif | TGAGTCA | 234 | 248 | endosperm expression regulatory element |
| SiRAF1 | SETIT_016154mg | GCN4_motif | TGAGTCA | 366 | 380 | endosperm expression regulatory element |
| SiRAF1 | SETIT_016154mg | GCN4_motif | TGAGTCA | 475 | 489 | endosperm expression regulatory element |
| SiRAF1 | SETIT_016154mg | TATC-box | TATCCCA | 1797 | 1811 | gibberellin response element |
| SiRAF1 | SETIT_016154mg | GC-motif | CCCCCG | 683 | 695 | hypoxia-specifically induced elements |
| SiRAF1 | SETIT_016154mg | GC-motif | CCCCCG | 807 | 819 | hypoxia-specifically induced elements |
| SiRAF1 | SETIT_016154mg | GC-motif | CCCCCG | 957 | 969 | hypoxia-specifically induced elements |
| SiRAF1 | SETIT_016154mg | GC-motif | CCCCCG | 1152 | 1164 | hypoxia-specifically induced elements |
| SiRAF1 | SETIT_016154mg | ACE | GACACGTATG | 544 | 562 | light responsive element |
| SiRAF1 | SETIT_016154mg | ACE | CTAACGTATT | 1706 | 1724 | light responsive element |
| SiRAF1 | SETIT_016154mg | G-box | TACGTG | 549 | 561 | light responsive element |
| SiRAF1 | SETIT_016154mg | G-box | CACGAC | 1546 | 1558 | light responsive element |
| SiRAF1 | SETIT_016154mg | G-box | CACGAC | 1918 | 1930 | light responsive element |
| SiRAF1 | SETIT_016154mg | Sp1 | GGGCGG | 578 | 590 | light responsive element |
| SiRAF1 | SETIT_016154mg | Sp1 | GGGCGG | 680 | 692 | light responsive element |
| SiRAF1 | SETIT_016154mg | Sp1 | GGGCGG | 695 | 707 | light responsive element |
| SiRAF1 | SETIT_016154mg | Sp1 | GGGCGG | 810 | 822 | light responsive element |
| SiRAF1 | SETIT_016154mg | Sp1 | GGGCGG | 814 | 826 | light responsive element |
| SiRAF1 | SETIT_016154mg | Sp1 | GGGCGG | 1387 | 1399 | light responsive element |
| SiRAF1 | SETIT_016154mg | ATCT-motif | AATCTAATCC | 219 | 237 | light responsive element |
| SiRAF1 | SETIT_016154mg | TCT-motif | TCTTAC | 1240 | 1252 | light responsive element |
| SiRAF1 | SETIT_016154mg | I-box | cGATAAGGCG | 1354 | 1372 | light responsive element |
| SiRAF1 | SETIT_016154mg | Gap-box | CAAATGAA(A/G)A | 94.5 | 113.5 | light responsive element |
| SiRAF1 | SETIT_016154mg | AE-box | AGAAACAA | 1512 | 1528 | light responsive element |
| SiRAF1 | SETIT_016154mg | TGACG-motif | TGACG | 1355 | 1365 | MeJA response regulatory element |
| SiRAF1 | SETIT_016154mg | TGACG-motif | TGACG | 1470 | 1480 | MeJA response regulatory element |
| SiRAF1 | SETIT_016154mg | CGTCA-motif | CGTCA | 1355 | 1365 | MeJA response regulatory element |
| SiRAF1 | SETIT_016154mg | CGTCA-motif | CGTCA | 1470 | 1480 | MeJA response regulatory element |
| SiRAF1 | SETIT_016154mg | CAT-box | GCCACT | 721 | 733 | meristem expression regulatory element |
| SiRAF1 | SETIT_016154mg | MBS | CAACTG | 1101 | 1113 | MYB binding site involved in drought-inducibility |
| SiRAF1 | SETIT_016154mg | MRE | AACCTAA | 259 | 273 | MYB binding site involved in light responsiveness |
| SiRAF1 | SETIT_016154mg | MRE | AACCTAA | 1436 | 1450 | MYB binding site involved in light responsiveness |
| SiRAF1 | SETIT_016154mg | CCAAT-box | CAACGG | 138 | 150 | MYBHv1 binding site |
| SiRAF1 | SETIT_016154mg | A-box | CCGTCC | 1246 | 1258 | promoter and enhancer cis-acting regulatory elements |
| SiRAF2 | SETIT_005722mg | ABRE | ACGTG | 14 | 24 | abscisic acid response element |
| SiRAF2 | SETIT_005722mg | ABRE | ACGTG | 687 | 697 | abscisic acid response element |
| SiRAF2 | SETIT_005722mg | ABRE | CACGTG | 1190 | 1202 | abscisic acid response element |
| SiRAF2 | SETIT_005722mg | ABRE | ACGTG | 1192 | 1202 | abscisic acid response element |
| SiRAF2 | SETIT_005722mg | ABRE | CGCACGTGTC | 1552 | 1570 | abscisic acid response element |
| SiRAF2 | SETIT_005722mg | ABRE | ACGTG | 1714 | 1724 | abscisic acid response element |
| SiRAF2 | SETIT_005722mg | ARE | AAACCA | 1025 | 1037 | anaerobic inducing element |
| SiRAF2 | SETIT_005722mg | CAAT-box | CAAAT | 170 | 180 | common cis-acting element |
| SiRAF2 | SETIT_005722mg | CAAT-box | CAAAT | 350 | 360 | common cis-acting element |
| SiRAF2 | SETIT_005722mg | CAAT-box | CAAAT | 367 | 377 | common cis-acting element |
| SiRAF2 | SETIT_005722mg | CAAT-box | CAAAT | 579 | 589 | common cis-acting element |
| SiRAF2 | SETIT_005722mg | CAAT-box | CCAAT | 1116 | 1126 | common cis-acting element |
| SiRAF2 | SETIT_005722mg | CAAT-box | CAAAT | 1432 | 1442 | common cis-acting element |
| SiRAF2 | SETIT_005722mg | TATA-box | TATAAA | 1241 | 1253 | core promoter element |
| SiRAF2 | SETIT_005722mg | TATA-box | TATAA | 1243 | 1253 | core promoter element |
| SiRAF2 | SETIT_005722mg | TATA-box | TATA | 1245 | 1253 | core promoter element |
| SiRAF2 | SETIT_005722mg | TATA-box | TATAAA | 1327 | 1339 | core promoter element |
| SiRAF2 | SETIT_005722mg | TATA-box | TATAA | 1329 | 1339 | core promoter element |
| SiRAF2 | SETIT_005722mg | TATA-box | TATA | 1331 | 1339 | core promoter element |
| SiRAF2 | SETIT_005722mg | TATC-box | TATCCCA | 976 | 990 | gibberellin response element |
| SiRAF2 | SETIT_005722mg | TATC-box | TATCCCA | 1087 | 1101 | gibberellin response element |
| SiRAF2 | SETIT_005722mg | GC-motif | CCCCCG | 1530 | 1542 | hypoxia-specifically induced elements |
| SiRAF2 | SETIT_005722mg | GC-motif | CCCCCG | 1665 | 1677 | hypoxia-specifically induced elements |
| SiRAF2 | SETIT_005722mg | GC-motif | CCCCCG | 1845 | 1857 | hypoxia-specifically induced elements |
| SiRAF2 | SETIT_005722mg | GC-motif | CCCCCG | 1941 | 1953 | hypoxia-specifically induced elements |
| SiRAF2 | SETIT_005722mg | G-box | TACGTG | 685 | 697 | light responsive element |
| SiRAF2 | SETIT_005722mg | G-box | CACGTG | 1190 | 1202 | light responsive element |
| SiRAF2 | SETIT_005722mg | G-box | CACGAC | 1580 | 1592 | light responsive element |
| SiRAF2 | SETIT_005722mg | G-box | CACGTC | 1713 | 1725 | light responsive element |
| SiRAF2 | SETIT_005722mg | G-Box | CACGTT | 13 | 25 | light responsive element |
| SiRAF2 | SETIT_005722mg | G-Box | CACGTGAAA | 1184 | 1202 | light responsive element |
| SiRAF2 | SETIT_005722mg | G-Box | CACGTG | 1190 | 1202 | light responsive element |
| SiRAF2 | SETIT_005722mg | Sp1 | GGGCGG | 1718 | 1730 | light responsive element |
| SiRAF2 | SETIT_005722mg | TCCC-motif | TCTCCCT | 1798 | 1812 | light responsive element |
| SiRAF2 | SETIT_005722mg | TCCC-motif | TCTCCCT | 1830 | 1844 | light responsive element |
| SiRAF2 | SETIT_005722mg | TCCC-motif | TCTCCCT | 1949 | 1963 | light responsive element |
| SiRAF2 | SETIT_005722mg | I-box | GTATAAGGCC | 1536 | 1554 | light responsive element |
| SiRAF2 | SETIT_005722mg | GATA-motif | GATAGGG | 1795 | 1809 | light responsive element |
| SiRAF2 | SETIT_005722mg | TGACG-motif | TGACG | 58 | 68 | MeJA response regulatory element |
| SiRAF2 | SETIT_005722mg | CGTCA-motif | CGTCA | 58 | 68 | MeJA response regulatory element |
| SiRAF2 | SETIT_005722mg | CAT-box | GCCACT | 1805 | 1817 | meristem expression regulatory element |
| SiRAF2 | SETIT_005722mg | MRE | AACCTAA | 694 | 708 | MYB binding site involved in light responsiveness |
| SiRAF2 | SETIT_005722mg | CCAAT-box | CAACGG | 1722 | 1734 | MYBHv1 binding site |
| SiRAF2 | SETIT_005722mg | A-box | CCGTCC | 1054 | 1066 | promoter and enhancer cis-acting regulatory elements |
| SiRAF2 | SETIT_005722mg | A-box | CCGTCC | 1205 | 1217 | promoter and enhancer cis-acting regulatory elements |
| SiRAF2 | SETIT_005722mg | O2-site | GATGACATGG | 1339 | 1357 | Zein metabolism regulatory elements |
| SiRAF3 | SETIT_005743mg | ABRE | CGCACGTGTC | 101 | 119 | abscisic acid response element |
| SiRAF3 | SETIT_005743mg | ABRE | GCCGCGTGGC | 698 | 716 | abscisic acid response element |
| SiRAF3 | SETIT_005743mg | ABRE | CACGTG | 1002 | 1014 | abscisic acid response element |
| SiRAF3 | SETIT_005743mg | ABRE | ACGTG | 1004 | 1014 | abscisic acid response element |
| SiRAF3 | SETIT_005743mg | ABRE | ACGTG | 1208 | 1218 | abscisic acid response element |
| SiRAF3 | SETIT_005743mg | ABRE | CGCACGTGTC | 1383 | 1401 | abscisic acid response element |
| SiRAF3 | SETIT_005743mg | ABRE | CACGTG | 1388 | 1400 | abscisic acid response element |
| SiRAF3 | SETIT_005743mg | ABRE | ACGTG | 1390 | 1400 | abscisic acid response element |
| SiRAF3 | SETIT_005743mg | ARE | AAACCA | 1447 | 1459 | anaerobic inducing element |
| SiRAF3 | SETIT_005743mg | ARE | AAACCA | 1713 | 1725 | anaerobic inducing element |
| SiRAF3 | SETIT_005743mg | MSA-like | (T/C)C(T/C)AACGG(T/C)(T/C)A | 1107 | 1125 | cell cycle control element |
| SiRAF3 | SETIT_005743mg | CAAT-box | CCAAT | 279 | 289 | common cis-acting element |
| SiRAF3 | SETIT_005743mg | CAAT-box | CAAAT | 325 | 335 | common cis-acting element |
| SiRAF3 | SETIT_005743mg | CAAT-box | CAAAT | 338 | 348 | common cis-acting element |
| SiRAF3 | SETIT_005743mg | CAAT-box | CAAAT | 384 | 394 | common cis-acting element |
| SiRAF3 | SETIT_005743mg | CAAT-box | CAAAT | 401 | 411 | common cis-acting element |
| SiRAF3 | SETIT_005743mg | CAAT-box | CAAAT | 1122 | 1132 | common cis-acting element |
| SiRAF3 | SETIT_005743mg | CAAT-box | CCAAT | 1149 | 1159 | common cis-acting element |
| SiRAF3 | SETIT_005743mg | CAAT-box | CCAAT | 1328 | 1338 | common cis-acting element |
| SiRAF3 | SETIT_005743mg | CAAT-box | CCAAT | 1451 | 1461 | common cis-acting element |
| SiRAF3 | SETIT_005743mg | CAAT-box | CAAAT | 1531 | 1541 | common cis-acting element |
| SiRAF3 | SETIT_005743mg | CAAT-box | CAAAT | 1731 | 1741 | common cis-acting element |
| SiRAF3 | SETIT_005743mg | CAAT-box | CAAAT | 1839 | 1849 | common cis-acting element |
| SiRAF3 | SETIT_005743mg | TATA-box | TATA | 873 | 881 | core promoter element |
| SiRAF3 | SETIT_005743mg | TATA-box | TACAAAA | 1029 | 1043 | core promoter element |
| SiRAF3 | SETIT_005743mg | TATA-box | TATACA | 1089 | 1101 | core promoter element |
| SiRAF3 | SETIT_005743mg | TATA-box | TATA | 1093 | 1101 | core promoter element |
| SiRAF3 | SETIT_005743mg | TATA-box | TACAAAA | 1442 | 1456 | core promoter element |
| SiRAF3 | SETIT_005743mg | TATA-box | TACAAAA | 1617 | 1631 | core promoter element |
| SiRAF3 | SETIT_005743mg | TC-rich repeats | GTTTTCTTAC | 1359 | 1377 | defense and stress response elements |
| SiRAF3 | SETIT_005743mg | TC-rich repeats | GTTTTCTTAC | 1693 | 1711 | defense and stress response elements |
| SiRAF3 | SETIT_005743mg | RY-element | CATGCATG | 490 | 506 | elements involved in seed-specific regulation |
| SiRAF3 | SETIT_005743mg | GARE-motif | TCTGTTG | 1178 | 1192 | gibberellin-responsive element |
| SiRAF3 | SETIT_005743mg | GC-motif | CCCCCG | 43 | 55 | hypoxia-specifically induced elements |
| SiRAF3 | SETIT_005743mg | GC-motif | CCCCCG | 85 | 97 | hypoxia-specifically induced elements |
| SiRAF3 | SETIT_005743mg | GC-motif | CCCCCG | 1914 | 1926 | hypoxia-specifically induced elements |
| SiRAF3 | SETIT_005743mg | G-Box | CACGTG | 1002 | 1014 | light responsive element |
| SiRAF3 | SETIT_005743mg | G-Box | CACGTG | 1388 | 1400 | light responsive element |
| SiRAF3 | SETIT_005743mg | G-box | CACGTG | 1002 | 1014 | light responsive element |
| SiRAF3 | SETIT_005743mg | G-box | TACGTG | 1207 | 1219 | light responsive element |
| SiRAF3 | SETIT_005743mg | G-box | CACGTG | 1388 | 1400 | light responsive element |
| SiRAF3 | SETIT_005743mg | Sp1 | GGGCGG | 46 | 58 | light responsive element |
| SiRAF3 | SETIT_005743mg | Sp1 | GGGCGG | 88 | 100 | light responsive element |
| SiRAF3 | SETIT_005743mg | Sp1 | GGGCGG | 1911 | 1923 | light responsive element |
| SiRAF3 | SETIT_005743mg | Sp1 | GGGCGG | 1943 | 1955 | light responsive element |
| SiRAF3 | SETIT_005743mg | ATC-motif | AGTAATCT | 1101 | 1117 | light responsive element |
| SiRAF3 | SETIT_005743mg | Box 4 | ATTAAT | 1509 | 1521 | light responsive element |
| SiRAF3 | SETIT_005743mg | GTGGC-motif | CAGCGTGTGGC | 25 | 45 | light responsive element |
| SiRAF3 | SETIT_005743mg | TCT-motif | TCTTAC | 1769 | 1781 | light responsive element |
| SiRAF3 | SETIT_005743mg | GATA-motif | GATAGGA | 908 | 922 | light responsive element |
| SiRAF3 | SETIT_005743mg | Pc-CMA2c | GCCCACGCA | 96 | 114 | light responsive element |
| SiRAF3 | SETIT_005743mg | CGTCA-motif | CGTCA | 1194 | 1204 | MeJA response regulatory element |
| SiRAF3 | SETIT_005743mg | CGTCA-motif | CGTCA | 1217 | 1227 | MeJA response regulatory element |
| SiRAF3 | SETIT_005743mg | CGTCA-motif | CGTCA | 1672 | 1682 | MeJA response regulatory element |
| SiRAF3 | SETIT_005743mg | CGTCA-motif | CGTCA | 1749 | 1759 | MeJA response regulatory element |
| SiRAF3 | SETIT_005743mg | TGACG-motif | TGACG | 1194 | 1204 | MeJA response regulatory element |
| SiRAF3 | SETIT_005743mg | TGACG-motif | TGACG | 1217 | 1227 | MeJA response regulatory element |
| SiRAF3 | SETIT_005743mg | TGACG-motif | TGACG | 1672 | 1682 | MeJA response regulatory element |
| SiRAF3 | SETIT_005743mg | TGACG-motif | TGACG | 1749 | 1759 | MeJA response regulatory element |
| SiRAF3 | SETIT_005743mg | CAT-box | GCCACT | 201 | 213 | meristem expression regulatory element |
| SiRAF3 | SETIT_005743mg | MBS | CAACTG | 1820 | 1832 | MYB binding site involved in drought-inducibility |
| SiRAF3 | SETIT_005743mg | CCAAT-box | CAACGG | 1113 | 1125 | MYBHv1 binding site |
| SiRAF3 | SETIT_005743mg | A-box | CCGTCC | 754 | 766 | promoter and enhancer cis-acting regulatory elements |
| SiRAF4 | SETIT_016157mg | ABRE | TACGGTC | 81 | 95 | abscisic acid response element |
| SiRAF4 | SETIT_016157mg | ABRE | ACGTG | 503 | 513 | abscisic acid response element |
| SiRAF4 | SETIT_016157mg | ABRE | ACGTG | 563 | 573 | abscisic acid response element |
| SiRAF4 | SETIT_016157mg | AuxRR-core | GGTCCAT | 1545 | 1559 | auxin response element |
| SiRAF4 | SETIT_016157mg | TGA-element | AACGAC | 1799 | 1811 | auxin-responsive element |
| SiRAF4 | SETIT_016157mg | CAAT-box | CAAAT | 139 | 149 | common cis-acting element |
| SiRAF4 | SETIT_016157mg | CAAT-box | CAAAT | 362 | 372 | common cis-acting element |
| SiRAF4 | SETIT_016157mg | CAAT-box | CAAAT | 725 | 735 | common cis-acting element |
| SiRAF4 | SETIT_016157mg | CAAT-box | CAAAT | 777 | 787 | common cis-acting element |
| SiRAF4 | SETIT_016157mg | CAAT-box | CAAAT | 1468 | 1478 | common cis-acting element |
| SiRAF4 | SETIT_016157mg | CAAT-box | CCAAT | 1599 | 1609 | common cis-acting element |
| SiRAF4 | SETIT_016157mg | CAAT-box | CAAAT | 1643 | 1653 | common cis-acting element |
| SiRAF4 | SETIT_016157mg | TATA-box | TACAAAA | 292 | 306 | core promoter element |
| SiRAF4 | SETIT_016157mg | TATA-box | TATAAAA | 458 | 472 | core promoter element |
| SiRAF4 | SETIT_016157mg | TATA-box | TATAAA | 460 | 472 | core promoter element |
| SiRAF4 | SETIT_016157mg | TATA-box | TATAA | 462 | 472 | core promoter element |
| SiRAF4 | SETIT_016157mg | TATA-box | TATA | 464 | 472 | core promoter element |
| SiRAF4 | SETIT_016157mg | TATA-box | TACAAAA | 643 | 657 | core promoter element |
| SiRAF4 | SETIT_016157mg | TATA-box | ATTATA | 1462 | 1474 | core promoter element |
| SiRAF4 | SETIT_016157mg | TATA-box | TATAA | 1464 | 1474 | core promoter element |
| SiRAF4 | SETIT_016157mg | TATA-box | TATA | 1466 | 1474 | core promoter element |
| SiRAF4 | SETIT_016157mg | TATC-box | TATCCCA | 1127 | 1141 | gibberellin response element |
| SiRAF4 | SETIT_016157mg | P-box | CCTTTTG | 67 | 81 | gibberellin-responsive element |
| SiRAF4 | SETIT_016157mg | GC-motif | CCCCCG | 870 | 882 | hypoxia-specifically induced elements |
| SiRAF4 | SETIT_016157mg | GC-motif | CCCCCG | 1002 | 1014 | hypoxia-specifically induced elements |
| SiRAF4 | SETIT_016157mg | G-box | CACGAC | 30 | 42 | light responsive element |
| SiRAF4 | SETIT_016157mg | G-Box | CACGTT | 501 | 513 | light responsive element |
| SiRAF4 | SETIT_016157mg | G-Box | CACGTT | 561 | 573 | light responsive element |
| SiRAF4 | SETIT_016157mg | G-Box | TCCACATGGCA | 972 | 992 | light responsive element |
| SiRAF4 | SETIT_016157mg | Sp1 | GGGCGG | 892 | 904 | light responsive element |
| SiRAF4 | SETIT_016157mg | Sp1 | GGGCGG | 1005 | 1017 | light responsive element |
| SiRAF4 | SETIT_016157mg | GT1-motif | GGTTAAT | 1649 | 1663 | light responsive element |
| SiRAF4 | SETIT_016157mg | GT1-motif | GGTTAA | 1651 | 1663 | light responsive element |
| SiRAF4 | SETIT_016157mg | Box 4 | ATTAAT | 403 | 415 | light responsive element |
| SiRAF4 | SETIT_016157mg | Box 4 | ATTAAT | 1594 | 1606 | light responsive element |
| SiRAF4 | SETIT_016157mg | L-box | ATCCCACCTAC | 1414 | 1434 | light responsive element |
| SiRAF4 | SETIT_016157mg | I-box | TGATAATGT | 361 | 379 | light responsive element |
| SiRAF4 | SETIT_016157mg | I-box | TAGATAACC | 735 | 753 | light responsive element |
| SiRAF4 | SETIT_016157mg | TCT-motif | TCTTAC | 148 | 160 | light responsive element |
| SiRAF4 | SETIT_016157mg | GATT-motif | CTCCTGATTAGC | 479 | 501 | light responsive element |
| SiRAF4 | SETIT_016157mg | LTR | CCGAAA | 1818 | 1830 | low temperature response element |
| SiRAF4 | SETIT_016157mg | TGACG-motif | TGACG | 1285 | 1295 | MeJA response regulatory element |
| SiRAF4 | SETIT_016157mg | TGACG-motif | TGACG | 1888 | 1898 | MeJA response regulatory element |
| SiRAF4 | SETIT_016157mg | CGTCA-motif | CGTCA | 1285 | 1295 | MeJA response regulatory element |
| SiRAF4 | SETIT_016157mg | CGTCA-motif | CGTCA | 1888 | 1898 | MeJA response regulatory element |
| SiRAF4 | SETIT_016157mg | CAT-box | GCCACT | 1096 | 1108 | meristem expression regulatory element |
| SiRAF4 | SETIT_016157mg | CAT-box | GCCACT | 1823 | 1835 | meristem expression regulatory element |
| SiRAF4 | SETIT_016157mg | CAT-box | GCCACT | 1891 | 1903 | meristem expression regulatory element |
| SiRAF4 | SETIT_016157mg | MRE | AACCTAA | 694 | 708 | MYB binding site involved in light responsiveness |
| SiRAF4 | SETIT_016157mg | CCAAT-box | CAACGG | 1532 | 1544 | MYBHv1 binding site |
| SiRAF4 | SETIT_016157mg | A-box | CCGTCC | 1737 | 1749 | promoter and enhancer cis-acting regulatory elements |
| SiRAF4 | SETIT_016157mg | TCA-element | TCAGAAGAGG | 796 | 814 | salicylic acid response element |
| SiRAF4 | SETIT_016157mg | O2-site | GATGA(C/T)(A/G)TG(A/G) | 90 | 106 | Zein metabolism regulatory elements |
| SiRAF4 | SETIT_016157mg | O2-site | GATGATGTGG | 362 | 380 | Zein metabolism regulatory elements |
| SiRAF5 | SETIT_034063mg | ABRE | ACGTG | 670 | 680 | abscisic acid response element |
| SiRAF5 | SETIT_034063mg | ABRE | GACACGTGGC | 1486 | 1504 | abscisic acid response element |
| SiRAF5 | SETIT_034063mg | ABRE | ACGTG | 1598 | 1608 | abscisic acid response element |
| SiRAF5 | SETIT_034063mg | ARE | AAACCA | 537 | 549 | anaerobic inducing element |
| SiRAF5 | SETIT_034063mg | ARE | AAACCA | 1671 | 1683 | anaerobic inducing element |
| SiRAF5 | SETIT_034063mg | AT-rich element | ATAGAAATCAA | 1085 | 1105 | ATBP-1 binding site |
| SiRAF5 | SETIT_034063mg | TGA-element | AACGAC | 517 | 529 | auxin-responsive element |
| SiRAF5 | SETIT_034063mg | CAAT-box | CCAAT | 646 | 656 | common cis-acting element |
| SiRAF5 | SETIT_034063mg | CAAT-box | CAAAT | 651 | 661 | common cis-acting element |
| SiRAF5 | SETIT_034063mg | CAAT-box | CCAAT | 1000 | 1010 | common cis-acting element |
| SiRAF5 | SETIT_034063mg | CAAT-box | CCAAT | 1008 | 1018 | common cis-acting element |
| SiRAF5 | SETIT_034063mg | CAAT-box | CCAAT | 1024 | 1034 | common cis-acting element |
| SiRAF5 | SETIT_034063mg | CAAT-box | CAAAT | 1173 | 1183 | common cis-acting element |
| SiRAF5 | SETIT_034063mg | CAAT-box | CAAAT | 1342 | 1352 | common cis-acting element |
| SiRAF5 | SETIT_034063mg | CAAT-box | CAAAT | 1373 | 1383 | common cis-acting element |
| SiRAF5 | SETIT_034063mg | CAAT-box | CAAAT | 1455 | 1465 | common cis-acting element |
| SiRAF5 | SETIT_034063mg | TATA-box | TATA | 167 | 175 | core promoter element |
| SiRAF5 | SETIT_034063mg | TATA-box | TATA | 989 | 997 | core promoter element |
| SiRAF5 | SETIT_034063mg | TATA-box | ATATAA | 1002 | 1014 | core promoter element |
| SiRAF5 | SETIT_034063mg | TATA-box | TATA | 1005 | 1013 | core promoter element |
| SiRAF5 | SETIT_034063mg | TATA-box | TATAA | 1196 | 1206 | core promoter element |
| SiRAF5 | SETIT_034063mg | TATA-box | TATA | 1198 | 1206 | core promoter element |
| SiRAF5 | SETIT_034063mg | TC-rich repeats | GTTTTCTTAC | 517 | 535 | defense and stress response elements |
| SiRAF5 | SETIT_034063mg | P-box | CCTTTTG | 1278 | 1292 | gibberellin-responsive element |
| SiRAF5 | SETIT_034063mg | GC-motif | CCCCCG | 1532 | 1544 | hypoxia-specifically induced elements |
| SiRAF5 | SETIT_034063mg | GC-motif | CCCCCG | 1556 | 1568 | hypoxia-specifically induced elements |
| SiRAF5 | SETIT_034063mg | GC-motif | CCCCCG | 1616 | 1628 | hypoxia-specifically induced elements |
| SiRAF5 | SETIT_034063mg | GC-motif | CCCCCG | 1741 | 1753 | hypoxia-specifically induced elements |
| SiRAF5 | SETIT_034063mg | GC-motif | CCCCCG | 1851 | 1863 | hypoxia-specifically induced elements |
| SiRAF5 | SETIT_034063mg | G-box | TACGTG | 668 | 680 | light responsive element |
| SiRAF5 | SETIT_034063mg | G-box | CACGTC | 1597 | 1609 | light responsive element |
| SiRAF5 | SETIT_034063mg | GT1-motif | GGTTAAT | 1661 | 1675 | light responsive element |
| SiRAF5 | SETIT_034063mg | GT1-motif | GGTTAA | 1663 | 1675 | light responsive element |
| SiRAF5 | SETIT_034063mg | Sp1 | GGGCGG | 745 | 757 | light responsive element |
| SiRAF5 | SETIT_034063mg | Sp1 | GGGCGG | 1581 | 1593 | light responsive element |
| SiRAF5 | SETIT_034063mg | Sp1 | GGGCGG | 1819 | 1831 | light responsive element |
| SiRAF5 | SETIT_034063mg | Sp1 | GGGCGG | 1914 | 1926 | light responsive element |
| SiRAF5 | SETIT_034063mg | Sp1 | GGGCGG | 1963 | 1975 | light responsive element |
| SiRAF5 | SETIT_034063mg | Sp1 | GGGCGG | 1968 | 1980 | light responsive element |
| SiRAF5 | SETIT_034063mg | ATCT-motif | AATCTAATCC | 1631 | 1649 | light responsive element |
| SiRAF5 | SETIT_034063mg | ATC-motif | TGCTATCCA | 1269 | 1287 | light responsive element |
| SiRAF5 | SETIT_034063mg | ATC-motif | AGTAATCT | 1346 | 1362 | light responsive element |
| SiRAF5 | SETIT_034063mg | CGTCA-motif | CGTCA | 150 | 160 | MeJA response regulatory element |
| SiRAF5 | SETIT_034063mg | CGTCA-motif | CGTCA | 1425 | 1435 | MeJA response regulatory element |
| SiRAF5 | SETIT_034063mg | CGTCA-motif | CGTCA | 1872 | 1882 | MeJA response regulatory element |
| SiRAF5 | SETIT_034063mg | TGACG-motif | TGACG | 150 | 160 | MeJA response regulatory element |
| SiRAF5 | SETIT_034063mg | TGACG-motif | TGACG | 1425 | 1435 | MeJA response regulatory element |
| SiRAF5 | SETIT_034063mg | TGACG-motif | TGACG | 1872 | 1882 | MeJA response regulatory element |
| SiRAF5 | SETIT_034063mg | CAT-box | GCCACT | 397 | 409 | meristem expression regulatory element |
| SiRAF5 | SETIT_034063mg | MBS | CAACTG | 112 | 124 | MYB binding site involved in drought-inducibility |
| SiRAF5 | SETIT_034063mg | MBS | CAACTG | 417 | 429 | MYB binding site involved in drought-inducibility |
| SiRAF5 | SETIT_034063mg | MRE | AACCTAA | 1395 | 1409 | MYB binding site involved in light responsiveness |
| SiRAF5 | SETIT_034063mg | CCAAT-box | CAACGG | 1854 | 1866 | MYBHv1 binding site |
| SiRAF6 | SETIT_005733mg | ABRE | ACGTG | 318 | 328 | abscisic acid response element |
| SiRAF6 | SETIT_005733mg | ABRE | ACGTG | 465 | 475 | abscisic acid response element |
| SiRAF6 | SETIT_005733mg | ABRE | TACGGTC | 559 | 573 | abscisic acid response element |
| SiRAF6 | SETIT_005733mg | ABRE | ACGTG | 631 | 641 | abscisic acid response element |
| SiRAF6 | SETIT_005733mg | ABRE | ACGTG | 644 | 654 | abscisic acid response element |
| SiRAF6 | SETIT_005733mg | ABRE | CGCACGTGTC | 1018 | 1036 | abscisic acid response element |
| SiRAF6 | SETIT_005733mg | ABRE | ACGTG | 1025 | 1035 | abscisic acid response element |
| SiRAF6 | SETIT_005733mg | ABRE | CACGTG | 1221 | 1233 | abscisic acid response element |
| SiRAF6 | SETIT_005733mg | ABRE | ACGTG | 1223 | 1233 | abscisic acid response element |
| SiRAF6 | SETIT_005733mg | ABRE | TACGGTC | 1266 | 1280 | abscisic acid response element |
| SiRAF6 | SETIT_005733mg | ABRE | ACGTG | 1350 | 1360 | abscisic acid response element |
| SiRAF6 | SETIT_005733mg | ABRE | ACGTG | 1745 | 1755 | abscisic acid response element |
| SiRAF6 | SETIT_005733mg | ARE | AAACCA | 274 | 286 | anaerobic inducing element |
| SiRAF6 | SETIT_005733mg | ARE | AAACCA | 1612 | 1624 | anaerobic inducing element |
| SiRAF6 | SETIT_005733mg | ARE | AAACCA | 1781 | 1793 | anaerobic inducing element |
| SiRAF6 | SETIT_005733mg | TGA-element | AACGAC | 397 | 409 | auxin-responsive element |
| SiRAF6 | SETIT_005733mg | circadian | CAAAGATATC | 465 | 483 | circadian rhythm control element |
| SiRAF6 | SETIT_005733mg | circadian | CAAAGATATC | 1172 | 1190 | circadian rhythm control element |
| SiRAF6 | SETIT_005733mg | CAAT-box | CCAAT | 59 | 69 | common cis-acting element |
| SiRAF6 | SETIT_005733mg | CAAT-box | CAAAT | 376 | 386 | common cis-acting element |
| SiRAF6 | SETIT_005733mg | CAAT-box | CAAAT | 585 | 595 | common cis-acting element |
| SiRAF6 | SETIT_005733mg | CAAT-box | CAAAT | 639 | 649 | common cis-acting element |
| SiRAF6 | SETIT_005733mg | CAAT-box | CCAAT | 681 | 691 | common cis-acting element |
| SiRAF6 | SETIT_005733mg | CAAT-box | CCAAT | 791 | 801 | common cis-acting element |
| SiRAF6 | SETIT_005733mg | CAAT-box | CAAAT | 1083 | 1093 | common cis-acting element |
| SiRAF6 | SETIT_005733mg | CAAT-box | CAAAT | 1247 | 1257 | common cis-acting element |
| SiRAF6 | SETIT_005733mg | CAAT-box | CAAAT | 1291 | 1301 | common cis-acting element |
| SiRAF6 | SETIT_005733mg | CAAT-box | CCAAT | 1328 | 1338 | common cis-acting element |
| SiRAF6 | SETIT_005733mg | CAAT-box | CAAAT | 1490 | 1500 | common cis-acting element |
| SiRAF6 | SETIT_005733mg | TATA-box | TATA | 37 | 45 | core promoter element |
| SiRAF6 | SETIT_005733mg | TATA-box | TATA | 374 | 382 | core promoter element |
| SiRAF6 | SETIT_005733mg | TATA-box | TATA | 395 | 403 | core promoter element |
| SiRAF6 | SETIT_005733mg | TATA-box | TATA | 441 | 449 | core promoter element |
| SiRAF6 | SETIT_005733mg | TATA-box | TATA | 767 | 775 | core promoter element |
| SiRAF6 | SETIT_005733mg | TATA-box | TATA | 1081 | 1089 | core promoter element |
| SiRAF6 | SETIT_005733mg | TATA-box | TATA | 1148 | 1156 | core promoter element |
| SiRAF6 | SETIT_005733mg | TATA-box | TATA | 1260 | 1268 | core promoter element |
| SiRAF6 | SETIT_005733mg | TATC-box | TATCCCA | -4 | 10 | gibberellin response element |
| SiRAF6 | SETIT_005733mg | GC-motif | CCCCCG | 1696 | 1708 | hypoxia-specifically induced elements |
| SiRAF6 | SETIT_005733mg | GC-motif | CCCCCG | 1885 | 1897 | hypoxia-specifically induced elements |
| SiRAF6 | SETIT_005733mg | G-Box | CACGTT | 316 | 328 | light responsive element |
| SiRAF6 | SETIT_005733mg | G-Box | CACGTT | 630 | 642 | light responsive element |
| SiRAF6 | SETIT_005733mg | G-Box | CACGTT | 1023 | 1035 | light responsive element |
| SiRAF6 | SETIT_005733mg | G-Box | CACGTG | 1221 | 1233 | light responsive element |
| SiRAF6 | SETIT_005733mg | G-Box | CACGTT | 1744 | 1756 | light responsive element |
| SiRAF6 | SETIT_005733mg | G-box | TACGTG | 464 | 476 | light responsive element |
| SiRAF6 | SETIT_005733mg | G-box | TAACACGTAG | 521 | 539 | light responsive element |
| SiRAF6 | SETIT_005733mg | G-box | CACGTC | 642 | 654 | light responsive element |
| SiRAF6 | SETIT_005733mg | G-box | GCCACGTGGA | 1216 | 1234 | light responsive element |
| SiRAF6 | SETIT_005733mg | G-box | CACGTG | 1221 | 1233 | light responsive element |
| SiRAF6 | SETIT_005733mg | G-box | CACGTC | 1348 | 1360 | light responsive element |
| SiRAF6 | SETIT_005733mg | Sp1 | GGGCGG | 1699 | 1711 | light responsive element |
| SiRAF6 | SETIT_005733mg | Sp1 | GGGCGG | 1857 | 1869 | light responsive element |
| SiRAF6 | SETIT_005733mg | I-box | AGATAAGG | 328 | 344 | light responsive element |
| SiRAF6 | SETIT_005733mg | GATA-motif | AAGGATAAGG | 147 | 165 | light responsive element |
| SiRAF6 | SETIT_005733mg | GATA-motif | GATAGGA | 728 | 742 | light responsive element |
| SiRAF6 | SETIT_005733mg | TCCC-motif | TCTCCCT | 1748 | 1762 | light responsive element |
| SiRAF6 | SETIT_005733mg | LTR | CCGAAA | 1599 | 1611 | low temperature response element |
| SiRAF6 | SETIT_005733mg | CGTCA-motif | CGTCA | 258 | 268 | MeJA response regulatory element |
| SiRAF6 | SETIT_005733mg | CGTCA-motif | CGTCA | 432 | 442 | MeJA response regulatory element |
| SiRAF6 | SETIT_005733mg | CGTCA-motif | CGTCA | 642 | 652 | MeJA response regulatory element |
| SiRAF6 | SETIT_005733mg | CGTCA-motif | CGTCA | 1139 | 1149 | MeJA response regulatory element |
| SiRAF6 | SETIT_005733mg | TGACG-motif | TGACG | 258 | 268 | MeJA response regulatory element |
| SiRAF6 | SETIT_005733mg | TGACG-motif | TGACG | 432 | 442 | MeJA response regulatory element |
| SiRAF6 | SETIT_005733mg | TGACG-motif | TGACG | 642 | 652 | MeJA response regulatory element |
| SiRAF6 | SETIT_005733mg | TGACG-motif | TGACG | 1139 | 1149 | MeJA response regulatory element |
| SiRAF6 | SETIT_005733mg | CAT-box | GCCACT | 1538 | 1550 | meristem expression regulatory element |
| SiRAF6 | SETIT_005733mg | MBS | CAACTG | 1454 | 1466 | MYB binding site involved in drought-inducibility |
| SiRAF6 | SETIT_005733mg | CCAAT-box | CAACGG | 487 | 499 | MYBHv1 binding site |
| SiRAF6 | SETIT_005733mg | A-box | CCGTCC | 1710 | 1722 | promoter and enhancer cis-acting regulatory elements |
| SiRAF7 | SETIT_016359mg | ABRE | TACGTGTC | 1601 | 1617 | abscisic acid response element |
| SiRAF7 | SETIT_016359mg | ABRE | ACGTG | 1606 | 1616 | abscisic acid response element |
| SiRAF7 | SETIT_016359mg | CAAT-box | CCAAT | 7 | 17 | common cis-acting element |
| SiRAF7 | SETIT_016359mg | CAAT-box | CCAAT | 378 | 388 | common cis-acting element |
| SiRAF7 | SETIT_016359mg | CAAT-box | CCAAT | 432 | 442 | common cis-acting element |
| SiRAF7 | SETIT_016359mg | CAAT-box | CCAAT | 481 | 491 | common cis-acting element |
| SiRAF7 | SETIT_016359mg | CAAT-box | CAAAT | 495 | 505 | common cis-acting element |
| SiRAF7 | SETIT_016359mg | CAAT-box | CAAAT | 544 | 554 | common cis-acting element |
| SiRAF7 | SETIT_016359mg | CAAT-box | CAAAT | 656 | 666 | common cis-acting element |
| SiRAF7 | SETIT_016359mg | CAAT-box | CAAAT | 783 | 793 | common cis-acting element |
| SiRAF7 | SETIT_016359mg | CAAT-box | CCAAT | 876 | 886 | common cis-acting element |
| SiRAF7 | SETIT_016359mg | CAAT-box | CCAAT | 932 | 942 | common cis-acting element |
| SiRAF7 | SETIT_016359mg | CAAT-box | CCAAT | 948 | 958 | common cis-acting element |
| SiRAF7 | SETIT_016359mg | CAAT-box | CAAAT | 1008 | 1018 | common cis-acting element |
| SiRAF7 | SETIT_016359mg | CAAT-box | CAAAT | 1298 | 1308 | common cis-acting element |
| SiRAF7 | SETIT_016359mg | CAAT-box | CCAAT | 1335 | 1345 | common cis-acting element |
| SiRAF7 | SETIT_016359mg | CAAT-box | CAAAT | 1357 | 1367 | common cis-acting element |
| SiRAF7 | SETIT_016359mg | CAAT-box | CCAAT | 1374 | 1384 | common cis-acting element |
| SiRAF7 | SETIT_016359mg | CAAT-box | CAAAT | 1438 | 1448 | common cis-acting element |
| SiRAF7 | SETIT_016359mg | CAAT-box | CCAAT | 1484 | 1494 | common cis-acting element |
| SiRAF7 | SETIT_016359mg | CAAT-box | CAAAT | 1571 | 1581 | common cis-acting element |
| SiRAF7 | SETIT_016359mg | CAAT-box | CAAAT | 1883 | 1893 | common cis-acting element |
| SiRAF7 | SETIT_016359mg | CAAT-box | CAAAT | 1886 | 1896 | common cis-acting element |
| SiRAF7 | SETIT_016359mg | CAAT-box | CAAAT | 1913 | 1923 | common cis-acting element |
| SiRAF7 | SETIT_016359mg | TATA-box | TATAAA | 407 | 419 | core promoter element |
| SiRAF7 | SETIT_016359mg | TATA-box | TATAA | 409 | 419 | core promoter element |
| SiRAF7 | SETIT_016359mg | TATA-box | TATA | 411 | 419 | core promoter element |
| SiRAF7 | SETIT_016359mg | TATA-box | ATATAT | 686 | 698 | core promoter element |
| SiRAF7 | SETIT_016359mg | TATA-box | TATA | 689 | 697 | core promoter element |
| SiRAF7 | SETIT_016359mg | TATA-box | TATACA | 693 | 705 | core promoter element |
| SiRAF7 | SETIT_016359mg | TATA-box | TATA | 697 | 705 | core promoter element |
| SiRAF7 | SETIT_016359mg | TATA-box | TATAAAT | 1693 | 1707 | core promoter element |
| SiRAF7 | SETIT_016359mg | TATA-box | TATAAA | 1695 | 1707 | core promoter element |
| SiRAF7 | SETIT_016359mg | TATA-box | TATAA | 1697 | 1707 | core promoter element |
| SiRAF7 | SETIT_016359mg | TATA-box | TATA | 1699 | 1707 | core promoter element |
| SiRAF7 | SETIT_016359mg | TATA-box | TATA | 1975 | 1983 | core promoter element |
| SiRAF7 | SETIT_016359mg | GCN4_motif | TGAGTCA | 1649 | 1663 | endosperm expression regulatory element |
| SiRAF7 | SETIT_016359mg | TATC-box | TATCCCA | 876 | 890 | gibberellin response element |
| SiRAF7 | SETIT_016359mg | ACE | GACACGTATG | 1600 | 1618 | light responsive element |
| SiRAF7 | SETIT_016359mg | G-box | CACGAC | 1600 | 1612 | light responsive element |
| SiRAF7 | SETIT_016359mg | G-box | TACGTG | 1605 | 1617 | light responsive element |
| SiRAF7 | SETIT_016359mg | GT1-motif | GGTTAA | 78 | 90 | light responsive element |
| SiRAF7 | SETIT_016359mg | Box 4 | ATTAAT | 871 | 883 | light responsive element |
| SiRAF7 | SETIT_016359mg | Box 4 | ATTAAT | 1058 | 1070 | light responsive element |
| SiRAF7 | SETIT_016359mg | CGTCA-motif | CGTCA | 1193 | 1203 | MeJA response regulatory element |
| SiRAF7 | SETIT_016359mg | TGACG-motif | TGACG | 1193 | 1203 | MeJA response regulatory element |
| SiRAF7 | SETIT_016359mg | MBS | CAACTG | 900 | 912 | MYB binding site involved in drought-inducibility |
| SiRAF7 | SETIT_016359mg | MBS | CAACTG | 1037 | 1049 | MYB binding site involved in drought-inducibility |
| SiRAF7 | SETIT_016359mg | MRE | AACCTAA | 269 | 283 | MYB binding site involved in light responsiveness |
| SiRAF7 | SETIT_016359mg | MRE | AACCTAA | 346 | 360 | MYB binding site involved in light responsiveness |
| SiRAF7 | SETIT_016359mg | O2-site | GTTGACGTGA | 271 | 289 | Zein metabolism regulatory elements |
| SiRAF7 | SETIT_016359mg | O2-site | GATGATGTGG | 1246 | 1264 | Zein metabolism regulatory elements |
| SiRAF8 | SETIT_034087mg | ABRE | ACGTG | 746 | 756 | abscisic acid response element |
| SiRAF8 | SETIT_034087mg | ABRE | ACGTG | 1071 | 1081 | abscisic acid response element |
| SiRAF8 | SETIT_034087mg | ABRE | ACGTG | 1083 | 1093 | abscisic acid response element |
| SiRAF8 | SETIT_034087mg | ABRE | ACGTG | 1170 | 1180 | abscisic acid response element |
| SiRAF8 | SETIT_034087mg | ABRE | TACGGTC | 1201 | 1215 | abscisic acid response element |
| SiRAF8 | SETIT_034087mg | ABRE | TACGGTC | 1340 | 1354 | abscisic acid response element |
| SiRAF8 | SETIT_034087mg | ABRE | CACGTG | 1593 | 1605 | abscisic acid response element |
| SiRAF8 | SETIT_034087mg | ABRE | ACGTG | 1595 | 1605 | abscisic acid response element |
| SiRAF8 | SETIT_034087mg | ARE | AAACCA | 817 | 829 | anaerobic inducing element |
| SiRAF8 | SETIT_034087mg | TGA-element | AACGAC | 1116 | 1128 | auxin-responsive element |
| SiRAF8 | SETIT_034087mg | CAAT-box | CAAAT | 39 | 49 | common cis-acting element |
| SiRAF8 | SETIT_034087mg | CAAT-box | CAAAT | 129 | 139 | common cis-acting element |
| SiRAF8 | SETIT_034087mg | CAAT-box | CAAAT | 367 | 377 | common cis-acting element |
| SiRAF8 | SETIT_034087mg | CAAT-box | CAAAT | 408 | 418 | common cis-acting element |
| SiRAF8 | SETIT_034087mg | CAAT-box | CAAAT | 486 | 496 | common cis-acting element |
| SiRAF8 | SETIT_034087mg | CAAT-box | TGCCAAC | 952 | 966 | common cis-acting element |
| SiRAF8 | SETIT_034087mg | CAAT-box | CAAAT | 974 | 984 | common cis-acting element |
| SiRAF8 | SETIT_034087mg | CAAT-box | CAAAT | 1051 | 1061 | common cis-acting element |
| SiRAF8 | SETIT_034087mg | CAAT-box | TGCCAAC | 1084 | 1098 | common cis-acting element |
| SiRAF8 | SETIT_034087mg | CAAT-box | CCAAT | 1250 | 1260 | common cis-acting element |
| SiRAF8 | SETIT_034087mg | CAAT-box | CAAAT | 1316 | 1326 | common cis-acting element |
| SiRAF8 | SETIT_034087mg | CAAT-box | CCAAT | 1441 | 1451 | common cis-acting element |
| SiRAF8 | SETIT_034087mg | CAAT-box | CCAAT | 1446 | 1456 | common cis-acting element |
| SiRAF8 | SETIT_034087mg | TATA-box | TATACA | 46 | 58 | core promoter element |
| SiRAF8 | SETIT_034087mg | TATA-box | TATA | 50 | 58 | core promoter element |
| SiRAF8 | SETIT_034087mg | TATA-box | ATTATA | 131 | 143 | core promoter element |
| SiRAF8 | SETIT_034087mg | TATA-box | TATAA | 133 | 143 | core promoter element |
| SiRAF8 | SETIT_034087mg | TATA-box | TATA | 135 | 143 | core promoter element |
| SiRAF8 | SETIT_034087mg | TATA-box | TATAAAA | 166 | 180 | core promoter element |
| SiRAF8 | SETIT_034087mg | TATA-box | TATAAA | 168 | 180 | core promoter element |
| SiRAF8 | SETIT_034087mg | TATA-box | TATAA | 170 | 180 | core promoter element |
| SiRAF8 | SETIT_034087mg | TATA-box | TATA | 172 | 180 | core promoter element |
| SiRAF8 | SETIT_034087mg | TATA-box | ATATAA | 181 | 193 | core promoter element |
| SiRAF8 | SETIT_034087mg | TATA-box | TATA | 184 | 192 | core promoter element |
| SiRAF8 | SETIT_034087mg | TATA-box | ATATAA | 197 | 209 | core promoter element |
| SiRAF8 | SETIT_034087mg | TATA-box | TATA | 200 | 208 | core promoter element |
| SiRAF8 | SETIT_034087mg | TATA-box | TACAAAA | 214 | 228 | core promoter element |
| SiRAF8 | SETIT_034087mg | TATA-box | TATA | 268 | 276 | core promoter element |
| SiRAF8 | SETIT_034087mg | TATA-box | TACAAAA | 293 | 307 | core promoter element |
| SiRAF8 | SETIT_034087mg | TATA-box | TATA | 340 | 348 | core promoter element |
| SiRAF8 | SETIT_034087mg | TATA-box | TATAAAA | 376 | 390 | core promoter element |
| SiRAF8 | SETIT_034087mg | TATA-box | TATAAA | 378 | 390 | core promoter element |
| SiRAF8 | SETIT_034087mg | TATA-box | TATAA | 380 | 390 | core promoter element |
| SiRAF8 | SETIT_034087mg | TATA-box | TATA | 382 | 390 | core promoter element |
| SiRAF8 | SETIT_034087mg | TATA-box | ATATAA | 389 | 401 | core promoter element |
| SiRAF8 | SETIT_034087mg | TATA-box | TATA | 392 | 400 | core promoter element |
| SiRAF8 | SETIT_034087mg | TATA-box | TATATAA | 504 | 518 | core promoter element |
| SiRAF8 | SETIT_034087mg | TATA-box | TATATA | 506 | 518 | core promoter element |
| SiRAF8 | SETIT_034087mg | TATA-box | TATA | 510 | 518 | core promoter element |
| SiRAF8 | SETIT_034087mg | TATA-box | taTATAAAtc | 613 | 631 | core promoter element |
| SiRAF8 | SETIT_034087mg | TATA-box | TATA | 620 | 628 | core promoter element |
| SiRAF8 | SETIT_034087mg | TATA-box | ATATAT | 789 | 801 | core promoter element |
| SiRAF8 | SETIT_034087mg | TATA-box | TATA | 792 | 800 | core promoter element |
| SiRAF8 | SETIT_034087mg | TATA-box | ATATAT | 964 | 976 | core promoter element |
| SiRAF8 | SETIT_034087mg | TATA-box | TATA | 967 | 975 | core promoter element |
| SiRAF8 | SETIT_034087mg | TATA-box | TATA | 982 | 990 | core promoter element |
| SiRAF8 | SETIT_034087mg | TATA-box | TATA | 1018 | 1026 | core promoter element |
| SiRAF8 | SETIT_034087mg | TATA-box | TATA | 1098 | 1106 | core promoter element |
| SiRAF8 | SETIT_034087mg | TATA-box | ccTATAAAaa | 1286 | 1304 | core promoter element |
| SiRAF8 | SETIT_034087mg | TATA-box | TATAAAA | 1289 | 1303 | core promoter element |
| SiRAF8 | SETIT_034087mg | TATA-box | TATAAA | 1291 | 1303 | core promoter element |
| SiRAF8 | SETIT_034087mg | TATA-box | TATAA | 1293 | 1303 | core promoter element |
| SiRAF8 | SETIT_034087mg | TATA-box | TATA | 1295 | 1303 | core promoter element |
| SiRAF8 | SETIT_034087mg | TATA-box | ccTATAAAaa | 1317 | 1335 | core promoter element |
| SiRAF8 | SETIT_034087mg | TATA-box | TATAAAA | 1320 | 1334 | core promoter element |
| SiRAF8 | SETIT_034087mg | TATA-box | TATAAA | 1322 | 1334 | core promoter element |
| SiRAF8 | SETIT_034087mg | TATA-box | TATAA | 1324 | 1334 | core promoter element |
| SiRAF8 | SETIT_034087mg | TATA-box | TATA | 1326 | 1334 | core promoter element |
| SiRAF8 | SETIT_034087mg | TATA-box | TACAAAA | 1331 | 1345 | core promoter element |
| SiRAF8 | SETIT_034087mg | TATA-box | TATA | 1350 | 1358 | core promoter element |
| SiRAF8 | SETIT_034087mg | TATA-box | TATA | 1376 | 1384 | core promoter element |
| SiRAF8 | SETIT_034087mg | TATA-box | TATA | 1385 | 1393 | core promoter element |
| SiRAF8 | SETIT_034087mg | TATA-box | TATACA | 1388 | 1400 | core promoter element |
| SiRAF8 | SETIT_034087mg | TATA-box | TATA | 1392 | 1400 | core promoter element |
| SiRAF8 | SETIT_034087mg | TATA-box | TATAA | 1467 | 1477 | core promoter element |
| SiRAF8 | SETIT_034087mg | TATA-box | TATA | 1469 | 1477 | core promoter element |
| SiRAF8 | SETIT_034087mg | TATA-box | ATATAT | 1545 | 1557 | core promoter element |
| SiRAF8 | SETIT_034087mg | TATA-box | TATA | 1548 | 1556 | core promoter element |
| SiRAF8 | SETIT_034087mg | TATA-box | TATACA | 1597 | 1609 | core promoter element |
| SiRAF8 | SETIT_034087mg | TATA-box | TATA | 1601 | 1609 | core promoter element |
| SiRAF8 | SETIT_034087mg | TATA-box | TATA | 1630 | 1638 | core promoter element |
| SiRAF8 | SETIT_034087mg | RY-element | CATGCATG | 657 | 673 | elements involved in seed-specific regulation |
| SiRAF8 | SETIT_034087mg | ACE | CTAACGTATT | 873 | 891 | light responsive element |
| SiRAF8 | SETIT_034087mg | ACE | GACACGTATG | 1182 | 1200 | light responsive element |
| SiRAF8 | SETIT_034087mg | G-Box | CACGTG | 1593 | 1605 | light responsive element |
| SiRAF8 | SETIT_034087mg | G-box | CAGACGTGGCA | 738 | 758 | light responsive element |
| SiRAF8 | SETIT_034087mg | G-box | CACGTC | 744 | 756 | light responsive element |
| SiRAF8 | SETIT_034087mg | G-box | TACGTG | 1069 | 1081 | light responsive element |
| SiRAF8 | SETIT_034087mg | G-box | TACGTG | 1081 | 1093 | light responsive element |
| SiRAF8 | SETIT_034087mg | G-box | TACGTG | 1168 | 1180 | light responsive element |
| SiRAF8 | SETIT_034087mg | G-box | CACGTG | 1593 | 1605 | light responsive element |
| SiRAF8 | SETIT_034087mg | G-box | CACGAC | 1639 | 1651 | light responsive element |
| SiRAF8 | SETIT_034087mg | 3-AF1 binding site | TAAGAGAGGAA | 788 | 808 | light responsive element |
| SiRAF8 | SETIT_034087mg | ATCT-motif | AATCTAATCC | 1439 | 1457 | light responsive element |
| SiRAF8 | SETIT_034087mg | ATCT-motif | AATCTAATCC | 1818 | 1836 | light responsive element |
| SiRAF8 | SETIT_034087mg | Box 4 | ATTAAT | 62 | 74 | light responsive element |
| SiRAF8 | SETIT_034087mg | CGTCA-motif | CGTCA | 26 | 36 | MeJA response regulatory element |
| SiRAF8 | SETIT_034087mg | CGTCA-motif | CGTCA | 1159 | 1169 | MeJA response regulatory element |
| SiRAF8 | SETIT_034087mg | TGACG-motif | TGACG | 26 | 36 | MeJA response regulatory element |
| SiRAF8 | SETIT_034087mg | TGACG-motif | TGACG | 1159 | 1169 | MeJA response regulatory element |
| SiRAF8 | SETIT_034087mg | CAT-box | GCCACT | 861 | 873 | meristem expression regulatory element |
| SiRAF8 | SETIT_034087mg | CAT-box | GCCACT | 1654 | 1666 | meristem expression regulatory element |
| SiRAF8 | SETIT_034087mg | CAT-box | GCCACT | 1733 | 1745 | meristem expression regulatory element |
| SiRAF8 | SETIT_034087mg | CAT-box | GCCACT | 1800 | 1812 | meristem expression regulatory element |
| SiRAF8 | SETIT_034087mg | CAT-box | GCCACT | 1834 | 1846 | meristem expression regulatory element |
| SiRAF8 | SETIT_034087mg | A-box | CCGTCC | 244 | 256 | promoter and enhancer cis-acting regulatory elements |
| SiRAF8 | SETIT_034087mg | A-box | CCGTCC | 606 | 618 | promoter and enhancer cis-acting regulatory elements |
| SiRAF8 | SETIT_034087mg | A-box | CCGTCC | 1683 | 1695 | promoter and enhancer cis-acting regulatory elements |
| SiRAF8 | SETIT_034087mg | SARE | TTCGACCATCTT | 1606 | 1630 | salicylic acid response element |
| SiRAF8 | SETIT_034087mg | O2-site | GATGATGTGG | 722 | 740 | Zein metabolism regulatory elements |
| SiRAF9 | SETIT_021297mg | ABRE | ACGTG | 1116 | 1126 | abscisic acid response element |
| SiRAF9 | SETIT_021297mg | ARE | AAACCA | 109 | 121 | anaerobic inducing element |
| SiRAF9 | SETIT_021297mg | TGA-element | AACGAC | 504 | 516 | auxin-responsive element |
| SiRAF9 | SETIT_021297mg | CAAT-box | CCAAT | 41 | 51 | common cis-acting element |
| SiRAF9 | SETIT_021297mg | CAAT-box | CAAAT | 64 | 74 | common cis-acting element |
| SiRAF9 | SETIT_021297mg | CAAT-box | CAAAT | 463 | 473 | common cis-acting element |
| SiRAF9 | SETIT_021297mg | CAAT-box | CAAAT | 584 | 594 | common cis-acting element |
| SiRAF9 | SETIT_021297mg | CAAT-box | CCAAT | 792 | 802 | common cis-acting element |
| SiRAF9 | SETIT_021297mg | CAAT-box | CCAAT | 1235 | 1245 | common cis-acting element |
| SiRAF9 | SETIT_021297mg | CAAT-box | CCAAT | 1243 | 1253 | common cis-acting element |
| SiRAF9 | SETIT_021297mg | CAAT-box | CCAAT | 1250 | 1260 | common cis-acting element |
| SiRAF9 | SETIT_021297mg | CAAT-box | TGCCAAC | 1284 | 1298 | common cis-acting element |
| SiRAF9 | SETIT_021297mg | CAAT-box | CAAAT | 1376 | 1386 | common cis-acting element |
| SiRAF9 | SETIT_021297mg | CAAT-box | CCAAT | 1516 | 1526 | common cis-acting element |
| SiRAF9 | SETIT_021297mg | TATA-box | TATA | 175 | 183 | core promoter element |
| SiRAF9 | SETIT_021297mg | TATA-box | ATTATA | 191 | 203 | core promoter element |
| SiRAF9 | SETIT_021297mg | TATA-box | TATAA | 193 | 203 | core promoter element |
| SiRAF9 | SETIT_021297mg | TATA-box | TATA | 195 | 203 | core promoter element |
| SiRAF9 | SETIT_021297mg | TATA-box | TATA | 205 | 213 | core promoter element |
| SiRAF9 | SETIT_021297mg | TATA-box | TATA | 309 | 317 | core promoter element |
| SiRAF9 | SETIT_021297mg | TATA-box | ccTATAAAaa | 314 | 332 | core promoter element |
| SiRAF9 | SETIT_021297mg | TATA-box | TATAAAA | 317 | 331 | core promoter element |
| SiRAF9 | SETIT_021297mg | TATA-box | TATAAA | 319 | 331 | core promoter element |
| SiRAF9 | SETIT_021297mg | TATA-box | TATAA | 321 | 331 | core promoter element |
| SiRAF9 | SETIT_021297mg | TATA-box | TATA | 323 | 331 | core promoter element |
| SiRAF9 | SETIT_021297mg | TATA-box | ATATAT | 332 | 344 | core promoter element |
| SiRAF9 | SETIT_021297mg | TATA-box | TATA | 335 | 343 | core promoter element |
| SiRAF9 | SETIT_021297mg | TATA-box | ATATAA | 383 | 395 | core promoter element |
| SiRAF9 | SETIT_021297mg | TATA-box | TATA | 386 | 394 | core promoter element |
| SiRAF9 | SETIT_021297mg | TATA-box | TACAAAA | 391 | 405 | core promoter element |
| SiRAF9 | SETIT_021297mg | TATA-box | TATA | 540 | 548 | core promoter element |
| SiRAF9 | SETIT_021297mg | TATA-box | ATATAT | 549 | 561 | core promoter element |
| SiRAF9 | SETIT_021297mg | TATA-box | TATA | 552 | 560 | core promoter element |
| SiRAF9 | SETIT_021297mg | TATA-box | TATA | 631 | 639 | core promoter element |
| SiRAF9 | SETIT_021297mg | TATA-box | TATAA | 707 | 717 | core promoter element |
| SiRAF9 | SETIT_021297mg | TATA-box | TATA | 709 | 717 | core promoter element |
| SiRAF9 | SETIT_021297mg | TATA-box | TATA | 884 | 892 | core promoter element |
| SiRAF9 | SETIT_021297mg | TATA-box | TATA | 993 | 1001 | core promoter element |
| SiRAF9 | SETIT_021297mg | TATA-box | ATTATA | 1359 | 1371 | core promoter element |
| SiRAF9 | SETIT_021297mg | TATA-box | TATAA | 1361 | 1371 | core promoter element |
| SiRAF9 | SETIT_021297mg | TATA-box | TATA | 1363 | 1371 | core promoter element |
| SiRAF9 | SETIT_021297mg | TATA-box | TACATAAA | 1449 | 1465 | core promoter element |
| SiRAF9 | SETIT_021297mg | TATA-box | TATATA | 1576 | 1588 | core promoter element |
| SiRAF9 | SETIT_021297mg | TATA-box | ATATAT | 1577 | 1589 | core promoter element |
| SiRAF9 | SETIT_021297mg | TATA-box | TATATA | 1578 | 1590 | core promoter element |
| SiRAF9 | SETIT_021297mg | TATA-box | ATATAT | 1579 | 1591 | core promoter element |
| SiRAF9 | SETIT_021297mg | TATA-box | TATA | 1582 | 1590 | core promoter element |
| SiRAF9 | SETIT_021297mg | P-box | CCTTTTG | 1002 | 1016 | gibberellin-responsive element |
| SiRAF9 | SETIT_021297mg | G-box | GCCACGTGGA | 727 | 745 | light responsive element |
| SiRAF9 | SETIT_021297mg | G-box | TACGTG | 1115 | 1127 | light responsive element |
| SiRAF9 | SETIT_021297mg | Sp1 | GGGCGG | 1911 | 1923 | light responsive element |
| SiRAF9 | SETIT_021297mg | Box 4 | ATTAAT | 176 | 188 | light responsive element |
| SiRAF9 | SETIT_021297mg | Box 4 | ATTAAT | 405 | 417 | light responsive element |
| SiRAF9 | SETIT_021297mg | GATA-motif | GATAGGG | 1309 | 1323 | light responsive element |
| SiRAF9 | SETIT_021297mg | I-box | cCATATCCAAT | 1564 | 1584 | light responsive element |
| SiRAF9 | SETIT_021297mg | TCCC-motif | TCTCCCT | 1672 | 1686 | light responsive element |
| SiRAF9 | SETIT_021297mg | AE-box | AGAAACAA | 634 | 650 | light responsive element |
| SiRAF9 | SETIT_021297mg | LTR | CCGAAA | 941 | 953 | low temperature response element |
| SiRAF9 | SETIT_021297mg | LTR | CCGAAA | 1138 | 1150 | low temperature response element |
| SiRAF9 | SETIT_021297mg | CGTCA-motif | CGTCA | 10 | 20 | MeJA response regulatory element |
| SiRAF9 | SETIT_021297mg | TGACG-motif | TGACG | 10 | 20 | MeJA response regulatory element |
| SiRAF9 | SETIT_021297mg | MBS | CAACTG | 136 | 148 | MYB binding site involved in drought-inducibility |
| SiRAF9 | SETIT_021297mg | MBS | CAACTG | 975 | 987 | MYB binding site involved in drought-inducibility |
| SiRAF9 | SETIT_021297mg | MBS | CAACTG | 1095 | 1107 | MYB binding site involved in drought-inducibility |
| SiRAF9 | SETIT_021297mg | O2-site | GATGACATGG | 430 | 448 | Zein metabolism regulatory elements |
| SiRAF10 | SETIT_029013mg | ABRE | ACGTG | 14 | 24 | abscisic acid response element |
| SiRAF10 | SETIT_029013mg | ABRE | ACGTG | 1780 | 1790 | abscisic acid response element |
| SiRAF10 | SETIT_029013mg | ARE | AAACCA | 1470 | 1482 | anaerobic inducing element |
| SiRAF10 | SETIT_029013mg | CAAT-box | CAAAT | 101 | 111 | common cis-acting element |
| SiRAF10 | SETIT_029013mg | CAAT-box | CAAAT | 168 | 178 | common cis-acting element |
| SiRAF10 | SETIT_029013mg | CAAT-box | CCAAT | 783 | 793 | common cis-acting element |
| SiRAF10 | SETIT_029013mg | CAAT-box | TGCCAAC | 945 | 959 | common cis-acting element |
| SiRAF10 | SETIT_029013mg | CAAT-box | CAAAT | 1205 | 1215 | common cis-acting element |
| SiRAF10 | SETIT_029013mg | CAAT-box | CAAAT | 1232 | 1242 | common cis-acting element |
| SiRAF10 | SETIT_029013mg | CAAT-box | CAAAT | 1270 | 1280 | common cis-acting element |
| SiRAF10 | SETIT_029013mg | CAAT-box | CAAAT | 1427 | 1437 | common cis-acting element |
| SiRAF10 | SETIT_029013mg | CAAT-box | CAAAT | 1457 | 1467 | common cis-acting element |
| SiRAF10 | SETIT_029013mg | TATA-box | TATAA | 75 | 85 | core promoter element |
| SiRAF10 | SETIT_029013mg | TATA-box | TATA | 77 | 85 | core promoter element |
| SiRAF10 | SETIT_029013mg | TATA-box | TATAA | 90 | 100 | core promoter element |
| SiRAF10 | SETIT_029013mg | TATA-box | TATA | 92 | 100 | core promoter element |
| SiRAF10 | SETIT_029013mg | TATA-box | TATAA | 134 | 144 | core promoter element |
| SiRAF10 | SETIT_029013mg | TATA-box | TATA | 136 | 144 | core promoter element |
| SiRAF10 | SETIT_029013mg | TATA-box | TATAA | 146 | 156 | core promoter element |
| SiRAF10 | SETIT_029013mg | TATA-box | TATA | 148 | 156 | core promoter element |
| SiRAF10 | SETIT_029013mg | TATA-box | ATTATA | 1129 | 1141 | core promoter element |
| SiRAF10 | SETIT_029013mg | TATA-box | TATAA | 1131 | 1141 | core promoter element |
| SiRAF10 | SETIT_029013mg | TATA-box | TATA | 1133 | 1141 | core promoter element |
| SiRAF10 | SETIT_029013mg | TATA-box | TATA | 1156 | 1164 | core promoter element |
| SiRAF10 | SETIT_029013mg | TATA-box | TACAAAA | 1295 | 1309 | core promoter element |
| SiRAF10 | SETIT_029013mg | TATA-box | ATTATA | 1308 | 1320 | core promoter element |
| SiRAF10 | SETIT_029013mg | TATA-box | TATAA | 1310 | 1320 | core promoter element |
| SiRAF10 | SETIT_029013mg | TATA-box | TATA | 1312 | 1320 | core promoter element |
| SiRAF10 | SETIT_029013mg | TATA-box | TATAA | 1488 | 1498 | core promoter element |
| SiRAF10 | SETIT_029013mg | TATA-box | TATA | 1490 | 1498 | core promoter element |
| SiRAF10 | SETIT_029013mg | GC-motif | CCCCCG | 263 | 275 | hypoxia-specifically induced elements |
| SiRAF10 | SETIT_029013mg | GC-motif | CCCCCG | 735 | 747 | hypoxia-specifically induced elements |
| SiRAF10 | SETIT_029013mg | GC-motif | CCCCCG | 798 | 810 | hypoxia-specifically induced elements |
| SiRAF10 | SETIT_029013mg | G-box | CACGTC | 13 | 25 | light responsive element |
| SiRAF10 | SETIT_029013mg | G-box | CACGAC | 476 | 488 | light responsive element |
| SiRAF10 | SETIT_029013mg | G-box | CACGAC | 918 | 930 | light responsive element |
| SiRAF10 | SETIT_029013mg | G-box | CACGTC | 1779 | 1791 | light responsive element |
| SiRAF10 | SETIT_029013mg | Sp1 | GGGCGG | 24 | 36 | light responsive element |
| SiRAF10 | SETIT_029013mg | Sp1 | GGGCGG | 332 | 344 | light responsive element |
| SiRAF10 | SETIT_029013mg | Sp1 | GGGCGG | 711 | 723 | light responsive element |
| SiRAF10 | SETIT_029013mg | Sp1 | GGGCGG | 731 | 743 | light responsive element |
| SiRAF10 | SETIT_029013mg | Sp1 | GGGCGG | 1595 | 1607 | light responsive element |
| SiRAF10 | SETIT_029013mg | Sp1 | GGGCGG | 1732 | 1744 | light responsive element |
| SiRAF10 | SETIT_029013mg | Sp1 | GGGCGG | 1739 | 1751 | light responsive element |
| SiRAF10 | SETIT_029013mg | Sp1 | GGGCGG | 1747 | 1759 | light responsive element |
| SiRAF10 | SETIT_029013mg | Sp1 | GGGCGG | 1930 | 1942 | light responsive element |
| SiRAF10 | SETIT_029013mg | Sp1 | GGGCGG | 1974 | 1986 | light responsive element |
| SiRAF10 | SETIT_029013mg | Box 4 | ATTAAT | 1188 | 1200 | light responsive element |
| SiRAF10 | SETIT_029013mg | CAG-motif | GAAAGGCAGAC | 1899 | 1919 | light responsive element |
| SiRAF10 | SETIT_029013mg | GATA-motif | GATAGGG | 1696 | 1710 | light responsive element |
| SiRAF10 | SETIT_029013mg | GTGGC-motif | CAGCGTGTGGC | 1675 | 1695 | light responsive element |
| SiRAF10 | SETIT_029013mg | TCCC-motif | TCTCCCT | 242 | 256 | light responsive element |
| SiRAF10 | SETIT_029013mg | GATT-motif | CTCCTGATTAGC | 1096 | 1118 | light responsive element |
| SiRAF10 | SETIT_029013mg | LTR | CCGAAA | 1891 | 1903 | low temperature response element |
| SiRAF10 | SETIT_029013mg | CGTCA-motif | CGTCA | 16 | 26 | MeJA response regulatory element |
| SiRAF10 | SETIT_029013mg | CGTCA-motif | CGTCA | 877 | 887 | MeJA response regulatory element |
| SiRAF10 | SETIT_029013mg | CGTCA-motif | CGTCA | 1782 | 1792 | MeJA response regulatory element |
| SiRAF10 | SETIT_029013mg | TGACG-motif | TGACG | 16 | 26 | MeJA response regulatory element |
| SiRAF10 | SETIT_029013mg | TGACG-motif | TGACG | 877 | 887 | MeJA response regulatory element |
| SiRAF10 | SETIT_029013mg | TGACG-motif | TGACG | 1782 | 1792 | MeJA response regulatory element |
| SiRAF10 | SETIT_029013mg | CAT-box | GCCACT | 1609 | 1621 | meristem expression regulatory element |
| SiRAF10 | SETIT_029013mg | CAT-box | GCCACT | 1688 | 1700 | meristem expression regulatory element |
| SiRAF10 | SETIT_029013mg | O2-site | GTTGACGTGA | 9 | 27 | Zein metabolism regulatory elements |
| SiRAF11 | SETIT_016333mg | ABRE | ACGTG | 7 | 17 | abscisic acid response element |
| SiRAF11 | SETIT_016333mg | ABRE | ACGTG | 1275 | 1285 | abscisic acid response element |
| SiRAF11 | SETIT_016333mg | ABRE | ACGTG | 1370 | 1380 | abscisic acid response element |
| SiRAF11 | SETIT_016333mg | ABRE | ACGTG | 1488 | 1498 | abscisic acid response element |
| SiRAF11 | SETIT_016333mg | ABRE | ACGTG | 1502 | 1512 | abscisic acid response element |
| SiRAF11 | SETIT_016333mg | ABRE | GCCGCGTGGC | 1758 | 1776 | abscisic acid response element |
| SiRAF11 | SETIT_016333mg | TGA-element | AACGAC | 1099 | 1111 | auxin-responsive element |
| SiRAF11 | SETIT_016333mg | circadian | CAAAGATATC | 126 | 144 | circadian rhythm control element |
| SiRAF11 | SETIT_016333mg | CAAT-box | CCAAT | 473 | 483 | common cis-acting element |
| SiRAF11 | SETIT_016333mg | CAAT-box | CAAAT | 536 | 546 | common cis-acting element |
| SiRAF11 | SETIT_016333mg | CAAT-box | CAAAT | 558 | 568 | common cis-acting element |
| SiRAF11 | SETIT_016333mg | CAAT-box | CCAAT | 621 | 631 | common cis-acting element |
| SiRAF11 | SETIT_016333mg | CAAT-box | CCAAT | 692 | 702 | common cis-acting element |
| SiRAF11 | SETIT_016333mg | CAAT-box | CAAAT | 831 | 841 | common cis-acting element |
| SiRAF11 | SETIT_016333mg | CAAT-box | CAAAT | 872 | 882 | common cis-acting element |
| SiRAF11 | SETIT_016333mg | CAAT-box | CAAAT | 875 | 885 | common cis-acting element |
| SiRAF11 | SETIT_016333mg | CAAT-box | CAAAT | 911 | 921 | common cis-acting element |
| SiRAF11 | SETIT_016333mg | CAAT-box | CAAAT | 933 | 943 | common cis-acting element |
| SiRAF11 | SETIT_016333mg | CAAT-box | CCAAT | 1024 | 1034 | common cis-acting element |
| SiRAF11 | SETIT_016333mg | CAAT-box | TGCCAAC | 1546 | 1560 | common cis-acting element |
| SiRAF11 | SETIT_016333mg | TATA-box | TATA | 464 | 472 | core promoter element |
| SiRAF11 | SETIT_016333mg | TATA-box | TATA | 1486 | 1494 | core promoter element |
| SiRAF11 | SETIT_016333mg | GC-motif | CCCCCG | 1565 | 1577 | hypoxia-specifically induced elements |
| SiRAF11 | SETIT_016333mg | G-box | TAACACGTAG | 0 | 18 | light responsive element |
| SiRAF11 | SETIT_016333mg | G-box | TACGTG | 6 | 18 | light responsive element |
| SiRAF11 | SETIT_016333mg | G-box | TAACACGTAG | 1268 | 1288 | light responsive element |
| SiRAF11 | SETIT_016333mg | G-box | TACGTG | 1273 | 1285 | light responsive element |
| SiRAF11 | SETIT_016333mg | G-box | TACGTG | 1369 | 1381 | light responsive element |
| SiRAF11 | SETIT_016333mg | G-box | TACGTG | 1486 | 1498 | light responsive element |
| SiRAF11 | SETIT_016333mg | G-box | CACGTC | 1501 | 1513 | light responsive element |
| SiRAF11 | SETIT_016333mg | Sp1 | GGGCGG | 1203 | 1215 | light responsive element |
| SiRAF11 | SETIT_016333mg | Sp1 | GGGCGG | 1463 | 1475 | light responsive element |
| SiRAF11 | SETIT_016333mg | Sp1 | GGGCGG | 1531 | 1543 | light responsive element |
| SiRAF11 | SETIT_016333mg | Sp1 | GGGCGG | 1535 | 1547 | light responsive element |
| SiRAF11 | SETIT_016333mg | Sp1 | GGGCGG | 1687 | 1699 | light responsive element |
| SiRAF11 | SETIT_016333mg | Sp1 | GGGCGG | 1873 | 1885 | light responsive element |
| SiRAF11 | SETIT_016333mg | 3-AF1 binding site | TAAGAGAGGAA | 237 | 257 | light responsive element |
| SiRAF11 | SETIT_016333mg | GT1-motif | GGTTAA | 56 | 68 | light responsive element |
| SiRAF11 | SETIT_016333mg | ATCT-motif | AATCTAATCC | 1399 | 1417 | light responsive element |
| SiRAF11 | SETIT_016333mg | Box 4 | ATTAAT | 102 | 114 | light responsive element |
| SiRAF11 | SETIT_016333mg | Box 4 | ATTAAT | 955 | 967 | light responsive element |
| SiRAF11 | SETIT_016333mg | Box II | ACACGTAGA | 1268 | 1286 | light responsive element |
| SiRAF11 | SETIT_016333mg | chs-CMA1a | TTACTTAA | 109 | 125 | light responsive element |
| SiRAF11 | SETIT_016333mg | TCCC-motif | TCTCCCT | 1169 | 1183 | light responsive element |
| SiRAF11 | SETIT_016333mg | TCT-motif | TCTTAC | 247 | 259 | light responsive element |
| SiRAF11 | SETIT_016333mg | AE-box | AGAAACAA | 285 | 301 | light responsive element |
| SiRAF11 | SETIT_016333mg | AE-box | AGAAACAA | 325 | 341 | light responsive element |
| SiRAF11 | SETIT_016333mg | LTR | CCGAAA | 1052 | 1064 | low temperature response element |
| SiRAF11 | SETIT_016333mg | TGACG-motif | TGACG | 819 | 829 | MeJA response regulatory element |
| SiRAF11 | SETIT_016333mg | TGACG-motif | TGACG | 1328 | 1338 | MeJA response regulatory element |
| SiRAF11 | SETIT_016333mg | TGACG-motif | TGACG | 1601 | 1611 | MeJA response regulatory element |
| SiRAF11 | SETIT_016333mg | CGTCA-motif | CGTCA | 819 | 829 | MeJA response regulatory element |
| SiRAF11 | SETIT_016333mg | CGTCA-motif | CGTCA | 1328 | 1338 | MeJA response regulatory element |
| SiRAF11 | SETIT_016333mg | CGTCA-motif | CGTCA | 1601 | 1611 | MeJA response regulatory element |
| SiRAF11 | SETIT_016333mg | CAT-box | GCCACT | 123 | 135 | meristem expression regulatory element |
| SiRAF11 | SETIT_016333mg | CAT-box | GCCACT | 1560 | 1572 | meristem expression regulatory element |
| SiRAF11 | SETIT_016333mg | CCAAT-box | CAACGG | 1608 | 1620 | MYBHv1 binding site |
| SiRAF11 | SETIT_016333mg | A-box | CCGTCC | 1848 | 1860 | promoter and enhancer cis-acting regulatory elements |
| SiRAF11 | SETIT_016333mg | NON-box | AGATCGACG | 1948 | 1966 | regulatory elements related to specific activation of meristems |
| SiRAF12 | SETIT_009672mg | ABRE | CACGTG | 417 | 429 | abscisic acid response element |
| SiRAF12 | SETIT_009672mg | ABRE | ACGTG | 419 | 429 | abscisic acid response element |
| SiRAF12 | SETIT_009672mg | ABRE | CGCACGTGTC | 1037 | 1055 | abscisic acid response element |
| SiRAF12 | SETIT_009672mg | ABRE | CACGTG | 1042 | 1054 | abscisic acid response element |
| SiRAF12 | SETIT_009672mg | ABRE | ACGTG | 1044 | 1054 | abscisic acid response element |
| SiRAF12 | SETIT_009672mg | ABRE | CACGTG | 1819 | 1831 | abscisic acid response element |
| SiRAF12 | SETIT_009672mg | ABRE | ACGTG | 1821 | 1831 | abscisic acid response element |
| SiRAF12 | SETIT_009672mg | ARE | AAACCA | 1571 | 1583 | anaerobic inducing element |
| SiRAF12 | SETIT_009672mg | AuxRR-core | GGTCCAT | 1617 | 1631 | auxin response element |
| SiRAF12 | SETIT_009672mg | CAAT-box | CAAAT | 551 | 561 | common cis-acting element |
| SiRAF12 | SETIT_009672mg | CAAT-box | CCAAT | 696 | 706 | common cis-acting element |
| SiRAF12 | SETIT_009672mg | CAAT-box | CAAAT | 1453 | 1463 | common cis-acting element |
| SiRAF12 | SETIT_009672mg | CAAT-box | CAAAT | 1466 | 1476 | common cis-acting element |
| SiRAF12 | SETIT_009672mg | CAAT-box | CAAAT | 1693 | 1703 | common cis-acting element |
| SiRAF12 | SETIT_009672mg | TATA-box | TATA | 1278 | 1286 | core promoter element |
| SiRAF12 | SETIT_009672mg | TATA-box | TATACA | 1508 | 1520 | core promoter element |
| SiRAF12 | SETIT_009672mg | TATA-box | TATA | 1512 | 1520 | core promoter element |
| SiRAF12 | SETIT_009672mg | TATA-box | TATATA | 1600 | 1612 | core promoter element |
| SiRAF12 | SETIT_009672mg | TATA-box | ATATAT | 1601 | 1613 | core promoter element |
| SiRAF12 | SETIT_009672mg | TATA-box | TATA | 1604 | 1612 | core promoter element |
| SiRAF12 | SETIT_009672mg | GC-motif | CCCCCG | 426 | 438 | hypoxia-specifically induced elements |
| SiRAF12 | SETIT_009672mg | GC-motif | CCCCCG | 446 | 458 | hypoxia-specifically induced elements |
| SiRAF12 | SETIT_009672mg | GC-motif | CCCCCG | 700 | 712 | hypoxia-specifically induced elements |
| SiRAF12 | SETIT_009672mg | GC-motif | CCCCCG | 1128 | 1140 | hypoxia-specifically induced elements |
| SiRAF12 | SETIT_009672mg | GC-motif | CCCCCG | 1164 | 1176 | hypoxia-specifically induced elements |
| SiRAF12 | SETIT_009672mg | G-Box | CACGTG | 417 | 429 | light responsive element |
| SiRAF12 | SETIT_009672mg | G-Box | CACGTG | 1042 | 1054 | light responsive element |
| SiRAF12 | SETIT_009672mg | G-Box | CACGTG | 1819 | 1831 | light responsive element |
| SiRAF12 | SETIT_009672mg | G-box | CACGTG | 417 | 429 | light responsive element |
| SiRAF12 | SETIT_009672mg | G-box | CACGTG | 1042 | 1054 | light responsive element |
| SiRAF12 | SETIT_009672mg | G-box | CACGTG | 1819 | 1831 | light responsive element |
| SiRAF12 | SETIT_009672mg | G-box | CACGAC | 1955 | 1967 | light responsive element |
| SiRAF12 | SETIT_009672mg | GT1-motif | GGTTAA | 781 | 793 | light responsive element |
| SiRAF12 | SETIT_009672mg | GT1-motif | GGTTAAT | 1546 | 1560 | light responsive element |
| SiRAF12 | SETIT_009672mg | Sp1 | GGGCGG | 313 | 325 | light responsive element |
| SiRAF12 | SETIT_009672mg | Sp1 | GGGCGG | 1131 | 1143 | light responsive element |
| SiRAF12 | SETIT_009672mg | Sp1 | GGGCGG | 1136 | 1148 | light responsive element |
| SiRAF12 | SETIT_009672mg | TCT-motif | TCTTAC | 1881 | 1893 | light responsive element |
| SiRAF12 | SETIT_009672mg | TCCC-motif | TCTCCCT | 1082 | 1096 | light responsive element |
| SiRAF12 | SETIT_009672mg | LTR | CCGAAA | 277 | 289 | low temperature response element |
| SiRAF12 | SETIT_009672mg | CGTCA-motif | CGTCA | 1824 | 1834 | MeJA response regulatory element |
| SiRAF12 | SETIT_009672mg | TGACG-motif | TGACG | 1824 | 1834 | MeJA response regulatory element |
| SiRAF12 | SETIT_009672mg | CAT-box | GCCACT | 1594 | 1606 | meristem expression regulatory element |
| SiRAF12 | SETIT_009672mg | MBS | CAACTG | 1840 | 1852 | MYB binding site involved in drought-inducibility |
| SiRAF12 | SETIT_009672mg | MRE | AACCTAA | 586 | 600 | MYB binding site involved in light responsiveness |
| SiRAF12 | SETIT_009672mg | CCAAT-box | CAACGG | 885 | 897 | MYBHv1 binding site |
| SiRAF12 | SETIT_009672mg | CCAAT-box | CAACGG | 1712 | 1724 | MYBHv1 binding site |
| SiRAF12 | SETIT_009672mg | A-box | CCGTCC | 220 | 232 | promoter and enhancer cis-acting regulatory elements |
| SiRAF12 | SETIT_009672mg | A-box | CCGTCC | 460 | 472 | promoter and enhancer cis-acting regulatory elements |
| SiRAF12 | SETIT_009672mg | HD-Zip 3 | GTAAT(G/C)ATTAC | 1405.5 | 1424.5 | protein binding site |
| SiRAF13 | SETIT_034839mg | ABRE | GACACGTGGC | 61 | 79 | abscisic acid response element |
| SiRAF13 | SETIT_034839mg | ABRE | ACGTG | 543 | 553 | abscisic acid response element |
| SiRAF13 | SETIT_034839mg | ABRE | ACGTG | 693 | 703 | abscisic acid response element |
| SiRAF13 | SETIT_034839mg | AuxRR-core | GGTCCAT | 1229 | 1243 | auxin response element |
| SiRAF13 | SETIT_034839mg | AuxRR-core | GGTCCAT | 1440 | 1454 | auxin response element |
| SiRAF13 | SETIT_034839mg | CAAT-box | CAAAT | 183 | 193 | common cis-acting element |
| SiRAF13 | SETIT_034839mg | CAAT-box | CAAAT | 333 | 343 | common cis-acting element |
| SiRAF13 | SETIT_034839mg | CAAT-box | CAAAT | 707 | 717 | common cis-acting element |
| SiRAF13 | SETIT_034839mg | CAAT-box | CAAAT | 740 | 750 | common cis-acting element |
| SiRAF13 | SETIT_034839mg | CAAT-box | CAAAT | 946 | 956 | common cis-acting element |
| SiRAF13 | SETIT_034839mg | CAAT-box | CAAAT | 1176 | 1186 | common cis-acting element |
| SiRAF13 | SETIT_034839mg | CAAT-box | CAAAT | 1254 | 1264 | common cis-acting element |
| SiRAF13 | SETIT_034839mg | CAAT-box | CCAAT | 1315 | 1325 | common cis-acting element |
| SiRAF13 | SETIT_034839mg | CAAT-box | CAAAT | 1591 | 1601 | common cis-acting element |
| SiRAF13 | SETIT_034839mg | CAAT-box | CCAAT | 1800 | 1810 | common cis-acting element |
| SiRAF13 | SETIT_034839mg | CAAT-box | CAAAT | 1863 | 1873 | common cis-acting element |
| SiRAF13 | SETIT_034839mg | TATA-box | TATACA | 1149 | 1161 | core promoter element |
| SiRAF13 | SETIT_034839mg | TATA-box | TATA | 1153 | 1161 | core promoter element |
| SiRAF13 | SETIT_034839mg | G-box | CACGAC | 312 | 324 | light responsive element |
| SiRAF13 | SETIT_034839mg | G-box | CACGTC | 541 | 553 | light responsive element |
| SiRAF13 | SETIT_034839mg | G-box | CACGTC | 692 | 704 | light responsive element |
| SiRAF13 | SETIT_034839mg | TCCC-motif | TCTCCCT | 1421 | 1435 | light responsive element |
| SiRAF13 | SETIT_034839mg | TCCC-motif | TCTCCCT | 1538 | 1552 | light responsive element |
| SiRAF13 | SETIT_034839mg | chs-CMA2a | TCACTTGA | 1164 | 1180 | light responsive element |
| SiRAF13 | SETIT_034839mg | AE-box | AGAAACAA | 1655 | 1671 | light responsive element |
| SiRAF13 | SETIT_034839mg | CGTCA-motif | CGTCA | 89 | 99 | MeJA response regulatory element |
| SiRAF13 | SETIT_034839mg | CGTCA-motif | CGTCA | 117 | 127 | MeJA response regulatory element |
| SiRAF13 | SETIT_034839mg | CGTCA-motif | CGTCA | 180 | 190 | MeJA response regulatory element |
| SiRAF13 | SETIT_034839mg | CGTCA-motif | CGTCA | 509 | 519 | MeJA response regulatory element |
| SiRAF13 | SETIT_034839mg | CGTCA-motif | CGTCA | 541 | 551 | MeJA response regulatory element |
| SiRAF13 | SETIT_034839mg | CGTCA-motif | CGTCA | 695 | 705 | MeJA response regulatory element |
| SiRAF13 | SETIT_034839mg | TGACG-motif | TGACG | 89 | 99 | MeJA response regulatory element |
| SiRAF13 | SETIT_034839mg | TGACG-motif | TGACG | 117 | 127 | MeJA response regulatory element |
| SiRAF13 | SETIT_034839mg | TGACG-motif | TGACG | 180 | 190 | MeJA response regulatory element |
| SiRAF13 | SETIT_034839mg | TGACG-motif | TGACG | 509 | 519 | MeJA response regulatory element |
| SiRAF13 | SETIT_034839mg | TGACG-motif | TGACG | 541 | 551 | MeJA response regulatory element |
| SiRAF13 | SETIT_034839mg | TGACG-motif | TGACG | 695 | 705 | MeJA response regulatory element |
| SiRAF13 | SETIT_034839mg | CAT-box | GCCACT | 68 | 80 | meristem expression regulatory element |
| SiRAF13 | SETIT_034839mg | CAT-box | GCCACT | 803 | 815 | meristem expression regulatory element |
| SiRAF13 | SETIT_034839mg | CAT-box | GCCACT | 845 | 857 | meristem expression regulatory element |
| SiRAF13 | SETIT_034839mg | MBS | CAACTG | 5 | 17 | MYB binding site involved in drought-inducibility |
| SiRAF13 | SETIT_034839mg | MBS | CAACTG | 1672 | 1684 | MYB binding site involved in drought-inducibility |
| SiRAF13 | SETIT_034839mg | CCAAT-box | CAACGG | 100 | 112 | MYBHv1 binding site |
| SiRAF13 | SETIT_034839mg | CCAAT-box | CAACGG | 125 | 137 | MYBHv1 binding site |
| SiRAF13 | SETIT_034839mg | CCAAT-box | CAACGG | 294 | 306 | MYBHv1 binding site |
| SiRAF13 | SETIT_034839mg | A-box | CCGTCC | 109 | 121 | promoter and enhancer cis-acting regulatory elements |
| SiRAF13 | SETIT_034839mg | TCA-element | CCATCTTTTT | 760 | 778 | salicylic acid response element |
| SiRAF14 | SETIT_016301mg | ABRE | ACGTG | 984 | 994 | abscisic acid response element |
| SiRAF14 | SETIT_016301mg | ABRE | GACACGTGGC | 1039 | 1057 | abscisic acid response element |
| SiRAF14 | SETIT_016301mg | ABRE | CACGTG | 1044 | 1056 | abscisic acid response element |
| SiRAF14 | SETIT_016301mg | ABRE | ACGTG | 1046 | 1056 | abscisic acid response element |
| SiRAF14 | SETIT_016301mg | ABRE | ACGTG | 1324 | 1334 | abscisic acid response element |
| SiRAF14 | SETIT_016301mg | ABRE | ACGTG | 1385 | 1395 | abscisic acid response element |
| SiRAF14 | SETIT_016301mg | ABRE | ACGTG | 1510 | 1520 | abscisic acid response element |
| SiRAF14 | SETIT_016301mg | ABRE | ACGTG | 1825 | 1835 | abscisic acid response element |
| SiRAF14 | SETIT_016301mg | ARE | AAACCA | 599 | 611 | anaerobic inducing element |
| SiRAF14 | SETIT_016301mg | ARE | AAACCA | 1394 | 1406 | anaerobic inducing element |
| SiRAF14 | SETIT_016301mg | ARE | AAACCA | 1567 | 1579 | anaerobic inducing element |
| SiRAF14 | SETIT_016301mg | AT-rich element | ATAGAAATCAA | 322 | 342 | ATBP-1 binding site |
| SiRAF14 | SETIT_016301mg | CAAT-box | CAAAT | 50 | 60 | common cis-acting element |
| SiRAF14 | SETIT_016301mg | CAAT-box | CAAAT | 139 | 149 | common cis-acting element |
| SiRAF14 | SETIT_016301mg | CAAT-box | CCAAT | 198 | 208 | common cis-acting element |
| SiRAF14 | SETIT_016301mg | CAAT-box | CAAAT | 230 | 240 | common cis-acting element |
| SiRAF14 | SETIT_016301mg | CAAT-box | CAAAT | 319 | 329 | common cis-acting element |
| SiRAF14 | SETIT_016301mg | CAAT-box | CAAAT | 325 | 335 | common cis-acting element |
| SiRAF14 | SETIT_016301mg | CAAT-box | CAAAT | 453 | 463 | common cis-acting element |
| SiRAF14 | SETIT_016301mg | CAAT-box | CAAAT | 615 | 625 | common cis-acting element |
| SiRAF14 | SETIT_016301mg | CAAT-box | CAAAT | 618 | 628 | common cis-acting element |
| SiRAF14 | SETIT_016301mg | CAAT-box | CAAAT | 633 | 643 | common cis-acting element |
| SiRAF14 | SETIT_016301mg | CAAT-box | CAAAT | 717 | 727 | common cis-acting element |
| SiRAF14 | SETIT_016301mg | CAAT-box | CAAAT | 766 | 776 | common cis-acting element |
| SiRAF14 | SETIT_016301mg | CAAT-box | CCAAT | 926 | 936 | common cis-acting element |
| SiRAF14 | SETIT_016301mg | CAAT-box | CAAAT | 1300 | 1310 | common cis-acting element |
| SiRAF14 | SETIT_016301mg | CAAT-box | CCAAT | 1305 | 1315 | common cis-acting element |
| SiRAF14 | SETIT_016301mg | CAAT-box | CAAAT | 1408 | 1418 | common cis-acting element |
| SiRAF14 | SETIT_016301mg | CAAT-box | CAAAT | 1432 | 1442 | common cis-acting element |
| SiRAF14 | SETIT_016301mg | CAAT-box | CCAAT | 1692 | 1702 | common cis-acting element |
| SiRAF14 | SETIT_016301mg | CAAT-box | CCAAT | 1799 | 1809 | common cis-acting element |
| SiRAF14 | SETIT_016301mg | CAAT-box | CCAAT | 1913 | 1923 | common cis-acting element |
| SiRAF14 | SETIT_016301mg | TATA-box | TATTTAAA | -5 | 11 | core promoter element |
| SiRAF14 | SETIT_016301mg | TATA-box | ATATAA | 107 | 119 | core promoter element |
| SiRAF14 | SETIT_016301mg | TATA-box | TATA | 110 | 118 | core promoter element |
| SiRAF14 | SETIT_016301mg | TATA-box | ATTATA | 203 | 215 | core promoter element |
| SiRAF14 | SETIT_016301mg | TATA-box | TATAA | 205 | 215 | core promoter element |
| SiRAF14 | SETIT_016301mg | TATA-box | TATA | 207 | 215 | core promoter element |
| SiRAF14 | SETIT_016301mg | TATA-box | TATAAAT | 508 | 522 | core promoter element |
| SiRAF14 | SETIT_016301mg | TATA-box | TATAAA | 510 | 522 | core promoter element |
| SiRAF14 | SETIT_016301mg | TATA-box | TATAA | 512 | 522 | core promoter element |
| SiRAF14 | SETIT_016301mg | TATA-box | TATA | 514 | 522 | core promoter element |
| SiRAF14 | SETIT_016301mg | TATA-box | TATAAAA | 850 | 864 | core promoter element |
| SiRAF14 | SETIT_016301mg | TATA-box | TATAAA | 852 | 864 | core promoter element |
| SiRAF14 | SETIT_016301mg | TATA-box | TATAA | 854 | 864 | core promoter element |
| SiRAF14 | SETIT_016301mg | TATA-box | TATA | 856 | 864 | core promoter element |
| SiRAF14 | SETIT_016301mg | TATA-box | TATA | 954 | 962 | core promoter element |
| SiRAF14 | SETIT_016301mg | TATA-box | TATA | 1014 | 1022 | core promoter element |
| SiRAF14 | SETIT_016301mg | TATA-box | ATATAA | 1135 | 1147 | core promoter element |
| SiRAF14 | SETIT_016301mg | TATA-box | TATA | 1138 | 1146 | core promoter element |
| SiRAF14 | SETIT_016301mg | GCN4_motif | TGAGTCA | 816 | 830 | endosperm expression regulatory element |
| SiRAF14 | SETIT_016301mg | P-box | CCTTTTG | 210 | 224 | gibberellin-responsive element |
| SiRAF14 | SETIT_016301mg | GC-motif | CCCCCG | 1490 | 1502 | hypoxia-specifically induced elements |
| SiRAF14 | SETIT_016301mg | GC-motif | CCCCCG | 1545 | 1557 | hypoxia-specifically induced elements |
| SiRAF14 | SETIT_016301mg | GC-motif | CCCCCG | 1553 | 1565 | hypoxia-specifically induced elements |
| SiRAF14 | SETIT_016301mg | GC-motif | CCCCCG | 1957 | 1969 | hypoxia-specifically induced elements |
| SiRAF14 | SETIT_016301mg | G-Box | CACGTG | 1044 | 1056 | light responsive element |
| SiRAF14 | SETIT_016301mg | G-box | TACGTG | 983 | 995 | light responsive element |
| SiRAF14 | SETIT_016301mg | G-box | ACACGTG(G/t)CACC | 1034 | 1056 | light responsive element |
| SiRAF14 | SETIT_016301mg | G-box | tgACACGTGGCA | 1036 | 1058 | light responsive element |
| SiRAF14 | SETIT_016301mg | G-box | CACGTG | 1044 | 1056 | light responsive element |
| SiRAF14 | SETIT_016301mg | G-box | CACGTC | 1323 | 1335 | light responsive element |
| SiRAF14 | SETIT_016301mg | G-box | CACGTC | 1383 | 1395 | light responsive element |
| SiRAF14 | SETIT_016301mg | G-box | CACGTC | 1509 | 1521 | light responsive element |
| SiRAF14 | SETIT_016301mg | G-box | CACGAC | 1786 | 1798 | light responsive element |
| SiRAF14 | SETIT_016301mg | G-box | CACGTC | 1824 | 1836 | light responsive element |
| SiRAF14 | SETIT_016301mg | Sp1 | GGGCGG | 1526 | 1538 | light responsive element |
| SiRAF14 | SETIT_016301mg | Sp1 | GGGCGG | 1531 | 1543 | light responsive element |
| SiRAF14 | SETIT_016301mg | Sp1 | GGGCGG | 1548 | 1560 | light responsive element |
| SiRAF14 | SETIT_016301mg | Sp1 | GGGCGG | 1556 | 1568 | light responsive element |
| SiRAF14 | SETIT_016301mg | Box II | TGGTAATAA | 1584 | 1602 | light responsive element |
| SiRAF14 | SETIT_016301mg | GA-motif | ATAGATAA | 1080 | 1096 | light responsive element |
| SiRAF14 | SETIT_016301mg | Gap-box | CAAATGAA(A/G)A | 225.5 | 244.5 | light responsive element |
| SiRAF14 | SETIT_016301mg | LTR | CCGAAA | 57 | 69 | low temperature response element |
| SiRAF14 | SETIT_016301mg | CGTCA-motif | CGTCA | 1512 | 1522 | MeJA response regulatory element |
| SiRAF14 | SETIT_016301mg | CGTCA-motif | CGTCA | 1563 | 1573 | MeJA response regulatory element |
| SiRAF14 | SETIT_016301mg | CGTCA-motif | CGTCA | 1827 | 1837 | MeJA response regulatory element |
| SiRAF14 | SETIT_016301mg | TGACG-motif | TGACG | 1512 | 1522 | MeJA response regulatory element |
| SiRAF14 | SETIT_016301mg | TGACG-motif | TGACG | 1563 | 1573 | MeJA response regulatory element |
| SiRAF14 | SETIT_016301mg | TGACG-motif | TGACG | 1827 | 1837 | MeJA response regulatory element |
| SiRAF14 | SETIT_016301mg | CCAAT-box | CAACGG | 1285 | 1297 | MYBHv1 binding site |
| SiRAF14 | SETIT_016301mg | A-box | CCGTCC | 1536 | 1548 | promoter and enhancer cis-acting regulatory elements |
| SiRAF15 | SETIT_028893mg | ARE | AAACCA | 795 | 807 | anaerobic inducing element |
| SiRAF15 | SETIT_028893mg | ARE | AAACCA | 823 | 835 | anaerobic inducing element |
| SiRAF15 | SETIT_028893mg | ARE | AAACCA | 1452 | 1464 | anaerobic inducing element |
| SiRAF15 | SETIT_028893mg | ARE | AAACCA | 1890 | 1902 | anaerobic inducing element |
| SiRAF15 | SETIT_028893mg | CAAT-box | CAAAT | 89 | 99 | common cis-acting element |
| SiRAF15 | SETIT_028893mg | CAAT-box | CCAAT | 101 | 111 | common cis-acting element |
| SiRAF15 | SETIT_028893mg | CAAT-box | CAAAT | 228 | 238 | common cis-acting element |
| SiRAF15 | SETIT_028893mg | CAAT-box | CAAAT | 522 | 532 | common cis-acting element |
| SiRAF15 | SETIT_028893mg | CAAT-box | CAAAT | 561 | 571 | common cis-acting element |
| SiRAF15 | SETIT_028893mg | CAAT-box | CCAAT | 777 | 787 | common cis-acting element |
| SiRAF15 | SETIT_028893mg | CAAT-box | CCAAT | 799 | 809 | common cis-acting element |
| SiRAF15 | SETIT_028893mg | CAAT-box | CCAAT | 848 | 858 | common cis-acting element |
| SiRAF15 | SETIT_028893mg | CAAT-box | CAAAT | 865 | 875 | common cis-acting element |
| SiRAF15 | SETIT_028893mg | CAAT-box | CAAAT | 1199 | 1209 | common cis-acting element |
| SiRAF15 | SETIT_028893mg | CAAT-box | CCAAT | 1214 | 1224 | common cis-acting element |
| SiRAF15 | SETIT_028893mg | CAAT-box | CCAAT | 1615 | 1625 | common cis-acting element |
| SiRAF15 | SETIT_028893mg | CAAT-box | TGCCAAC | 1662 | 1676 | common cis-acting element |
| SiRAF15 | SETIT_028893mg | CAAT-box | CAAAT | 1679 | 1689 | common cis-acting element |
| SiRAF15 | SETIT_028893mg | CAAT-box | CCAAT | 1816 | 1826 | common cis-acting element |
| SiRAF15 | SETIT_028893mg | CAAT-box | CAAAT | 1949 | 1959 | common cis-acting element |
| SiRAF15 | SETIT_028893mg | TATA-box | TATA | 239 | 247 | core promoter element |
| SiRAF15 | SETIT_028893mg | TATA-box | TATACA | 254 | 266 | core promoter element |
| SiRAF15 | SETIT_028893mg | TATA-box | TATA | 258 | 266 | core promoter element |
| SiRAF15 | SETIT_028893mg | TATA-box | TATA | 283 | 291 | core promoter element |
| SiRAF15 | SETIT_028893mg | TATA-box | TATAAA | 395 | 407 | core promoter element |
| SiRAF15 | SETIT_028893mg | TATA-box | TATAA | 397 | 407 | core promoter element |
| SiRAF15 | SETIT_028893mg | TATA-box | TATA | 399 | 407 | core promoter element |
| SiRAF15 | SETIT_028893mg | TATA-box | TACAAAA | 497 | 511 | core promoter element |
| SiRAF15 | SETIT_028893mg | TATA-box | ATATAT | 545 | 557 | core promoter element |
| SiRAF15 | SETIT_028893mg | TATA-box | TATA | 548 | 556 | core promoter element |
| SiRAF15 | SETIT_028893mg | TATA-box | TATTTAAA | 614 | 630 | core promoter element |
| SiRAF15 | SETIT_028893mg | TATA-box | TATATA | 755 | 767 | core promoter element |
| SiRAF15 | SETIT_028893mg | TATA-box | TATA | 759 | 767 | core promoter element |
| SiRAF15 | SETIT_028893mg | TATA-box | TATATA | 903 | 915 | core promoter element |
| SiRAF15 | SETIT_028893mg | TATA-box | ATATAT | 904 | 916 | core promoter element |
| SiRAF15 | SETIT_028893mg | TATA-box | TATATA | 905 | 917 | core promoter element |
| SiRAF15 | SETIT_028893mg | TATA-box | ATATAA | 906 | 918 | core promoter element |
| SiRAF15 | SETIT_028893mg | TATA-box | TATA | 909 | 917 | core promoter element |
| SiRAF15 | SETIT_028893mg | TATA-box | TACAAAA | 1029 | 1043 | core promoter element |
| SiRAF15 | SETIT_028893mg | TATA-box | TATA | 1145 | 1153 | core promoter element |
| SiRAF15 | SETIT_028893mg | TATA-box | ATATAA | 1193 | 1205 | core promoter element |
| SiRAF15 | SETIT_028893mg | TATA-box | TATA | 1196 | 1204 | core promoter element |
| SiRAF15 | SETIT_028893mg | TATA-box | TATAA | 1210 | 1220 | core promoter element |
| SiRAF15 | SETIT_028893mg | TATA-box | TATA | 1212 | 1220 | core promoter element |
| SiRAF15 | SETIT_028893mg | TATA-box | TACAAAA | 1291 | 1305 | core promoter element |
| SiRAF15 | SETIT_028893mg | TATA-box | TATA | 1588 | 1596 | core promoter element |
| SiRAF15 | SETIT_028893mg | TATA-box | ATATAT | 1971 | 1983 | core promoter element |
| SiRAF15 | SETIT_028893mg | TATA-box | TATATA | 1972 | 1984 | core promoter element |
| SiRAF15 | SETIT_028893mg | TATA-box | ATATAA | 1973 | 1985 | core promoter element |
| SiRAF15 | SETIT_028893mg | TATA-box | TATA | 1976 | 1984 | core promoter element |
| SiRAF15 | SETIT_028893mg | TC-rich repeats | ATTCTCTAAC | 1261 | 1279 | defense and stress response elements |
| SiRAF15 | SETIT_028893mg | GT1-motif | GGTTAA | 1404 | 1416 | light responsive element |
| SiRAF15 | SETIT_028893mg | Box 4 | ATTAAT | 801 | 813 | light responsive element |
| SiRAF15 | SETIT_028893mg | I-box | AAGATAAGGCT | 1332 | 1352 | light responsive element |
| SiRAF15 | SETIT_028893mg | I-box | AGATAAGG | 1336 | 1352 | light responsive element |
| SiRAF15 | SETIT_028893mg | TCT-motif | TCTTAC | 150 | 162 | light responsive element |
| SiRAF15 | SETIT_028893mg | TCT-motif | TCTTAC | 1343 | 1355 | light responsive element |
| SiRAF15 | SETIT_028893mg | AE-box | AGAAACAA | 1605 | 1621 | light responsive element |
| SiRAF15 | SETIT_028893mg | CGTCA-motif | CGTCA | 1632 | 1642 | MeJA response regulatory element |
| SiRAF15 | SETIT_028893mg | TGACG-motif | TGACG | 1632 | 1642 | MeJA response regulatory element |
| SiRAF15 | SETIT_028893mg | CAT-box | GCCACT | 158 | 170 | meristem expression regulatory element |
| SiRAF15 | SETIT_028893mg | MBS | CAACTG | 216 | 228 | MYB binding site involved in drought-inducibility |
| SiRAF15 | SETIT_028893mg | O2-site | GATGACATGG | 813 | 831 | Zein metabolism regulatory elements |
| SiRAF15 | SETIT_028893mg | O2-site | GATGACATGG | 1092 | 1110 | Zein metabolism regulatory elements |
| SiRAF16 | SETIT_0306602mg | ARE | AAACCA | 788 | 800 | anaerobic inducing element |
| SiRAF16 | SETIT_0306602mg | ARE | AAACCA | 958 | 970 | anaerobic inducing element |
| SiRAF16 | SETIT_0306602mg | ARE | AAACCA | 1466 | 1478 | anaerobic inducing element |
| SiRAF16 | SETIT_0306602mg | TGA-element | AACGAC | 138 | 150 | auxin-responsive element |
| SiRAF16 | SETIT_0306602mg | TGA-element | AACGAC | 990 | 1002 | auxin-responsive element |
| SiRAF16 | SETIT_0306602mg | CAAT-box | CAAAT | 19 | 29 | common cis-acting element |
| SiRAF16 | SETIT_0306602mg | CAAT-box | CAAAT | 224 | 234 | common cis-acting element |
| SiRAF16 | SETIT_0306602mg | CAAT-box | CAAAT | 248 | 258 | common cis-acting element |
| SiRAF16 | SETIT_0306602mg | CAAT-box | CCAAT | 310 | 320 | common cis-acting element |
| SiRAF16 | SETIT_0306602mg | CAAT-box | CCAAT | 746 | 756 | common cis-acting element |
| SiRAF16 | SETIT_0306602mg | CAAT-box | CAAAT | 825 | 835 | common cis-acting element |
| SiRAF16 | SETIT_0306602mg | CAAT-box | CCAAT | 929 | 939 | common cis-acting element |
| SiRAF16 | SETIT_0306602mg | CAAT-box | CCAAT | 1076 | 1086 | common cis-acting element |
| SiRAF16 | SETIT_0306602mg | CAAT-box | CAAAT | 1129 | 1139 | common cis-acting element |
| SiRAF16 | SETIT_0306602mg | CAAT-box | CAAAT | 1261 | 1271 | common cis-acting element |
| SiRAF16 | SETIT_0306602mg | TATA-box | TATA | 8 | 16 | core promoter element |
| SiRAF16 | SETIT_0306602mg | TATA-box | ATATAA | 147 | 159 | core promoter element |
| SiRAF16 | SETIT_0306602mg | TATA-box | TATA | 150 | 158 | core promoter element |
| SiRAF16 | SETIT_0306602mg | TATA-box | TATA | 196 | 204 | core promoter element |
| SiRAF16 | SETIT_0306602mg | TATA-box | TATAAAA | 541 | 555 | core promoter element |
| SiRAF16 | SETIT_0306602mg | TATA-box | TATAAA | 543 | 555 | core promoter element |
| SiRAF16 | SETIT_0306602mg | TATA-box | TATAA | 545 | 555 | core promoter element |
| SiRAF16 | SETIT_0306602mg | TATA-box | TATA | 547 | 555 | core promoter element |
| SiRAF16 | SETIT_0306602mg | TATA-box | TATAA | 684 | 694 | core promoter element |
| SiRAF16 | SETIT_0306602mg | TATA-box | TATA | 686 | 694 | core promoter element |
| SiRAF16 | SETIT_0306602mg | TATA-box | TACAAAA | 1023 | 1037 | core promoter element |
| SiRAF16 | SETIT_0306602mg | TATA-box | TATA | 1342 | 1350 | core promoter element |
| SiRAF16 | SETIT_0306602mg | TATA-box | ATTATA | 1541 | 1553 | core promoter element |
| SiRAF16 | SETIT_0306602mg | TATA-box | TATAA | 1543 | 1553 | core promoter element |
| SiRAF16 | SETIT_0306602mg | TATA-box | TATA | 1545 | 1553 | core promoter element |
| SiRAF16 | SETIT_0306602mg | TATA-box | TATAAAA | 1763 | 1777 | core promoter element |
| SiRAF16 | SETIT_0306602mg | TATA-box | TATAAA | 1765 | 1777 | core promoter element |
| SiRAF16 | SETIT_0306602mg | TATA-box | TATAA | 1767 | 1777 | core promoter element |
| SiRAF16 | SETIT_0306602mg | TATA-box | TATA | 1769 | 1777 | core promoter element |
| SiRAF16 | SETIT_0306602mg | TATA-box | TATAA | 1778 | 1788 | core promoter element |
| SiRAF16 | SETIT_0306602mg | TATA-box | TATA | 1780 | 1788 | core promoter element |
| SiRAF16 | SETIT_0306602mg | TATA-box | TATA | 1869 | 1877 | core promoter element |
| SiRAF16 | SETIT_0306602mg | TATA-box | TATAAAT | 1934 | 1948 | core promoter element |
| SiRAF16 | SETIT_0306602mg | TATA-box | TATAAA | 1936 | 1948 | core promoter element |
| SiRAF16 | SETIT_0306602mg | TATA-box | TATAA | 1938 | 1948 | core promoter element |
| SiRAF16 | SETIT_0306602mg | TATA-box | TATA | 1940 | 1948 | core promoter element |
| SiRAF16 | SETIT_0306602mg | TC-rich repeats | ATTCTCTAAC | 1526 | 1544 | defense and stress response elements |
| SiRAF16 | SETIT_0306602mg | TC-rich repeats | GTTTTCTTAC | 1751 | 1769 | defense and stress response elements |
| SiRAF16 | SETIT_0306602mg | TC-rich repeats | GTTTTCTTAC | 1830 | 1848 | defense and stress response elements |
| SiRAF16 | SETIT_0306602mg | GARE-motif | TCTGTTG | 645 | 659 | gibberellin-responsive element |
| SiRAF16 | SETIT_0306602mg | P-box | CCTTTTG | 1912 | 1926 | gibberellin-responsive element |
| SiRAF16 | SETIT_0306602mg | GT1-motif | GGTTAA | 121 | 133 | light responsive element |
| SiRAF16 | SETIT_0306602mg | GT1-motif | GGTTAA | 322 | 334 | light responsive element |
| SiRAF16 | SETIT_0306602mg | GT1-motif | GGTTAAT | 464 | 478 | light responsive element |
| SiRAF16 | SETIT_0306602mg | GT1-motif | GGTTAA | 466 | 478 | light responsive element |
| SiRAF16 | SETIT_0306602mg | AAAC-motif | CAATCAAAACCT | 1236 | 1258 | light responsive element |
| SiRAF16 | SETIT_0306602mg | ATC-motif | AGTAATCT | 1887 | 1903 | light responsive element |
| SiRAF16 | SETIT_0306602mg | AE-box | AGAAACTT | 1491 | 1507 | light responsive element |
| SiRAF16 | SETIT_0306602mg | CGTCA-motif | CGTCA | 1557 | 1567 | MeJA response regulatory element |
| SiRAF16 | SETIT_0306602mg | TGACG-motif | TGACG | 1557 | 1567 | MeJA response regulatory element |
| SiRAF16 | SETIT_0306602mg | CAT-box | GCCACT | 179 | 191 | meristem expression regulatory element |
| SiRAF16 | SETIT_0306602mg | MBSI | aaaAaaC(G/C)GTTA | 462.5 | 483.5 | MYB binding site involved in flavonoid biosynthetic genes regulation |
| SiRAF16 | SETIT_0306602mg | TCA-element | CCATCTTTTT | 712 | 732 | salicylic acid response element |
| SiRAF16 | SETIT_0306602mg | TCA-element | CCATCTTTTT | 1415 | 1433 | salicylic acid response element |
| SiRAF16 | SETIT_0306602mg | O2-site | GATGACATGG | 1615 | 1633 | Zein metabolism regulatory elements |
| SiRAF16 | SETIT_0306602mg | O2-site | GATGACATGG | 1795 | 1813 | Zein metabolism regulatory elements |
| SiRAF17 | SETIT_016766mg | ABRE | CACGTG | 1880 | 1892 | abscisic acid response element |
| SiRAF17 | SETIT_016766mg | ABRE | ACGTG | 1882 | 1892 | abscisic acid response element |
| SiRAF17 | SETIT_016766mg | ARE | AAACCA | 1644 | 1656 | anaerobic inducing element |
| SiRAF17 | SETIT_016766mg | ARE | AAACCA | 1737 | 1749 | anaerobic inducing element |
| SiRAF17 | SETIT_016766mg | CAAT-box | CAAAT | 133 | 143 | common cis-acting element |
| SiRAF17 | SETIT_016766mg | CAAT-box | CAAAT | 203 | 213 | common cis-acting element |
| SiRAF17 | SETIT_016766mg | CAAT-box | CAAAT | 231 | 241 | common cis-acting element |
| SiRAF17 | SETIT_016766mg | CAAT-box | CAAAT | 373 | 383 | common cis-acting element |
| SiRAF17 | SETIT_016766mg | CAAT-box | CAAAT | 666 | 676 | common cis-acting element |
| SiRAF17 | SETIT_016766mg | CAAT-box | CAAAT | 671 | 681 | common cis-acting element |
| SiRAF17 | SETIT_016766mg | CAAT-box | CAAAT | 890 | 900 | common cis-acting element |
| SiRAF17 | SETIT_016766mg | CAAT-box | CAAAT | 933 | 943 | common cis-acting element |
| SiRAF17 | SETIT_016766mg | CAAT-box | CAAAT | 1158 | 1168 | common cis-acting element |
| SiRAF17 | SETIT_016766mg | CAAT-box | CAAAT | 1535 | 1545 | common cis-acting element |
| SiRAF17 | SETIT_016766mg | CAAT-box | CAAAT | 1585 | 1595 | common cis-acting element |
| SiRAF17 | SETIT_016766mg | CAAT-box | CAAAT | 1591 | 1601 | common cis-acting element |
| SiRAF17 | SETIT_016766mg | CAAT-box | CAAAT | 1661 | 1671 | common cis-acting element |
| SiRAF17 | SETIT_016766mg | CAAT-box | CAAAT | 1666 | 1676 | common cis-acting element |
| SiRAF17 | SETIT_016766mg | CAAT-box | CAAAT | 1763 | 1773 | common cis-acting element |
| SiRAF17 | SETIT_016766mg | TATA-box | ATTATA | 101 | 113 | core promoter element |
| SiRAF17 | SETIT_016766mg | TATA-box | TATAA | 103 | 113 | core promoter element |
| SiRAF17 | SETIT_016766mg | TATA-box | TATA | 105 | 113 | core promoter element |
| SiRAF17 | SETIT_016766mg | TATA-box | TACAAAA | 266 | 280 | core promoter element |
| SiRAF17 | SETIT_016766mg | TATA-box | ATATAT | 505 | 517 | core promoter element |
| SiRAF17 | SETIT_016766mg | TATA-box | TATA | 508 | 516 | core promoter element |
| SiRAF17 | SETIT_016766mg | TATA-box | TACATAAA | 580 | 596 | core promoter element |
| SiRAF17 | SETIT_016766mg | TATA-box | TATAA | 798 | 808 | core promoter element |
| SiRAF17 | SETIT_016766mg | TATA-box | TATA | 800 | 808 | core promoter element |
| SiRAF17 | SETIT_016766mg | TATA-box | TATAA | 850 | 860 | core promoter element |
| SiRAF17 | SETIT_016766mg | TATA-box | TATA | 852 | 860 | core promoter element |
| SiRAF17 | SETIT_016766mg | TATA-box | TATA | 1048 | 1056 | core promoter element |
| SiRAF17 | SETIT_016766mg | TATA-box | TATA | 1097 | 1105 | core promoter element |
| SiRAF17 | SETIT_016766mg | TATA-box | ATTATA | 1108 | 1120 | core promoter element |
| SiRAF17 | SETIT_016766mg | TATA-box | TATATAA | 1108 | 1122 | core promoter element |
| SiRAF17 | SETIT_016766mg | TATA-box | TATATA | 1110 | 1122 | core promoter element |
| SiRAF17 | SETIT_016766mg | TATA-box | ATATAT | 1111 | 1123 | core promoter element |
| SiRAF17 | SETIT_016766mg | TATA-box | TATA | 1114 | 1122 | core promoter element |
| SiRAF17 | SETIT_016766mg | TATA-box | ATTATA | 1152 | 1164 | core promoter element |
| SiRAF17 | SETIT_016766mg | TATA-box | TATAA | 1154 | 1164 | core promoter element |
| SiRAF17 | SETIT_016766mg | TATA-box | TATA | 1156 | 1164 | core promoter element |
| SiRAF17 | SETIT_016766mg | TATA-box | TATA | 1657 | 1665 | core promoter element |
| SiRAF17 | SETIT_016766mg | TATA-box | TATA | 1851 | 1859 | core promoter element |
| SiRAF17 | SETIT_016766mg | RY-element | CATGCATG | 627 | 643 | elements involved in seed-specific regulation |
| SiRAF17 | SETIT_016766mg | RY-element | CATGCATG | 1899 | 1915 | elements involved in seed-specific regulation |
| SiRAF17 | SETIT_016766mg | TATC-box | TATCCCA | 697 | 711 | gibberellin response element |
| SiRAF17 | SETIT_016766mg | G-Box | CACGTG | 1880 | 1892 | light responsive element |
| SiRAF17 | SETIT_016766mg | G-box | CACGAC | 1074 | 1086 | light responsive element |
| SiRAF17 | SETIT_016766mg | G-box | CACGTG | 1880 | 1892 | light responsive element |
| SiRAF17 | SETIT_016766mg | GT1-motif | GGTTAA | 1347 | 1359 | light responsive element |
| SiRAF17 | SETIT_016766mg | Box 4 | ATTAAT | 7 | 19 | light responsive element |
| SiRAF17 | SETIT_016766mg | Box 4 | ATTAAT | 159 | 171 | light responsive element |
| SiRAF17 | SETIT_016766mg | Box 4 | ATTAAT | 274 | 286 | light responsive element |
| SiRAF17 | SETIT_016766mg | Box 4 | ATTAAT | 473 | 485 | light responsive element |
| SiRAF17 | SETIT_016766mg | Box 4 | ATTAAT | 704 | 716 | light responsive element |
| SiRAF17 | SETIT_016766mg | Box 4 | ATTAAT | 1124 | 1136 | light responsive element |
| SiRAF17 | SETIT_016766mg | Box 4 | ATTAAT | 1982 | 1994 | light responsive element |
| SiRAF17 | SETIT_016766mg | GATT-motif | CTCCTGATTAGC | 68 | 90 | light responsive element |
| SiRAF17 | SETIT_016766mg | I-box | TGATAATGT | 192 | 210 | light responsive element |
| SiRAF17 | SETIT_016766mg | I-box | AAGATAAGGCT | 722 | 742 | light responsive element |
| SiRAF17 | SETIT_016766mg | I-box | AGATAAGG | 1630 | 1646 | light responsive element |
| SiRAF17 | SETIT_016766mg | CGTCA-motif | CGTCA | 1234 | 1244 | MeJA response regulatory element |
| SiRAF17 | SETIT_016766mg | CGTCA-motif | CGTCA | 1968 | 1978 | MeJA response regulatory element |
| SiRAF17 | SETIT_016766mg | TGACG-motif | TGACG | 1234 | 1244 | MeJA response regulatory element |
| SiRAF17 | SETIT_016766mg | TGACG-motif | TGACG | 1968 | 1978 | MeJA response regulatory element |
| SiRAF17 | SETIT_016766mg | MBS | CAACTG | 1337 | 1349 | MYB binding site involved in drought-inducibility |
| SiRAF17 | SETIT_016766mg | MRE | AACCTAA | 216 | 230 | MYB binding site involved in light responsiveness |
| SiRAF17 | SETIT_016766mg | A-box | CCGTCC | 1195 | 1207 | promoter and enhancer cis-acting regulatory elements |
| SiRAF17 | SETIT_016766mg | Box III | atCATTTTCACt | 1505 | 1527 | protein binding site |
| SiRAF17 | SETIT_016766mg | TCA-element | CCATCTTTTT | 813 | 831 | salicylic acid response element |
| SiRAF18 | SETIT_017053mg | ABRE | ACGTG | 1048 | 1058 | abscisic acid response element |
| SiRAF18 | SETIT_017053mg | ABRE | GCAACGTGTC | 1545 | 1563 | abscisic acid response element |
| SiRAF18 | SETIT_017053mg | ABRE | CACGTG | 1550 | 1562 | abscisic acid response element |
| SiRAF18 | SETIT_017053mg | ABRE | ACGTG | 1552 | 1562 | abscisic acid response element |
| SiRAF18 | SETIT_017053mg | CAAT-box | CCAAT | 33 | 43 | common cis-acting element |
| SiRAF18 | SETIT_017053mg | CAAT-box | CAAAT | 36 | 46 | common cis-acting element |
| SiRAF18 | SETIT_017053mg | CAAT-box | CAAAT | 283 | 293 | common cis-acting element |
| SiRAF18 | SETIT_017053mg | CAAT-box | CCAAT | 606 | 616 | common cis-acting element |
| SiRAF18 | SETIT_017053mg | CAAT-box | CAAAT | 673 | 683 | common cis-acting element |
| SiRAF18 | SETIT_017053mg | CAAT-box | CCAAT | 725 | 735 | common cis-acting element |
| SiRAF18 | SETIT_017053mg | CAAT-box | CAAAT | 846 | 856 | common cis-acting element |
| SiRAF18 | SETIT_017053mg | CAAT-box | CAAAT | 966 | 976 | common cis-acting element |
| SiRAF18 | SETIT_017053mg | CAAT-box | CCAAT | 982 | 992 | common cis-acting element |
| SiRAF18 | SETIT_017053mg | CAAT-box | CCAAT | 990 | 1000 | common cis-acting element |
| SiRAF18 | SETIT_017053mg | CAAT-box | TGCCAAC | 1336 | 1350 | common cis-acting element |
| SiRAF18 | SETIT_017053mg | CAAT-box | CAACCAACTCC | 1752 | 1772 | common cis-acting element |
| SiRAF18 | SETIT_017053mg | CAAT-box | CAAAT | 1786 | 1796 | common cis-acting element |
| SiRAF18 | SETIT_017053mg | CAAT-box | CCAAT | 1818 | 1828 | common cis-acting element |
| SiRAF18 | SETIT_017053mg | CAAT-box | CCAAT | 1821 | 1831 | common cis-acting element |
| SiRAF18 | SETIT_017053mg | CAAT-box | CCAAT | 1857 | 1867 | common cis-acting element |
| SiRAF18 | SETIT_017053mg | CAAT-box | CCAAT | 1871 | 1881 | common cis-acting element |
| SiRAF18 | SETIT_017053mg | TATA-box | TATA | 65 | 73 | core promoter element |
| SiRAF18 | SETIT_017053mg | TATA-box | ATTATA | 68 | 80 | core promoter element |
| SiRAF18 | SETIT_017053mg | TATA-box | TATAA | 70 | 80 | core promoter element |
| SiRAF18 | SETIT_017053mg | TATA-box | TATA | 72 | 80 | core promoter element |
| SiRAF18 | SETIT_017053mg | TATA-box | TATACA | 74 | 86 | core promoter element |
| SiRAF18 | SETIT_017053mg | TATA-box | TATATA | 76 | 88 | core promoter element |
| SiRAF18 | SETIT_017053mg | TATA-box | TATA | 80 | 88 | core promoter element |
| SiRAF18 | SETIT_017053mg | TATA-box | TATACA | 105 | 117 | core promoter element |
| SiRAF18 | SETIT_017053mg | TATA-box | TATA | 109 | 117 | core promoter element |
| SiRAF18 | SETIT_017053mg | TATA-box | TATACA | 116 | 128 | core promoter element |
| SiRAF18 | SETIT_017053mg | TATA-box | TATA | 120 | 128 | core promoter element |
| SiRAF18 | SETIT_017053mg | TATA-box | TACAAAA | 135 | 149 | core promoter element |
| SiRAF18 | SETIT_017053mg | TATA-box | ATTATA | 169 | 181 | core promoter element |
| SiRAF18 | SETIT_017053mg | TATA-box | TATAA | 171 | 181 | core promoter element |
| SiRAF18 | SETIT_017053mg | TATA-box | TATA | 173 | 181 | core promoter element |
| SiRAF18 | SETIT_017053mg | TATA-box | TATAA | 197 | 207 | core promoter element |
| SiRAF18 | SETIT_017053mg | TATA-box | TATA | 199 | 207 | core promoter element |
| SiRAF18 | SETIT_017053mg | TATA-box | TACAAAA | 290 | 304 | core promoter element |
| SiRAF18 | SETIT_017053mg | TATA-box | TATACA | 316 | 328 | core promoter element |
| SiRAF18 | SETIT_017053mg | TATA-box | TATA | 320 | 328 | core promoter element |
| SiRAF18 | SETIT_017053mg | TATA-box | TATAA | 325 | 335 | core promoter element |
| SiRAF18 | SETIT_017053mg | TATA-box | TATA | 327 | 335 | core promoter element |
| SiRAF18 | SETIT_017053mg | TATA-box | ATATAA | 679 | 691 | core promoter element |
| SiRAF18 | SETIT_017053mg | TATA-box | TATA | 682 | 690 | core promoter element |
| SiRAF18 | SETIT_017053mg | TATA-box | ATATAT | 750 | 762 | core promoter element |
| SiRAF18 | SETIT_017053mg | TATA-box | TATATA | 751 | 763 | core promoter element |
| SiRAF18 | SETIT_017053mg | TATA-box | ATATAT | 752 | 764 | core promoter element |
| SiRAF18 | SETIT_017053mg | TATA-box | TATATA | 753 | 765 | core promoter element |
| SiRAF18 | SETIT_017053mg | TATA-box | TATA | 757 | 765 | core promoter element |
| SiRAF18 | SETIT_017053mg | TATA-box | TACAAAA | 771 | 785 | core promoter element |
| SiRAF18 | SETIT_017053mg | TATA-box | ccTATAAAaa | 796 | 814 | core promoter element |
| SiRAF18 | SETIT_017053mg | TATA-box | TATATA | 809 | 821 | core promoter element |
| SiRAF18 | SETIT_017053mg | TATA-box | ATATAA | 810 | 822 | core promoter element |
| SiRAF18 | SETIT_017053mg | TATA-box | TATA | 813 | 821 | core promoter element |
| SiRAF18 | SETIT_017053mg | TATA-box | TATA | 865 | 873 | core promoter element |
| SiRAF18 | SETIT_017053mg | TATA-box | TATA | 1253 | 1261 | core promoter element |
| SiRAF18 | SETIT_017053mg | TATA-box | TATA | 1280 | 1288 | core promoter element |
| SiRAF18 | SETIT_017053mg | TATA-box | ATATAT | 1709 | 1721 | core promoter element |
| SiRAF18 | SETIT_017053mg | TATA-box | TATATA | 1710 | 1722 | core promoter element |
| SiRAF18 | SETIT_017053mg | TATA-box | ATATAT | 1711 | 1723 | core promoter element |
| SiRAF18 | SETIT_017053mg | TATA-box | TATA | 1714 | 1722 | core promoter element |
| SiRAF18 | SETIT_017053mg | GC-motif | CCCCCG | 504 | 516 | hypoxia-specifically induced elements |
| SiRAF18 | SETIT_017053mg | GC-motif | CCCCCG | 1595 | 1607 | hypoxia-specifically induced elements |
| SiRAF18 | SETIT_017053mg | GC-motif | CCCCCG | 1642 | 1654 | hypoxia-specifically induced elements |
| SiRAF18 | SETIT_017053mg | GC-motif | CCCCCG | 1830 | 1842 | hypoxia-specifically induced elements |
| SiRAF18 | SETIT_017053mg | ACE | CTAACGTATT | 1041 | 1059 | light responsive element |
| SiRAF18 | SETIT_017053mg | G-Box | CACGTT | 1046 | 1058 | light responsive element |
| SiRAF18 | SETIT_017053mg | G-Box | CACGTG | 1550 | 1562 | light responsive element |
| SiRAF18 | SETIT_017053mg | G-box | ACACGTG(G/t)CACC | 1540.5 | 1561.5 | light responsive element |
| SiRAF18 | SETIT_017053mg | G-box | tgACACGTGGCA | 1542 | 1564 | light responsive element |
| SiRAF18 | SETIT_017053mg | G-box | ACACGTGGC | 1546 | 1564 | light responsive element |
| SiRAF18 | SETIT_017053mg | G-box | CACGTG | 1550 | 1562 | light responsive element |
| SiRAF18 | SETIT_017053mg | Sp1 | GGGCGG | 501 | 513 | light responsive element |
| SiRAF18 | SETIT_017053mg | Sp1 | GGGCGG | 1846 | 1858 | light responsive element |
| SiRAF18 | SETIT_017053mg | Sp1 | GGGCGG | 1864 | 1876 | light responsive element |
| SiRAF18 | SETIT_017053mg | Box 4 | ATTAAT | 767 | 779 | light responsive element |
| SiRAF18 | SETIT_017053mg | ATCT-motif | AATCTAATCC | 1192 | 1210 | light responsive element |
| SiRAF18 | SETIT_017053mg | I-box | gGATAAGGTG | 381 | 399 | light responsive element |
| SiRAF18 | SETIT_017053mg | I-box | cCATATCCAAT | 714 | 734 | light responsive element |
| SiRAF18 | SETIT_017053mg | I-box | atGATAAGGTC | 1135 | 1155 | light responsive element |
| SiRAF18 | SETIT_017053mg | TCT-motif | TCTTAC | 921 | 933 | light responsive element |
| SiRAF18 | SETIT_017053mg | GATA-motif | AAGGATAAGG | 1 | 19 | light responsive element |
| SiRAF18 | SETIT_017053mg | GATA-motif | AAGGATAAGG | 1138 | 1156 | light responsive element |
| SiRAF18 | SETIT_017053mg | LTR | CCGAAA | 482 | 494 | low temperature response element |
| SiRAF18 | SETIT_017053mg | CGTCA-motif | CGTCA | 304 | 314 | MeJA response regulatory element |
| SiRAF18 | SETIT_017053mg | TGACG-motif | TGACG | 304 | 314 | MeJA response regulatory element |
| SiRAF18 | SETIT_017053mg | CAT-box | GCCACT | 462 | 474 | meristem expression regulatory element |
| SiRAF18 | SETIT_017053mg | CAT-box | GCCACT | 630 | 642 | meristem expression regulatory element |
| SiRAF18 | SETIT_017053mg | MBS | CAACTG | 1367 | 1379 | MYB binding site involved in drought-inducibility |
| SiRAF18 | SETIT_017053mg | CCAAT-box | CAACGG | 1724 | 1736 | MYBHv1 binding site |
| SiRAF18 | SETIT_017053mg | A-box | CCGTCC | 1458 | 1470 | promoter and enhancer cis-acting regulatory elements |
| SiRAF18 | SETIT_017053mg | TCA-element | TCAGAAGAGG | 571 | 589 | salicylic acid response element |
| SiRAF18 | SETIT_017053mg | TCA-element | CCATCTTTTT | 591 | 609 | salicylic acid response element |
| SiRAF19 | SETIT_003916mg | ABRE | GCCGCGTGGC | 176 | 194 | abscisic acid response element |
| SiRAF19 | SETIT_003916mg | ABRE | ACGTG | 242 | 252 | abscisic acid response element |
| SiRAF19 | SETIT_003916mg | ABRE | ACGTG | 308 | 318 | abscisic acid response element |
| SiRAF19 | SETIT_003916mg | ABRE | ACGTG | 740 | 750 | abscisic acid response element |
| SiRAF19 | SETIT_003916mg | ABRE | GACACGTACGT | 743 | 763 | abscisic acid response element |
| SiRAF19 | SETIT_003916mg | ABRE | ACGTG | 752 | 762 | abscisic acid response element |
| SiRAF19 | SETIT_003916mg | ARE | AAACCA | 1532 | 1544 | anaerobic inducing element |
| SiRAF19 | SETIT_003916mg | AuxRR-core | GGTCCAT | 609 | 623 | auxin response element |
| SiRAF19 | SETIT_003916mg | TGA-element | AACGAC | 1158 | 1170 | auxin-responsive element |
| SiRAF19 | SETIT_003916mg | TGA-element | AACGAC | 1472 | 1484 | auxin-responsive element |
| SiRAF19 | SETIT_003916mg | MSA-like | TCCAACGGT | 1677 | 1695 | cell cycle control element |
| SiRAF19 | SETIT_003916mg | circadian | CAAAGATATC | 1310 | 1328 | circadian rhythm control element |
| SiRAF19 | SETIT_003916mg | CAAT-box | CAAAT | 952 | 962 | common cis-acting element |
| SiRAF19 | SETIT_003916mg | CAAT-box | CCAAT | 973 | 983 | common cis-acting element |
| SiRAF19 | SETIT_003916mg | CAAT-box | CCAAT | 1025 | 1035 | common cis-acting element |
| SiRAF19 | SETIT_003916mg | CAAT-box | CAAAT | 1371 | 1381 | common cis-acting element |
| SiRAF19 | SETIT_003916mg | CAAT-box | CAAAT | 1440 | 1450 | common cis-acting element |
| SiRAF19 | SETIT_003916mg | TATA-box | TATA | 1171 | 1179 | core promoter element |
| SiRAF19 | SETIT_003916mg | TATA-box | ATTATA | 1442 | 1454 | core promoter element |
| SiRAF19 | SETIT_003916mg | TATA-box | TATAA | 1444 | 1454 | core promoter element |
| SiRAF19 | SETIT_003916mg | TATA-box | TATA | 1446 | 1454 | core promoter element |
| SiRAF19 | SETIT_003916mg | TATA-box | TATAAAA | 1549 | 1563 | core promoter element |
| SiRAF19 | SETIT_003916mg | TATA-box | TATAAA | 1551 | 1563 | core promoter element |
| SiRAF19 | SETIT_003916mg | TATA-box | TATAA | 1553 | 1563 | core promoter element |
| SiRAF19 | SETIT_003916mg | TATA-box | TATA | 1555 | 1563 | core promoter element |
| SiRAF19 | SETIT_003916mg | TATA-box | TATA | 1574 | 1582 | core promoter element |
| SiRAF19 | SETIT_003916mg | TATA-box | TATATA | 1742 | 1754 | core promoter element |
| SiRAF19 | SETIT_003916mg | TATA-box | TATA | 1746 | 1754 | core promoter element |
| SiRAF19 | SETIT_003916mg | P-box | CCTTTTG | 1000 | 1014 | gibberellin-responsive element |
| SiRAF19 | SETIT_003916mg | GARE-motif | TCTGTTG | 333 | 347 | gibberellin-responsive element |
| SiRAF19 | SETIT_003916mg | ACE | GCGACGTACC | 364 | 382 | light responsive element |
| SiRAF19 | SETIT_003916mg | G-box | CACGAC | 223 | 235 | light responsive element |
| SiRAF19 | SETIT_003916mg | G-box | CACGTC | 241 | 253 | light responsive element |
| SiRAF19 | SETIT_003916mg | G-box | CACGTC | 306 | 318 | light responsive element |
| SiRAF19 | SETIT_003916mg | G-box | CACGAC | 493 | 505 | light responsive element |
| SiRAF19 | SETIT_003916mg | G-box | CACGTC | 738 | 750 | light responsive element |
| SiRAF19 | SETIT_003916mg | G-box | TACGTG | 750 | 762 | light responsive element |
| SiRAF19 | SETIT_003916mg | Box 4 | ATTAAT | 1352 | 1364 | light responsive element |
| SiRAF19 | SETIT_003916mg | GATA-motif | GATAGGG | 981 | 995 | light responsive element |
| SiRAF19 | SETIT_003916mg | GATA-motif | AAGGATAAGG | 1101 | 1119 | light responsive element |
| SiRAF19 | SETIT_003916mg | GATA-motif | AAGATAAGATT | 1370 | 1390 | light responsive element |
| SiRAF19 | SETIT_003916mg | TCT-motif | TCTTAC | 1722 | 1734 | light responsive element |
| SiRAF19 | SETIT_003916mg | LTR | CCGAAA | 893 | 905 | low temperature response element |
| SiRAF19 | SETIT_003916mg | LTR | CCGAAA | 958 | 970 | low temperature response element |
| SiRAF19 | SETIT_003916mg | LTR | CCGAAA | 1643 | 1655 | low temperature response element |
| SiRAF19 | SETIT_003916mg | TGACG-motif | TGACG | 244 | 254 | MeJA response regulatory element |
| SiRAF19 | SETIT_003916mg | TGACG-motif | TGACG | 1732 | 1742 | MeJA response regulatory element |
| SiRAF19 | SETIT_003916mg | CGTCA-motif | CGTCA | 244 | 254 | MeJA response regulatory element |
| SiRAF19 | SETIT_003916mg | CGTCA-motif | CGTCA | 1732 | 1742 | MeJA response regulatory element |
| SiRAF19 | SETIT_003916mg | CCAAT-box | CAACGG | 700 | 712 | MYBHv1 binding site |
| SiRAF19 | SETIT_003916mg | CCAAT-box | CAACGG | 1682 | 1694 | MYBHv1 binding site |
| SiRAF19 | SETIT_003916mg | A-box | CCGTCC | 996 | 1008 | promoter and enhancer cis-acting regulatory elements |
| SiRAF19 | SETIT_003916mg | TCA-element | CCATCTTTTT | 1215 | 1233 | salicylic acid response element |
| SiRAF19 | SETIT_003916mg | TCA-element | CCATCTTTTT | 1499 | 1517 | salicylic acid response element |
| SiRAF19 | SETIT_003916mg | TCA-element | TCAGAAGAGG | 1830 | 1848 | salicylic acid response element |
| SiRAF19 | SETIT_003916mg | O2-site | GATGACATGG | 651 | 669 | Zein metabolism regulatory elements |
| SiRAF20 | SETIT_015231mg | ABRE | GCCGCGTGGC | 133 | 151 | abscisic acid response element |
| SiRAF20 | SETIT_015231mg | ABRE | GACACGTGGC | 839 | 857 | abscisic acid response element |
| SiRAF20 | SETIT_015231mg | ABRE | CACGTG | 844 | 856 | abscisic acid response element |
| SiRAF20 | SETIT_015231mg | ABRE | ACGTG | 846 | 856 | abscisic acid response element |
| SiRAF20 | SETIT_015231mg | TGA-element | AACGAC | 448 | 460 | auxin-responsive element |
| SiRAF20 | SETIT_015231mg | CAAT-box | CAAAT | 365 | 375 | common cis-acting element |
| SiRAF20 | SETIT_015231mg | CAAT-box | CAAAT | 460 | 470 | common cis-acting element |
| SiRAF20 | SETIT_015231mg | CAAT-box | CAAAT | 468 | 478 | common cis-acting element |
| SiRAF20 | SETIT_015231mg | CAAT-box | CAAAT | 487 | 497 | common cis-acting element |
| SiRAF20 | SETIT_015231mg | CAAT-box | TGCCAAC | 651 | 665 | common cis-acting element |
| SiRAF20 | SETIT_015231mg | CAAT-box | CAAAT | 762 | 772 | common cis-acting element |
| SiRAF20 | SETIT_015231mg | CAAT-box | CAAAT | 790 | 800 | common cis-acting element |
| SiRAF20 | SETIT_015231mg | CAAT-box | CCAAT | 1224 | 1234 | common cis-acting element |
| SiRAF20 | SETIT_015231mg | CAAT-box | CAAAT | 1324 | 1334 | common cis-acting element |
| SiRAF20 | SETIT_015231mg | CAAT-box | CAAAT | 1498 | 1508 | common cis-acting element |
| SiRAF20 | SETIT_015231mg | CAAT-box | CCAAT | 1909 | 1919 | common cis-acting element |
| SiRAF20 | SETIT_015231mg | TATA-box | ATTATA | 336 | 348 | core promoter element |
| SiRAF20 | SETIT_015231mg | TATA-box | TATAA | 338 | 348 | core promoter element |
| SiRAF20 | SETIT_015231mg | TATA-box | TATA | 340 | 348 | core promoter element |
| SiRAF20 | SETIT_015231mg | TATA-box | TATAAA | 398 | 410 | core promoter element |
| SiRAF20 | SETIT_015231mg | TATA-box | TATAA | 400 | 410 | core promoter element |
| SiRAF20 | SETIT_015231mg | TATA-box | TATA | 402 | 410 | core promoter element |
| SiRAF20 | SETIT_015231mg | TATA-box | ATTATA | 411 | 423 | core promoter element |
| SiRAF20 | SETIT_015231mg | TATA-box | TATAA | 413 | 423 | core promoter element |
| SiRAF20 | SETIT_015231mg | TATA-box | TATA | 415 | 423 | core promoter element |
| SiRAF20 | SETIT_015231mg | TATA-box | TAAAGATT | 1460 | 1476 | core promoter element |
| SiRAF20 | SETIT_015231mg | TATA-box | TATA | 1707 | 1715 | core promoter element |
| SiRAF20 | SETIT_015231mg | TATA-box | TATA | 1764 | 1772 | core promoter element |
| SiRAF20 | SETIT_015231mg | TATA-box | TACAAAA | 1796 | 1810 | core promoter element |
| SiRAF20 | SETIT_015231mg | GARE-motif | TCTGTTG | 514 | 528 | gibberellin-responsive element |
| SiRAF20 | SETIT_015231mg | GC-motif | CCCCCG | 837 | 849 | hypoxia-specifically induced elements |
| SiRAF20 | SETIT_015231mg | GC-motif | CCCCCG | 1162 | 1174 | hypoxia-specifically induced elements |
| SiRAF20 | SETIT_015231mg | GC-motif | CCCCCG | 1359 | 1371 | hypoxia-specifically induced elements |
| SiRAF20 | SETIT_015231mg | G-Box | CACGTG | 844 | 856 | light responsive element |
| SiRAF20 | SETIT_015231mg | G-box | CACGAC | 634 | 646 | light responsive element |
| SiRAF20 | SETIT_015231mg | G-box | CACGTG | 844 | 856 | light responsive element |
| SiRAF20 | SETIT_015231mg | GT1-motif | GGTTAA | 432 | 444 | light responsive element |
| SiRAF20 | SETIT_015231mg | GT1-motif | GGTTAA | 849 | 861 | light responsive element |
| SiRAF20 | SETIT_015231mg | GT1-motif | GGTTAA | 851 | 863 | light responsive element |
| SiRAF20 | SETIT_015231mg | Sp1 | GGGCGG | 1337 | 1349 | light responsive element |
| SiRAF20 | SETIT_015231mg | TCCC-motif | TCTCCCT | 265 | 279 | light responsive element |
| SiRAF20 | SETIT_015231mg | TCCC-motif | TCTCCCT | 1944 | 1958 | light responsive element |
| SiRAF20 | SETIT_015231mg | AE-box | AGAAACAA | 1753 | 1769 | light responsive element |
| SiRAF20 | SETIT_015231mg | LTR | CCGAAA | 1512 | 1524 | low temperature response element |
| SiRAF20 | SETIT_015231mg | LTR | CCGAAA | 1688 | 1700 | low temperature response element |
| SiRAF20 | SETIT_015231mg | CGTCA-motif | CGTCA | 127 | 137 | MeJA response regulatory element |
| SiRAF20 | SETIT_015231mg | CGTCA-motif | CGTCA | 732 | 742 | MeJA response regulatory element |
| SiRAF20 | SETIT_015231mg | TGACG-motif | TGACG | 127 | 137 | MeJA response regulatory element |
| SiRAF20 | SETIT_015231mg | TGACG-motif | TGACG | 732 | 742 | MeJA response regulatory element |
| SiRAF20 | SETIT_015231mg | MBS | CAACTG | 584 | 596 | MYB binding site involved in drought-inducibility |
| SiRAF20 | SETIT_015231mg | CCAAT-box | CAACGG | 1624 | 1636 | MYBHv1 binding site |
| SiRAF20 | SETIT_015231mg | A-box | CCGTCC | 683 | 695 | promoter and enhancer cis-acting regulatory elements |
| SiRAF20 | SETIT_015231mg | A-box | CCGTCC | 1205 | 1217 | promoter and enhancer cis-acting regulatory elements |
| SiRAF21 | SETIT_017293mg | ABRE | ACGTG | 630 | 640 | abscisic acid response element |
| SiRAF21 | SETIT_017293mg | ABRE | GCCGCGTGGC | 1419 | 1437 | abscisic acid response element |
| SiRAF21 | SETIT_017293mg | ARE | AAACCA | 1714 | 1726 | anaerobic inducing element |
| SiRAF21 | SETIT_017293mg | CAAT-box | CAAAT | 75 | 85 | common cis-acting element |
| SiRAF21 | SETIT_017293mg | CAAT-box | CAAAT | 229 | 239 | common cis-acting element |
| SiRAF21 | SETIT_017293mg | CAAT-box | CCAAT | 385 | 395 | common cis-acting element |
| SiRAF21 | SETIT_017293mg | CAAT-box | CAAAT | 457 | 467 | common cis-acting element |
| SiRAF21 | SETIT_017293mg | CAAT-box | CCAAT | 871 | 881 | common cis-acting element |
| SiRAF21 | SETIT_017293mg | CAAT-box | CCAAT | 888 | 898 | common cis-acting element |
| SiRAF21 | SETIT_017293mg | CAAT-box | CCAAT | 1283 | 1293 | common cis-acting element |
| SiRAF21 | SETIT_017293mg | CAAT-box | CAAAT | 1381 | 1391 | common cis-acting element |
| SiRAF21 | SETIT_017293mg | CAAT-box | CAAAT | 1412 | 1422 | common cis-acting element |
| SiRAF21 | SETIT_017293mg | CAAT-box | CCAAT | 1530 | 1540 | common cis-acting element |
| SiRAF21 | SETIT_017293mg | CAAT-box | CCAAT | 1547 | 1557 | common cis-acting element |
| SiRAF21 | SETIT_017293mg | CAAT-box | CAAAT | 1730 | 1740 | common cis-acting element |
| SiRAF21 | SETIT_017293mg | CAAT-box | CAAAT | 1886 | 1896 | common cis-acting element |
| SiRAF21 | SETIT_017293mg | CAAT-box | CAAAT | 1980 | 1990 | common cis-acting element |
| SiRAF21 | SETIT_017293mg | TATA-box | TATAA | 84 | 94 | core promoter element |
| SiRAF21 | SETIT_017293mg | TATA-box | TATA | 86 | 94 | core promoter element |
| SiRAF21 | SETIT_017293mg | TATA-box | ATATAA | 137 | 149 | core promoter element |
| SiRAF21 | SETIT_017293mg | TATA-box | TATA | 140 | 148 | core promoter element |
| SiRAF21 | SETIT_017293mg | TATA-box | TATAA | 412 | 422 | core promoter element |
| SiRAF21 | SETIT_017293mg | TATA-box | TATA | 414 | 422 | core promoter element |
| SiRAF21 | SETIT_017293mg | TATA-box | ATTATA | 780 | 792 | core promoter element |
| SiRAF21 | SETIT_017293mg | TATA-box | TATAA | 782 | 792 | core promoter element |
| SiRAF21 | SETIT_017293mg | TATA-box | TATA | 784 | 792 | core promoter element |
| SiRAF21 | SETIT_017293mg | TATA-box | TATA | 1069 | 1077 | core promoter element |
| SiRAF21 | SETIT_017293mg | RY-element | CATGCATG | 738 | 754 | elements involved in seed-specific regulation |
| SiRAF21 | SETIT_017293mg | GCN4_motif | TGAGTCA | 705 | 719 | endosperm expression regulatory element |
| SiRAF21 | SETIT_017293mg | G-box | CACGAC | 1164 | 1176 | light responsive element |
| SiRAF21 | SETIT_017293mg | G-Box | CACGTT | 629 | 641 | light responsive element |
| SiRAF21 | SETIT_017293mg | Sp1 | GGGCGG | 1452 | 1464 | light responsive element |
| SiRAF21 | SETIT_017293mg | GT1-motif | GGTTAA | 42 | 54 | light responsive element |
| SiRAF21 | SETIT_017293mg | GT1-motif | GGTTAA | 424 | 436 | light responsive element |
| SiRAF21 | SETIT_017293mg | Box 4 | ATTAAT | 1881 | 1893 | light responsive element |
| SiRAF21 | SETIT_017293mg | GATA-motif | GATAGGG | 1646 | 1660 | light responsive element |
| SiRAF21 | SETIT_017293mg | LTR | CCGAAA | 834 | 846 | low temperature response element |
| SiRAF21 | SETIT_017293mg | TGACG-motif | TGACG | 685 | 695 | MeJA response regulatory element |
| SiRAF21 | SETIT_017293mg | TGACG-motif | TGACG | 1671 | 1681 | MeJA response regulatory element |
| SiRAF21 | SETIT_017293mg | CGTCA-motif | CGTCA | 685 | 695 | MeJA response regulatory element |
| SiRAF21 | SETIT_017293mg | CGTCA-motif | CGTCA | 1671 | 1681 | MeJA response regulatory element |
| SiRAF21 | SETIT_017293mg | MBS | CAACTG | 1345 | 1357 | MYB binding site involved in drought-inducibility |
| SiRAF21 | SETIT_017293mg | MBS | CAACTG | 1593 | 1605 | MYB binding site involved in drought-inducibility |
| SiRAF21 | SETIT_017293mg | CCAAT-box | CAACGG | 531 | 543 | MYBHv1 binding site |
| SiRAF21 | SETIT_017293mg | A-box | CCGTCC | 1019 | 1031 | promoter and enhancer cis-acting regulatory elements |
| SiRAF21 | SETIT_017293mg | O2-site | GATGATGTGG | 253 | 271 | Zein metabolism regulatory elements |
| SiRAF22 | SETIT_010212mg | ABRE | ACGTG | 474 | 484 | abscisic acid response element |
| SiRAF22 | SETIT_010212mg | ABRE | ACGTG | 982 | 992 | abscisic acid response element |
| SiRAF22 | SETIT_010212mg | ABRE | CACGTG | 986 | 998 | abscisic acid response element |
| SiRAF22 | SETIT_010212mg | ABRE | ACGTG | 988 | 998 | abscisic acid response element |
| SiRAF22 | SETIT_010212mg | ARE | AAACCA | 1150 | 1162 | anaerobic inducing element |
| SiRAF22 | SETIT_010212mg | ARE | AAACCA | 1499 | 1511 | anaerobic inducing element |
| SiRAF22 | SETIT_010212mg | ARE | AAACCA | 1570 | 1582 | anaerobic inducing element |
| SiRAF22 | SETIT_010212mg | ARE | AAACCA | 1603 | 1615 | anaerobic inducing element |
| SiRAF22 | SETIT_010212mg | CAAT-box | CAAAT | 102 | 112 | common cis-acting element |
| SiRAF22 | SETIT_010212mg | CAAT-box | CCAAT | 360 | 370 | common cis-acting element |
| SiRAF22 | SETIT_010212mg | CAAT-box | CCAAT | 385 | 395 | common cis-acting element |
| SiRAF22 | SETIT_010212mg | CAAT-box | TGCCAAC | 1037 | 1051 | common cis-acting element |
| SiRAF22 | SETIT_010212mg | CAAT-box | CAAAT | 1515 | 1525 | common cis-acting element |
| SiRAF22 | SETIT_010212mg | CAAT-box | CAAAT | 1568 | 1578 | common cis-acting element |
| SiRAF22 | SETIT_010212mg | CAAT-box | CCAAT | 1711 | 1721 | common cis-acting element |
| SiRAF22 | SETIT_010212mg | CAAT-box | CAAAT | 1738 | 1748 | common cis-acting element |
| SiRAF22 | SETIT_010212mg | CAAT-box | CAAAT | 1780 | 1790 | common cis-acting element |
| SiRAF22 | SETIT_010212mg | TATA-box | TATA | 28 | 36 | core promoter element |
| SiRAF22 | SETIT_010212mg | TATA-box | ATATAT | 36 | 48 | core promoter element |
| SiRAF22 | SETIT_010212mg | TATA-box | TATA | 39 | 47 | core promoter element |
| SiRAF22 | SETIT_010212mg | TATA-box | TATA | 1771 | 1779 | core promoter element |
| SiRAF22 | SETIT_010212mg | TC-rich repeats | GTTTTCTTAC | 423 | 441 | defense and stress response elements |
| SiRAF22 | SETIT_010212mg | TATC-box | TATCCCA | 1781 | 1795 | gibberellin response element |
| SiRAF22 | SETIT_010212mg | P-box | CCTTTTG | 1531 | 1545 | gibberellin-responsive element |
| SiRAF22 | SETIT_010212mg | GC-motif | CCCCCG | 1418 | 1430 | hypoxia-specifically induced elements |
| SiRAF22 | SETIT_010212mg | G-box | CACGTC | 472 | 484 | light responsive element |
| SiRAF22 | SETIT_010212mg | G-box | CACGAC | 974 | 986 | light responsive element |
| SiRAF22 | SETIT_010212mg | G-box | CACGTC | 981 | 993 | light responsive element |
| SiRAF22 | SETIT_010212mg | G-box | CACGTG | 986 | 998 | light responsive element |
| SiRAF22 | SETIT_010212mg | G-Box | CACGTG | 986 | 998 | light responsive element |
| SiRAF22 | SETIT_010212mg | Sp1 | GGGCGG | 685 | 697 | light responsive element |
| SiRAF22 | SETIT_010212mg | Sp1 | GGGCGG | 1523 | 1535 | light responsive element |
| SiRAF22 | SETIT_010212mg | GT1-motif | GGTTAA | 484 | 496 | light responsive element |
| SiRAF22 | SETIT_010212mg | GT1-motif | GGTTAA | 1179 | 1191 | light responsive element |
| SiRAF22 | SETIT_010212mg | Box 4 | ATTAAT | 905 | 917 | light responsive element |
| SiRAF22 | SETIT_010212mg | TCCC-motif | TCTCCCT | 216 | 230 | light responsive element |
| SiRAF22 | SETIT_010212mg | TCCC-motif | TCTCCCT | 1968 | 1982 | light responsive element |
| SiRAF22 | SETIT_010212mg | GATA-motif | GATAGGA | 1134 | 1148 | light responsive element |
| SiRAF22 | SETIT_010212mg | LTR | CCGAAA | 666 | 678 | low temperature response element |
| SiRAF22 | SETIT_010212mg | LTR | CCGAAA | 1430 | 1442 | low temperature response element |
| SiRAF22 | SETIT_010212mg | LTR | CCGAAA | 1759 | 1771 | low temperature response element |
| SiRAF22 | SETIT_010212mg | TGACG-motif | TGACG | 776 | 786 | MeJA response regulatory element |
| SiRAF22 | SETIT_010212mg | TGACG-motif | TGACG | 984 | 994 | MeJA response regulatory element |
| SiRAF22 | SETIT_010212mg | TGACG-motif | TGACG | 1683 | 1693 | MeJA response regulatory element |
| SiRAF22 | SETIT_010212mg | CGTCA-motif | CGTCA | 776 | 786 | MeJA response regulatory element |
| SiRAF22 | SETIT_010212mg | CGTCA-motif | CGTCA | 984 | 994 | MeJA response regulatory element |
| SiRAF22 | SETIT_010212mg | CGTCA-motif | CGTCA | 1683 | 1693 | MeJA response regulatory element |
| SiRAF22 | SETIT_010212mg | CAT-box | GCCACT | 1922 | 1934 | meristem expression regulatory element |
| SiRAF22 | SETIT_010212mg | MBS | CAACTG | 1036 | 1048 | MYB binding site involved in drought-inducibility |
| SiRAF22 | SETIT_010212mg | MRE | AACCTAA | 1575 | 1589 | MYB binding site involved in light responsiveness |
| SiRAF22 | SETIT_010212mg | CCAAT-box | CAACGG | 962 | 974 | MYBHv1 binding site |
| SiRAF22 | SETIT_010212mg | CCAAT-box | CAACGG | 1130 | 1142 | MYBHv1 binding site |
| SiRAF22 | SETIT_010212mg | A-box | CCGTCC | 1347 | 1359 | promoter and enhancer cis-acting regulatory elements |
| SiRAF22 | SETIT_010212mg | O2-site | GATGACATGG | 1593 | 1611 | Zein metabolism regulatory elements |
| SiRAF23 | SETIT_029452mg | ABRE | GCCGCGTGGC | 268 | 286 | abscisic acid response element |
| SiRAF23 | SETIT_029452mg | ABRE | GCCGCGTGGC | 1946 | 1964 | abscisic acid response element |
| SiRAF23 | SETIT_029452mg | ARE | AAACCA | 1151 | 1163 | anaerobic inducing element |
| SiRAF23 | SETIT_029452mg | ARE | AAACCA | 1299 | 1311 | anaerobic inducing element |
| SiRAF23 | SETIT_029452mg | ARE | AAACCA | 1484 | 1496 | anaerobic inducing element |
| SiRAF23 | SETIT_029452mg | CAAT-box | CCAAT | 11 | 21 | common cis-acting element |
| SiRAF23 | SETIT_029452mg | CAAT-box | CAAAT | 70 | 80 | common cis-acting element |
| SiRAF23 | SETIT_029452mg | CAAT-box | CAAAT | 180 | 190 | common cis-acting element |
| SiRAF23 | SETIT_029452mg | CAAT-box | CCAAT | 346 | 356 | common cis-acting element |
| SiRAF23 | SETIT_029452mg | CAAT-box | CCAAT | 357 | 367 | common cis-acting element |
| SiRAF23 | SETIT_029452mg | CAAT-box | CAAAT | 569 | 579 | common cis-acting element |
| SiRAF23 | SETIT_029452mg | CAAT-box | CAAAT | 776 | 786 | common cis-acting element |
| SiRAF23 | SETIT_029452mg | CAAT-box | CAAAT | 901 | 911 | common cis-acting element |
| SiRAF23 | SETIT_029452mg | CAAT-box | CAAAT | 1045 | 1055 | common cis-acting element |
| SiRAF23 | SETIT_029452mg | CAAT-box | CAAAT | 1133 | 1143 | common cis-acting element |
| SiRAF23 | SETIT_029452mg | CAAT-box | CCAAT | 1249 | 1259 | common cis-acting element |
| SiRAF23 | SETIT_029452mg | CAAT-box | CAAAT | 1326 | 1336 | common cis-acting element |
| SiRAF23 | SETIT_029452mg | CAAT-box | CCCAATTT | 1507 | 1523 | common cis-acting element |
| SiRAF23 | SETIT_029452mg | CAAT-box | CCAAT | 1511 | 1521 | common cis-acting element |
| SiRAF23 | SETIT_029452mg | CAAT-box | CAAAT | 1590 | 1600 | common cis-acting element |
| SiRAF23 | SETIT_029452mg | TATA-box | TACAAAA | 139 | 153 | core promoter element |
| SiRAF23 | SETIT_029452mg | TATA-box | TATATA | 213 | 225 | core promoter element |
| SiRAF23 | SETIT_029452mg | TATA-box | ATATAT | 214 | 226 | core promoter element |
| SiRAF23 | SETIT_029452mg | TATA-box | TATA | 217 | 225 | core promoter element |
| SiRAF23 | SETIT_029452mg | TATA-box | TATA | 368 | 376 | core promoter element |
| SiRAF23 | SETIT_029452mg | TATA-box | TACAAAA | 499 | 513 | core promoter element |
| SiRAF23 | SETIT_029452mg | TATA-box | TACAAAA | 665 | 679 | core promoter element |
| SiRAF23 | SETIT_029452mg | TATA-box | TACAAAA | 850 | 864 | core promoter element |
| SiRAF23 | SETIT_029452mg | TATA-box | TATA | 879 | 887 | core promoter element |
| SiRAF23 | SETIT_029452mg | TATA-box | TATA | 887 | 895 | core promoter element |
| SiRAF23 | SETIT_029452mg | TATA-box | TATA | 965 | 973 | core promoter element |
| SiRAF23 | SETIT_029452mg | TATA-box | ATATAT | 1004 | 1016 | core promoter element |
| SiRAF23 | SETIT_029452mg | TATA-box | TATA | 1007 | 1015 | core promoter element |
| SiRAF23 | SETIT_029452mg | TATA-box | taTATAAAtc | 1007 | 1025 | core promoter element |
| SiRAF23 | SETIT_029452mg | TATA-box | ATATAT | 1027 | 1039 | core promoter element |
| SiRAF23 | SETIT_029452mg | TATA-box | TATATA | 1028 | 1040 | core promoter element |
| SiRAF23 | SETIT_029452mg | TATA-box | ATATAT | 1029 | 1041 | core promoter element |
| SiRAF23 | SETIT_029452mg | TATA-box | TATA | 1032 | 1040 | core promoter element |
| SiRAF23 | SETIT_029452mg | TATA-box | taTATAAAtc | 1032 | 1050 | core promoter element |
| SiRAF23 | SETIT_029452mg | TATA-box | TATA | 1204 | 1212 | core promoter element |
| SiRAF23 | SETIT_029452mg | TATA-box | ccTATAAAaa | 1404 | 1422 | core promoter element |
| SiRAF23 | SETIT_029452mg | TATA-box | TATAAAA | 1407 | 1421 | core promoter element |
| SiRAF23 | SETIT_029452mg | TATA-box | TATAAA | 1409 | 1421 | core promoter element |
| SiRAF23 | SETIT_029452mg | TATA-box | TATAA | 1411 | 1421 | core promoter element |
| SiRAF23 | SETIT_029452mg | TATA-box | TATA | 1413 | 1421 | core promoter element |
| SiRAF23 | SETIT_029452mg | TATA-box | TATAA | 1600 | 1610 | core promoter element |
| SiRAF23 | SETIT_029452mg | TATA-box | TATA | 1602 | 1610 | core promoter element |
| SiRAF23 | SETIT_029452mg | TATA-box | TATA | 1899 | 1907 | core promoter element |
| SiRAF23 | SETIT_029452mg | TC-rich repeats | ATTCTCTAAC | 592 | 610 | defense and stress response elements |
| SiRAF23 | SETIT_029452mg | TC-rich repeats | GTTTTCTTAC | 1484 | 1502 | defense and stress response elements |
| SiRAF23 | SETIT_029452mg | GCN4_motif | TGAGTCA | 1854 | 1868 | endosperm expression regulatory element |
| SiRAF23 | SETIT_029452mg | P-box | CCTTTTG | 27 | 41 | gibberellin-responsive element |
| SiRAF23 | SETIT_029452mg | GARE-motif | TCTGTTG | 788 | 802 | gibberellin-responsive element |
| SiRAF23 | SETIT_029452mg | G-box | CACGAC | 1914 | 1926 | light responsive element |
| SiRAF23 | SETIT_029452mg | Sp1 | GGGCGG | 1819 | 1831 | light responsive element |
| SiRAF23 | SETIT_029452mg | Sp1 | GGGCGG | 1927 | 1939 | light responsive element |
| SiRAF23 | SETIT_029452mg | Sp1 | GGGCGG | 1955 | 1967 | light responsive element |
| SiRAF23 | SETIT_029452mg | Sp1 | GGGCGG | 1984 | 1996 | light responsive element |
| SiRAF23 | SETIT_029452mg | Sp1 | GGGCGG | 1988 | 2000 | light responsive element |
| SiRAF23 | SETIT_029452mg | ATC-motif | TGCTATCCA | 740 | 758 | light responsive element |
| SiRAF23 | SETIT_029452mg | TCT-motif | TCTTAC | 1491 | 1503 | light responsive element |
| SiRAF23 | SETIT_029452mg | A-box | CCGTCC | 1232 | 1244 | promoter and enhancer cis-acting regulatory elements |
| SiRAF23 | SETIT_029452mg | TCA-element | CCATCTTTTT | 920 | 938 | salicylic acid response element |
| SiRAF23 | SETIT_029452mg | O2-site | GATGACATGG | 242 | 260 | Zein metabolism regulatory elements |
| SiRAF24 | SETIT_001285mg | ABRE | ACGTG | 17 | 27 | abscisic acid response element |
| SiRAF24 | SETIT_001285mg | ABRE | ACGTG | 584 | 594 | abscisic acid response element |
| SiRAF24 | SETIT_001285mg | ABRE | ACGTG | 878 | 888 | abscisic acid response element |
| SiRAF24 | SETIT_001285mg | ABRE | ACGTG | 1551 | 1561 | abscisic acid response element |
| SiRAF24 | SETIT_001285mg | ABRE | ACGTG | 1634 | 1644 | abscisic acid response element |
| SiRAF24 | SETIT_001285mg | ARE | AAACCA | 19 | 31 | anaerobic inducing element |
| SiRAF24 | SETIT_001285mg | ARE | AAACCA | 927 | 939 | anaerobic inducing element |
| SiRAF24 | SETIT_001285mg | ARE | AAACCA | 1280 | 1292 | anaerobic inducing element |
| SiRAF24 | SETIT_001285mg | TGA-element | AACGAC | 741 | 753 | auxin-responsive element |
| SiRAF24 | SETIT_001285mg | TGA-element | AACGAC | 1349 | 1361 | auxin-responsive element |
| SiRAF24 | SETIT_001285mg | CAAT-box | CCAAT | 285 | 295 | common cis-acting element |
| SiRAF24 | SETIT_001285mg | CAAT-box | CAAAT | 669 | 679 | common cis-acting element |
| SiRAF24 | SETIT_001285mg | CAAT-box | CAAAT | 694 | 704 | common cis-acting element |
| SiRAF24 | SETIT_001285mg | CAAT-box | CAAAT | 1018 | 1028 | common cis-acting element |
| SiRAF24 | SETIT_001285mg | CAAT-box | CCAAT | 1058 | 1068 | common cis-acting element |
| SiRAF24 | SETIT_001285mg | CAAT-box | CAAAT | 1358 | 1368 | common cis-acting element |
| SiRAF24 | SETIT_001285mg | CAAT-box | CCAAT | 1389 | 1399 | common cis-acting element |
| SiRAF24 | SETIT_001285mg | CAAT-box | CAAAT | 1399 | 1409 | common cis-acting element |
| SiRAF24 | SETIT_001285mg | CAAT-box | CAAAT | 1408 | 1418 | common cis-acting element |
| SiRAF24 | SETIT_001285mg | CAAT-box | CCAAT | 1420 | 1430 | common cis-acting element |
| SiRAF24 | SETIT_001285mg | CAAT-box | CAAAT | 1429 | 1439 | common cis-acting element |
| SiRAF24 | SETIT_001285mg | CAAT-box | CAACCAACTCC | 1723 | 1743 | common cis-acting element |
| SiRAF24 | SETIT_001285mg | TATA-box | TATA | 549 | 557 | core promoter element |
| SiRAF24 | SETIT_001285mg | TATA-box | ATATAA | 1382 | 1394 | core promoter element |
| SiRAF24 | SETIT_001285mg | TATA-box | TATA | 1385 | 1393 | core promoter element |
| SiRAF24 | SETIT_001285mg | TATA-box | TATA | 1416 | 1424 | core promoter element |
| SiRAF24 | SETIT_001285mg | TATA-box | TATA | 1434 | 1442 | core promoter element |
| SiRAF24 | SETIT_001285mg | RY-element | CATGCATG | 297 | 313 | elements involved in seed-specific regulation |
| SiRAF24 | SETIT_001285mg | GARE-motif | TCTGTTG | 170 | 184 | gibberellin-responsive element |
| SiRAF24 | SETIT_001285mg | GARE-motif | TCTGTTG | 205 | 219 | gibberellin-responsive element |
| SiRAF24 | SETIT_001285mg | G-box | CACGTC | 582 | 594 | light responsive element |
| SiRAF24 | SETIT_001285mg | G-box | CACGTC | 877 | 889 | light responsive element |
| SiRAF24 | SETIT_001285mg | G-box | CACGAC | 1527 | 1539 | light responsive element |
| SiRAF24 | SETIT_001285mg | G-box | CACGTC | 1550 | 1562 | light responsive element |
| SiRAF24 | SETIT_001285mg | G-box | CACGTC | 1633 | 1645 | light responsive element |
| SiRAF24 | SETIT_001285mg | G-Box | CACGTT | 15 | 27 | light responsive element |
| SiRAF24 | SETIT_001285mg | Sp1 | GGGCGG | 87 | 99 | light responsive element |
| SiRAF24 | SETIT_001285mg | Sp1 | GGGCGG | 1893 | 1905 | light responsive element |
| SiRAF24 | SETIT_001285mg | GT1-motif | GGTTAAT | 44 | 58 | light responsive element |
| SiRAF24 | SETIT_001285mg | I-box | TAGATAACC | 860 | 878 | light responsive element |
| SiRAF24 | SETIT_001285mg | I-box | ccttatcct | 979 | 997 | light responsive element |
| SiRAF24 | SETIT_001285mg | I-box | AAGATAAGGCT | 1300 | 1320 | light responsive element |
| SiRAF24 | SETIT_001285mg | GATA-motif | AAGGATAAGG | 979 | 997 | light responsive element |
| SiRAF24 | SETIT_001285mg | LTR | CCGAAA | 1570 | 1582 | low temperature response element |
| SiRAF24 | SETIT_001285mg | LTR | CCGAAA | 1946 | 1958 | low temperature response element |
| SiRAF24 | SETIT_001285mg | TGACG-motif | TGACG | 519 | 529 | MeJA response regulatory element |
| SiRAF24 | SETIT_001285mg | TGACG-motif | TGACG | 735 | 745 | MeJA response regulatory element |
| SiRAF24 | SETIT_001285mg | TGACG-motif | TGACG | 948 | 958 | MeJA response regulatory element |
| SiRAF24 | SETIT_001285mg | TGACG-motif | TGACG | 1553 | 1563 | MeJA response regulatory element |
| SiRAF24 | SETIT_001285mg | TGACG-motif | TGACG | 1636 | 1646 | MeJA response regulatory element |
| SiRAF24 | SETIT_001285mg | TGACG-motif | TGACG | 1941 | 1951 | MeJA response regulatory element |
| SiRAF24 | SETIT_001285mg | TGACG-motif | TGACG | 1985 | 1995 | MeJA response regulatory element |
| SiRAF24 | SETIT_001285mg | CGTCA-motif | CGTCA | 519 | 529 | MeJA response regulatory element |
| SiRAF24 | SETIT_001285mg | CGTCA-motif | CGTCA | 735 | 745 | MeJA response regulatory element |
| SiRAF24 | SETIT_001285mg | CGTCA-motif | CGTCA | 948 | 958 | MeJA response regulatory element |
| SiRAF24 | SETIT_001285mg | CGTCA-motif | CGTCA | 1553 | 1563 | MeJA response regulatory element |
| SiRAF24 | SETIT_001285mg | CGTCA-motif | CGTCA | 1636 | 1646 | MeJA response regulatory element |
| SiRAF24 | SETIT_001285mg | CGTCA-motif | CGTCA | 1941 | 1951 | MeJA response regulatory element |
| SiRAF24 | SETIT_001285mg | CGTCA-motif | CGTCA | 1985 | 1995 | MeJA response regulatory element |
| SiRAF24 | SETIT_001285mg | CAT-box | GCCACT | 225 | 237 | meristem expression regulatory element |
| SiRAF24 | SETIT_001285mg | CAT-box | GCCACT | 1375 | 1387 | meristem expression regulatory element |
| SiRAF24 | SETIT_001285mg | CCAAT-box | CAACGG | 1699 | 1711 | MYBHv1 binding site |
| SiRAF24 | SETIT_001285mg | A-box | CCGTCC | 1677 | 1689 | promoter and enhancer cis-acting regulatory elements |
| SiRAF24 | SETIT_001285mg | O2-site | GTTGACGTGA | 1629 | 1647 | Zein metabolism regulatory elements |
| SiRAF25 | SETIT_036191mg | ABRE | CACGTG | 572 | 584 | abscisic acid response element |
| SiRAF25 | SETIT_036191mg | ABRE | ACGTG | 574 | 584 | abscisic acid response element |
| SiRAF25 | SETIT_036191mg | ABRE | GACACGTGGC | 1205 | 1223 | abscisic acid response element |
| SiRAF25 | SETIT_036191mg | ABRE | ACGTG | 1212 | 1222 | abscisic acid response element |
| SiRAF25 | SETIT_036191mg | ABRE | ACGTG | 1735 | 1745 | abscisic acid response element |
| SiRAF25 | SETIT_036191mg | ARE | AAACCA | 968 | 980 | anaerobic inducing element |
| SiRAF25 | SETIT_036191mg | ARE | AAACCA | 1001 | 1013 | anaerobic inducing element |
| SiRAF25 | SETIT_036191mg | CAAT-box | CAAAT | 238 | 248 | common cis-acting element |
| SiRAF25 | SETIT_036191mg | CAAT-box | CCAAT | 355 | 365 | common cis-acting element |
| SiRAF25 | SETIT_036191mg | CAAT-box | CAAAT | 390 | 400 | common cis-acting element |
| SiRAF25 | SETIT_036191mg | CAAT-box | CAAAT | 409 | 419 | common cis-acting element |
| SiRAF25 | SETIT_036191mg | CAAT-box | CCAAT | 505 | 515 | common cis-acting element |
| SiRAF25 | SETIT_036191mg | CAAT-box | CAAAT | 633 | 643 | common cis-acting element |
| SiRAF25 | SETIT_036191mg | CAAT-box | CAAAT | 722 | 732 | common cis-acting element |
| SiRAF25 | SETIT_036191mg | CAAT-box | CAAAT | 831 | 841 | common cis-acting element |
| SiRAF25 | SETIT_036191mg | CAAT-box | CAAAT | 870 | 880 | common cis-acting element |
| SiRAF25 | SETIT_036191mg | CAAT-box | CAAAT | 887 | 897 | common cis-acting element |
| SiRAF25 | SETIT_036191mg | CAAT-box | CCAAT | 936 | 946 | common cis-acting element |
| SiRAF25 | SETIT_036191mg | CAAT-box | CCAAT | 947 | 957 | common cis-acting element |
| SiRAF25 | SETIT_036191mg | CAAT-box | CAAAT | 998 | 1008 | common cis-acting element |
| SiRAF25 | SETIT_036191mg | CAAT-box | CAAAT | 1401 | 1411 | common cis-acting element |
| SiRAF25 | SETIT_036191mg | CAAT-box | CCAAT | 1463 | 1473 | common cis-acting element |
| SiRAF25 | SETIT_036191mg | CAAT-box | CCAAT | 1466 | 1476 | common cis-acting element |
| SiRAF25 | SETIT_036191mg | CAAT-box | CCAAT | 1539 | 1549 | common cis-acting element |
| SiRAF25 | SETIT_036191mg | CAAT-box | CCAAT | 1744 | 1754 | common cis-acting element |
| SiRAF25 | SETIT_036191mg | CAAT-box | CAAAT | 1765 | 1775 | common cis-acting element |
| SiRAF25 | SETIT_036191mg | CAAT-box | CAAAT | 1826 | 1836 | common cis-acting element |
| SiRAF25 | SETIT_036191mg | TATA-box | TATTTAAA | 54 | 70 | core promoter element |
| SiRAF25 | SETIT_036191mg | TATA-box | TATTTAAA | 56 | 72 | core promoter element |
| SiRAF25 | SETIT_036191mg | TATA-box | TATAAAA | 99 | 113 | core promoter element |
| SiRAF25 | SETIT_036191mg | TATA-box | TATAAA | 101 | 113 | core promoter element |
| SiRAF25 | SETIT_036191mg | TATA-box | TATAA | 103 | 113 | core promoter element |
| SiRAF25 | SETIT_036191mg | TATA-box | TATA | 105 | 113 | core promoter element |
| SiRAF25 | SETIT_036191mg | TATA-box | ATTATA | 272 | 284 | core promoter element |
| SiRAF25 | SETIT_036191mg | TATA-box | TATAA | 274 | 284 | core promoter element |
| SiRAF25 | SETIT_036191mg | TATA-box | TATA | 276 | 284 | core promoter element |
| SiRAF25 | SETIT_036191mg | TATA-box | taTATAAAtc | 396 | 414 | core promoter element |
| SiRAF25 | SETIT_036191mg | TATA-box | ATATAT | 400 | 412 | core promoter element |
| SiRAF25 | SETIT_036191mg | TATA-box | TATATA | 401 | 413 | core promoter element |
| SiRAF25 | SETIT_036191mg | TATA-box | ATATAT | 402 | 414 | core promoter element |
| SiRAF25 | SETIT_036191mg | TATA-box | TATATA | 403 | 415 | core promoter element |
| SiRAF25 | SETIT_036191mg | TATA-box | ATATAT | 404 | 416 | core promoter element |
| SiRAF25 | SETIT_036191mg | TATA-box | TATA | 407 | 415 | core promoter element |
| SiRAF25 | SETIT_036191mg | TATA-box | TATA | 461 | 469 | core promoter element |
| SiRAF25 | SETIT_036191mg | TATA-box | TATAA | 629 | 639 | core promoter element |
| SiRAF25 | SETIT_036191mg | TATA-box | TATA | 631 | 639 | core promoter element |
| SiRAF25 | SETIT_036191mg | TATA-box | TATACA | 647 | 659 | core promoter element |
| SiRAF25 | SETIT_036191mg | TATA-box | TATA | 651 | 659 | core promoter element |
| SiRAF25 | SETIT_036191mg | TATA-box | ATATAT | 691 | 703 | core promoter element |
| SiRAF25 | SETIT_036191mg | TATA-box | TATA | 694 | 702 | core promoter element |
| SiRAF25 | SETIT_036191mg | TATA-box | TATAA | 718 | 728 | core promoter element |
| SiRAF25 | SETIT_036191mg | TATA-box | TATA | 720 | 728 | core promoter element |
| SiRAF25 | SETIT_036191mg | TATA-box | TATA | 759 | 767 | core promoter element |
| SiRAF25 | SETIT_036191mg | TATA-box | ATATAT | 781 | 793 | core promoter element |
| SiRAF25 | SETIT_036191mg | TATA-box | TATA | 784 | 792 | core promoter element |
| SiRAF25 | SETIT_036191mg | TATA-box | TATA | 848 | 856 | core promoter element |
| SiRAF25 | SETIT_036191mg | TATA-box | TATA | 892 | 900 | core promoter element |
| SiRAF25 | SETIT_036191mg | TATA-box | TATAAAT | 976 | 990 | core promoter element |
| SiRAF25 | SETIT_036191mg | TATA-box | TATAAA | 978 | 990 | core promoter element |
| SiRAF25 | SETIT_036191mg | TATA-box | TATAA | 980 | 990 | core promoter element |
| SiRAF25 | SETIT_036191mg | TATA-box | TATA | 982 | 990 | core promoter element |
| SiRAF25 | SETIT_036191mg | TATA-box | TATA | 1013 | 1021 | core promoter element |
| SiRAF25 | SETIT_036191mg | GC-motif | CCCCCG | 1367 | 1379 | hypoxia-specifically induced elements |
| SiRAF25 | SETIT_036191mg | G-Box | CACGTG | 572 | 584 | light responsive element |
| SiRAF25 | SETIT_036191mg | G-Box | CACGTT | 1734 | 1746 | light responsive element |
| SiRAF25 | SETIT_036191mg | G-box | TAACACGTAG | 67 | 85 | light responsive element |
| SiRAF25 | SETIT_036191mg | G-box | CACGTG | 572 | 584 | light responsive element |
| SiRAF25 | SETIT_036191mg | G-box | CACGTC | 1210 | 1222 | light responsive element |
| SiRAF25 | SETIT_036191mg | Sp1 | GGGCGG | 1303 | 1315 | light responsive element |
| SiRAF25 | SETIT_036191mg | Sp1 | GGGCGG | 1357 | 1369 | light responsive element |
| SiRAF25 | SETIT_036191mg | Box 4 | ATTAAT | 542 | 554 | light responsive element |
| SiRAF25 | SETIT_036191mg | Box 4 | ATTAAT | 1085 | 1097 | light responsive element |
| SiRAF25 | SETIT_036191mg | GATA-motif | GATAGGA | 132 | 146 | light responsive element |
| SiRAF25 | SETIT_036191mg | GATA-motif | AAGATAAGATT | 1021 | 1041 | light responsive element |
| SiRAF25 | SETIT_036191mg | TCT-motif | TCTTAC | 817 | 829 | light responsive element |
| SiRAF25 | SETIT_036191mg | AE-box | AGAAACTT | 191 | 207 | light responsive element |
| SiRAF25 | SETIT_036191mg | CGTCA-motif | CGTCA | 1344 | 1354 | MeJA response regulatory element |
| SiRAF25 | SETIT_036191mg | CGTCA-motif | CGTCA | 1590 | 1600 | MeJA response regulatory element |
| SiRAF25 | SETIT_036191mg | TGACG-motif | TGACG | 1344 | 1354 | MeJA response regulatory element |
| SiRAF25 | SETIT_036191mg | TGACG-motif | TGACG | 1590 | 1600 | MeJA response regulatory element |
| SiRAF25 | SETIT_036191mg | CAT-box | GCCACT | 1327 | 1339 | meristem expression regulatory element |
| SiRAF25 | SETIT_036191mg | A-box | CCGTCC | 1246 | 1258 | promoter and enhancer cis-acting regulatory elements |
| SiRAF25 | SETIT_036191mg | Unnamed__1 | GAATTTAATTAA | 512 | 534 | protein binding site |
| SiRAF26 | SETIT_035834mg | ABRE | ACGTG | 491 | 501 | abscisic acid response element |
| SiRAF26 | SETIT_035834mg | ABRE | ACGTG | 533 | 543 | abscisic acid response element |
| SiRAF26 | SETIT_035834mg | ABRE | CACGTG | 579 | 591 | abscisic acid response element |
| SiRAF26 | SETIT_035834mg | ABRE | ACGTG | 581 | 591 | abscisic acid response element |
| SiRAF26 | SETIT_035834mg | ABRE | ACGTG | 831 | 841 | abscisic acid response element |
| SiRAF26 | SETIT_035834mg | ABRE | ACGTG | 925 | 935 | abscisic acid response element |
| SiRAF26 | SETIT_035834mg | ABRE | ACGTG | 1063 | 1073 | abscisic acid response element |
| SiRAF26 | SETIT_035834mg | ARE | AAACCA | 1201 | 1213 | anaerobic inducing element |
| SiRAF26 | SETIT_035834mg | ARE | AAACCA | 1227 | 1239 | anaerobic inducing element |
| SiRAF26 | SETIT_035834mg | ARE | AAACCA | 1625 | 1637 | anaerobic inducing element |
| SiRAF26 | SETIT_035834mg | ARE | AAACCA | 1795 | 1807 | anaerobic inducing element |
| SiRAF26 | SETIT_035834mg | TGA-element | AACGAC | 795 | 807 | auxin-responsive element |
| SiRAF26 | SETIT_035834mg | TGA-element | AACGAC | 942 | 954 | auxin-responsive element |
| SiRAF26 | SETIT_035834mg | TGA-element | AACGAC | 1828 | 1840 | auxin-responsive element |
| SiRAF26 | SETIT_035834mg | circadian | CAAAGATATC | 1003 | 1021 | circadian rhythm control element |
| SiRAF26 | SETIT_035834mg | CAAT-box | CCAAT | 100 | 110 | common cis-acting element |
| SiRAF26 | SETIT_035834mg | CAAT-box | CAAAT | 179 | 189 | common cis-acting element |
| SiRAF26 | SETIT_035834mg | CAAT-box | CAAAT | 346 | 356 | common cis-acting element |
| SiRAF26 | SETIT_035834mg | CAAT-box | CAAAT | 409 | 419 | common cis-acting element |
| SiRAF26 | SETIT_035834mg | CAAT-box | CAAAT | 463 | 473 | common cis-acting element |
| SiRAF26 | SETIT_035834mg | CAAT-box | CCAAT | 515 | 525 | common cis-acting element |
| SiRAF26 | SETIT_035834mg | CAAT-box | CAAAT | 613 | 623 | common cis-acting element |
| SiRAF26 | SETIT_035834mg | CAAT-box | CCAAT | 716 | 726 | common cis-acting element |
| SiRAF26 | SETIT_035834mg | CAAT-box | CCAAT | 851 | 861 | common cis-acting element |
| SiRAF26 | SETIT_035834mg | CAAT-box | CCAAT | 1027 | 1037 | common cis-acting element |
| SiRAF26 | SETIT_035834mg | CAAT-box | CAAAT | 1211 | 1221 | common cis-acting element |
| SiRAF26 | SETIT_035834mg | CAAT-box | CAAAT | 1251 | 1261 | common cis-acting element |
| SiRAF26 | SETIT_035834mg | CAAT-box | CCAAT | 1346 | 1356 | common cis-acting element |
| SiRAF26 | SETIT_035834mg | CAAT-box | CAAAT | 1542 | 1552 | common cis-acting element |
| SiRAF26 | SETIT_035834mg | CAAT-box | CCAAT | 1950 | 1960 | common cis-acting element |
| SiRAF26 | SETIT_035834mg | TATA-box | TATA | 31 | 39 | core promoter element |
| SiRAF26 | SETIT_035834mg | TATA-box | TATATAA | 75 | 89 | core promoter element |
| SiRAF26 | SETIT_035834mg | TATA-box | TATATA | 77 | 89 | core promoter element |
| SiRAF26 | SETIT_035834mg | TATA-box | ATATAT | 78 | 90 | core promoter element |
| SiRAF26 | SETIT_035834mg | TATA-box | TATA | 81 | 89 | core promoter element |
| SiRAF26 | SETIT_035834mg | TATA-box | TATACA | 83 | 95 | core promoter element |
| SiRAF26 | SETIT_035834mg | TATA-box | TATA | 87 | 95 | core promoter element |
| SiRAF26 | SETIT_035834mg | TATA-box | TATA | 117 | 125 | core promoter element |
| SiRAF26 | SETIT_035834mg | TATA-box | ATATAT | 124 | 136 | core promoter element |
| SiRAF26 | SETIT_035834mg | TATA-box | TATA | 127 | 135 | core promoter element |
| SiRAF26 | SETIT_035834mg | TATA-box | TATAAAA | 141 | 155 | core promoter element |
| SiRAF26 | SETIT_035834mg | TATA-box | TATAAA | 143 | 155 | core promoter element |
| SiRAF26 | SETIT_035834mg | TATA-box | TATAA | 145 | 155 | core promoter element |
| SiRAF26 | SETIT_035834mg | TATA-box | TATA | 147 | 155 | core promoter element |
| SiRAF26 | SETIT_035834mg | TATA-box | ATTATA | 257 | 269 | core promoter element |
| SiRAF26 | SETIT_035834mg | TATA-box | TATAA | 259 | 269 | core promoter element |
| SiRAF26 | SETIT_035834mg | TATA-box | TATA | 261 | 269 | core promoter element |
| SiRAF26 | SETIT_035834mg | TATA-box | TATTTAAA | 259 | 275 | core promoter element |
| SiRAF26 | SETIT_035834mg | TATA-box | ATATAA | 478 | 490 | core promoter element |
| SiRAF26 | SETIT_035834mg | TATA-box | TATA | 481 | 489 | core promoter element |
| SiRAF26 | SETIT_035834mg | TATA-box | TATA | 508 | 516 | core promoter element |
| SiRAF26 | SETIT_035834mg | TATA-box | TATAA | 525 | 535 | core promoter element |
| SiRAF26 | SETIT_035834mg | TATA-box | TATA | 527 | 535 | core promoter element |
| SiRAF26 | SETIT_035834mg | TATA-box | TATA | 608 | 616 | core promoter element |
| SiRAF26 | SETIT_035834mg | TATA-box | TATA | 829 | 837 | core promoter element |
| SiRAF26 | SETIT_035834mg | TATA-box | TATA | 891 | 899 | core promoter element |
| SiRAF26 | SETIT_035834mg | TATA-box | TATA | 904 | 912 | core promoter element |
| SiRAF26 | SETIT_035834mg | TATA-box | TATA | 1032 | 1040 | core promoter element |
| SiRAF26 | SETIT_035834mg | TATA-box | ccTATAAAaa | 1407 | 1425 | core promoter element |
| SiRAF26 | SETIT_035834mg | TATA-box | TATA | 1414 | 1422 | core promoter element |
| SiRAF26 | SETIT_035834mg | TATC-box | TATCCCA | 674 | 688 | gibberellin response element |
| SiRAF26 | SETIT_035834mg | GC-motif | CCCCCG | 1334 | 1346 | hypoxia-specifically induced elements |
| SiRAF26 | SETIT_035834mg | GC-motif | CCCCCG | 1443 | 1455 | hypoxia-specifically induced elements |
| SiRAF26 | SETIT_035834mg | ACE | CTAACGTATT | 771 | 789 | light responsive element |
| SiRAF26 | SETIT_035834mg | G-box | CACGTC | 489 | 501 | light responsive element |
| SiRAF26 | SETIT_035834mg | G-box | CACGTG | 579 | 591 | light responsive element |
| SiRAF26 | SETIT_035834mg | G-box | TACGTG | 829 | 841 | light responsive element |
| SiRAF26 | SETIT_035834mg | G-box | CACGTC | 923 | 935 | light responsive element |
| SiRAF26 | SETIT_035834mg | G-box | CACGTC | 1061 | 1073 | light responsive element |
| SiRAF26 | SETIT_035834mg | G-box | GCCACGTGGA | 1233 | 1251 | light responsive element |
| SiRAF26 | SETIT_035834mg | G-box | CACGAC | 1490 | 1502 | light responsive element |
| SiRAF26 | SETIT_035834mg | G-Box | CACGTT | 531 | 543 | light responsive element |
| SiRAF26 | SETIT_035834mg | G-Box | CACGTG | 579 | 591 | light responsive element |
| SiRAF26 | SETIT_035834mg | ATCT-motif | AATCTAATCC | 1540 | 1558 | light responsive element |
| SiRAF26 | SETIT_035834mg | GATA-motif | AAGGATAAGG | 554 | 572 | light responsive element |
| SiRAF26 | SETIT_035834mg | GATA-motif | GATAGGA | 948 | 962 | light responsive element |
| SiRAF26 | SETIT_035834mg | GATA-motif | GATAGGA | 1019 | 1033 | light responsive element |
| SiRAF26 | SETIT_035834mg | GATA-motif | GATAGGA | 1086 | 1100 | light responsive element |
| SiRAF26 | SETIT_035834mg | TCT-motif | TCTTAC | 66 | 78 | light responsive element |
| SiRAF26 | SETIT_035834mg | TCT-motif | TCTTAC | 1713 | 1725 | light responsive element |
| SiRAF26 | SETIT_035834mg | Gap-box | CAAATGAA(A/G)A | 341.5 | 360.5 | light responsive element |
| SiRAF26 | SETIT_035834mg | TGACG-motif | TGACG | 489 | 499 | MeJA response regulatory element |
| SiRAF26 | SETIT_035834mg | TGACG-motif | TGACG | 638 | 648 | MeJA response regulatory element |
| SiRAF26 | SETIT_035834mg | TGACG-motif | TGACG | 776 | 786 | MeJA response regulatory element |
| SiRAF26 | SETIT_035834mg | TGACG-motif | TGACG | 923 | 933 | MeJA response regulatory element |
| SiRAF26 | SETIT_035834mg | TGACG-motif | TGACG | 1500 | 1510 | MeJA response regulatory element |
| SiRAF26 | SETIT_035834mg | CGTCA-motif | CGTCA | 489 | 499 | MeJA response regulatory element |
| SiRAF26 | SETIT_035834mg | CGTCA-motif | CGTCA | 638 | 648 | MeJA response regulatory element |
| SiRAF26 | SETIT_035834mg | CGTCA-motif | CGTCA | 776 | 786 | MeJA response regulatory element |
| SiRAF26 | SETIT_035834mg | CGTCA-motif | CGTCA | 923 | 933 | MeJA response regulatory element |
| SiRAF26 | SETIT_035834mg | CGTCA-motif | CGTCA | 1500 | 1510 | MeJA response regulatory element |
| SiRAF26 | SETIT_035834mg | CAT-box | GCCACT | 340 | 352 | meristem expression regulatory element |
| SiRAF26 | SETIT_035834mg | TCA-element | CCATCTTTTT | 145 | 163 | salicylic acid response element |
| SiRAF26 | SETIT_035834mg | TCA-element | CCATCTTTTT | 306 | 324 | salicylic acid response element |
| SiRAF27 | SETIT_017658mg | ABRE | ACGTG | 1273 | 1283 | abscisic acid response element |
| SiRAF27 | SETIT_017658mg | ABRE | ACGTG | 1370 | 1380 | abscisic acid response element |
| SiRAF27 | SETIT_017658mg | ABRE | CACGTG | 1744 | 1756 | abscisic acid response element |
| SiRAF27 | SETIT_017658mg | ABRE | ACGTG | 1746 | 1756 | abscisic acid response element |
| SiRAF27 | SETIT_017658mg | ARE | AAACCA | 1013 | 1025 | anaerobic inducing element |
| SiRAF27 | SETIT_017658mg | circadian | CAAAGATATC | 866 | 884 | circadian rhythm control element |
| SiRAF27 | SETIT_017658mg | CAAT-box | CAAAT | 63 | 73 | common cis-acting element |
| SiRAF27 | SETIT_017658mg | CAAT-box | CAAAT | 138 | 148 | common cis-acting element |
| SiRAF27 | SETIT_017658mg | CAAT-box | CAAAT | 278 | 288 | common cis-acting element |
| SiRAF27 | SETIT_017658mg | CAAT-box | CAAAT | 287 | 297 | common cis-acting element |
| SiRAF27 | SETIT_017658mg | CAAT-box | CCAAT | 306 | 316 | common cis-acting element |
| SiRAF27 | SETIT_017658mg | CAAT-box | CAAAT | 340 | 350 | common cis-acting element |
| SiRAF27 | SETIT_017658mg | CAAT-box | CAAAT | 399 | 409 | common cis-acting element |
| SiRAF27 | SETIT_017658mg | CAAT-box | CAAAT | 626 | 636 | common cis-acting element |
| SiRAF27 | SETIT_017658mg | CAAT-box | CAAAT | 1315 | 1325 | common cis-acting element |
| SiRAF27 | SETIT_017658mg | CAAT-box | CAAAT | 1397 | 1407 | common cis-acting element |
| SiRAF27 | SETIT_017658mg | CAAT-box | CAAAT | 1512 | 1522 | common cis-acting element |
| SiRAF27 | SETIT_017658mg | CAAT-box | CAAAT | 1542 | 1552 | common cis-acting element |
| SiRAF27 | SETIT_017658mg | CAAT-box | CCAAT | 1582 | 1592 | common cis-acting element |
| SiRAF27 | SETIT_017658mg | CAAT-box | CAAAT | 1616 | 1626 | common cis-acting element |
| SiRAF27 | SETIT_017658mg | CAAT-box | CAAAT | 1733 | 1743 | common cis-acting element |
| SiRAF27 | SETIT_017658mg | CAAT-box | CAAAT | 1820 | 1830 | common cis-acting element |
| SiRAF27 | SETIT_017658mg | TATA-box | TATA | 41 | 49 | core promoter element |
| SiRAF27 | SETIT_017658mg | TATA-box | TATA | 196 | 204 | core promoter element |
| SiRAF27 | SETIT_017658mg | TATA-box | TATA | 917 | 925 | core promoter element |
| SiRAF27 | SETIT_017658mg | TATA-box | TATA | 979 | 987 | core promoter element |
| SiRAF27 | SETIT_017658mg | TATA-box | TACAAAA | 1282 | 1296 | core promoter element |
| SiRAF27 | SETIT_017658mg | TATA-box | TATA | 1432 | 1440 | core promoter element |
| SiRAF27 | SETIT_017658mg | TATA-box | TATAAAT | 1434 | 1448 | core promoter element |
| SiRAF27 | SETIT_017658mg | TATA-box | TATAAA | 1436 | 1448 | core promoter element |
| SiRAF27 | SETIT_017658mg | TATA-box | TATAA | 1438 | 1448 | core promoter element |
| SiRAF27 | SETIT_017658mg | TATA-box | TATA | 1440 | 1448 | core promoter element |
| SiRAF27 | SETIT_017658mg | TATA-box | TATAA | 1480 | 1490 | core promoter element |
| SiRAF27 | SETIT_017658mg | TATA-box | TATA | 1482 | 1490 | core promoter element |
| SiRAF27 | SETIT_017658mg | TATA-box | ATATAT | 1507 | 1519 | core promoter element |
| SiRAF27 | SETIT_017658mg | TATA-box | TATA | 1510 | 1518 | core promoter element |
| SiRAF27 | SETIT_017658mg | TATA-box | ATATAT | 1648 | 1660 | core promoter element |
| SiRAF27 | SETIT_017658mg | TATA-box | TATATA | 1649 | 1661 | core promoter element |
| SiRAF27 | SETIT_017658mg | TATA-box | ATATAT | 1650 | 1662 | core promoter element |
| SiRAF27 | SETIT_017658mg | TATA-box | TATATA | 1651 | 1663 | core promoter element |
| SiRAF27 | SETIT_017658mg | TATA-box | TATA | 1655 | 1663 | core promoter element |
| SiRAF27 | SETIT_017658mg | TATA-box | TATA | 1856 | 1864 | core promoter element |
| SiRAF27 | SETIT_017658mg | TATA-box | TATAA | 1885 | 1895 | core promoter element |
| SiRAF27 | SETIT_017658mg | TATA-box | TATA | 1887 | 1895 | core promoter element |
| SiRAF27 | SETIT_017658mg | P-box | CCTTTTG | 1623 | 1637 | gibberellin-responsive element |
| SiRAF27 | SETIT_017658mg | GC-motif | CCCCCG | 295 | 307 | hypoxia-specifically induced elements |
| SiRAF27 | SETIT_017658mg | ACE | GACACGTATG | 753 | 771 | light responsive element |
| SiRAF27 | SETIT_017658mg | G-box | TAACACGTAG | 263 | 281 | light responsive element |
| SiRAF27 | SETIT_017658mg | G-box | CACGTC | 1271 | 1283 | light responsive element |
| SiRAF27 | SETIT_017658mg | G-box | CACGTG | 1744 | 1756 | light responsive element |
| SiRAF27 | SETIT_017658mg | G-box | CACGAC | 1950 | 1962 | light responsive element |
| SiRAF27 | SETIT_017658mg | G-Box | CACGTT | 1368 | 1380 | light responsive element |
| SiRAF27 | SETIT_017658mg | G-Box | CACGTG | 1744 | 1756 | light responsive element |
| SiRAF27 | SETIT_017658mg | Sp1 | GGGCGG | 292 | 304 | light responsive element |
| SiRAF27 | SETIT_017658mg | Sp1 | GGGCGG | 500 | 512 | light responsive element |
| SiRAF27 | SETIT_017658mg | Sp1 | GGGCGG | 767 | 779 | light responsive element |
| SiRAF27 | SETIT_017658mg | Sp1 | GGGCGG | 933 | 945 | light responsive element |
| SiRAF27 | SETIT_017658mg | ATC-motif | AGCTATCCA | 1782 | 1800 | light responsive element |
| SiRAF27 | SETIT_017658mg | chs-CMA2a | TCACTTGA | 1462 | 1478 | light responsive element |
| SiRAF27 | SETIT_017658mg | TCCC-motif | TCTCCCT | 675 | 689 | light responsive element |
| SiRAF27 | SETIT_017658mg | TGACG-motif | TGACG | 1271 | 1281 | MeJA response regulatory element |
| SiRAF27 | SETIT_017658mg | CGTCA-motif | CGTCA | 1271 | 1281 | MeJA response regulatory element |
| SiRAF27 | SETIT_017658mg | CAT-box | GCCACT | 1006 | 1018 | meristem expression regulatory element |
| SiRAF27 | SETIT_017658mg | MBS | CAACTG | 1444 | 1456 | MYB binding site involved in drought-inducibility |
| SiRAF27 | SETIT_017658mg | MRE | AACCTAA | 1110 | 1124 | MYB binding site involved in light responsiveness |
| SiRAF27 | SETIT_017658mg | CCAAT-box | CAACGG | 521 | 533 | MYBHv1 binding site |
| SiRAF27 | SETIT_017658mg | A-box | CCGTCC | 212 | 224 | promoter and enhancer cis-acting regulatory elements |
| SiRAF27 | SETIT_017658mg | O2-site | GATGA(C/T)(A/G)TG(A/G) | 1266 | 1282 | Zein metabolism regulatory elements |
| SiRAF28 | SETIT_001833mg | ABRE | ACGTG | 361 | 371 | abscisic acid response element |
| SiRAF28 | SETIT_001833mg | ABRE | ACGTG | 823 | 833 | abscisic acid response element |
| SiRAF28 | SETIT_001833mg | ABRE | ACGTG | 865 | 875 | abscisic acid response element |
| SiRAF28 | SETIT_001833mg | ABRE | ACGTG | 1405 | 1415 | abscisic acid response element |
| SiRAF28 | SETIT_001833mg | ABRE | CACGTG | 1889 | 1901 | abscisic acid response element |
| SiRAF28 | SETIT_001833mg | ABRE | ACGTG | 1891 | 1901 | abscisic acid response element |
| SiRAF28 | SETIT_001833mg | ARE | AAACCA | 1885 | 1897 | anaerobic inducing element |
| SiRAF28 | SETIT_001833mg | CAAT-box | CAAAT | 9 | 19 | common cis-acting element |
| SiRAF28 | SETIT_001833mg | CAAT-box | CCAAT | 249 | 259 | common cis-acting element |
| SiRAF28 | SETIT_001833mg | CAAT-box | CCAAT | 288 | 298 | common cis-acting element |
| SiRAF28 | SETIT_001833mg | CAAT-box | CCCAATTT | 970 | 986 | common cis-acting element |
| SiRAF28 | SETIT_001833mg | CAAT-box | CCAAT | 974 | 984 | common cis-acting element |
| SiRAF28 | SETIT_001833mg | CAAT-box | CAAAT | 977 | 987 | common cis-acting element |
| SiRAF28 | SETIT_001833mg | CAAT-box | CCAAT | 1054 | 1064 | common cis-acting element |
| SiRAF28 | SETIT_001833mg | CAAT-box | CAAAT | 1201 | 1211 | common cis-acting element |
| SiRAF28 | SETIT_001833mg | TATA-box | ccTATAAAaa | 195 | 215 | core promoter element |
| SiRAF28 | SETIT_001833mg | TATA-box | TATAAAA | 199 | 213 | core promoter element |
| SiRAF28 | SETIT_001833mg | TATA-box | TATAAA | 201 | 213 | core promoter element |
| SiRAF28 | SETIT_001833mg | TATA-box | TATAA | 203 | 213 | core promoter element |
| SiRAF28 | SETIT_001833mg | TATA-box | TATA | 205 | 213 | core promoter element |
| SiRAF28 | SETIT_001833mg | TATA-box | TATA | 1052 | 1060 | core promoter element |
| SiRAF28 | SETIT_001833mg | TATA-box | ccTATAAAaa | 1150 | 1168 | core promoter element |
| SiRAF28 | SETIT_001833mg | TATA-box | TATAAAA | 1153 | 1167 | core promoter element |
| SiRAF28 | SETIT_001833mg | TATA-box | TATAAA | 1155 | 1167 | core promoter element |
| SiRAF28 | SETIT_001833mg | TATA-box | TATAA | 1157 | 1167 | core promoter element |
| SiRAF28 | SETIT_001833mg | TATA-box | TATA | 1159 | 1167 | core promoter element |
| SiRAF28 | SETIT_001833mg | TATA-box | TATA | 1329 | 1337 | core promoter element |
| SiRAF28 | SETIT_001833mg | GCN4_motif | TGAGTCA | 802 | 816 | endosperm expression regulatory element |
| SiRAF28 | SETIT_001833mg | P-box | CCTTTTG | 1009 | 1023 | gibberellin-responsive element |
| SiRAF28 | SETIT_001833mg | P-box | CCTTTTG | 1584 | 1598 | gibberellin-responsive element |
| SiRAF28 | SETIT_001833mg | GC-motif | CCCCCG | 641 | 653 | hypoxia-specifically induced elements |
| SiRAF28 | SETIT_001833mg | GC-motif | CCCCCG | 1740 | 1752 | hypoxia-specifically induced elements |
| SiRAF28 | SETIT_001833mg | GC-motif | CCCCCG | 1747 | 1759 | hypoxia-specifically induced elements |
| SiRAF28 | SETIT_001833mg | G-Box | CACGTT | 1403 | 1415 | light responsive element |
| SiRAF28 | SETIT_001833mg | G-Box | CACGTG | 1889 | 1901 | light responsive element |
| SiRAF28 | SETIT_001833mg | G-box | CACGTC | 360 | 372 | light responsive element |
| SiRAF28 | SETIT_001833mg | G-box | CACGAC | 587 | 599 | light responsive element |
| SiRAF28 | SETIT_001833mg | G-box | CACGTC | 822 | 834 | light responsive element |
| SiRAF28 | SETIT_001833mg | G-box | TACGTG | 863 | 875 | light responsive element |
| SiRAF28 | SETIT_001833mg | G-box | CACGTG | 1889 | 1901 | light responsive element |
| SiRAF28 | SETIT_001833mg | Sp1 | GGGCGG | 617 | 629 | light responsive element |
| SiRAF28 | SETIT_001833mg | Box 4 | ATTAAT | 1375 | 1387 | light responsive element |
| SiRAF28 | SETIT_001833mg | I-box | ccttatcct | 236 | 254 | light responsive element |
| SiRAF28 | SETIT_001833mg | TCT-motif | TCTTAC | 406 | 418 | light responsive element |
| SiRAF28 | SETIT_001833mg | GATA-motif | AAGGATAAGG | 236 | 254 | light responsive element |
| SiRAF28 | SETIT_001833mg | GATA-motif | AAGGATAAGG | 1384 | 1402 | light responsive element |
| SiRAF28 | SETIT_001833mg | GATA-motif | AAGATAAGATT | 1459 | 1479 | light responsive element |
| SiRAF28 | SETIT_001833mg | TCCC-motif | TCTCCCT | 1166 | 1180 | light responsive element |
| SiRAF28 | SETIT_001833mg | AE-box | AGAAACTT | 1092 | 1108 | light responsive element |
| SiRAF28 | SETIT_001833mg | ACA-motif | ATCGACAGCCATT | 1095 | 1119 | light responsive element |
| SiRAF28 | SETIT_001833mg | CGTCA-motif | CGTCA | 498 | 508 | MeJA response regulatory element |
| SiRAF28 | SETIT_001833mg | CGTCA-motif | CGTCA | 677 | 687 | MeJA response regulatory element |
| SiRAF28 | SETIT_001833mg | CGTCA-motif | CGTCA | 825 | 835 | MeJA response regulatory element |
| SiRAF28 | SETIT_001833mg | CGTCA-motif | CGTCA | 1607 | 1617 | MeJA response regulatory element |
| SiRAF28 | SETIT_001833mg | CGTCA-motif | CGTCA | 1676 | 1686 | MeJA response regulatory element |
| SiRAF28 | SETIT_001833mg | TGACG-motif | TGACG | 498 | 508 | MeJA response regulatory element |
| SiRAF28 | SETIT_001833mg | TGACG-motif | TGACG | 677 | 687 | MeJA response regulatory element |
| SiRAF28 | SETIT_001833mg | TGACG-motif | TGACG | 825 | 835 | MeJA response regulatory element |
| SiRAF28 | SETIT_001833mg | TGACG-motif | TGACG | 1607 | 1617 | MeJA response regulatory element |
| SiRAF28 | SETIT_001833mg | TGACG-motif | TGACG | 1676 | 1686 | MeJA response regulatory element |
| SiRAF28 | SETIT_001833mg | CAT-box | GCCACT | 1669 | 1681 | meristem expression regulatory element |
| SiRAF28 | SETIT_001833mg | MBS | CAACTG | 1723 | 1735 | MYB binding site involved in drought-inducibility |
| SiRAF28 | SETIT_001833mg | MRE | AACCTAA | 1480 | 1494 | MYB binding site involved in light responsiveness |
| SiRAF28 | SETIT_001833mg | A-box | CCGTCC | 638 | 650 | promoter and enhancer cis-acting regulatory elements |
| SiRAF28 | SETIT_001833mg | motif I | gGTACGTGGCG | 857 | 877 | root-specific regulatory elements |
| SiRAF29 | SETIT_001885mg | ABRE | ACGTG | 101 | 111 | abscisic acid response element |
| SiRAF29 | SETIT_001885mg | ABRE | ACGTG | 1845 | 1855 | abscisic acid response element |
| SiRAF29 | SETIT_001885mg | ARE | AAACCA | 440 | 452 | anaerobic inducing element |
| SiRAF29 | SETIT_001885mg | ARE | AAACCA | 814 | 826 | anaerobic inducing element |
| SiRAF29 | SETIT_001885mg | ARE | AAACCA | 1170 | 1182 | anaerobic inducing element |
| SiRAF29 | SETIT_001885mg | TGA-element | AACGAC | 1036 | 1048 | auxin-responsive element |
| SiRAF29 | SETIT_001885mg | CAAT-box | CCAAT | 24 | 34 | common cis-acting element |
| SiRAF29 | SETIT_001885mg | CAAT-box | CAAAT | 79 | 89 | common cis-acting element |
| SiRAF29 | SETIT_001885mg | CAAT-box | CCAAT | 387 | 397 | common cis-acting element |
| SiRAF29 | SETIT_001885mg | CAAT-box | CCAAT | 420 | 430 | common cis-acting element |
| SiRAF29 | SETIT_001885mg | CAAT-box | CAAAT | 445 | 455 | common cis-acting element |
| SiRAF29 | SETIT_001885mg | CAAT-box | CCAAT | 457 | 467 | common cis-acting element |
| SiRAF29 | SETIT_001885mg | CAAT-box | CAAAT | 508 | 518 | common cis-acting element |
| SiRAF29 | SETIT_001885mg | CAAT-box | CAAAT | 599 | 609 | common cis-acting element |
| SiRAF29 | SETIT_001885mg | CAAT-box | CAAAT | 641 | 651 | common cis-acting element |
| SiRAF29 | SETIT_001885mg | CAAT-box | CAAAT | 697 | 707 | common cis-acting element |
| SiRAF29 | SETIT_001885mg | CAAT-box | CCAAT | 863 | 873 | common cis-acting element |
| SiRAF29 | SETIT_001885mg | CAAT-box | CAAAT | 940 | 950 | common cis-acting element |
| SiRAF29 | SETIT_001885mg | CAAT-box | CAAAT | 975 | 985 | common cis-acting element |
| SiRAF29 | SETIT_001885mg | CAAT-box | CAAAT | 1032 | 1042 | common cis-acting element |
| SiRAF29 | SETIT_001885mg | CAAT-box | CAAAT | 1051 | 1061 | common cis-acting element |
| SiRAF29 | SETIT_001885mg | CAAT-box | CCAAT | 1216 | 1226 | common cis-acting element |
| SiRAF29 | SETIT_001885mg | CAAT-box | CAAAT | 1296 | 1306 | common cis-acting element |
| SiRAF29 | SETIT_001885mg | CAAT-box | CCAAT | 1779 | 1789 | common cis-acting element |
| SiRAF29 | SETIT_001885mg | TATA-box | ATTATA | 118 | 130 | core promoter element |
| SiRAF29 | SETIT_001885mg | TATA-box | TATAA | 120 | 130 | core promoter element |
| SiRAF29 | SETIT_001885mg | TATA-box | TATA | 122 | 130 | core promoter element |
| SiRAF29 | SETIT_001885mg | TATA-box | ccTATAAAaa | 416 | 434 | core promoter element |
| SiRAF29 | SETIT_001885mg | TATA-box | TACAAAA | 435 | 449 | core promoter element |
| SiRAF29 | SETIT_001885mg | TATA-box | TATATA | 530 | 542 | core promoter element |
| SiRAF29 | SETIT_001885mg | TATA-box | TATA | 534 | 542 | core promoter element |
| SiRAF29 | SETIT_001885mg | TATA-box | TATA | 560 | 568 | core promoter element |
| SiRAF29 | SETIT_001885mg | TATA-box | ATTATA | 603 | 615 | core promoter element |
| SiRAF29 | SETIT_001885mg | TATA-box | TATAA | 605 | 615 | core promoter element |
| SiRAF29 | SETIT_001885mg | TATA-box | TATA | 607 | 615 | core promoter element |
| SiRAF29 | SETIT_001885mg | TATA-box | ATTATA | 608 | 620 | core promoter element |
| SiRAF29 | SETIT_001885mg | TATA-box | TATAA | 610 | 620 | core promoter element |
| SiRAF29 | SETIT_001885mg | TATA-box | TATA | 612 | 620 | core promoter element |
| SiRAF29 | SETIT_001885mg | TATA-box | ATTATA | 613 | 625 | core promoter element |
| SiRAF29 | SETIT_001885mg | TATA-box | TATAA | 615 | 625 | core promoter element |
| SiRAF29 | SETIT_001885mg | TATA-box | TATA | 617 | 625 | core promoter element |
| SiRAF29 | SETIT_001885mg | TC-rich repeats | GTTTTCTTAC | 145 | 163 | defense and stress response elements |
| SiRAF29 | SETIT_001885mg | RY-element | CATGCATG | 1162 | 1178 | elements involved in seed-specific regulation |
| SiRAF29 | SETIT_001885mg | GC-motif | CCCCCG | 376 | 388 | hypoxia-specifically induced elements |
| SiRAF29 | SETIT_001885mg | G-box | TACGTG | 100 | 112 | light responsive element |
| SiRAF29 | SETIT_001885mg | G-box | CACGTC | 1843 | 1855 | light responsive element |
| SiRAF29 | SETIT_001885mg | GT1-motif | GGTTAA | 1290 | 1302 | light responsive element |
| SiRAF29 | SETIT_001885mg | Sp1 | GGGCGG | 1693 | 1705 | light responsive element |
| SiRAF29 | SETIT_001885mg | Box 4 | ATTAAT | 191 | 203 | light responsive element |
| SiRAF29 | SETIT_001885mg | TCT-motif | TCTTAC | 1207 | 1219 | light responsive element |
| SiRAF29 | SETIT_001885mg | GATA-motif | GATAGGA | 681 | 695 | light responsive element |
| SiRAF29 | SETIT_001885mg | Box II | TGGTAATAA | 228 | 246 | light responsive element |
| SiRAF29 | SETIT_001885mg | GTGGC-motif | CAGCGTGTGGC | 284 | 304 | light responsive element |
| SiRAF29 | SETIT_001885mg | chs-CMA2a | TCACTTGA | 1236 | 1252 | light responsive element |
| SiRAF29 | SETIT_001885mg | LTR | CCGAAA | 1665 | 1677 | low temperature response element |
| SiRAF29 | SETIT_001885mg | LTR | CCGAAA | 1769 | 1781 | low temperature response element |
| SiRAF29 | SETIT_001885mg | CGTCA-motif | CGTCA | 266 | 276 | MeJA response regulatory element |
| SiRAF29 | SETIT_001885mg | TGACG-motif | TGACG | 266 | 276 | MeJA response regulatory element |
| SiRAF29 | SETIT_001885mg | CAT-box | GCCACT | 254 | 266 | meristem expression regulatory element |
| SiRAF29 | SETIT_001885mg | CAT-box | GCCACT | 502 | 514 | meristem expression regulatory element |
| SiRAF29 | SETIT_001885mg | CAT-box | GCCACT | 1583 | 1595 | meristem expression regulatory element |
| SiRAF29 | SETIT_001885mg | TCA-element | CCATCTTTTT | 140 | 158 | salicylic acid response element |
| SiRAF29 | SETIT_001885mg | O2-site | GATGACATGG | 276 | 294 | Zein metabolism regulatory elements |
| SiRAF29 | SETIT_001885mg | O2-site | GATGACATGG | 1275 | 1293 | Zein metabolism regulatory elements |
| SiRAF30 | SETIT_008355mg | ABRE | ACGTG | 139 | 149 | abscisic acid response element |
| SiRAF30 | SETIT_008355mg | circadian | CAAAGATATC | 501 | 519 | circadian rhythm control element |
| SiRAF30 | SETIT_008355mg | circadian | CAAAGATATC | 711 | 729 | circadian rhythm control element |
| SiRAF30 | SETIT_008355mg | CAAT-box | CCAAT | 317 | 327 | common cis-acting element |
| SiRAF30 | SETIT_008355mg | CAAT-box | CCAAT | 389 | 399 | common cis-acting element |
| SiRAF30 | SETIT_008355mg | CAAT-box | CCCAATTT | 449 | 465 | common cis-acting element |
| SiRAF30 | SETIT_008355mg | CAAT-box | CCAAT | 453 | 463 | common cis-acting element |
| SiRAF30 | SETIT_008355mg | CAAT-box | CAAAT | 456 | 466 | common cis-acting element |
| SiRAF30 | SETIT_008355mg | CAAT-box | CAAAT | 998 | 1008 | common cis-acting element |
| SiRAF30 | SETIT_008355mg | CAAT-box | CCAAT | 1104 | 1114 | common cis-acting element |
| SiRAF30 | SETIT_008355mg | CAAT-box | CAAAT | 1983 | 1993 | common cis-acting element |
| SiRAF30 | SETIT_008355mg | TATA-box | TACAAAA | 334 | 348 | core promoter element |
| SiRAF30 | SETIT_008355mg | TATA-box | TATAAAA | 368 | 382 | core promoter element |
| SiRAF30 | SETIT_008355mg | TATA-box | TATAAA | 370 | 382 | core promoter element |
| SiRAF30 | SETIT_008355mg | TATA-box | TATATAA | 370 | 384 | core promoter element |
| SiRAF30 | SETIT_008355mg | TATA-box | TATATA | 372 | 384 | core promoter element |
| SiRAF30 | SETIT_008355mg | TATA-box | ATATAA | 373 | 385 | core promoter element |
| SiRAF30 | SETIT_008355mg | TATA-box | TATA | 376 | 384 | core promoter element |
| SiRAF30 | SETIT_008355mg | TATA-box | TATA | 1361 | 1369 | core promoter element |
| SiRAF30 | SETIT_008355mg | GC-motif | CCCCCG | 1420 | 1432 | hypoxia-specifically induced elements |
| SiRAF30 | SETIT_008355mg | GC-motif | CCCCCG | 1453 | 1465 | hypoxia-specifically induced elements |
| SiRAF30 | SETIT_008355mg | G-box | TACGTG | 137 | 149 | light responsive element |
| SiRAF30 | SETIT_008355mg | G-box | CACGAC | 1585 | 1597 | light responsive element |
| SiRAF30 | SETIT_008355mg | C-box | ACGAGCACCGCC | 1783 | 1805 | light responsive element |
| SiRAF30 | SETIT_008355mg | I-box | cGATAAGGCG | 1955 | 1973 | light responsive element |
| SiRAF30 | SETIT_008355mg | GATA-motif | GATAGGG | 1065 | 1079 | light responsive element |
| SiRAF30 | SETIT_008355mg | TCCC-motif | TCTCCCT | 695 | 709 | light responsive element |
| SiRAF30 | SETIT_008355mg | TGACG-motif | TGACG | 513 | 523 | MeJA response regulatory element |
| SiRAF30 | SETIT_008355mg | TGACG-motif | TGACG | 1610 | 1620 | MeJA response regulatory element |
| SiRAF30 | SETIT_008355mg | TGACG-motif | TGACG | 1760 | 1770 | MeJA response regulatory element |
| SiRAF30 | SETIT_008355mg | CGTCA-motif | CGTCA | 513 | 523 | MeJA response regulatory element |
| SiRAF30 | SETIT_008355mg | CGTCA-motif | CGTCA | 1610 | 1620 | MeJA response regulatory element |
| SiRAF30 | SETIT_008355mg | CGTCA-motif | CGTCA | 1760 | 1770 | MeJA response regulatory element |
| SiRAF30 | SETIT_008355mg | CAT-box | GCCACT | 183 | 195 | meristem expression regulatory element |
| SiRAF30 | SETIT_008355mg | CCAAT-box | CAACGG | 737 | 749 | MYBHv1 binding site |
| SiRAF30 | SETIT_008355mg | CCAAT-box | CAACGG | 1145 | 1157 | MYBHv1 binding site |
| SiRAF30 | SETIT_008355mg | TCA-element | TCAGAAGAGG | 840 | 858 | salicylic acid response element |
| SiRAF30 | SETIT_008355mg | O2-site | GATGACATGG | 1546 | 1566 | Zein metabolism regulatory elements |
| SiRAF31 | SETIT_029825mg | ABRE | CACGTG | 39 | 51 | abscisic acid response element |
| SiRAF31 | SETIT_029825mg | ABRE | ACGTG | 41 | 51 | abscisic acid response element |
| SiRAF31 | SETIT_029825mg | ABRE | ACGTG | 413 | 423 | abscisic acid response element |
| SiRAF31 | SETIT_029825mg | ABRE | ACGTG | 543 | 553 | abscisic acid response element |
| SiRAF31 | SETIT_029825mg | ABRE | ACGTG | 677 | 687 | abscisic acid response element |
| SiRAF31 | SETIT_029825mg | ABRE | CGTACGTGCA | 1378 | 1396 | abscisic acid response element |
| SiRAF31 | SETIT_029825mg | TGA-element | AACGAC | 1595 | 1607 | auxin-responsive element |
| SiRAF31 | SETIT_029825mg | circadian | CAAAGATATC | 755 | 773 | circadian rhythm control element |
| SiRAF31 | SETIT_029825mg | CAAT-box | CAAAT | 73 | 83 | common cis-acting element |
| SiRAF31 | SETIT_029825mg | CAAT-box | CAAAT | 304 | 314 | common cis-acting element |
| SiRAF31 | SETIT_029825mg | CAAT-box | CAAAT | 507 | 517 | common cis-acting element |
| SiRAF31 | SETIT_029825mg | CAAT-box | CCAAT | 594 | 604 | common cis-acting element |
| SiRAF31 | SETIT_029825mg | CAAT-box | CCAAT | 619 | 629 | common cis-acting element |
| SiRAF31 | SETIT_029825mg | CAAT-box | CCAAT | 980 | 990 | common cis-acting element |
| SiRAF31 | SETIT_029825mg | CAAT-box | CCAAT | 1270 | 1280 | common cis-acting element |
| SiRAF31 | SETIT_029825mg | CAAT-box | CCAAT | 1568 | 1578 | common cis-acting element |
| SiRAF31 | SETIT_029825mg | TATA-box | TACAAAA | 203 | 217 | core promoter element |
| SiRAF31 | SETIT_029825mg | TATA-box | TATA | 261 | 269 | core promoter element |
| SiRAF31 | SETIT_029825mg | TATA-box | ATATAT | 554 | 566 | core promoter element |
| SiRAF31 | SETIT_029825mg | TATA-box | TATATA | 555 | 567 | core promoter element |
| SiRAF31 | SETIT_029825mg | TATA-box | ATATAA | 556 | 568 | core promoter element |
| SiRAF31 | SETIT_029825mg | TATA-box | TATA | 559 | 567 | core promoter element |
| SiRAF31 | SETIT_029825mg | TATA-box | ccTATAAAaa | 751 | 769 | core promoter element |
| SiRAF31 | SETIT_029825mg | TATA-box | TACAAAA | 755 | 769 | core promoter element |
| SiRAF31 | SETIT_029825mg | RY-element | CATGCATG | 165 | 181 | elements involved in seed-specific regulation |
| SiRAF31 | SETIT_029825mg | GC-motif | CCCCCG | 1853 | 1865 | hypoxia-specifically induced elements |
| SiRAF31 | SETIT_029825mg | ACE | GCGACGTACC | 475 | 493 | light responsive element |
| SiRAF31 | SETIT_029825mg | G-Box | CACGTG | 39 | 51 | light responsive element |
| SiRAF31 | SETIT_029825mg | G-box | CACGTG | 39 | 51 | light responsive element |
| SiRAF31 | SETIT_029825mg | G-box | CACGTC | 412 | 424 | light responsive element |
| SiRAF31 | SETIT_029825mg | G-box | CACGTC | 541 | 553 | light responsive element |
| SiRAF31 | SETIT_029825mg | G-box | CACGTC | 676 | 688 | light responsive element |
| SiRAF31 | SETIT_029825mg | GT1-motif | GGTTAA | 1618 | 1630 | light responsive element |
| SiRAF31 | SETIT_029825mg | GT1-motif | GGTTAA | 1626 | 1638 | light responsive element |
| SiRAF31 | SETIT_029825mg | Sp1 | GGGCGG | 1915 | 1927 | light responsive element |
| SiRAF31 | SETIT_029825mg | Sp1 | GGGCGG | 1976 | 1988 | light responsive element |
| SiRAF31 | SETIT_029825mg | chs-Unit 1 m1 | ACCTAACCCGC | 1635 | 1655 | light responsive element |
| SiRAF31 | SETIT_029825mg | TCCC-motif | TCTCCCT | 1829 | 1843 | light responsive element |
| SiRAF31 | SETIT_029825mg | I-box | ccttatcct | 645 | 663 | light responsive element |
| SiRAF31 | SETIT_029825mg | GATA-motif | AAGGATAAGG | 644 | 662 | light responsive element |
| SiRAF31 | SETIT_029825mg | AE-box | AGAAACTT | 881 | 897 | light responsive element |
| SiRAF31 | SETIT_029825mg | LTR | CCGAAA | 1407 | 1419 | low temperature response element |
| SiRAF31 | SETIT_029825mg | CGTCA-motif | CGTCA | 163 | 173 | MeJA response regulatory element |
| SiRAF31 | SETIT_029825mg | CGTCA-motif | CGTCA | 679 | 689 | MeJA response regulatory element |
| SiRAF31 | SETIT_029825mg | CGTCA-motif | CGTCA | 771 | 781 | MeJA response regulatory element |
| SiRAF31 | SETIT_029825mg | TGACG-motif | TGACG | 163 | 173 | MeJA response regulatory element |
| SiRAF31 | SETIT_029825mg | TGACG-motif | TGACG | 679 | 689 | MeJA response regulatory element |
| SiRAF31 | SETIT_029825mg | TGACG-motif | TGACG | 771 | 781 | MeJA response regulatory element |
| SiRAF31 | SETIT_029825mg | MBS | CAACTG | 788 | 800 | MYB binding site involved in drought-inducibility |
| SiRAF31 | SETIT_029825mg | CCAAT-box | CAACGG | 1467 | 1479 | MYBHv1 binding site |
| SiRAF31 | SETIT_029825mg | A-box | CCGTCC | 775 | 787 | promoter and enhancer cis-acting regulatory elements |
| SiRAF32 | SETIT_013509mg | ABRE | CGTACGTGCA | 1146 | 1164 | abscisic acid response element |
| SiRAF32 | SETIT_013509mg | ABRE | ACGTG | 1372 | 1382 | abscisic acid response element |
| SiRAF32 | SETIT_013509mg | ARE | AAACCA | 475 | 487 | anaerobic inducing element |
| SiRAF32 | SETIT_013509mg | ARE | AAACCA | 701 | 713 | anaerobic inducing element |
| SiRAF32 | SETIT_013509mg | ARE | AAACCA | 1114 | 1126 | anaerobic inducing element |
| SiRAF32 | SETIT_013509mg | CAAT-box | CAAAT | 0 | 10 | common cis-acting element |
| SiRAF32 | SETIT_013509mg | CAAT-box | CAAAT | 50 | 60 | common cis-acting element |
| SiRAF32 | SETIT_013509mg | CAAT-box | CAAAT | 188 | 198 | common cis-acting element |
| SiRAF32 | SETIT_013509mg | CAAT-box | CAAAT | 433 | 443 | common cis-acting element |
| SiRAF32 | SETIT_013509mg | CAAT-box | CCAAT | 538 | 548 | common cis-acting element |
| SiRAF32 | SETIT_013509mg | CAAT-box | CAAAT | 661 | 671 | common cis-acting element |
| SiRAF32 | SETIT_013509mg | CAAT-box | CCAAT | 700 | 710 | common cis-acting element |
| SiRAF32 | SETIT_013509mg | CAAT-box | CAAAT | 750 | 760 | common cis-acting element |
| SiRAF32 | SETIT_013509mg | CAAT-box | CAAAT | 778 | 788 | common cis-acting element |
| SiRAF32 | SETIT_013509mg | CAAT-box | CAAAT | 804 | 814 | common cis-acting element |
| SiRAF32 | SETIT_013509mg | CAAT-box | CAAAT | 949 | 959 | common cis-acting element |
| SiRAF32 | SETIT_013509mg | CAAT-box | CAAAT | 1002 | 1012 | common cis-acting element |
| SiRAF32 | SETIT_013509mg | CAAT-box | CAAAT | 1103 | 1113 | common cis-acting element |
| SiRAF32 | SETIT_013509mg | CAAT-box | CAAAT | 1145 | 1155 | common cis-acting element |
| SiRAF32 | SETIT_013509mg | CAAT-box | CAAAT | 1190 | 1200 | common cis-acting element |
| SiRAF32 | SETIT_013509mg | CAAT-box | CAAAT | 1414 | 1424 | common cis-acting element |
| SiRAF32 | SETIT_013509mg | CAAT-box | CAAAT | 1597 | 1607 | common cis-acting element |
| SiRAF32 | SETIT_013509mg | CAAT-box | CCAAT | 1620 | 1630 | common cis-acting element |
| SiRAF32 | SETIT_013509mg | CAAT-box | CAAAT | 1686 | 1696 | common cis-acting element |
| SiRAF32 | SETIT_013509mg | CAAT-box | CCAAT | 1859 | 1869 | common cis-acting element |
| SiRAF32 | SETIT_013509mg | TATA-box | TATAA | -4 | 6 | core promoter element |
| SiRAF32 | SETIT_013509mg | TATA-box | TATA | -2 | 6 | core promoter element |
| SiRAF32 | SETIT_013509mg | TATA-box | TATAAA | 13 | 25 | core promoter element |
| SiRAF32 | SETIT_013509mg | TATA-box | TATAA | 15 | 25 | core promoter element |
| SiRAF32 | SETIT_013509mg | TATA-box | TATA | 17 | 25 | core promoter element |
| SiRAF32 | SETIT_013509mg | TATA-box | TATAA | 27 | 37 | core promoter element |
| SiRAF32 | SETIT_013509mg | TATA-box | TATA | 29 | 37 | core promoter element |
| SiRAF32 | SETIT_013509mg | TATA-box | ATATAT | 128 | 140 | core promoter element |
| SiRAF32 | SETIT_013509mg | TATA-box | TATA | 131 | 139 | core promoter element |
| SiRAF32 | SETIT_013509mg | TATA-box | TATATA | 526 | 538 | core promoter element |
| SiRAF32 | SETIT_013509mg | TATA-box | ATATAT | 527 | 539 | core promoter element |
| SiRAF32 | SETIT_013509mg | TATA-box | TATA | 530 | 538 | core promoter element |
| SiRAF32 | SETIT_013509mg | TATA-box | ATATAT | 550 | 562 | core promoter element |
| SiRAF32 | SETIT_013509mg | TATA-box | TATA | 553 | 561 | core promoter element |
| SiRAF32 | SETIT_013509mg | TATA-box | ATTATA | 602 | 614 | core promoter element |
| SiRAF32 | SETIT_013509mg | TATA-box | TATAA | 604 | 614 | core promoter element |
| SiRAF32 | SETIT_013509mg | TATA-box | TATA | 606 | 614 | core promoter element |
| SiRAF32 | SETIT_013509mg | TATA-box | ccTATAAAaa | 935 | 953 | core promoter element |
| SiRAF32 | SETIT_013509mg | TATA-box | TATAAAA | 938 | 952 | core promoter element |
| SiRAF32 | SETIT_013509mg | TATA-box | TATAAA | 940 | 952 | core promoter element |
| SiRAF32 | SETIT_013509mg | TATA-box | TATAA | 942 | 952 | core promoter element |
| SiRAF32 | SETIT_013509mg | TATA-box | TATA | 944 | 952 | core promoter element |
| SiRAF32 | SETIT_013509mg | TATA-box | ATTATA | 972 | 984 | core promoter element |
| SiRAF32 | SETIT_013509mg | TATA-box | TATAA | 974 | 984 | core promoter element |
| SiRAF32 | SETIT_013509mg | TATA-box | TATA | 976 | 984 | core promoter element |
| SiRAF32 | SETIT_013509mg | TATA-box | TATAA | 1331 | 1341 | core promoter element |
| SiRAF32 | SETIT_013509mg | TATA-box | TATA | 1333 | 1341 | core promoter element |
| SiRAF32 | SETIT_013509mg | TATA-box | TATA | 1474 | 1482 | core promoter element |
| SiRAF32 | SETIT_013509mg | TATA-box | ATTATA | 1475 | 1487 | core promoter element |
| SiRAF32 | SETIT_013509mg | TATA-box | TATAA | 1477 | 1487 | core promoter element |
| SiRAF32 | SETIT_013509mg | TATA-box | TATA | 1479 | 1487 | core promoter element |
| SiRAF32 | SETIT_013509mg | TATA-box | TATATA | 1711 | 1723 | core promoter element |
| SiRAF32 | SETIT_013509mg | TATA-box | ATATAT | 1712 | 1724 | core promoter element |
| SiRAF32 | SETIT_013509mg | TATA-box | TATA | 1715 | 1723 | core promoter element |
| SiRAF32 | SETIT_013509mg | TATA-box | TATA | 1903 | 1911 | core promoter element |
| SiRAF32 | SETIT_013509mg | TC-rich repeats | ATTCTCTAAC | 1701 | 1719 | defense and stress response elements |
| SiRAF32 | SETIT_013509mg | GCN4_motif | TGAGTCA | 1358 | 1372 | endosperm expression regulatory element |
| SiRAF32 | SETIT_013509mg | TATC-box | TATCCCA | 1399 | 1413 | gibberellin response element |
| SiRAF32 | SETIT_013509mg | P-box | CCTTTTG | 167 | 181 | gibberellin-responsive element |
| SiRAF32 | SETIT_013509mg | P-box | CCTTTTG | 501 | 515 | gibberellin-responsive element |
| SiRAF32 | SETIT_013509mg | ACE | CTAACGTATT | 207 | 225 | light responsive element |
| SiRAF32 | SETIT_013509mg | ACE | CTAACGTATT | 1227 | 1245 | light responsive element |
| SiRAF32 | SETIT_013509mg | G-box | CACGTC | 1371 | 1383 | light responsive element |
| SiRAF32 | SETIT_013509mg | GT1-motif | GGTTAA | 872 | 884 | light responsive element |
| SiRAF32 | SETIT_013509mg | GT1-motif | GGTTAA | 1736 | 1748 | light responsive element |
| SiRAF32 | SETIT_013509mg | Box 4 | ATTAAT | 1666 | 1678 | light responsive element |
| SiRAF32 | SETIT_013509mg | GATA-motif | AAGGATAAGG | 240 | 258 | light responsive element |
| SiRAF32 | SETIT_013509mg | I-box | TGATAATGT | 640 | 658 | light responsive element |
| SiRAF32 | SETIT_013509mg | TCT-motif | TCTTAC | 121 | 133 | light responsive element |
| SiRAF32 | SETIT_013509mg | TCT-motif | TCTTAC | 215 | 227 | light responsive element |
| SiRAF32 | SETIT_013509mg | AE-box | AGAAACAA | 786 | 802 | light responsive element |
| SiRAF32 | SETIT_013509mg | TGACG-motif | TGACG | 191 | 201 | MeJA response regulatory element |
| SiRAF32 | SETIT_013509mg | TGACG-motif | TGACG | 320 | 330 | MeJA response regulatory element |
| SiRAF32 | SETIT_013509mg | TGACG-motif | TGACG | 1174 | 1184 | MeJA response regulatory element |
| SiRAF32 | SETIT_013509mg | TGACG-motif | TGACG | 1242 | 1252 | MeJA response regulatory element |
| SiRAF32 | SETIT_013509mg | CGTCA-motif | CGTCA | 191 | 201 | MeJA response regulatory element |
| SiRAF32 | SETIT_013509mg | CGTCA-motif | CGTCA | 320 | 330 | MeJA response regulatory element |
| SiRAF32 | SETIT_013509mg | CGTCA-motif | CGTCA | 1174 | 1184 | MeJA response regulatory element |
| SiRAF32 | SETIT_013509mg | CGTCA-motif | CGTCA | 1242 | 1252 | MeJA response regulatory element |
| SiRAF32 | SETIT_013509mg | CAT-box | GCCACT | 425 | 437 | meristem expression regulatory element |
| SiRAF32 | SETIT_013509mg | TCA-element | CCATCTTTTT | 1027 | 1045 | salicylic acid response element |
| SiRAF32 | SETIT_013509mg | O2-site | GATGATGTGG | 485 | 503 | Zein metabolism regulatory elements |
| SiRAF33 | SETIT_035970mg | ABRE | ACGTG | 103 | 113 | abscisic acid response element |
| SiRAF33 | SETIT_035970mg | ABRE | ACGTG | 779 | 789 | abscisic acid response element |
| SiRAF33 | SETIT_035970mg | ABRE | ACGTG | 1524 | 1534 | abscisic acid response element |
| SiRAF33 | SETIT_035970mg | AuxRR-core | GGTCCAT | 1985 | 1999 | auxin response element |
| SiRAF33 | SETIT_035970mg | TGA-element | AACGAC | 1452 | 1464 | auxin-responsive element |
| SiRAF33 | SETIT_035970mg | Unnamed_1 | GGATTTTACAGT | 1586 | 1608 | cis-acting element down-regulated expression of phytochrome |
| SiRAF33 | SETIT_035970mg | CAAT-box | CAAAT | 277 | 287 | common cis-acting element |
| SiRAF33 | SETIT_035970mg | CAAT-box | CCAAT | 501 | 511 | common cis-acting element |
| SiRAF33 | SETIT_035970mg | CAAT-box | CCAAT | 556 | 566 | common cis-acting element |
| SiRAF33 | SETIT_035970mg | CAAT-box | CCAAT | 644 | 654 | common cis-acting element |
| SiRAF33 | SETIT_035970mg | CAAT-box | CAAAT | 1114 | 1124 | common cis-acting element |
| SiRAF33 | SETIT_035970mg | CAAT-box | CCAAT | 1192 | 1202 | common cis-acting element |
| SiRAF33 | SETIT_035970mg | CAAT-box | CAAAT | 1260 | 1270 | common cis-acting element |
| SiRAF33 | SETIT_035970mg | CAAT-box | CCAAT | 1426 | 1436 | common cis-acting element |
| SiRAF33 | SETIT_035970mg | CAAT-box | CAAAT | 1653 | 1663 | common cis-acting element |
| SiRAF33 | SETIT_035970mg | CAAT-box | CAAAT | 1810 | 1820 | common cis-acting element |
| SiRAF33 | SETIT_035970mg | CAAT-box | CAAAT | 1828 | 1838 | common cis-acting element |
| SiRAF33 | SETIT_035970mg | CAAT-box | CAAAT | 1844 | 1854 | common cis-acting element |
| SiRAF33 | SETIT_035970mg | CAAT-box | CAAAT | 1872 | 1882 | common cis-acting element |
| SiRAF33 | SETIT_035970mg | TATA-box | TATA | 340 | 348 | core promoter element |
| SiRAF33 | SETIT_035970mg | TATA-box | TATA | 364 | 372 | core promoter element |
| SiRAF33 | SETIT_035970mg | TATA-box | TATA | 929 | 937 | core promoter element |
| SiRAF33 | SETIT_035970mg | TATA-box | TATAAA | 943 | 955 | core promoter element |
| SiRAF33 | SETIT_035970mg | TATA-box | TATAA | 945 | 955 | core promoter element |
| SiRAF33 | SETIT_035970mg | TATA-box | TATA | 947 | 955 | core promoter element |
| SiRAF33 | SETIT_035970mg | TATA-box | TATACA | 990 | 1002 | core promoter element |
| SiRAF33 | SETIT_035970mg | TATA-box | TATATA | 992 | 1004 | core promoter element |
| SiRAF33 | SETIT_035970mg | TATA-box | TATA | 996 | 1004 | core promoter element |
| SiRAF33 | SETIT_035970mg | TATA-box | TACAAAA | 1029 | 1043 | core promoter element |
| SiRAF33 | SETIT_035970mg | TATA-box | TATAAATA | 1095 | 1111 | core promoter element |
| SiRAF33 | SETIT_035970mg | TATA-box | TATAAAT | 1097 | 1111 | core promoter element |
| SiRAF33 | SETIT_035970mg | TATA-box | TATAAA | 1099 | 1111 | core promoter element |
| SiRAF33 | SETIT_035970mg | TATA-box | TATAA | 1101 | 1111 | core promoter element |
| SiRAF33 | SETIT_035970mg | TATA-box | TATA | 1103 | 1111 | core promoter element |
| SiRAF33 | SETIT_035970mg | TATA-box | TATA | 1169 | 1177 | core promoter element |
| SiRAF33 | SETIT_035970mg | TATA-box | ATATAA | 1249 | 1261 | core promoter element |
| SiRAF33 | SETIT_035970mg | TATA-box | TATA | 1252 | 1260 | core promoter element |
| SiRAF33 | SETIT_035970mg | TATA-box | TATAAAT | 1386 | 1400 | core promoter element |
| SiRAF33 | SETIT_035970mg | TATA-box | TATAAA | 1388 | 1400 | core promoter element |
| SiRAF33 | SETIT_035970mg | TATA-box | TATAA | 1390 | 1400 | core promoter element |
| SiRAF33 | SETIT_035970mg | TATA-box | TATA | 1392 | 1400 | core promoter element |
| SiRAF33 | SETIT_035970mg | TATA-box | TATAA | 1683 | 1693 | core promoter element |
| SiRAF33 | SETIT_035970mg | TATA-box | TATA | 1685 | 1693 | core promoter element |
| SiRAF33 | SETIT_035970mg | TC-rich repeats | ATTCTCTAAC | 871 | 889 | defense and stress response elements |
| SiRAF33 | SETIT_035970mg | GARE-motif | TCTGTTG | 1491 | 1505 | gibberellin-responsive element |
| SiRAF33 | SETIT_035970mg | P-box | CCTTTTG | 1713 | 1727 | gibberellin-responsive element |
| SiRAF33 | SETIT_035970mg | GC-motif | CCCCCG | 198 | 210 | hypoxia-specifically induced elements |
| SiRAF33 | SETIT_035970mg | G-box | TACGTG | 101 | 113 | light responsive element |
| SiRAF33 | SETIT_035970mg | G-box | CACGTC | 1523 | 1535 | light responsive element |
| SiRAF33 | SETIT_035970mg | G-Box | CACGTT | 777 | 789 | light responsive element |
| SiRAF33 | SETIT_035970mg | Sp1 | GGGCGG | 171 | 183 | light responsive element |
| SiRAF33 | SETIT_035970mg | GT1-motif | GGTTAA | 1726 | 1738 | light responsive element |
| SiRAF33 | SETIT_035970mg | GA-motif | ATAGATAA | 540 | 556 | light responsive element |
| SiRAF33 | SETIT_035970mg | TCCC-motif | TCTCCCT | 149 | 163 | light responsive element |
| SiRAF33 | SETIT_035970mg | TCCC-motif | TCTCCCT | 244 | 258 | light responsive element |
| SiRAF33 | SETIT_035970mg | TCT-motif | TCTTAC | 891 | 903 | light responsive element |
| SiRAF33 | SETIT_035970mg | GATA-motif | AAGGATAAGG | 1638 | 1656 | light responsive element |
| SiRAF33 | SETIT_035970mg | GATA-motif | GATAGGG | 1962 | 1976 | light responsive element |
| SiRAF33 | SETIT_035970mg | TGACG-motif | TGACG | 134 | 144 | MeJA response regulatory element |
| SiRAF33 | SETIT_035970mg | TGACG-motif | TGACG | 1111 | 1121 | MeJA response regulatory element |
| SiRAF33 | SETIT_035970mg | TGACG-motif | TGACG | 1526 | 1536 | MeJA response regulatory element |
| SiRAF33 | SETIT_035970mg | TGACG-motif | TGACG | 1958 | 1968 | MeJA response regulatory element |
| SiRAF33 | SETIT_035970mg | CGTCA-motif | CGTCA | 134 | 144 | MeJA response regulatory element |
| SiRAF33 | SETIT_035970mg | CGTCA-motif | CGTCA | 1111 | 1121 | MeJA response regulatory element |
| SiRAF33 | SETIT_035970mg | CGTCA-motif | CGTCA | 1526 | 1536 | MeJA response regulatory element |
| SiRAF33 | SETIT_035970mg | CGTCA-motif | CGTCA | 1958 | 1968 | MeJA response regulatory element |
| SiRAF33 | SETIT_035970mg | CAT-box | GCCACT | 1104 | 1116 | meristem expression regulatory element |
| SiRAF33 | SETIT_035970mg | CCAAT-box | CAACGG | 1587 | 1599 | MYBHv1 binding site |
| SiRAF33 | SETIT_035970mg | A-box | CCGTCC | 1930 | 1942 | promoter and enhancer cis-acting regulatory elements |
| SiRAF33 | SETIT_035970mg | Box III | atCATTTTCACt | 261 | 283 | protein binding site |
| SiRAF33 | SETIT_035970mg | O2-site | GATGATGTGG | 46 | 64 | Zein metabolism regulatory elements |
| SiRAF33 | SETIT_035970mg | O2-site | GATGA(C/T)(A/G)TG(A/G) | 1520 | 1536 | Zein metabolism regulatory elements |
| SiRAF34 | SETIT_027523mg | ABRE | ACGTG | 581 | 591 | abscisic acid response element |
| SiRAF34 | SETIT_027523mg | ABRE | ACGTG | 647 | 657 | abscisic acid response element |
| SiRAF34 | SETIT_027523mg | ARE | AAACCA | 1524 | 1536 | anaerobic inducing element |
| SiRAF34 | SETIT_027523mg | TGA-element | AACGAC | 649 | 661 | auxin-responsive element |
| SiRAF34 | SETIT_027523mg | CAAT-box | CAAAT | 275 | 285 | common cis-acting element |
| SiRAF34 | SETIT_027523mg | CAAT-box | CAAAT | 303 | 313 | common cis-acting element |
| SiRAF34 | SETIT_027523mg | CAAT-box | TGCCAAC | 509 | 523 | common cis-acting element |
| SiRAF34 | SETIT_027523mg | CAAT-box | CAAAT | 735 | 745 | common cis-acting element |
| SiRAF34 | SETIT_027523mg | CAAT-box | CAAAT | 783 | 793 | common cis-acting element |
| SiRAF34 | SETIT_027523mg | CAAT-box | CAAAT | 788 | 798 | common cis-acting element |
| SiRAF34 | SETIT_027523mg | CAAT-box | CAAAT | 806 | 816 | common cis-acting element |
| SiRAF34 | SETIT_027523mg | CAAT-box | CAAAT | 924 | 934 | common cis-acting element |
| SiRAF34 | SETIT_027523mg | CAAT-box | CAAAT | 1146 | 1156 | common cis-acting element |
| SiRAF34 | SETIT_027523mg | CAAT-box | CAAAT | 1263 | 1273 | common cis-acting element |
| SiRAF34 | SETIT_027523mg | CAAT-box | CCAAT | 1443 | 1453 | common cis-acting element |
| SiRAF34 | SETIT_027523mg | CAAT-box | CAAAT | 1534 | 1544 | common cis-acting element |
| SiRAF34 | SETIT_027523mg | CAAT-box | CCAAT | 1549 | 1559 | common cis-acting element |
| SiRAF34 | SETIT_027523mg | CAAT-box | CAAAT | 1555 | 1565 | common cis-acting element |
| SiRAF34 | SETIT_027523mg | CAAT-box | CAAAT | 1784 | 1794 | common cis-acting element |
| SiRAF34 | SETIT_027523mg | CAAT-box | CAAAT | 1787 | 1797 | common cis-acting element |
| SiRAF34 | SETIT_027523mg | CAAT-box | CAAAT | 1965 | 1975 | common cis-acting element |
| SiRAF34 | SETIT_027523mg | TATA-box | ATTATA | 1213 | 1225 | core promoter element |
| SiRAF34 | SETIT_027523mg | TATA-box | TATAA | 1215 | 1225 | core promoter element |
| SiRAF34 | SETIT_027523mg | TATA-box | TATA | 1217 | 1225 | core promoter element |
| SiRAF34 | SETIT_027523mg | TATA-box | TATAAAA | 1218 | 1232 | core promoter element |
| SiRAF34 | SETIT_027523mg | TATA-box | TATAAA | 1220 | 1232 | core promoter element |
| SiRAF34 | SETIT_027523mg | TATA-box | TATAA | 1222 | 1232 | core promoter element |
| SiRAF34 | SETIT_027523mg | TATA-box | TATA | 1224 | 1232 | core promoter element |
| SiRAF34 | SETIT_027523mg | TATA-box | TATA | 1452 | 1460 | core promoter element |
| SiRAF34 | SETIT_027523mg | TATA-box | ATATAT | 1468 | 1480 | core promoter element |
| SiRAF34 | SETIT_027523mg | TATA-box | TATATA | 1469 | 1481 | core promoter element |
| SiRAF34 | SETIT_027523mg | TATA-box | ATATAA | 1470 | 1482 | core promoter element |
| SiRAF34 | SETIT_027523mg | TATA-box | TATA | 1473 | 1481 | core promoter element |
| SiRAF34 | SETIT_027523mg | TATA-box | TATAA | 1892 | 1902 | core promoter element |
| SiRAF34 | SETIT_027523mg | TATA-box | TATA | 1894 | 1902 | core promoter element |
| SiRAF34 | SETIT_027523mg | P-box | CCTTTTG | 1814 | 1828 | gibberellin-responsive element |
| SiRAF34 | SETIT_027523mg | G-box | TACGTG | 580 | 592 | light responsive element |
| SiRAF34 | SETIT_027523mg | G-box | CACGTC | 646 | 658 | light responsive element |
| SiRAF34 | SETIT_027523mg | GTGGC-motif | GATTCTGTGGC | 1300 | 1320 | light responsive element |
| SiRAF34 | SETIT_027523mg | AE-box | AGAAACAA | 1326 | 1342 | light responsive element |
| SiRAF34 | SETIT_027523mg | LTR | CCGAAA | 22 | 34 | low temperature response element |
| SiRAF34 | SETIT_027523mg | LTR | CCGAAA | 282 | 294 | low temperature response element |
| SiRAF34 | SETIT_027523mg | LTR | CCGAAA | 911 | 923 | low temperature response element |
| SiRAF34 | SETIT_027523mg | MBS | CAACTG | 931 | 943 | MYB binding site involved in drought-inducibility |
| SiRAF34 | SETIT_027523mg | MBS | CAACTG | 1384 | 1396 | MYB binding site involved in drought-inducibility |
| SiRAF34 | SETIT_027523mg | MBS | CAACTG | 1615 | 1627 | MYB binding site involved in drought-inducibility |
| SiRAF34 | SETIT_027523mg | A-box | CCGTCC | 350 | 362 | promoter and enhancer cis-acting regulatory elements |
| SiRAF34 | SETIT_027523mg | TCA-element | CCATCTTTTT | 284 | 302 | salicylic acid response element |
| SiRAF35 | SETIT_021033mg | ABRE | GCCGCGTGGC | 645 | 665 | abscisic acid response element |
| SiRAF35 | SETIT_021033mg | ABRE | AACCCGG | 1550 | 1564 | abscisic acid response element |
| SiRAF35 | SETIT_021033mg | ARE | AAACCA | 1083 | 1095 | anaerobic inducing element |
| SiRAF35 | SETIT_021033mg | ARE | AAACCA | 1606 | 1618 | anaerobic inducing element |
| SiRAF35 | SETIT_021033mg | ARE | AAACCA | 1829 | 1841 | anaerobic inducing element |
| SiRAF35 | SETIT_021033mg | TGA-element | AACGAC | 1100 | 1112 | auxin-responsive element |
| SiRAF35 | SETIT_021033mg | CAAT-box | CAAAT | 343 | 353 | common cis-acting element |
| SiRAF35 | SETIT_021033mg | CAAT-box | CAAAT | 420 | 430 | common cis-acting element |
| SiRAF35 | SETIT_021033mg | CAAT-box | CAAAT | 436 | 446 | common cis-acting element |
| SiRAF35 | SETIT_021033mg | CAAT-box | CAAAT | 840 | 850 | common cis-acting element |
| SiRAF35 | SETIT_021033mg | CAAT-box | CAAAT | 985 | 995 | common cis-acting element |
| SiRAF35 | SETIT_021033mg | CAAT-box | CAAAT | 991 | 1001 | common cis-acting element |
| SiRAF35 | SETIT_021033mg | CAAT-box | CAAAT | 1042 | 1052 | common cis-acting element |
| SiRAF35 | SETIT_021033mg | CAAT-box | CAAAT | 1508 | 1518 | common cis-acting element |
| SiRAF35 | SETIT_021033mg | CAAT-box | CCAAT | 1614 | 1624 | common cis-acting element |
| SiRAF35 | SETIT_021033mg | CAAT-box | CAAAT | 1625 | 1635 | common cis-acting element |
| SiRAF35 | SETIT_021033mg | CAAT-box | CAAAT | 1706 | 1716 | common cis-acting element |
| SiRAF35 | SETIT_021033mg | CAAT-box | CAAAT | 1753 | 1763 | common cis-acting element |
| SiRAF35 | SETIT_021033mg | TATA-box | TATA | 64 | 72 | core promoter element |
| SiRAF35 | SETIT_021033mg | TATA-box | ATATAT | 87 | 99 | core promoter element |
| SiRAF35 | SETIT_021033mg | TATA-box | TATA | 90 | 98 | core promoter element |
| SiRAF35 | SETIT_021033mg | TATA-box | ATTATA | 94 | 106 | core promoter element |
| SiRAF35 | SETIT_021033mg | TATA-box | TATAA | 96 | 106 | core promoter element |
| SiRAF35 | SETIT_021033mg | TATA-box | TATA | 98 | 106 | core promoter element |
| SiRAF35 | SETIT_021033mg | TATA-box | TATA | 127 | 135 | core promoter element |
| SiRAF35 | SETIT_021033mg | TATA-box | ATATAT | 145 | 157 | core promoter element |
| SiRAF35 | SETIT_021033mg | TATA-box | TATATA | 146 | 158 | core promoter element |
| SiRAF35 | SETIT_021033mg | TATA-box | ATATAT | 147 | 159 | core promoter element |
| SiRAF35 | SETIT_021033mg | TATA-box | TATA | 150 | 158 | core promoter element |
| SiRAF35 | SETIT_021033mg | TATA-box | TATAAAT | 177 | 191 | core promoter element |
| SiRAF35 | SETIT_021033mg | TATA-box | TATAAA | 179 | 191 | core promoter element |
| SiRAF35 | SETIT_021033mg | TATA-box | TATAA | 181 | 191 | core promoter element |
| SiRAF35 | SETIT_021033mg | TATA-box | TATA | 183 | 191 | core promoter element |
| SiRAF35 | SETIT_021033mg | TATA-box | TACAAAA | 245 | 259 | core promoter element |
| SiRAF35 | SETIT_021033mg | TATA-box | ATATAA | 438 | 450 | core promoter element |
| SiRAF35 | SETIT_021033mg | TATA-box | TATA | 441 | 449 | core promoter element |
| SiRAF35 | SETIT_021033mg | TATA-box | ATATAA | 541 | 553 | core promoter element |
| SiRAF35 | SETIT_021033mg | TATA-box | TATA | 544 | 552 | core promoter element |
| SiRAF35 | SETIT_021033mg | TATA-box | TATA | 1664 | 1672 | core promoter element |
| SiRAF35 | SETIT_021033mg | TATA-box | ATTATA | 1779 | 1791 | core promoter element |
| SiRAF35 | SETIT_021033mg | TATA-box | TATAA | 1781 | 1791 | core promoter element |
| SiRAF35 | SETIT_021033mg | TATA-box | TATA | 1783 | 1791 | core promoter element |
| SiRAF35 | SETIT_021033mg | TATA-box | TATA | 1856 | 1864 | core promoter element |
| SiRAF35 | SETIT_021033mg | TATA-box | TACAAAA | 1877 | 1891 | core promoter element |
| SiRAF35 | SETIT_021033mg | TC-rich repeats | GTTTTCTTAC | 1529 | 1547 | defense and stress response elements |
| SiRAF35 | SETIT_021033mg | GC-motif | CCCCCG | 869 | 881 | hypoxia-specifically induced elements |
| SiRAF35 | SETIT_021033mg | GC-motif | CCCCCG | 1548 | 1560 | hypoxia-specifically induced elements |
| SiRAF35 | SETIT_021033mg | G-box | GCCACGTGGA | 1896 | 1914 | light responsive element |
| SiRAF35 | SETIT_021033mg | Sp1 | GGGCGG | 750 | 762 | light responsive element |
| SiRAF35 | SETIT_021033mg | Sp1 | GGGCGG | 973 | 985 | light responsive element |
| SiRAF35 | SETIT_021033mg | Sp1 | GGGCGG | 1126 | 1138 | light responsive element |
| SiRAF35 | SETIT_021033mg | ATCT-motif | AATCTAATCC | 1035 | 1053 | light responsive element |
| SiRAF35 | SETIT_021033mg | GTGGC-motif | CAGCGTGTGGC | 1816 | 1836 | light responsive element |
| SiRAF35 | SETIT_021033mg | LAMP-element | CCTTATCCA | 798 | 816 | light responsive element |
| SiRAF35 | SETIT_021033mg | CGTCA-motif | CGTCA | 1977 | 1987 | MeJA response regulatory element |
| SiRAF35 | SETIT_021033mg | TGACG-motif | TGACG | 1977 | 1987 | MeJA response regulatory element |
| SiRAF35 | SETIT_021033mg | CAT-box | GCCACT | 1397 | 1409 | meristem expression regulatory element |
| SiRAF35 | SETIT_021033mg | CAT-box | GCCACT | 1947 | 1959 | meristem expression regulatory element |
| SiRAF35 | SETIT_021033mg | MBS | CAACTG | 475 | 487 | MYB binding site involved in drought-inducibility |
| SiRAF35 | SETIT_021033mg | A-box | CCGTCC | 1146 | 1158 | promoter and enhancer cis-acting regulatory elements |
| SiRAF35 | SETIT_021033mg | A-box | CCGTCC | 1305 | 1317 | promoter and enhancer cis-acting regulatory elements |
| SiRAF35 | SETIT_021033mg | A-box | CCGTCC | 1418 | 1430 | promoter and enhancer cis-acting regulatory elements |
| SiRAF35 | SETIT_021033mg | TCA-element | CCATCTTTTT | 1516 | 1534 | salicylic acid response element |
| SiRAF36 | SETIT_009795mg | ABRE | CGTACGTGCA | 10 | 28 | abscisic acid response element |
| SiRAF36 | SETIT_009795mg | ABRE | ACGTG | 318 | 328 | abscisic acid response element |
| SiRAF36 | SETIT_009795mg | ABRE | ACGTG | 1513 | 1523 | abscisic acid response element |
| SiRAF36 | SETIT_009795mg | ABRE | ACGTG | 1548 | 1558 | abscisic acid response element |
| SiRAF36 | SETIT_009795mg | ABRE | ACGTG | 1579 | 1589 | abscisic acid response element |
| SiRAF36 | SETIT_009795mg | ABRE | GCCGCGTGGC | 1660 | 1680 | abscisic acid response element |
| SiRAF36 | SETIT_009795mg | ABRE | ACGTG | 1762 | 1772 | abscisic acid response element |
| SiRAF36 | SETIT_009795mg | ABRE | ACGTG | 1969 | 1979 | abscisic acid response element |
| SiRAF36 | SETIT_009795mg | ARE | AAACCA | 917 | 929 | anaerobic inducing element |
| SiRAF36 | SETIT_009795mg | ARE | AAACCA | 1535 | 1547 | anaerobic inducing element |
| SiRAF36 | SETIT_009795mg | AuxRR-core | GGTCCAT | 68 | 82 | auxin response element |
| SiRAF36 | SETIT_009795mg | CAAT-box | TGCAAATCT | -2 | 16 | common cis-acting element |
| SiRAF36 | SETIT_009795mg | CAAT-box | CAAAT | 4 | 14 | common cis-acting element |
| SiRAF36 | SETIT_009795mg | CAAT-box | CCAAT | 418 | 428 | common cis-acting element |
| SiRAF36 | SETIT_009795mg | CAAT-box | CCAAT | 483 | 493 | common cis-acting element |
| SiRAF36 | SETIT_009795mg | CAAT-box | CCAAT | 517 | 527 | common cis-acting element |
| SiRAF36 | SETIT_009795mg | CAAT-box | CAAAT | 657 | 667 | common cis-acting element |
| SiRAF36 | SETIT_009795mg | CAAT-box | CAAAT | 669 | 679 | common cis-acting element |
| SiRAF36 | SETIT_009795mg | CAAT-box | CCAAT | 795 | 805 | common cis-acting element |
| SiRAF36 | SETIT_009795mg | CAAT-box | CAAAT | 802 | 812 | common cis-acting element |
| SiRAF36 | SETIT_009795mg | CAAT-box | CAAAT | 897 | 907 | common cis-acting element |
| SiRAF36 | SETIT_009795mg | CAAT-box | CAAAT | 994 | 1004 | common cis-acting element |
| SiRAF36 | SETIT_009795mg | CAAT-box | CAAAT | 1078 | 1088 | common cis-acting element |
| SiRAF36 | SETIT_009795mg | CAAT-box | CAAAT | 1182 | 1192 | common cis-acting element |
| SiRAF36 | SETIT_009795mg | TATA-box | TATA | 357 | 365 | core promoter element |
| SiRAF36 | SETIT_009795mg | TATA-box | TATAA | 648 | 658 | core promoter element |
| SiRAF36 | SETIT_009795mg | TATA-box | TATA | 650 | 658 | core promoter element |
| SiRAF36 | SETIT_009795mg | TATA-box | TACATAAA | 730 | 746 | core promoter element |
| SiRAF36 | SETIT_009795mg | TATA-box | TATAAA | 1174 | 1186 | core promoter element |
| SiRAF36 | SETIT_009795mg | TATA-box | TATAA | 1176 | 1186 | core promoter element |
| SiRAF36 | SETIT_009795mg | TATA-box | TATA | 1178 | 1186 | core promoter element |
| SiRAF36 | SETIT_009795mg | TATA-box | TATA | 1270 | 1278 | core promoter element |
| SiRAF36 | SETIT_009795mg | TATA-box | TATAA | 1292 | 1302 | core promoter element |
| SiRAF36 | SETIT_009795mg | TATA-box | TATA | 1294 | 1302 | core promoter element |
| SiRAF36 | SETIT_009795mg | TATC-box | TATCCCA | 1435 | 1449 | gibberellin response element |
| SiRAF36 | SETIT_009795mg | GC-motif | CCCCCG | 1693 | 1705 | hypoxia-specifically induced elements |
| SiRAF36 | SETIT_009795mg | GC-motif | CCCCCG | 1981 | 1993 | hypoxia-specifically induced elements |
| SiRAF36 | SETIT_009795mg | G-Box | CACGTT | 1547 | 1559 | light responsive element |
| SiRAF36 | SETIT_009795mg | G-Box | CACGTT | 1578 | 1590 | light responsive element |
| SiRAF36 | SETIT_009795mg | G-box | CACGTC | 317 | 329 | light responsive element |
| SiRAF36 | SETIT_009795mg | G-box | CACGAC | 320 | 332 | light responsive element |
| SiRAF36 | SETIT_009795mg | G-box | TACGTG | 1512 | 1524 | light responsive element |
| SiRAF36 | SETIT_009795mg | G-box | TACGTG | 1761 | 1773 | light responsive element |
| SiRAF36 | SETIT_009795mg | G-box | CACGTC | 1967 | 1979 | light responsive element |
| SiRAF36 | SETIT_009795mg | Sp1 | GGGCGG | 1899 | 1911 | light responsive element |
| SiRAF36 | SETIT_009795mg | Sp1 | GGGCGG | 1946 | 1958 | light responsive element |
| SiRAF36 | SETIT_009795mg | AAAC-motif | CAATCAAAACCT | 478 | 500 | light responsive element |
| SiRAF36 | SETIT_009795mg | Box 4 | ATTAAT | 467 | 479 | light responsive element |
| SiRAF36 | SETIT_009795mg | Box 4 | ATTAAT | 659 | 671 | light responsive element |
| SiRAF36 | SETIT_009795mg | GATA-motif | GATAGGA | 1824 | 1838 | light responsive element |
| SiRAF36 | SETIT_009795mg | chs-CMA2a | TCACTTGA | 1351 | 1367 | light responsive element |
| SiRAF36 | SETIT_009795mg | LTR | CCGAAA | 300 | 312 | low temperature response element |
| SiRAF36 | SETIT_009795mg | CGTCA-motif | CGTCA | 25 | 35 | MeJA response regulatory element |
| SiRAF36 | SETIT_009795mg | CGTCA-motif | CGTCA | 1011 | 1021 | MeJA response regulatory element |
| SiRAF36 | SETIT_009795mg | CGTCA-motif | CGTCA | 1967 | 1977 | MeJA response regulatory element |
| SiRAF36 | SETIT_009795mg | TGACG-motif | TGACG | 25 | 35 | MeJA response regulatory element |
| SiRAF36 | SETIT_009795mg | TGACG-motif | TGACG | 1011 | 1021 | MeJA response regulatory element |
| SiRAF36 | SETIT_009795mg | TGACG-motif | TGACG | 1967 | 1977 | MeJA response regulatory element |
| SiRAF36 | SETIT_009795mg | O2-site | GATGACATGG | 1437 | 1455 | Zein metabolism regulatory elements |
| SiRAF37 | SETIT_026192mg | ABRE | ACGTG | 35 | 45 | abscisic acid response element |
| SiRAF37 | SETIT_026192mg | ARE | AAACCA | 1456 | 1468 | anaerobic inducing element |
| SiRAF37 | SETIT_026192mg | CAAT-box | CCAAT | 13 | 23 | common cis-acting element |
| SiRAF37 | SETIT_026192mg | CAAT-box | CCAAT | 43 | 53 | common cis-acting element |
| SiRAF37 | SETIT_026192mg | CAAT-box | CCAAT | 157 | 167 | common cis-acting element |
| SiRAF37 | SETIT_026192mg | CAAT-box | CCAAT | 564 | 574 | common cis-acting element |
| SiRAF37 | SETIT_026192mg | CAAT-box | CCAAT | 635 | 645 | common cis-acting element |
| SiRAF37 | SETIT_026192mg | CAAT-box | CCAAT | 712 | 722 | common cis-acting element |
| SiRAF37 | SETIT_026192mg | CAAT-box | CCAAT | 764 | 774 | common cis-acting element |
| SiRAF37 | SETIT_026192mg | CAAT-box | CAAAT | 845 | 855 | common cis-acting element |
| SiRAF37 | SETIT_026192mg | CAAT-box | CCAAT | 872 | 882 | common cis-acting element |
| SiRAF37 | SETIT_026192mg | CAAT-box | CAAAT | 882 | 892 | common cis-acting element |
| SiRAF37 | SETIT_026192mg | CAAT-box | CAAAT | 908 | 918 | common cis-acting element |
| SiRAF37 | SETIT_026192mg | CAAT-box | CCAAT | 1097 | 1107 | common cis-acting element |
| SiRAF37 | SETIT_026192mg | CAAT-box | CAAAT | 1163 | 1173 | common cis-acting element |
| SiRAF37 | SETIT_026192mg | CAAT-box | CAAAT | 1208 | 1218 | common cis-acting element |
| SiRAF37 | SETIT_026192mg | CAAT-box | CCAAT | 1228 | 1238 | common cis-acting element |
| SiRAF37 | SETIT_026192mg | CAAT-box | CAAAT | 1249 | 1259 | common cis-acting element |
| SiRAF37 | SETIT_026192mg | CAAT-box | CAAAT | 1573 | 1583 | common cis-acting element |
| SiRAF37 | SETIT_026192mg | CAAT-box | CAAAT | 1604 | 1614 | common cis-acting element |
| SiRAF37 | SETIT_026192mg | TATA-box | taTATAAAtc | 369 | 387 | core promoter element |
| SiRAF37 | SETIT_026192mg | TATA-box | TATA | 376 | 384 | core promoter element |
| SiRAF37 | SETIT_026192mg | TATA-box | TATAA | 729 | 739 | core promoter element |
| SiRAF37 | SETIT_026192mg | TATA-box | TATA | 731 | 739 | core promoter element |
| SiRAF37 | SETIT_026192mg | TATA-box | ATATAT | 743 | 755 | core promoter element |
| SiRAF37 | SETIT_026192mg | TATA-box | TATA | 746 | 754 | core promoter element |
| SiRAF37 | SETIT_026192mg | TATA-box | ATATAT | 897 | 909 | core promoter element |
| SiRAF37 | SETIT_026192mg | TATA-box | TATA | 900 | 908 | core promoter element |
| SiRAF37 | SETIT_026192mg | TATA-box | TATAA | 933 | 943 | core promoter element |
| SiRAF37 | SETIT_026192mg | TATA-box | TATA | 935 | 943 | core promoter element |
| SiRAF37 | SETIT_026192mg | TATA-box | TATA | 999 | 1007 | core promoter element |
| SiRAF37 | SETIT_026192mg | TATA-box | TATA | 1137 | 1145 | core promoter element |
| SiRAF37 | SETIT_026192mg | TATA-box | TATA | 1185 | 1193 | core promoter element |
| SiRAF37 | SETIT_026192mg | TATA-box | TATAAA | 1322 | 1334 | core promoter element |
| SiRAF37 | SETIT_026192mg | TATA-box | TATAA | 1324 | 1334 | core promoter element |
| SiRAF37 | SETIT_026192mg | TATA-box | TATA | 1326 | 1334 | core promoter element |
| SiRAF37 | SETIT_026192mg | TATA-box | TATA | 1452 | 1460 | core promoter element |
| SiRAF37 | SETIT_026192mg | TATA-box | TACAAAA | 1451 | 1465 | core promoter element |
| SiRAF37 | SETIT_026192mg | TATA-box | ATATAA | 1471 | 1483 | core promoter element |
| SiRAF37 | SETIT_026192mg | TATA-box | TATA | 1474 | 1482 | core promoter element |
| SiRAF37 | SETIT_026192mg | RY-element | CATGCATG | 962 | 978 | elements involved in seed-specific regulation |
| SiRAF37 | SETIT_026192mg | P-box | CCTTTTG | 916 | 930 | gibberellin-responsive element |
| SiRAF37 | SETIT_026192mg | GC-motif | CCCCCG | 1780 | 1792 | hypoxia-specifically induced elements |
| SiRAF37 | SETIT_026192mg | ACE | GCGACGTACC | 1931 | 1949 | light responsive element |
| SiRAF37 | SETIT_026192mg | G-box | CACGTC | 33 | 45 | light responsive element |
| SiRAF37 | SETIT_026192mg | Sp1 | GGGCGG | 242 | 254 | light responsive element |
| SiRAF37 | SETIT_026192mg | Box 4 | ATTAAT | 329 | 341 | light responsive element |
| SiRAF37 | SETIT_026192mg | Box 4 | ATTAAT | 1101 | 1113 | light responsive element |
| SiRAF37 | SETIT_026192mg | Box 4 | ATTAAT | 1432 | 1444 | light responsive element |
| SiRAF37 | SETIT_026192mg | Box 4 | ATTAAT | 1439 | 1451 | light responsive element |
| SiRAF37 | SETIT_026192mg | Box 4 | ATTAAT | 1467 | 1479 | light responsive element |
| SiRAF37 | SETIT_026192mg | TCT-motif | TCTTAC | 1268 | 1280 | light responsive element |
| SiRAF37 | SETIT_026192mg | TCT-motif | TCTTAC | 1767 | 1779 | light responsive element |
| SiRAF37 | SETIT_026192mg | I-box | GTATAAGGCC | 925 | 943 | light responsive element |
| SiRAF37 | SETIT_026192mg | GATA-motif | GATAGGG | 1631 | 1645 | light responsive element |
| SiRAF37 | SETIT_026192mg | LTR | CCGAAA | 1783 | 1795 | low temperature response element |
| SiRAF37 | SETIT_026192mg | CGTCA-motif | CGTCA | 33 | 43 | MeJA response regulatory element |
| SiRAF37 | SETIT_026192mg | TGACG-motif | TGACG | 33 | 43 | MeJA response regulatory element |
| SiRAF37 | SETIT_026192mg | CAT-box | GCCACT | 449 | 461 | meristem expression regulatory element |
| SiRAF37 | SETIT_026192mg | MBS | CAACTG | 1617 | 1629 | MYB binding site involved in drought-inducibility |
| SiRAF37 | SETIT_026192mg | CCAAT-box | CAACGG | 1520 | 1532 | MYBHv1 binding site |
| SiRAF37 | SETIT_026192mg | A-box | CCGTCC | 1724 | 1736 | promoter and enhancer cis-acting regulatory elements |
| SiRAF37 | SETIT_026192mg | TCA-element | CCATCTTTTT | 380 | 398 | salicylic acid response element |
| SiRAF37 | SETIT_026192mg | O2-site | GATGATGTGG | 111 | 129 | Zein metabolism regulatory elements |
| SiRAF38 | SETIT_024868mg | ABRE | ACGTG | 614 | 624 | abscisic acid response element |
| SiRAF38 | SETIT_024868mg | ABRE | AACCCGG | 1086 | 1100 | abscisic acid response element |
| SiRAF38 | SETIT_024868mg | ABRE | GCCGCGTGGC | 1543 | 1561 | abscisic acid response element |
| SiRAF38 | SETIT_024868mg | ARE | AAACCA | 478 | 490 | anaerobic inducing element |
| SiRAF38 | SETIT_024868mg | ARE | AAACCA | 555 | 567 | anaerobic inducing element |
| SiRAF38 | SETIT_024868mg | CAAT-box | CAAAT | 207 | 217 | common cis-acting element |
| SiRAF38 | SETIT_024868mg | CAAT-box | CAAAT | 263 | 273 | common cis-acting element |
| SiRAF38 | SETIT_024868mg | CAAT-box | CAAAT | 344 | 354 | common cis-acting element |
| SiRAF38 | SETIT_024868mg | CAAT-box | CCAAT | 504 | 514 | common cis-acting element |
| SiRAF38 | SETIT_024868mg | CAAT-box | CAAAT | 650 | 660 | common cis-acting element |
| SiRAF38 | SETIT_024868mg | CAAT-box | CAAAT | 1199 | 1209 | common cis-acting element |
| SiRAF38 | SETIT_024868mg | CAAT-box | CAAAT | 1296 | 1306 | common cis-acting element |
| SiRAF38 | SETIT_024868mg | CAAT-box | CCAAT | 1415 | 1425 | common cis-acting element |
| SiRAF38 | SETIT_024868mg | CAAT-box | CAAAT | 1616 | 1626 | common cis-acting element |
| SiRAF38 | SETIT_024868mg | CAAT-box | CCAAT | 1724 | 1734 | common cis-acting element |
| SiRAF38 | SETIT_024868mg | CAAT-box | CAAAT | 1762 | 1772 | common cis-acting element |
| SiRAF38 | SETIT_024868mg | TATA-box | TATAAA | 1231 | 1243 | core promoter element |
| SiRAF38 | SETIT_024868mg | TATA-box | TATAA | 1233 | 1243 | core promoter element |
| SiRAF38 | SETIT_024868mg | TATA-box | TATA | 1235 | 1243 | core promoter element |
| SiRAF38 | SETIT_024868mg | TATA-box | tcTATAAATAgg | 1786 | 1808 | core promoter element |
| SiRAF38 | SETIT_024868mg | TATA-box | TATA | 1795 | 1803 | core promoter element |
| SiRAF38 | SETIT_024868mg | P-box | CCTTTTG | 1714 | 1728 | gibberellin-responsive element |
| SiRAF38 | SETIT_024868mg | GC-motif | CCCCCG | 418 | 430 | hypoxia-specifically induced elements |
| SiRAF38 | SETIT_024868mg | GC-motif | CCCCCG | 1782 | 1794 | hypoxia-specifically induced elements |
| SiRAF38 | SETIT_024868mg | GC-motif | CCCCCG | 1852 | 1864 | hypoxia-specifically induced elements |
| SiRAF38 | SETIT_024868mg | GC-motif | CCCCCG | 1881 | 1893 | hypoxia-specifically induced elements |
| SiRAF38 | SETIT_024868mg | G-box | CACGAC | 2 | 14 | light responsive element |
| SiRAF38 | SETIT_024868mg | G-box | CACGAC | 181 | 193 | light responsive element |
| SiRAF38 | SETIT_024868mg | G-box | CACGAC | 609 | 621 | light responsive element |
| SiRAF38 | SETIT_024868mg | G-box | CACGTC | 612 | 624 | light responsive element |
| SiRAF38 | SETIT_024868mg | G-box | CACGAC | 1580 | 1592 | light responsive element |
| SiRAF38 | SETIT_024868mg | Sp1 | GGGCGG | 1166 | 1178 | light responsive element |
| SiRAF38 | SETIT_024868mg | Sp1 | GGGCGG | 1481 | 1493 | light responsive element |
| SiRAF38 | SETIT_024868mg | Sp1 | GGGCGG | 1785 | 1797 | light responsive element |
| SiRAF38 | SETIT_024868mg | Sp1 | GGGCGG | 1855 | 1867 | light responsive element |
| SiRAF38 | SETIT_024868mg | Sp1 | GGGCGG | 1859 | 1871 | light responsive element |
| SiRAF38 | SETIT_024868mg | Sp1 | GGGCGG | 1916 | 1928 | light responsive element |
| SiRAF38 | SETIT_024868mg | GT1-motif | GGTTAAT | 1089 | 1103 | light responsive element |
| SiRAF38 | SETIT_024868mg | ATCT-motif | AATCTAATCC | 284 | 302 | light responsive element |
| SiRAF38 | SETIT_024868mg | Box 4 | ATTAAT | 827 | 839 | light responsive element |
| SiRAF38 | SETIT_024868mg | chs-Unit 1 m1 | ACCTAACCCGG | 1175 | 1195 | light responsive element |
| SiRAF38 | SETIT_024868mg | LTR | CCGAAA | 295 | 307 | low temperature response element |
| SiRAF38 | SETIT_024868mg | TGACG-motif | TGACG | 750 | 760 | MeJA response regulatory element |
| SiRAF38 | SETIT_024868mg | TGACG-motif | TGACG | 1818 | 1828 | MeJA response regulatory element |
| SiRAF38 | SETIT_024868mg | CGTCA-motif | CGTCA | 750 | 760 | MeJA response regulatory element |
| SiRAF38 | SETIT_024868mg | CGTCA-motif | CGTCA | 1818 | 1828 | MeJA response regulatory element |
| SiRAF38 | SETIT_024868mg | CAT-box | GCCACT | 348 | 360 | meristem expression regulatory element |
| SiRAF38 | SETIT_024868mg | MBS | CAACTG | 577 | 589 | MYB binding site involved in drought-inducibility |
| SiRAF38 | SETIT_024868mg | MRE | AACCTAA | 1183 | 1197 | MYB binding site involved in light responsiveness |
| SiRAF38 | SETIT_024868mg | MRE | AACCTAA | 1301 | 1315 | MYB binding site involved in light responsiveness |
| SiRAF38 | SETIT_024868mg | CCAAT-box | CAACGG | 73 | 85 | MYBHv1 binding site |
| SiRAF38 | SETIT_024868mg | A-box | CCGTCC | 359 | 371 | promoter and enhancer cis-acting regulatory elements |
| SiRAF38 | SETIT_024868mg | A-box | CCGTCC | 1410 | 1422 | promoter and enhancer cis-acting regulatory elements |
| SiRAF38 | SETIT_024868mg | A-box | CCGTCC | 1510 | 1522 | promoter and enhancer cis-acting regulatory elements |
| SiRAF38 | SETIT_024868mg | Unnamed__1 | GAATTTAATTAA | 1235 | 1257 | protein binding site |
| SiZIK1 | SETIT_021438mg | ABRE | ACGTG | 672 | 682 | abscisic acid response element |
| SiZIK1 | SETIT_021438mg | ABRE | ACGTG | 810 | 820 | abscisic acid response element |
| SiZIK1 | SETIT_021438mg | ABRE | TACGTGTC | 856 | 872 | abscisic acid response element |
| SiZIK1 | SETIT_021438mg | ABRE | ACGTG | 861 | 871 | abscisic acid response element |
| SiZIK1 | SETIT_021438mg | ARE | AAACCA | 1561 | 1573 | anaerobic inducing element |
| SiZIK1 | SETIT_021438mg | AuxRR-core | GGTCCAT | 1203 | 1217 | auxin response element |
| SiZIK1 | SETIT_021438mg | TGA-element | AACGAC | 1324 | 1336 | auxin-responsive element |
| SiZIK1 | SETIT_021438mg | CAAT-box | CAAAT | 204 | 214 | common cis-acting element |
| SiZIK1 | SETIT_021438mg | CAAT-box | CAAAT | 210 | 220 | common cis-acting element |
| SiZIK1 | SETIT_021438mg | CAAT-box | CCAAT | 392 | 402 | common cis-acting element |
| SiZIK1 | SETIT_021438mg | CAAT-box | CCAAT | 550 | 560 | common cis-acting element |
| SiZIK1 | SETIT_021438mg | CAAT-box | CCAAT | 837 | 847 | common cis-acting element |
| SiZIK1 | SETIT_021438mg | CAAT-box | CAAAT | 848 | 858 | common cis-acting element |
| SiZIK1 | SETIT_021438mg | CAAT-box | CCAAT | 973 | 983 | common cis-acting element |
| SiZIK1 | SETIT_021438mg | CAAT-box | CAAAT | 997 | 1007 | common cis-acting element |
| SiZIK1 | SETIT_021438mg | CAAT-box | CAAAT | 1061 | 1071 | common cis-acting element |
| SiZIK1 | SETIT_021438mg | CAAT-box | CAAAT | 1077 | 1087 | common cis-acting element |
| SiZIK1 | SETIT_021438mg | CAAT-box | CCAAT | 1112 | 1122 | common cis-acting element |
| SiZIK1 | SETIT_021438mg | CAAT-box | CAAAT | 1236 | 1246 | common cis-acting element |
| SiZIK1 | SETIT_021438mg | CAAT-box | CAAAT | 1336 | 1346 | common cis-acting element |
| SiZIK1 | SETIT_021438mg | CAAT-box | CAAAT | 1368 | 1378 | common cis-acting element |
| SiZIK1 | SETIT_021438mg | CAAT-box | CAAAT | 1485 | 1495 | common cis-acting element |
| SiZIK1 | SETIT_021438mg | CAAT-box | CAAAT | 1532 | 1542 | common cis-acting element |
| SiZIK1 | SETIT_021438mg | CAAT-box | CCAAT | 1911 | 1921 | common cis-acting element |
| SiZIK1 | SETIT_021438mg | TATA-box | TATATA | 360 | 372 | core promoter element |
| SiZIK1 | SETIT_021438mg | TATA-box | ATATAA | 361 | 373 | core promoter element |
| SiZIK1 | SETIT_021438mg | TATA-box | TATA | 364 | 372 | core promoter element |
| SiZIK1 | SETIT_021438mg | TATA-box | TATA | 526 | 534 | core promoter element |
| SiZIK1 | SETIT_021438mg | TATA-box | TATACA | 630 | 642 | core promoter element |
| SiZIK1 | SETIT_021438mg | TATA-box | TATATA | 632 | 644 | core promoter element |
| SiZIK1 | SETIT_021438mg | TATA-box | TATA | 636 | 644 | core promoter element |
| SiZIK1 | SETIT_021438mg | TATA-box | TATA | 710 | 718 | core promoter element |
| SiZIK1 | SETIT_021438mg | TATA-box | taTATAAAtc | 1024 | 1042 | core promoter element |
| SiZIK1 | SETIT_021438mg | TATA-box | TATA | 1031 | 1039 | core promoter element |
| SiZIK1 | SETIT_021438mg | TATA-box | ATATAT | 1133 | 1145 | core promoter element |
| SiZIK1 | SETIT_021438mg | TATA-box | TATATA | 1134 | 1146 | core promoter element |
| SiZIK1 | SETIT_021438mg | TATA-box | TATA | 1138 | 1146 | core promoter element |
| SiZIK1 | SETIT_021438mg | TATA-box | TATA | 1160 | 1168 | core promoter element |
| SiZIK1 | SETIT_021438mg | TATA-box | TATA | 1189 | 1197 | core promoter element |
| SiZIK1 | SETIT_021438mg | TATA-box | TATA | 1194 | 1202 | core promoter element |
| SiZIK1 | SETIT_021438mg | TATA-box | ATATAT | 1209 | 1221 | core promoter element |
| SiZIK1 | SETIT_021438mg | TATA-box | TATATA | 1210 | 1222 | core promoter element |
| SiZIK1 | SETIT_021438mg | TATA-box | ATATAA | 1211 | 1223 | core promoter element |
| SiZIK1 | SETIT_021438mg | TATA-box | TATA | 1214 | 1222 | core promoter element |
| SiZIK1 | SETIT_021438mg | TATA-box | TATA | 1275 | 1283 | core promoter element |
| SiZIK1 | SETIT_021438mg | TATA-box | TATA | 1457 | 1465 | core promoter element |
| SiZIK1 | SETIT_021438mg | TATA-box | ATTATA | 1550 | 1562 | core promoter element |
| SiZIK1 | SETIT_021438mg | TATA-box | TATAA | 1552 | 1562 | core promoter element |
| SiZIK1 | SETIT_021438mg | TATA-box | TATA | 1554 | 1562 | core promoter element |
| SiZIK1 | SETIT_021438mg | TATA-box | TATAAAA | 1588 | 1602 | core promoter element |
| SiZIK1 | SETIT_021438mg | TATA-box | TATAAA | 1590 | 1602 | core promoter element |
| SiZIK1 | SETIT_021438mg | TATA-box | TATAA | 1592 | 1602 | core promoter element |
| SiZIK1 | SETIT_021438mg | TATA-box | TATA | 1594 | 1602 | core promoter element |
| SiZIK1 | SETIT_021438mg | P-box | CCTTTTG | 570 | 584 | gibberellin-responsive element |
| SiZIK1 | SETIT_021438mg | ACE | GACACGTATG | 854 | 874 | light responsive element |
| SiZIK1 | SETIT_021438mg | ACE | GCGACGTACC | 1925 | 1943 | light responsive element |
| SiZIK1 | SETIT_021438mg | G-box | TACGTG | 671 | 683 | light responsive element |
| SiZIK1 | SETIT_021438mg | G-box | TACGTG | 808 | 820 | light responsive element |
| SiZIK1 | SETIT_021438mg | G-box | TACGTG | 860 | 872 | light responsive element |
| SiZIK1 | SETIT_021438mg | GT1-motif | GGTTAA | 1499 | 1511 | light responsive element |
| SiZIK1 | SETIT_021438mg | Sp1 | GGGCGG | 1833 | 1845 | light responsive element |
| SiZIK1 | SETIT_021438mg | Sp1 | GGGCGG | 1974 | 1986 | light responsive element |
| SiZIK1 | SETIT_021438mg | TCT-motif | TCTTAC | 921 | 933 | light responsive element |
| SiZIK1 | SETIT_021438mg | CGTCA-motif | CGTCA | 468 | 478 | MeJA response regulatory element |
| SiZIK1 | SETIT_021438mg | CGTCA-motif | CGTCA | 930 | 940 | MeJA response regulatory element |
| SiZIK1 | SETIT_021438mg | TGACG-motif | TGACG | 468 | 478 | MeJA response regulatory element |
| SiZIK1 | SETIT_021438mg | TGACG-motif | TGACG | 930 | 940 | MeJA response regulatory element |
| SiZIK1 | SETIT_021438mg | CCAAT-box | CAACGG | 335 | 347 | MYBHv1 binding site |
| SiZIK1 | SETIT_021438mg | A-box | CCGTCC | 1969 | 1981 | promoter and enhancer cis-acting regulatory elements |
| SiZIK1 | SETIT_021438mg | O2-site | GTTGACGTGA | 783 | 801 | Zein metabolism regulatory elements |
| SiZIK2 | SETIT_016704mg | ABRE | CGTACGTGCA | 191 | 209 | abscisic acid response element |
| SiZIK2 | SETIT_016704mg | ABRE | ACGTG | 198 | 208 | abscisic acid response element |
| SiZIK2 | SETIT_016704mg | ARE | AAACCA | 399 | 411 | anaerobic inducing element |
| SiZIK2 | SETIT_016704mg | ARE | AAACCA | 1174 | 1186 | anaerobic inducing element |
| SiZIK2 | SETIT_016704mg | CAAT-box | CCAAT | 71 | 81 | common cis-acting element |
| SiZIK2 | SETIT_016704mg | CAAT-box | CCAAT | 168 | 178 | common cis-acting element |
| SiZIK2 | SETIT_016704mg | CAAT-box | CAAAT | 183 | 193 | common cis-acting element |
| SiZIK2 | SETIT_016704mg | CAAT-box | CAAAT | 207 | 217 | common cis-acting element |
| SiZIK2 | SETIT_016704mg | CAAT-box | CAAAT | 573 | 583 | common cis-acting element |
| SiZIK2 | SETIT_016704mg | CAAT-box | CAAAT | 1010 | 1020 | common cis-acting element |
| SiZIK2 | SETIT_016704mg | CAAT-box | CAAAT | 1126 | 1136 | common cis-acting element |
| SiZIK2 | SETIT_016704mg | CAAT-box | CAAAT | 1193 | 1203 | common cis-acting element |
| SiZIK2 | SETIT_016704mg | CAAT-box | CCAAT | 1330 | 1340 | common cis-acting element |
| SiZIK2 | SETIT_016704mg | CAAT-box | CAAAT | 1376 | 1386 | common cis-acting element |
| SiZIK2 | SETIT_016704mg | CAAT-box | CAAAT | 1386 | 1396 | common cis-acting element |
| SiZIK2 | SETIT_016704mg | CAAT-box | CCAAT | 1395 | 1405 | common cis-acting element |
| SiZIK2 | SETIT_016704mg | CAAT-box | CCAAT | 1406 | 1416 | common cis-acting element |
| SiZIK2 | SETIT_016704mg | CAAT-box | CCAAT | 1807 | 1817 | common cis-acting element |
| SiZIK2 | SETIT_016704mg | CAAT-box | CAAAT | 1961 | 1971 | common cis-acting element |
| SiZIK2 | SETIT_016704mg | TATA-box | TATACA | 47 | 59 | core promoter element |
| SiZIK2 | SETIT_016704mg | TATA-box | TATA | 51 | 59 | core promoter element |
| SiZIK2 | SETIT_016704mg | TATA-box | TATAAAA | 57 | 71 | core promoter element |
| SiZIK2 | SETIT_016704mg | TATA-box | TATAAA | 59 | 71 | core promoter element |
| SiZIK2 | SETIT_016704mg | TATA-box | TATAA | 61 | 71 | core promoter element |
| SiZIK2 | SETIT_016704mg | TATA-box | TATA | 63 | 71 | core promoter element |
| SiZIK2 | SETIT_016704mg | TATA-box | TATACA | 185 | 197 | core promoter element |
| SiZIK2 | SETIT_016704mg | TATA-box | TATA | 189 | 197 | core promoter element |
| SiZIK2 | SETIT_016704mg | TATA-box | TACAAAA | 649 | 663 | core promoter element |
| SiZIK2 | SETIT_016704mg | TATA-box | TATTTAAA | 1280 | 1296 | core promoter element |
| SiZIK2 | SETIT_016704mg | TATA-box | TATAA | 1313 | 1323 | core promoter element |
| SiZIK2 | SETIT_016704mg | TATA-box | TATA | 1315 | 1323 | core promoter element |
| SiZIK2 | SETIT_016704mg | TATA-box | TATAAA | 1582 | 1594 | core promoter element |
| SiZIK2 | SETIT_016704mg | TATA-box | TATAA | 1584 | 1594 | core promoter element |
| SiZIK2 | SETIT_016704mg | TATA-box | TATA | 1586 | 1594 | core promoter element |
| SiZIK2 | SETIT_016704mg | TATA-box | ATATAA | 1689 | 1701 | core promoter element |
| SiZIK2 | SETIT_016704mg | TATA-box | TATA | 1692 | 1700 | core promoter element |
| SiZIK2 | SETIT_016704mg | TATA-box | TATAA | 1724 | 1734 | core promoter element |
| SiZIK2 | SETIT_016704mg | TATA-box | TATA | 1726 | 1734 | core promoter element |
| SiZIK2 | SETIT_016704mg | TATA-box | ATATAT | 1854 | 1866 | core promoter element |
| SiZIK2 | SETIT_016704mg | TATA-box | TATA | 1857 | 1865 | core promoter element |
| SiZIK2 | SETIT_016704mg | TATA-box | TACAAAA | 1924 | 1938 | core promoter element |
| SiZIK2 | SETIT_016704mg | TC-rich repeats | ATTCTCTAAC | 1620 | 1638 | defense and stress response elements |
| SiZIK2 | SETIT_016704mg | ACE | GCGACGTACC | 691 | 709 | light responsive element |
| SiZIK2 | SETIT_016704mg | G-box | TACGTG | 196 | 208 | light responsive element |
| SiZIK2 | SETIT_016704mg | GT1-motif | GGTTAA | 35 | 47 | light responsive element |
| SiZIK2 | SETIT_016704mg | GT1-motif | GGTTAAT | 64 | 78 | light responsive element |
| SiZIK2 | SETIT_016704mg | GT1-motif | GGTTAA | 66 | 78 | light responsive element |
| SiZIK2 | SETIT_016704mg | Box 4 | ATTAAT | 3 | 15 | light responsive element |
| SiZIK2 | SETIT_016704mg | Box 4 | ATTAAT | 1619 | 1631 | light responsive element |
| SiZIK2 | SETIT_016704mg | I-box | gGATAAGGTG | 1051 | 1069 | light responsive element |
| SiZIK2 | SETIT_016704mg | GATT-motif | CTCCTGATTAGC | 78 | 100 | light responsive element |
| SiZIK2 | SETIT_016704mg | TCCC-motif | TCTCCCT | 406 | 420 | light responsive element |
| SiZIK2 | SETIT_016704mg | TCCC-motif | TCTCCCT | 469 | 483 | light responsive element |
| SiZIK2 | SETIT_016704mg | TCCC-motif | TCTCCCT | 720 | 734 | light responsive element |
| SiZIK2 | SETIT_016704mg | ACA-motif | AATCACAACCATA | 1332 | 1356 | light responsive element |
| SiZIK2 | SETIT_016704mg | LTR | CCGAAA | 1132 | 1144 | low temperature response element |
| SiZIK2 | SETIT_016704mg | LTR | CCGAAA | 1222 | 1234 | low temperature response element |
| SiZIK2 | SETIT_016704mg | TGACG-motif | TGACG | 276 | 286 | MeJA response regulatory element |
| SiZIK2 | SETIT_016704mg | CGTCA-motif | CGTCA | 276 | 286 | MeJA response regulatory element |
| SiZIK2 | SETIT_016704mg | MBS | CAACTG | 1982 | 1994 | MYB binding site involved in drought-inducibility |
| SiZIK2 | SETIT_016704mg | MRE | AACCTAA | 7 | 21 | MYB binding site involved in light responsiveness |
| SiZIK2 | SETIT_016704mg | MRE | AACCTAA | 1167 | 1181 | MYB binding site involved in light responsiveness |
| SiZIK2 | SETIT_016704mg | A-box | CCGTCC | 785 | 797 | promoter and enhancer cis-acting regulatory elements |
| SiZIK3 | SETIT_029371mg | TGACG-motif | TGACG | 387 | 397 | MeJA response regulatory element |
| SiZIK3 | SETIT_029371mg | TGACG-motif | TGACG | 523 | 533 | MeJA response regulatory element |
| SiZIK3 | SETIT_029371mg | TGACG-motif | TGACG | 642 | 652 | MeJA response regulatory element |
| SiZIK3 | SETIT_029371mg | TGACG-motif | TGACG | 789 | 799 | MeJA response regulatory element |
| SiZIK3 | SETIT_029371mg | TGACG-motif | TGACG | 1438 | 1448 | MeJA response regulatory element |
| SiZIK3 | SETIT_029371mg | TGACG-motif | TGACG | 1496 | 1506 | MeJA response regulatory element |
| SiZIK3 | SETIT_029371mg | TGACG-motif | TGACG | 1656 | 1666 | MeJA response regulatory element |
| SiZIK3 | SETIT_029371mg | MBS | CAACTG | 1392 | 1404 | MYB binding site involved in drought-inducibility |
| SiZIK3 | SETIT_029371mg | MRE | AACCTAA | 1061 | 1075 | MYB binding site involved in light responsiveness |
| SiZIK3 | SETIT_029371mg | CCAAT-box | CAACGG | 772 | 784 | MYBHv1 binding site |
| SiZIK3 | SETIT_029371mg | CCAAT-box | CAACGG | 1269 | 1281 | MYBHv1 binding site |
| SiZIK3 | SETIT_029371mg | CCAAT-box | CAACGG | 1637 | 1649 | MYBHv1 binding site |
| SiZIK3 | SETIT_029371mg | TCA-element | TCAGAAGAGG | 1722 | 1740 | salicylic acid response element |
| SiZIK3 | SETIT_029371mg | O2-site | GATGACATGG | 731 | 749 | Zein metabolism regulatory elements |
| SiZIK3 | SETIT_029371mg | O2-site | GATGATGTGG | 1902 | 1920 | Zein metabolism regulatory elements |
| SiZIK4 | SETIT_026086mg | ABRE | CGCACGTGTC | 1645 | 1663 | abscisic acid response element |
| SiZIK4 | SETIT_026086mg | ARE | AAACCA | 385 | 397 | anaerobic inducing element |
| SiZIK4 | SETIT_026086mg | ARE | AAACCA | 1054 | 1066 | anaerobic inducing element |
| SiZIK4 | SETIT_026086mg | ARE | AAACCA | 1351 | 1363 | anaerobic inducing element |
| SiZIK4 | SETIT_026086mg | AuxRR-core | GGTCCAT | 60 | 74 | auxin response element |
| SiZIK4 | SETIT_026086mg | TGA-element | AACGAC | 582 | 594 | auxin-responsive element |
| SiZIK4 | SETIT_026086mg | CAAT-box | CAAAT | 52 | 62 | common cis-acting element |
| SiZIK4 | SETIT_026086mg | CAAT-box | CAAAT | 96 | 106 | common cis-acting element |
| SiZIK4 | SETIT_026086mg | CAAT-box | CAAAT | 114 | 124 | common cis-acting element |
| SiZIK4 | SETIT_026086mg | CAAT-box | CAAAT | 117 | 127 | common cis-acting element |
| SiZIK4 | SETIT_026086mg | CAAT-box | CCAAT | 255 | 265 | common cis-acting element |
| SiZIK4 | SETIT_026086mg | CAAT-box | CAAAT | 345 | 355 | common cis-acting element |
| SiZIK4 | SETIT_026086mg | CAAT-box | CCAAT | 400 | 410 | common cis-acting element |
| SiZIK4 | SETIT_026086mg | CAAT-box | CCAAT | 563 | 573 | common cis-acting element |
| SiZIK4 | SETIT_026086mg | CAAT-box | CAAAT | 664 | 674 | common cis-acting element |
| SiZIK4 | SETIT_026086mg | CAAT-box | CAAAT | 730 | 740 | common cis-acting element |
| SiZIK4 | SETIT_026086mg | CAAT-box | CCAAT | 765 | 775 | common cis-acting element |
| SiZIK4 | SETIT_026086mg | CAAT-box | CCAAT | 888 | 898 | common cis-acting element |
| SiZIK4 | SETIT_026086mg | CAAT-box | CAAAT | 1003 | 1013 | common cis-acting element |
| SiZIK4 | SETIT_026086mg | CAAT-box | CCAAT | 1053 | 1063 | common cis-acting element |
| SiZIK4 | SETIT_026086mg | CAAT-box | CCAAT | 1078 | 1088 | common cis-acting element |
| SiZIK4 | SETIT_026086mg | CAAT-box | CCAAT | 1081 | 1091 | common cis-acting element |
| SiZIK4 | SETIT_026086mg | CAAT-box | CAAAT | 1190 | 1200 | common cis-acting element |
| SiZIK4 | SETIT_026086mg | CAAT-box | CAAAT | 1247 | 1257 | common cis-acting element |
| SiZIK4 | SETIT_026086mg | CAAT-box | CAAAT | 1308 | 1318 | common cis-acting element |
| SiZIK4 | SETIT_026086mg | CAAT-box | CCAAT | 1474 | 1484 | common cis-acting element |
| SiZIK4 | SETIT_026086mg | TATA-box | ATATAT | 329 | 341 | core promoter element |
| SiZIK4 | SETIT_026086mg | TATA-box | TATATA | 330 | 342 | core promoter element |
| SiZIK4 | SETIT_026086mg | TATA-box | TATA | 334 | 342 | core promoter element |
| SiZIK4 | SETIT_026086mg | TATA-box | ATATAT | 621 | 633 | core promoter element |
| SiZIK4 | SETIT_026086mg | TATA-box | TATA | 624 | 632 | core promoter element |
| SiZIK4 | SETIT_026086mg | TATA-box | ATTATA | 666 | 678 | core promoter element |
| SiZIK4 | SETIT_026086mg | TATA-box | TATAA | 668 | 678 | core promoter element |
| SiZIK4 | SETIT_026086mg | TATA-box | TATA | 670 | 678 | core promoter element |
| SiZIK4 | SETIT_026086mg | TATA-box | TACATAAA | 941 | 957 | core promoter element |
| SiZIK4 | SETIT_026086mg | G-box | CACGAC | 1625 | 1637 | light responsive element |
| SiZIK4 | SETIT_026086mg | GT1-motif | GGTTAA | 716 | 728 | light responsive element |
| SiZIK4 | SETIT_026086mg | Gap-box | CAAATGAA(A/G)A | 48 | 66 | light responsive element |
| SiZIK4 | SETIT_026086mg | CAT-box | GCCACT | 303 | 315 | meristem expression regulatory element |
| SiZIK4 | SETIT_026086mg | CAT-box | GCCACT | 1207 | 1219 | meristem expression regulatory element |
| SiZIK4 | SETIT_026086mg | MBSI | aaaAaaC(G/C)GTTA | 704.5 | 725.5 | MYB binding site involved in flavonoid biosynthetic genes regulation |
| SiZIK4 | SETIT_026086mg | TCA-element | TCAGAAGAGG | 1765 | 1783 | salicylic acid response element |
| SiZIK4 | SETIT_026086mg | O2-site | GATGA(C/T)(A/G)TG(A/G) | 1125 | 1141 | Zein metabolism regulatory elements |
| SiZIK5 | SETIT_009574mg | ARE | AAACCA | 82 | 94 | anaerobic inducing element |
| SiZIK5 | SETIT_009574mg | ARE | AAACCA | 118 | 130 | anaerobic inducing element |
| SiZIK5 | SETIT_009574mg | AuxRR-core | GGTCCAT | 1911 | 1925 | auxin response element |
| SiZIK5 | SETIT_009574mg | CAAT-box | CAAAT | 39 | 49 | common cis-acting element |
| SiZIK5 | SETIT_009574mg | CAAT-box | CAAAT | 78 | 88 | common cis-acting element |
| SiZIK5 | SETIT_009574mg | CAAT-box | CCAAT | 97 | 107 | common cis-acting element |
| SiZIK5 | SETIT_009574mg | CAAT-box | CAAAT | 114 | 124 | common cis-acting element |
| SiZIK5 | SETIT_009574mg | CAAT-box | CCAAT | 133 | 143 | common cis-acting element |
| SiZIK5 | SETIT_009574mg | CAAT-box | CCAAT | 371 | 381 | common cis-acting element |
| SiZIK5 | SETIT_009574mg | CAAT-box | CAAAT | 537 | 547 | common cis-acting element |
| SiZIK5 | SETIT_009574mg | CAAT-box | CCAAT | 572 | 582 | common cis-acting element |
| SiZIK5 | SETIT_009574mg | CAAT-box | CCAAT | 728 | 738 | common cis-acting element |
| SiZIK5 | SETIT_009574mg | CAAT-box | CCAAT | 828 | 838 | common cis-acting element |
| SiZIK5 | SETIT_009574mg | CAAT-box | CAAAT | 842 | 852 | common cis-acting element |
| SiZIK5 | SETIT_009574mg | CAAT-box | CCAAT | 914 | 924 | common cis-acting element |
| SiZIK5 | SETIT_009574mg | CAAT-box | CAAAT | 1028 | 1038 | common cis-acting element |
| SiZIK5 | SETIT_009574mg | CAAT-box | CAAAT | 1085 | 1095 | common cis-acting element |
| SiZIK5 | SETIT_009574mg | CAAT-box | CAAAT | 1175 | 1185 | common cis-acting element |
| SiZIK5 | SETIT_009574mg | CAAT-box | CCAAT | 1477 | 1487 | common cis-acting element |
| SiZIK5 | SETIT_009574mg | CAAT-box | CAACCAACTCC | 1531 | 1551 | common cis-acting element |
| SiZIK5 | SETIT_009574mg | CAAT-box | CAAAT | 1569 | 1579 | common cis-acting element |
| SiZIK5 | SETIT_009574mg | TATA-box | ATATAT | 387 | 399 | core promoter element |
| SiZIK5 | SETIT_009574mg | TATA-box | TATA | 390 | 398 | core promoter element |
| SiZIK5 | SETIT_009574mg | TATA-box | TATA | 485 | 493 | core promoter element |
| SiZIK5 | SETIT_009574mg | GC-motif | CCCCCG | 1364 | 1376 | hypoxia-specifically induced elements |
| SiZIK5 | SETIT_009574mg | G-box | TAACACGTAG | 523 | 541 | light responsive element |
| SiZIK5 | SETIT_009574mg | G-box | CACGAC | 1635 | 1647 | light responsive element |
| SiZIK5 | SETIT_009574mg | Sp1 | GGGCGG | 1751 | 1763 | light responsive element |
| SiZIK5 | SETIT_009574mg | TCT-motif | TCTTAC | 242 | 254 | light responsive element |
| SiZIK5 | SETIT_009574mg | AE-box | AGAAACAA | 1272 | 1288 | light responsive element |
| SiZIK5 | SETIT_009574mg | CGTCA-motif | CGTCA | 635 | 645 | MeJA response regulatory element |
| SiZIK5 | SETIT_009574mg | CGTCA-motif | CGTCA | 1692 | 1702 | MeJA response regulatory element |
| SiZIK5 | SETIT_009574mg | TGACG-motif | TGACG | 635 | 645 | MeJA response regulatory element |
| SiZIK5 | SETIT_009574mg | TGACG-motif | TGACG | 1692 | 1702 | MeJA response regulatory element |
| SiZIK5 | SETIT_009574mg | CAT-box | GCCACT | 1 | 13 | meristem expression regulatory element |
| SiZIK5 | SETIT_009574mg | MBS | CAACTG | 1318 | 1330 | MYB binding site involved in drought-inducibility |
| SiZIK5 | SETIT_009574mg | TCA-element | CCATCTTTTT | 352 | 370 | salicylic acid response element |
| SiZIK5 | SETIT_009574mg | TCA-element | TCAGAAGAGG | 1773 | 1791 | salicylic acid response element |
| SiZIK5 | SETIT_009574mg | O2-site | GTTGACGTGA | 1317 | 1335 | Zein metabolism regulatory elements |
| SiZIK6 | SETIT_027310mg | ABRE | ACGTG | 1737 | 1747 | abscisic acid response element |
| SiZIK6 | SETIT_027310mg | ABRE | CACGTG | 1798 | 1810 | abscisic acid response element |
| SiZIK6 | SETIT_027310mg | ABRE | ACGTG | 1800 | 1810 | abscisic acid response element |
| SiZIK6 | SETIT_027310mg | ARE | AAACCA | 1289 | 1301 | anaerobic inducing element |
| SiZIK6 | SETIT_027310mg | AT-rich element | ATAGAAATCAA | 1344 | 1364 | ATBP-1 binding site |
| SiZIK6 | SETIT_027310mg | TGA-element | AACGAC | 606 | 618 | auxin-responsive element |
| SiZIK6 | SETIT_027310mg | CAAT-box | CCAAT | 106 | 116 | common cis-acting element |
| SiZIK6 | SETIT_027310mg | CAAT-box | CAAAT | 285 | 295 | common cis-acting element |
| SiZIK6 | SETIT_027310mg | CAAT-box | CAAAT | 295 | 305 | common cis-acting element |
| SiZIK6 | SETIT_027310mg | CAAT-box | CAAAT | 336 | 346 | common cis-acting element |
| SiZIK6 | SETIT_027310mg | CAAT-box | CAAAT | 343 | 353 | common cis-acting element |
| SiZIK6 | SETIT_027310mg | CAAT-box | CCAAT | 527 | 537 | common cis-acting element |
| SiZIK6 | SETIT_027310mg | CAAT-box | CCAAT | 825 | 835 | common cis-acting element |
| SiZIK6 | SETIT_027310mg | CAAT-box | CAAAT | 997 | 1007 | common cis-acting element |
| SiZIK6 | SETIT_027310mg | CAAT-box | CAAAT | 1112 | 1122 | common cis-acting element |
| SiZIK6 | SETIT_027310mg | TATA-box | TATAA | 43 | 53 | core promoter element |
| SiZIK6 | SETIT_027310mg | TATA-box | TATA | 45 | 53 | core promoter element |
| SiZIK6 | SETIT_027310mg | TATA-box | TACAAAA | 163 | 177 | core promoter element |
| SiZIK6 | SETIT_027310mg | TATA-box | TACAAAA | 328 | 342 | core promoter element |
| SiZIK6 | SETIT_027310mg | TATA-box | TATA | 1035 | 1043 | core promoter element |
| SiZIK6 | SETIT_027310mg | TATA-box | TACAAAA | 1043 | 1057 | core promoter element |
| SiZIK6 | SETIT_027310mg | TATA-box | ATTATA | 1054 | 1066 | core promoter element |
| SiZIK6 | SETIT_027310mg | TATA-box | TATAA | 1056 | 1066 | core promoter element |
| SiZIK6 | SETIT_027310mg | TATA-box | TATA | 1058 | 1066 | core promoter element |
| SiZIK6 | SETIT_027310mg | TATA-box | TATAAAA | 1208 | 1222 | core promoter element |
| SiZIK6 | SETIT_027310mg | TATA-box | TATAAA | 1210 | 1222 | core promoter element |
| SiZIK6 | SETIT_027310mg | TATA-box | TATAA | 1212 | 1222 | core promoter element |
| SiZIK6 | SETIT_027310mg | TATA-box | TATA | 1214 | 1222 | core promoter element |
| SiZIK6 | SETIT_027310mg | TATA-box | TACAAAA | 1533 | 1547 | core promoter element |
| SiZIK6 | SETIT_027310mg | TATA-box | TATAA | 1578 | 1588 | core promoter element |
| SiZIK6 | SETIT_027310mg | TATA-box | TATA | 1580 | 1588 | core promoter element |
| SiZIK6 | SETIT_027310mg | TATA-box | TACATAAA | 1622 | 1638 | core promoter element |
| SiZIK6 | SETIT_027310mg | GARE-motif | TCTGTTG | 906 | 920 | gibberellin-responsive element |
| SiZIK6 | SETIT_027310mg | GC-motif | CCCCCG | 374 | 386 | hypoxia-specifically induced elements |
| SiZIK6 | SETIT_027310mg | GC-motif | CCCCCG | 1832 | 1844 | hypoxia-specifically induced elements |
| SiZIK6 | SETIT_027310mg | G-box | CACGAC | 205 | 217 | light responsive element |
| SiZIK6 | SETIT_027310mg | G-box | CACGAC | 221 | 233 | light responsive element |
| SiZIK6 | SETIT_027310mg | G-box | CACGTC | 1735 | 1747 | light responsive element |
| SiZIK6 | SETIT_027310mg | G-box | CACGTG | 1798 | 1810 | light responsive element |
| SiZIK6 | SETIT_027310mg | G-Box | CACGTG | 1798 | 1810 | light responsive element |
| SiZIK6 | SETIT_027310mg | ATC-motif | AGCTATCCA | 1904 | 1922 | light responsive element |
| SiZIK6 | SETIT_027310mg | Box 4 | ATTAAT | 1153 | 1165 | light responsive element |
| SiZIK6 | SETIT_027310mg | Box 4 | ATTAAT | 1240 | 1252 | light responsive element |
| SiZIK6 | SETIT_027310mg | GATA-motif | AAGATAAGATT | 789 | 809 | light responsive element |
| SiZIK6 | SETIT_027310mg | LTR | CCGAAA | 833 | 845 | low temperature response element |
| SiZIK6 | SETIT_027310mg | TGACG-motif | TGACG | 789 | 799 | MeJA response regulatory element |
| SiZIK6 | SETIT_027310mg | CGTCA-motif | CGTCA | 789 | 799 | MeJA response regulatory element |
| SiZIK6 | SETIT_027310mg | CAT-box | GCCACT | 1358 | 1370 | meristem expression regulatory element |
| SiZIK6 | SETIT_027310mg | MBS | CAACTG | 232 | 244 | MYB binding site involved in drought-inducibility |
| SiZIK6 | SETIT_027310mg | MBS | CAACTG | 1399 | 1411 | MYB binding site involved in drought-inducibility |
| SiZIK6 | SETIT_027310mg | CCAAT-box | CAACGG | 268 | 280 | MYBHv1 binding site |
| SiZIK6 | SETIT_027310mg | A-box | CCGTCC | 460 | 472 | promoter and enhancer cis-acting regulatory elements |
| SiZIK7 | SETIT_032842mg | ABRE | CACGTG | 533 | 545 | abscisic acid response element |
| SiZIK7 | SETIT_032842mg | ABRE | ACGTG | 535 | 545 | abscisic acid response element |
| SiZIK7 | SETIT_032842mg | ABRE | GACACGTACGT | 1085 | 1105 | abscisic acid response element |
| SiZIK7 | SETIT_032842mg | ABRE | CGCACGTGTC | 1087 | 1105 | abscisic acid response element |
| SiZIK7 | SETIT_032842mg | ABRE | ACGTG | 1094 | 1104 | abscisic acid response element |
| SiZIK7 | SETIT_032842mg | ABRE | CACGTG | 1476 | 1488 | abscisic acid response element |
| SiZIK7 | SETIT_032842mg | ABRE | ACGTG | 1478 | 1488 | abscisic acid response element |
| SiZIK7 | SETIT_032842mg | ABRE | ACGTG | 1737 | 1747 | abscisic acid response element |
| SiZIK7 | SETIT_032842mg | ABRE | GCCGCGTGGC | 1747 | 1765 | abscisic acid response element |
| SiZIK7 | SETIT_032842mg | ABRE | ACGTG | 1816 | 1826 | abscisic acid response element |
| SiZIK7 | SETIT_032842mg | ARE | AAACCA | 46 | 58 | anaerobic inducing element |
| SiZIK7 | SETIT_032842mg | ARE | AAACCA | 1656 | 1668 | anaerobic inducing element |
| SiZIK7 | SETIT_032842mg | ARE | AAACCA | 1786 | 1798 | anaerobic inducing element |
| SiZIK7 | SETIT_032842mg | TGA-element | AACGAC | 790 | 802 | auxin-responsive element |
| SiZIK7 | SETIT_032842mg | TGA-element | AACGAC | 1911 | 1923 | auxin-responsive element |
| SiZIK7 | SETIT_032842mg | CAAT-box | CCAAT | 125 | 135 | common cis-acting element |
| SiZIK7 | SETIT_032842mg | CAAT-box | CCAAT | 939 | 949 | common cis-acting element |
| SiZIK7 | SETIT_032842mg | CAAT-box | CAAAT | 968 | 978 | common cis-acting element |
| SiZIK7 | SETIT_032842mg | CAAT-box | CAAAT | 1017 | 1027 | common cis-acting element |
| SiZIK7 | SETIT_032842mg | CAAT-box | CCAAT | 1183 | 1193 | common cis-acting element |
| SiZIK7 | SETIT_032842mg | CAAT-box | CAAAT | 1432 | 1442 | common cis-acting element |
| SiZIK7 | SETIT_032842mg | CAAT-box | CCAAT | 1492 | 1502 | common cis-acting element |
| SiZIK7 | SETIT_032842mg | CAAT-box | CAAAT | 1677 | 1687 | common cis-acting element |
| SiZIK7 | SETIT_032842mg | CAAT-box | CAAAT | 1804 | 1814 | common cis-acting element |
| SiZIK7 | SETIT_032842mg | CAAT-box | CAACCAACTCC | 1931 | 1951 | common cis-acting element |
| SiZIK7 | SETIT_032842mg | TATA-box | TACAAAA | 1525 | 1539 | core promoter element |
| SiZIK7 | SETIT_032842mg | TATA-box | TATA | 1987 | 1995 | core promoter element |
| SiZIK7 | SETIT_032842mg | GC-motif | CCCCCG | 503 | 515 | hypoxia-specifically induced elements |
| SiZIK7 | SETIT_032842mg | ACE | GCGACGTACC | 1916 | 1934 | light responsive element |
| SiZIK7 | SETIT_032842mg | G-Box | CACGTG | 533 | 545 | light responsive element |
| SiZIK7 | SETIT_032842mg | G-Box | CACGTG | 1476 | 1488 | light responsive element |
| SiZIK7 | SETIT_032842mg | G-Box | CACGTT | 1736 | 1748 | light responsive element |
| SiZIK7 | SETIT_032842mg | G-box | CACGTG | 533 | 545 | light responsive element |
| SiZIK7 | SETIT_032842mg | G-box | CACGAC | 996 | 1008 | light responsive element |
| SiZIK7 | SETIT_032842mg | G-box | CACGTC | 1092 | 1104 | light responsive element |
| SiZIK7 | SETIT_032842mg | G-box | CACGTG | 1476 | 1488 | light responsive element |
| SiZIK7 | SETIT_032842mg | G-box | TACGTG | 1814 | 1826 | light responsive element |
| SiZIK7 | SETIT_032842mg | GT1-motif | GGTTAA | 1147 | 1159 | light responsive element |
| SiZIK7 | SETIT_032842mg | Sp1 | GGGCGG | 1133 | 1145 | light responsive element |
| SiZIK7 | SETIT_032842mg | GATA-motif | AAGGATAAGG | 1052 | 1072 | light responsive element |
| SiZIK7 | SETIT_032842mg | I-box | ccttatcct | 1054 | 1072 | light responsive element |
| SiZIK7 | SETIT_032842mg | LTR | CCGAAA | 764 | 776 | low temperature response element |
| SiZIK7 | SETIT_032842mg | CGTCA-motif | CGTCA | 690 | 700 | MeJA response regulatory element |
| SiZIK7 | SETIT_032842mg | TGACG-motif | TGACG | 690 | 700 | MeJA response regulatory element |
| SiZIK7 | SETIT_032842mg | CAT-box | GCCACT | 1028 | 1040 | meristem expression regulatory element |
| SiZIK7 | SETIT_032842mg | CCAAT-box | CAACGG | 757 | 769 | MYBHv1 binding site |
| SiZIK7 | SETIT_032842mg | A-box | CCGTCC | 742 | 754 | promoter and enhancer cis-acting regulatory elements |
| SiZIK7 | SETIT_032842mg | A-box | CCGTCC | 1870 | 1882 | promoter and enhancer cis-acting regulatory elements |
| SiZIK8 | SETIT_029157mg | ABRE | TACGGTC | 1450 | 1464 | abscisic acid response element |
| SiZIK8 | SETIT_029157mg | AuxRR-core | GGTCCAT | 746 | 760 | auxin response element |
| SiZIK8 | SETIT_029157mg | TGA-element | AACGAC | 1302 | 1314 | auxin-responsive element |
| SiZIK8 | SETIT_029157mg | CAAT-box | CAAAT | 84 | 94 | common cis-acting element |
| SiZIK8 | SETIT_029157mg | CAAT-box | CCAAT | 206 | 216 | common cis-acting element |
| SiZIK8 | SETIT_029157mg | CAAT-box | CAAAT | 345 | 355 | common cis-acting element |
| SiZIK8 | SETIT_029157mg | CAAT-box | CCAAT | 352 | 362 | common cis-acting element |
| SiZIK8 | SETIT_029157mg | CAAT-box | CAAAT | 359 | 369 | common cis-acting element |
[truncated: 138,034 more chars]
