# Supplementary figures and images for: Identification and expression analysis of MAPK cascade gene family in foxtail millet (Setaria italica)
Source: Plant Signal Behav. 2023 Aug 16;18(1):2246228. doi: 10.1080/15592324.2023.2246228 (PMC10435010; doi:10.1080/15592324.2023.2246228)

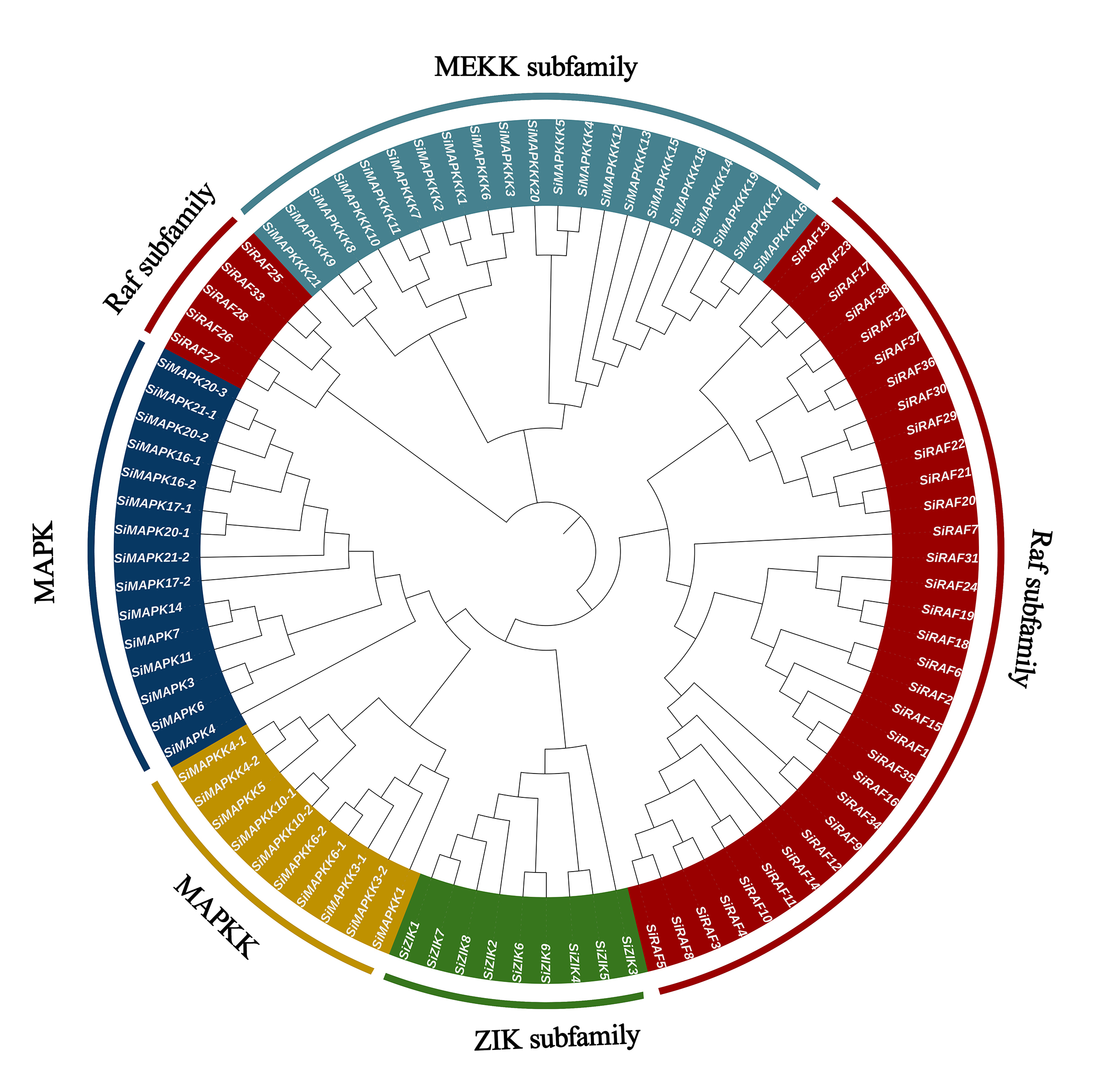

Supplement: Supplemental Material [file KPSB_A_2246228_SM9810.zip › Fig. S1.jpg]

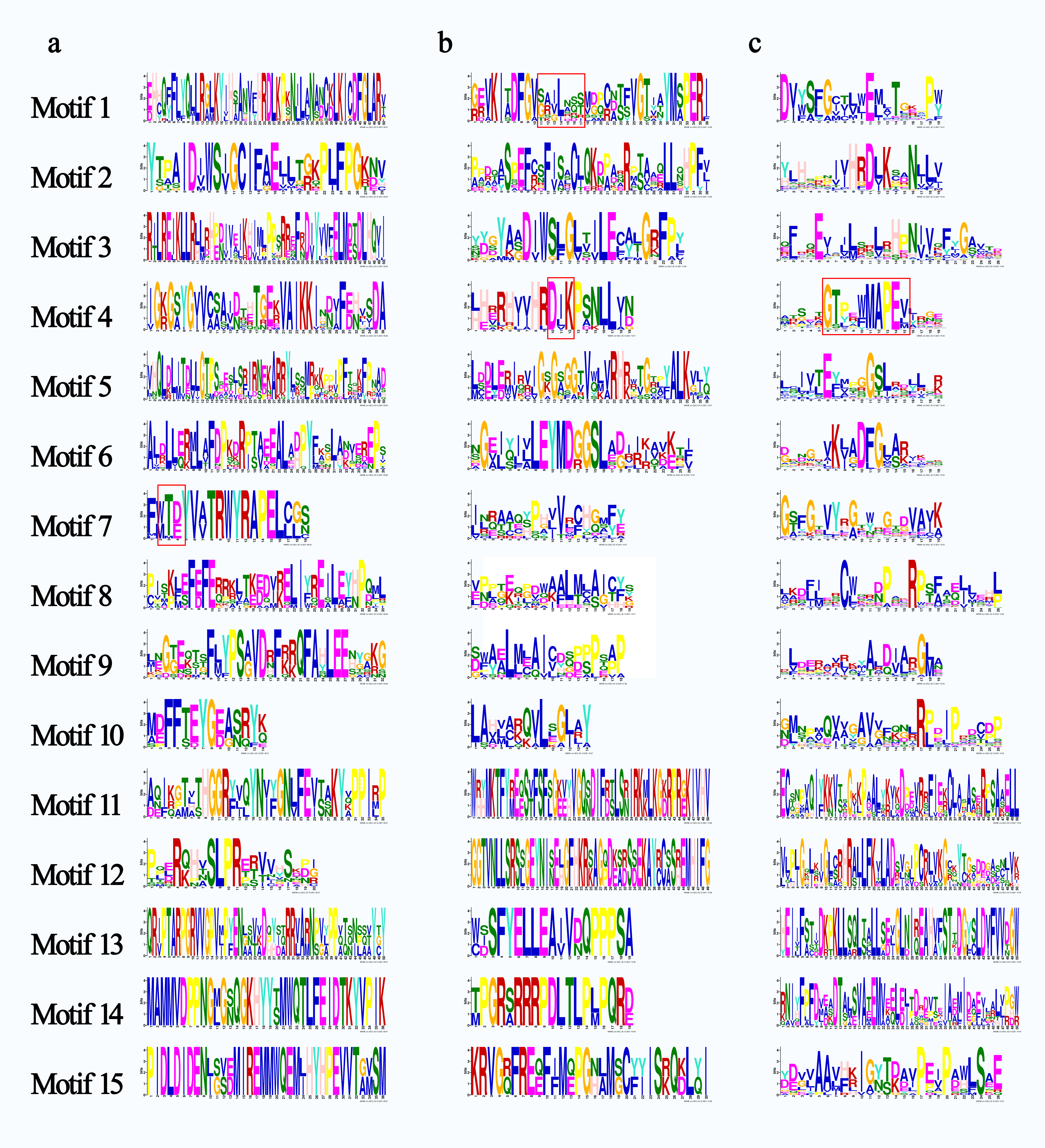

Supplement: Supplemental Material [file KPSB_A_2246228_SM9810.zip › Fig. S2.jpg]
